# Supplementary material for: Migratory allylic arylation of 1,n-enols enabled by nickel catalysis
Source: Nat Commun. 2023 Jun 7;14:3308. doi: 10.1038/s41467-023-38865-z (PMC10247813; doi:10.1038/s41467-023-38865-z)
Supplement: Supplementary file 1 — Supplementary Information [file 41467_2023_38865_MOESM1_ESM.pdf]

Supplementary Information

Migratory Allylic Arylation of 1,n-Enols Enabled by Nickel Catalysis

Dan Zhao[1,§], Bing Xu[1,2,§], and Can Zhu[1]*

[1]Department of Chemistry, Fudan University, 2005 Songhu Road, Shanghai 200438, China;

[2]Zhuhai Fudan Innovation Institute, Zhuhai 519000, China

[§]These authors contributed equally: Dan Zhao, Bing Xu.

*Email: zhucan@fudan.edu.cn (Can Zhu)

Table of contents

1. Supplementary Methods S2

2. Supplementary Discussion S3

2.1. General Procedure for the Synthesis of Enols S3

2.2. General procedure the migratory dehydroxylative allylic arylation of 1,n-enaols S30

2.3. Regio- and stereoconvergent reactions S48

2.4. Mechanistic studies S61

3. Supplementary Notes S65

3.1. Computational Details S65

3.2. Supplementary Figures S66

4. Supplementary reference S156

1. Supplementary Methods

Unless otherwise noted, all reactions were carried out in standard Schlenk techniques with magnetic stirring bar. Aryl boronic acids should be recrystallized to remove possible impurities, and confirmed by ^{1}H NMR analysis before use. Other materials obtained from commercial suppliers were used directly without further purification. ^{1}H NMR spectra were recorded on a BRUKER 400 (400 MHz) spectrometer in $CDCl_3$. Chemical shifts are reported in ppm, using the residual solvent peak in $CDCl_3$ (H = 7.26) as internal standard, Data are reported as follows: chemical shift, multiplicity (s = singlet, d = doublet, dd = doublet of doublet, t = triplet, q = quartet, m = multiplet), coupling constants (Hz), and integration. ^{13}C NMR spectra were recorded on a BRUKER 400 (100 MHz) spectrometer in $CDCl_3$ with complete proton decoupling. Chemical shifts are reported in ppm with the deuterium solvent as the internal standard ($CDCl_3$: 77.0 ppm). HRMS were recorded using EI and ESI techniques.

Anhydrous tetrahydrofuran (THF), toluene, acetonitrile and 1,2-dichloroethane (DCE) were obtained from solvent purification system; Unless otherwise noted, analytical grade solvents and commercially available reagents were used directly.

Reactions were monitored by thin layer chromatography (TLC) using pre-coated silica gel plates. Flash column chromatography was performed on silica gel 60 (particle size 300-400 mesh ASTM, purchased from Yantai, China).

2. Supplementary Discussion

2.1. General Procedure for the Synthesis of Enols

*Synthesis of 1-phenylpent-4-en-2-ol (**1c**)*[1]

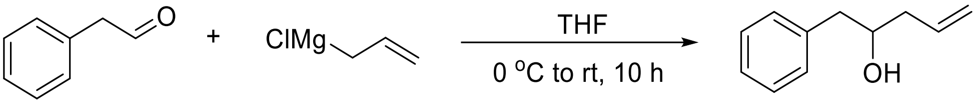

1.0 equiv. **1c**: 30%

To a 100 mL three-necked flask containing a solution of 2-phenylacetaldehyde (3.603 g, 30 mmol) in dry THF (30 mL) at 0 °C was slowly added allylmagnesium chloride (30 mL, 30 mmol, 1.0 M in THF). The solution was stirred at 0 °C for 1 h, then mixture was allowed to gradually warm to room temperature for 9 h. After the reaction was complete, the mixture was quenched with saturated NH_4Cl (aq.) carefully at 0 °C, extracted with EtOAc (10 mL×3). The combined organic layers were washed with brine (20 mL), dried over Na_2SO_4, filtered and concentrated in vacuo. The crude product was purified by column chromatography (eluent: petroleum ether/ethyl acetate = 30/1) to afford **1c** (1.460 g, 30% yield), colorless oil. ^{1}H NMR (400 MHz, Chloroform-*d*) δ 7.26-7.22 (m, 2H), 7.18-7.14 (m, 3H), 5.85-5.72 (m, 1H), 5.11-5.06 (m, 2H), 3.85-3.78 (m, 1H), 2.75 (dd, *J* = 13.6, 4.9 Hz, 1H), 2.65 (dd, *J* = 13.6, 7.9 Hz, 1H), 2.30-2.21 (m, 1H), 2.21-2.11 (m, 1H), 1.63 (s, 1H); ^{13}C NMR (100 MHz, Chloroform-*d*) δ 138.4, 134.7, 129.4, 128.5, 126.5, 118.1, 71.7, 43.3, 41.2.

*Synthesis of 1-phenylhex-5-en-2-ol (**1d**)*[2]

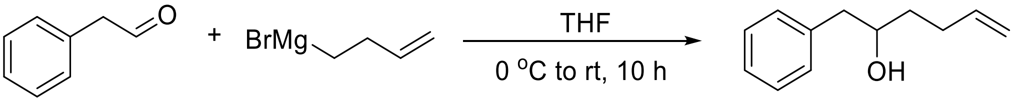

1.5 equiv. **1d**: 79%

To a 100 mL three-necked flask containing a solution of 2-phenylacetaldehyde (1.201 g, 10 mmol) in dry THF (20 mL) at 0 °C was slowly added but-3-en-1-ylmagnesium bromide (30 mL, 15 mmol, 0.5 M in THF). The solution was stirred at 0 °C for 1 h, then mixture was allowed to gradually warm to room temperature for 9 h. After the reaction was complete, the mixture was quenched with saturated NH_4Cl (aq.) carefully at 0 °C, extracted with EtOAc (10 mL×3). The combined organic layers were

washed with brine (20 mL), dried over Na_2SO_4, filtered and concentrated in vacuo. The crude product was purified by column chromatography (eluent: petroleum ether/ethyl acetate = 40/1) to afford **1d** (1.392 g, 79% yield), colorless oil; ^{1}H NMR (400 MHz, Chloroform-*d*) δ 7.37-7.33 (m, 2H), 7.29-7.21 (m, 3H), 5.87 (ddt, *J* = 16.9, 10.2, 6.6 Hz, 1H), 5.11-4.99 (m, 2H), 3.90-3.84 (m, 1H), 2.86 (dd, *J* = 13.5, 4.3 Hz, 1H), 2.70 (dd, *J* = 13.5, 8.4 Hz, 1H), 2.34-2.16 (m, 2H), 1.72-1.59 (m, 3H); ^{13}C NMR (100 MHz, Chloroform-*d*) δ 138.5, 138.4, 129.4, 128.5, 126.5, 114.8, 72.1, 44.0, 35.8, 30.1.

*Synthesis of (E)-1-phenylpent-2-en-1-ol (**1e**)*[3]

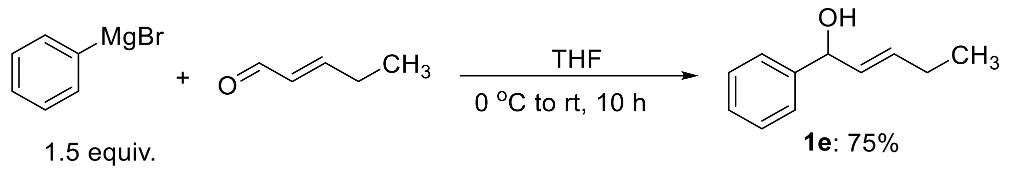

PhMgBr (15 mL, 1.0 M in THF, 15 mmol) was added dropwise to a stirred solution of (*E*)-pent-2-enal (0.841 g, 10 mmol) in THF (20 mL) at 0 °C under N_2. The solution was stirred at 0 °C for 1 h, then the mixture was allowed to gradually warm to room temperature and stirred for additional 9 h. The reaction was quenched with saturated NH_4Cl (aq.). The layers were separated, and the aqueous layer was extracted with EtOAc (10 mL×3). The combined organic layers were dried over Na_2SO_4, evaporated under reduced pressure, and then purified via column chromatography (eluent: petroleum ether/ethyl acetate = 40/1) to afford **1e** (1.210 g, 75% yield), colorless oil; ^{1}H NMR (400 MHz, Chloroform-*d*) δ 7.40-7.30 (m, 4H), 7.31-7.22 (m, 1H), 5.80 (dt, *J* = 15.0, 6.0 Hz, 1H), 5.65 (ddt, *J* = 15.3, 6.9, 1.5 Hz, 1H), 5.15 (dd, *J* = 6.9, 2.9 Hz, 1H), 2.11-2.04 (m, 2H), 2.00-1.97 (m, 1H), 1.00 (t, *J* = 7.4 Hz, 3H); ^{13}C NMR (100 MHz, Chloroform-*d*) δ 143.4, 134.3, 131.3, 128.4, 127.4, 126.1, 75.2, 25.2, 13.3.

*Synthesis of 1-phenylhex-4-en-3-ol (**1f**)*[4]

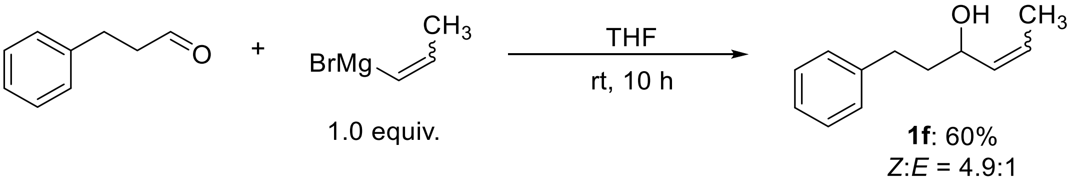

To a 100 mL three-necked flask containing a solution of 3-phenylpropanal (1.342 g, 10 mmol) in dry THF (30 mL) at room temperature was slowly added prop-1-en-1-ylmagnesium bromide (20 mL, 10 mmol, 0.5 M in THF). The solution was stirred at room temperature for 10 h. After the reaction was complete, the mixture was quenched with saturated NH_4Cl (aq.) carefully, extracted with EtOAc (10 mL×3). The combined organic layers were washed with brine (20 mL), dried over Na_2SO_4, filtered and concentrated in vacuo. The crude product was purified by column chromatography (eluent: petroleum ether/ethyl acetate = 30/1) to afford **1f** (1.057 g, 60% yield, *Z*:*E* = 4.9:1 determined by ^{1}H NMR analysis), colorless oil; ^{1}H NMR (400 MHz, Chloroform-*d*) δ 7.31-7.26 (m, 2H), 7.24-7.17 (m, 3H), 5.73-5.52 (m, 1H), 5.54-5.41 (m, 1H), 4.53-4.47 (*Z*, m, 0.83H), 4.10-4.04 (*E*, m, 0.17H), 2.75-2.63 (m, 2H), 2.00-1.74 (m, 2H), 1.73-1.65 (m, 3H), 1.44 (s, 1H); ^{13}C NMR (100 MHz, Chloroform-*d*) δ 141.9, 134.0, 133.2, 128.42, 128.38, 128.3, 127.2, 126.7, 125.79, 125.76, 72.4, 66.8, 38.9, 38.7, 31.8, 31.6, 17.7, 13.3.

*Synthesis of 1-phenylpent-3-en-1-ol (**1g**)*[5]

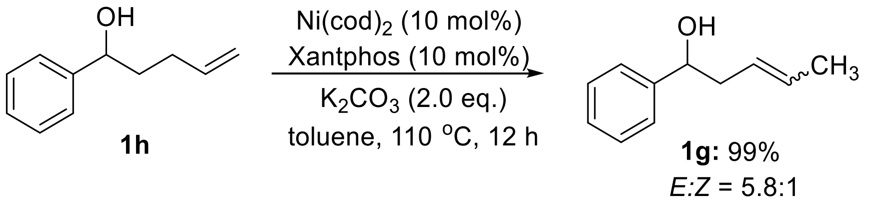

Under a nitrogen atmosphere, to a mixture of $Ni(cod)_2$ (5.5 mg, 0.02 mmol), Xantphos (11.6 mg, 0.02 mmol), K_2CO_3 (55.0 mg, 0.4 mmol) was added a solution of 1-phenylpent-4-en-1-ol **1h** (32.4 mg, 0.2 mmol) in toluene (1.0 mL). The reaction was sealed and stirred at 110 °C for 12 h. Subsequently, the reaction was cooled down to room temperature and the mixture was evaporated and purified via column chromatography on silica gel (eluent: petroleum ether/ethyl acetate = 30/1) afforded afford **1g** (32.1 mg, 99% yield, *Z*:*E* = 5.8:1 determined by ^{1}H NMR analysis), colorless oil; ^{1}H NMR (400 MHz, Chloroform-*d*) δ 7.41--7.31 (m, 4H), 7.35-7.23 (m, 1H), 5.69-5.54 (m, 1H), 5.50-5.37 (m, 1H), 4.76-4.64 (m, 1H), 2.63-2.37 (m, 2H), 2.13 (s, 1H),

1.70 (*E*, dd, J = 6.3, 1.3 Hz, 2.56H), 1.61 (*Z*, dd, J = 6.8, 0.9 Hz, 0.44H); ^{13}C NMR (100 MHz, Chloroform-*d*) δ 144.0, 129.5, 128.4, 128.3, 127.7, 127.5, 127.4, 126.8, 125.81, 125.77, 125.65, 73.8, 73.4, 42.8, 36.9, 18.0, 13.0.

*Synthesis of alkenyl alcohols (**1h-1m**)*

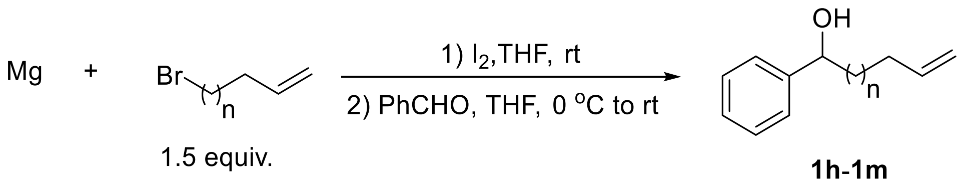

Alkyl bromide (15.0 mmol) in THF (15 mL) was partially added to cover the mixture of activated magnesium turnings (437.6 mg, 18.0 mmol) and a small crystal of iodine under N_2 atmosphere. Heat the mixture briefly with a hair dryer to initiate the reaction (the fading of the solution indicated reaction initiation). Then the rest of the alkyl bromide solution was added dropwise. The reaction was stirred for 2 h. Then the Grignard reagent were added dropwise to benzaldehyde solution (10.0 mmol, 1M in THF) with a syringe at 0 °C under N_2 atmosphere. The solution was stirred at 0 °C for 1 h, then the mixture was allowed to gradually warm to room temperature and stirred for additional 9 h. The reaction was quenched with saturated NH_4Cl (aq.). The layers were separated, and the aqueous layer was extracted with EtOAc (10 mL×3). The combined organic layers were dried over Na_2SO_4, evaporated under reduced pressure, and then purified via column chromatography (eluent: petroleum ether/ethyl acetate = 30/1) to afford corresponding alcohols.

*1-phenylpent-4-en-1-ol (**1h**)*[6]

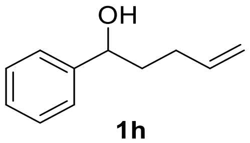

1h

70% isolated yield, colorless oil; ^{1}H NMR (400 MHz, Chloroform-*d*) δ 7.35-7.32 (m, 4H), 7.31-7.26 (m, 1H), 5.84 (ddt, J = 16.9, 10.1, 6.6 Hz, 1H), 5.10-4.96 (m, 2H), 4.68 (dd, J = 7.0, 5.4 Hz, 1H), 2.21-2.04 (m, 2H), 1.97 (s, 1H), 1.96-1.72 (m, 2H); ^{13}C

NMR (100 MHz, Chloroform-*d*) δ 144.6, 138.2, 128.4, 127.5, 125.9, 114.9, 74.0, 38.0, 30.0.

*1-phenylhex-5-en-1-ol (**1i**)*[7]

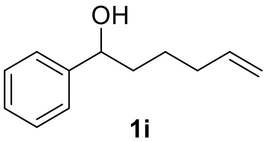

1i

72% isolated yield, colorless oil; ^{1}H NMR (400 MHz, Chloroform-*d*) δ 7.36-7.26 (m, 4H), 7.29-7.20 (m, 1H), 5.76 (ddt, *J* = 16.9, 10.1, 6.6 Hz, 1H), 5.06-4.86 (m, 2H), 4.61 (dd, *J* = 7.4, 5.9 Hz, 1H), 2.24 (s, 1H), 2.08-2.02 (m, 2H), 1.84-1.61 (m, 2H), 1.55-1.44 (m, 1H), 1.40-1.29 (m, 1H); ^{13}C NMR (100 MHz, Chloroform-*d*) δ 144.8, 138.5, 128.3, 127.4, 125.8, 114.6, 74.4, 38.4, 33.5, 25.0.

*1-phenylhept-6-en-1-ol (**1j**)*[8]

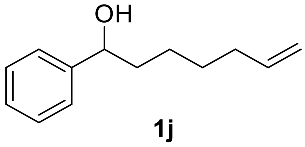

1j

68% isolated yield, colorless oil; ^{1}H NMR (400 MHz, Chloroform-*d*) δ 7.29-7.19 (m, 4H), 7.22-7.13 (m, 1H), 5.70 (ddt, *J* = 16.9, 10.2, 6.7 Hz, 1H), 4.95-4.80 (m, 2H), 4.53 (dd, *J* = 7.5, 5.8 Hz, 1H), 2.06 (s, 1H), 2.00-1.89 (m, 2H), 1.79-1.52 (m, 2H), 1.39-1.26 (m, 3H), 1.26-1.12 (m, 1H); ^{13}C NMR (100 MHz, Chloroform-*d*) δ 144.8, 138.8, 128.3, 127.4, 125.8, 114.3, 74.5, 38.8, 33.6, 28.7, 25.3.

*1-phenyloct-7-en-1-ol (**1k**)*[9]

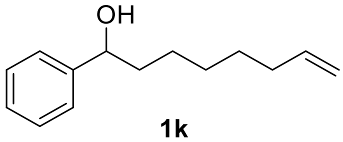

1k

65% isolated yield, colorless oil; ^{1}H NMR (400 MHz, Chloroform-*d*) δ 7.30-7.25 (m, 4H), 7.25-7.15 (m, 1H), 5.72 (ddt, *J* = 16.9, 10.1, 6.6 Hz, 1H), 5.01-4.80 (m, 2H), 4.58 (dd, *J* = 7.5, 5.8 Hz, 1H), 2.02-1.92 (m, 2H), 1.78 (s, 1H), 1.76-1.57 (m, 2H), 1.39-

1.15 (m, 6H); ^{13}C NMR (100 MHz, Chloroform-*d*) δ 144.9, 139.0, 128.4, 127.5, 125.9, 114.2, 74.6, 39.0, 33.7, 29.0, 28.8, 25.6.

*1-phenylnon-8-en-1-ol (**1l**)*[10]

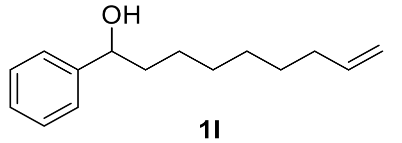

1l

60% isolated yield, colorless oil; ^{1}H NMR (400 MHz, Chloroform-*d*) δ 7.29-7.25 (m, 4H), 7.23-7.17 (m, 1H), 5.72 (ddt, *J* = 16.9, 10.2, 6.7 Hz, 1H), 5.03-4.78 (m, 2H), 4.58 (dd, *J* = 7.5, 5.8 Hz, 1H), 2.01-1.90 (m, 2H), 1.78 (s, 1H), 1.76-1.58 (m, 2H), 1.40-1.14 (m, 8H); ^{13}C NMR (100 MHz, Chloroform-*d*) δ 144.9, 139.1, 128.4, 127.5, 125.9, 114.2, 74.7, 39.1, 33.7, 29.3, 29.0, 28.8, 25.8.

*1-phenylundec-10-en-1-ol (**1m**)*[7]

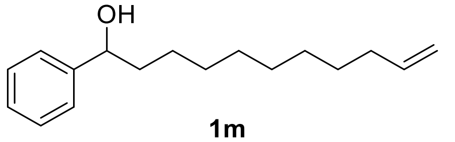

1m

55% isolated yield, colorless oil; ^{1}H NMR (400 MHz, Chloroform-*d*) δ 7.40-7.35 (m, 4H), 7.33-7.27 (m, 1H), 5.83 (ddt, *J* = 17.0, 10.2, 6.7 Hz, 1H), 5.13-4.87 (m, 2H), 4.68 (dd, *J* = 7.5, 5.8 Hz, 1H), 2.13-2.02 (m, 2H), 1.87 (s, 1H), 1.85-1.68 (m, 2H), 1.49-1.21 (m, 12H); ^{13}C NMR (100 MHz, Chloroform-*d*) δ 144.9, 139.2, 128.4, 127.5, 125.9, 114.1, 74.7, 39.1, 33.8, 29.5, 29.5, 29.4, 29.1, 28.9, 25.8.

*1-methoxy-4-((1E)-penta-1,3-dien-1-yl)benzene (**40**)*[11]

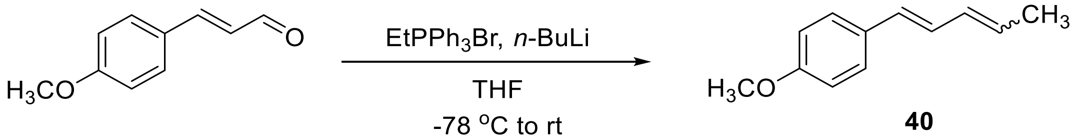

40
E:*Z* = 1:4

To a dry 100 mL round-bottom flask under N_2 were added ethyltriphenylphosphonium bromide (4.830 g, 13.0 mmol) and dry THF (30 mL). The flask was cooled to –78 °C and *n*-BuLi (14 mL, 14 mmol, 1.0 M in hexane) was added

dropwise. The reaction was allowed to warm to room temperature and stirred an additional 10 min before it was cooled to –78 °C. Then *trans*-4-methoxycinnamaldehyde (1.620 g, 10 mmol) was added slowly. The reaction was allowed to warm up to room temperature and stir overnight before being quenched with saturated NH_4Cl (aq.), extracted with EtOAc (10 mL×3). The combined organic layers were washed with brine (20 mL), dried over Na_2SO_4, filtered and concentrated in vacuo. The crude product was purified by column chromatography (eluent: petroleum ether/ethyl acetate = 200/1) to afford **40** (1.010 g, 58% yield, *E*:*Z* = 1:4 determined by ^{1}H NMR analysis), colorless oil; ^{1}H NMR (400 MHz, Chloroform-*d*) δ 7.38-7.30 (m, 2H), 7.00-6.60 (m, 3H), 6.50-6.36 (m, 1H), 6.25-6.14 (m, 1H), 5.83-5.74 (*E*, m, 0.2H), 5.59-5.51 (*Z*, m, 0.8H), 3.82-3.81 (m, 3H), 1.87-1.81 (m, 3H); ^{13}C NMR (100 MHz, Chloroform-*d*) δ 159.1, 132.0, 131.4, 130.5, 129.7, 129.2, 129.0, 127.5, 127.4, 127.2, 126.0, 122.3, 114.0, 55.3, 18.3, 13.6.

*Synthesis of 1-phenylpent-3-en-2-ol ((E)-**1a**)*

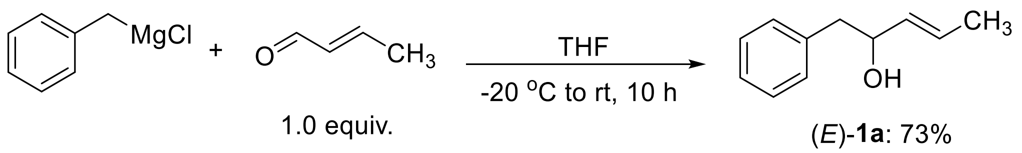

BnMgCl (30 mL, 1.0 M in THF, 30 mmol) was added dropwise to a stirred solution of crotonaldehyde (2.103 g, 30 mmol) in THF (20 mL) at -20 °C under N_2. The solution was stirred at -20 °C for 1 h, then the mixture was allowed to gradually warm to room temperature and stirred for additional 9 h. The reaction was quenched with saturated NH_4Cl (aq.). The layers were separated, and the aqueous layer was extracted with EtOAc (10 mL×3). The combined organic layers were dried over Na_2SO_4, evaporated under reduced pressure, and then purified via column chromatography (eluent: petroleum ether/ethyl acetate = 30/1) to afford (*E*)-**1a** (3.552 g, 73% yield), colorless oil. ^{1}H NMR (400 MHz, Chloroform-*d*) δ 7.33-7.29 (m, 2H), 7.25-7.21 (m, 3H), 5.72-5.63 (m, 1H), 5.59-5.52 (m, 1H), 4.31-4.26 (m, 1H), 2.85 (dd, *J* = 13.5, 5.0 Hz, 1H), 2.76 (dd, *J* = 13.6, 8.0 Hz, 1H), 1.69 (d, *J* = 6.3 Hz, 3H), 1.59 (s, 1H); ^{13}C NMR (100 MHz, Chloroform-*d*) δ 138.0, 133.2, 129.5, 128.4, 127.0, 126.4, 73.5, 44.1, 17.6;

HRMS (ESI) m/z calculated for $C_{11}H_{14}O$ $[M+Na]^+$: 185.0937, found: 185.0933.

*Synthesis of 1-phenylpent-3-en-2-ol (**1a**)*

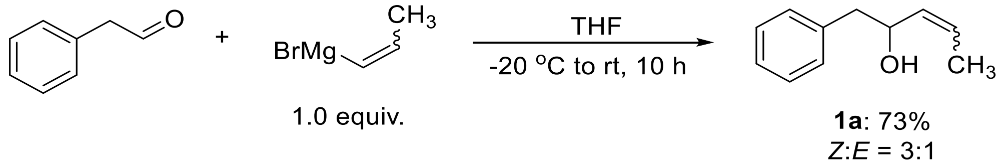

To a 100 mL three-necked flask containing a solution of 2-phenylacetaldehyde (1.201 g, 10 mmol) in dry THF (30 mL) at -20 °C was slowly added prop-1-en-1-ylmagnesium bromide (20 mL, 10 mmol, 0.5 M in THF). The solution was stirred at -20 °C for 1 h, then mixture was allowed to gradually warm to room temperature for 9 h. After the reaction was complete, the mixture was quenched with saturated NH_4Cl (aq.) carefully at 0 °C, extracted with EtOAc (10 mL×3). The combined organic layers were washed with brine (20 mL), dried over Na_2SO_4, filtered and concentrated in vacuo. The crude product was purified by column chromatography (eluent: petroleum ether/ethyl acetate = 30/1) to afford **1a** (923 mg, 57% yield, *Z*:*E* = 3:1 determined by ^1H NMR analysis), colorless oil. ^1H NMR (400 MHz, Chloroform-*d*) δ 7.34-7.30 (m, 2H), 7.26-7.22 (m, 3H), 5.73-5.64 (*E*, m, 0.25H), 5.62-5.54 (m, 1H), 5.50-5.44 (*Z*, m, 0.75H), 4.72-4.67 (*Z*, m, 0.75H), 4.32-4.27 (*E*, m, 0.25H), 2.88-2.75 (m, 2H), 1.72-1.56 (m, 4H); ^{13}C NMR (100 MHz, Chloroform-*d*) δ 138.0, 137.8, 133.2, 132.4, 129.6, 129.5, 128.39, 128.37, 127.0, 126.6, 126.4, 73.5, 68.4, 44.1, 43.9, 17.6, 13.1; HRMS (ESI) m/z calculated for $C_{11}H_{14}O$ $[M+Na]^+$: 185.0937, found: 185.0935.

*Synthesis of enol derivatives (**S1-19**)*

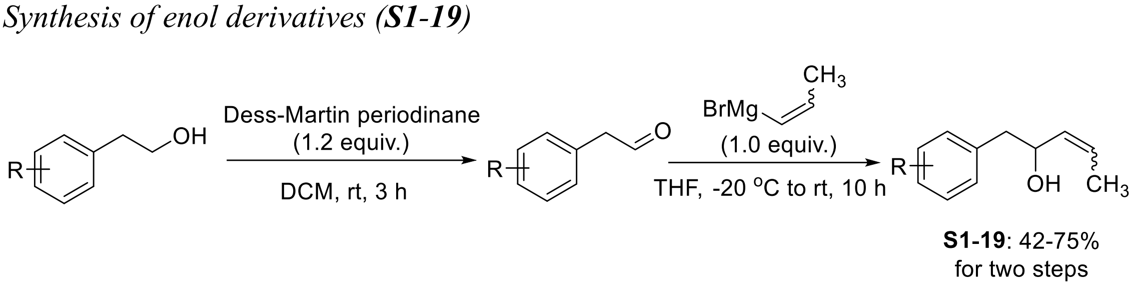

To a solution of alcohol (5.0 mmol) in CH_2Cl_2 (50 mL) was added Dess-Martin periodinane (2.545 g, 6.0 mmol). The reaction mixture was stirred at room temperature

for 3 h. After the alcohol was completely consumed as determined by TLC, the reaction mixture was quenched with saturated $Na_2S_2O_3$ (aq., 20 mL) and $NaHCO_3$ (aq., 20 mL), and extracted with CH_2Cl_2 (10 mL×3). The combined organic layers were dried over Na_2SO_4. After filtration, evaporation of the solvent afforded the crude aldehyde products[12], which were used as the starting material in the next step without further purification.

To a 100 mL three-necked flask containing a solution of corresponding aldehyde (5.0 mmol) in dry THF (30 mL) at -20 °C was slowly added prop-1-en-1-ylmagnesium bromide (10 mL, 5.0 mmol, 0.5 M in THF). The solution was stirred at -20 °C for 1 h, then reaction mixture was allowed to gradually warm to room temperature and stirred for additional for 9 h. After the reaction was complete, the mixture was quenched with saturated NH_4Cl (aq.) carefully at 0 °C, filtered and extracted with EtOAc (10 mL×3). The combined organic layers were washed with brine (20 mL), dried over Na_2SO_4, and concentrated in vacuo. The crude product was purified by column chromatography (eluent: petroleum ether/ethyl acetate = 30/1).

*1-(p-tolyl)pent-3-en-2-ol (**S1**)*

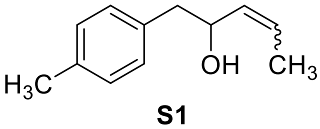

S1

Z:*E* = 3:1

56% isolated yield for 2 steps (*Z*:*E* = 3:1 determined by ^{1}H NMR analysis), slightly yellow oil. ^{1}H NMR (400 MHz, Chloroform-*d*) δ 7.17-7.10 (m, 4H), 5.74-5.41 (m, 2H), 4.69-4.63 (*Z*, m, 0.75H), 4.28-4.23 (*E*, m, 0.25H), 2.86-2.66 (m, 2H), 2.32 (s, 3H), 1.70-1.56 (m, 4H); ^{13}C NMR (100 MHz, Chloroform-*d*) δ 136.0, 134.7, 133.2, 132.5, 129.41, 129.39, 129.2, 129.1, 127.0, 126.5, 73.6, 68.5, 43.7, 43.4, 21.0, 17.7, 13.2; HRMS (ESI) m/z calculated for $C_{12}H_{16}O$ $[M+Na]^+$: 199.1093, found: 199.1091.

*1-(4-(tert-butyl)phenyl)pent-3-en-2-ol (**S2**)*

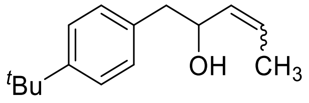

S2
Z:*E* = 3.5:1

50% isolated yield for 2 steps (*Z*:*E* = 3.5:1 determined by ^{1}H NMR analysis), slightly yellow oil. ^{1}H NMR (400 MHz, Chloroform-*d*) δ 7.33 (d, *J* = 8.3 Hz, 2H), 7.17 (d, *J* = 8.2 Hz, 2H), 5.76-5.44 (m, 2H), 4.70-4.65 (*Z*, m, 0.78H), 4.30-4.25 (*E*, m, 0.22H), 2.88-2.66 (m, 2H), 1.72-1.58 (m, 3H), 1.56 (s, 1H), 1.31 (s, 9H); ^{13}C NMR (100 MHz, Chloroform-*d*) δ 149.3, 134.7, 133.3, 132.5, 129.2, 129.2, 126.9, 126.5, 125.4, 73.5, 68.4, 43.6, 43.4, 34.4, 31.4, 17.7, 13.2; HRMS (ESI) m/z calculated for $C_{15}H_{22}O$ $[M+Na]^+$: 241.1563, found: 241.1568.

*1-(4-chlorophenyl)pent-3-en-2-ol (**S3**)*

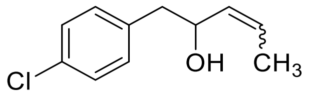

S3
Z:*E* = 3.8:1

42% isolated yield for 2 steps (*Z*:*E* = 3.8:1 determined by ^{1}H NMR analysis), slightly yellow oil. ^{1}H NMR (400 MHz, Chloroform-*d*) δ 7.29-7.24 (m, 2H), 7.19-7.13 (m, 2H), 5.71-5.37 (m, 2H), 4.68-4.63 (*Z*, m, 0.79H), 4.28-4.23 (*E*, m, 0.21H), 2.87-2.69 (m, 2H), 1.70-1.54 (m, 4H); ^{13}C NMR (100 MHz, Chloroform-*d*) δ 136.9, 136.7, 133.3, 132.6, 132.5, 131.23, 131.19, 131.0, 128.8, 127.8, 127.3, 73.8, 68.6, 43.6, 43.4, 18.0, 13.5; HRMS (ESI) m/z calculated for $C_{11}H_{12}{}^{35}Cl$ $[M+H-H_2O]^+$: 179.0622, found: 179.0620.

*1-(4-methoxyphenyl)pent-3-en-2-ol (**S4**)*

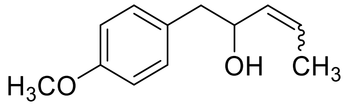

S4
Z:*E* = 3.5:1

52% isolated yield for 2 steps (*Z*:*E* = 3.5:1 determined by ^{1}H NMR analysis), slightly yellow oil. ^{1}H NMR (400 MHz, Chloroform-*d*) δ 7.17-7.12 (m, 2H), 6.85 (d, *J* = 8.6 Hz, 2H), 5.72-5.42 (m, 2H), 4.67-4.61 (*Z*, m, 0.78H), 4.26-4.21 (*E*, m, 0.22H), 3.79 (s, 3H), 2.82-2.65 (m, 2H), 1.70-1.56 (m, 4H); ^{13}C NMR (100 MHz, Chloroform-*d*) δ 158.3, 133.2, 132.5, 130.50, 130.46, 129.8, 127.0, 126.57, 126.55, 113.86, 113.84, 73.6, 68.5, 55.2, 43.2, 43.0, 17.7, 13.2; HRMS (ESI) m/z calculated for $C_{12}H_{16}O_2$ $[M+Na]^+$: 215.1043, found: 215.1048.

*1-(4-fluorophenyl)pent-3-en-2-ol (**S5**)*

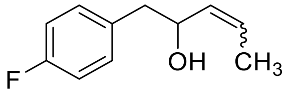

S5

Z:*E* = 4:1

43% isolated yield for 2 steps (*Z*:*E* = 4:1 determined by ^{1}H NMR analysis), slightly yellow oil. ^{1}H NMR (400 MHz, Chloroform-*d*) δ 7.21-7.15 (m, 2H), 7.01-6.93 (m, 2H), 5.73-5.38 (m, 2H), 4.68-4.62 (*Z*, m, 0.8H), 4.27-4.22 (*E*, m, 0.2H), 2.85-2.72 (m, 2H), 1.70-1.53 (m, 4H); ^{13}C NMR (100 MHz, Chloroform-*d*) δ 162.9, 160.5, 133.54, 133.51, 133.0, 132.2, 131.01, 130.96, 130.93, 130.88, 127.4, 126.9, 115.2, 115.0, 73.6, 68.4, 43.1, 42.9, 17.7, 13.2; ^{19}F NMR (376 MHz, Chloroform-*d*) δ -116.9; HRMS (ESI) m/z calculated for $C_{11}H_{12}F$ $[M+H-H_2O]^+$: 163.0918, found: 163.0915.

*(Z)-1-(naphthalen-1-yl)pent-3-en-2-ol (**S6**)*

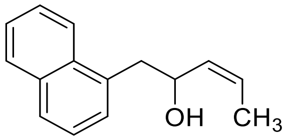

S6

(*Z*)

58% isolated yield for 2 steps, yellow oil. ^{1}H NMR (400 MHz, Chloroform-*d*) δ 8.09 (d, *J* = 8.6 Hz, 1H), 7.86 (dd, *J* = 8.4, 1.3 Hz, 1H), 7.76 (d, *J* = 8.0 Hz, 1H), 7.56-7.46 (m, 2H), 7.44-7.35 (m, 2H), 5.62-5.50 (m, 2H), 4.89-4.84 (m, 1H), 3.27 (d, *J* = 6.6 Hz, 2H), 1.58 (s, 1H), 1.49 (d, *J* = 5.1 Hz, 3H); ^{13}C NMR (100 MHz, Chloroform-*d*) δ

133.9, 132.7, 132.2, 128.8, 127.9, 127.4, 126.8, 126.0, 125.6, 125.4, 123.7, 67.7, 40.9, 13.2; HRMS (EI) m/z calculated for $C_{15}H_{16}O$ $[M]^+$: 212.1196, found: 212.1197.

*1-(m-tolyl)pent-3-en-2-ol (**S7**)*

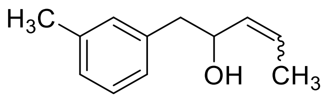

S7
Z:*E* = 4:1

75% isolated yield for 2 steps (*Z*:*E* = 4:1 determined by ^{1}H NMR analysis), slightly yellow oil. ^{1}H NMR (400 MHz, Chloroform-*d*) δ 7.22-7.18 (m, 1H), 7.08-7.02 (m, 3H), 5.77-5.42 (m, 2H), 4.71-4.66 (*Z*, m, 0.8H), 4.31-4.26 (*E*, m, 0.2H), 2.87-2.67 (m, 2H), 2.35 (s, 3H), 1.72-1.59 (m, 4H); ^{13}C NMR (100 MHz, Chloroform-*d*) δ 138.0, 137.7, 133.2, 132.5, 130.3, 128.3, 127.2, 127.0, 126.53, 126.51, 73.5, 68.4, 44.1, 43.8, 21.4, 17.7, 13.2; HRMS (ESI) m/z calculated for $C_{12}H_{16}O$ $[M+Na]^+$: 199.1093, found: 199.1090.

*(Z)-1-(3-fluorophenyl)pent-3-en-2-ol (**S8**)*

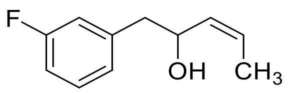

S8
(*Z*)

61% isolated yield for 2 steps, slightly yellow oil. ^{1}H NMR (400 MHz, Chloroform-*d*) δ 7.31-7.25 (m, 1H), 7.03 (d, *J* = 7.6 Hz, 1H), 7.00-6.91 (m, 2H), 5.64-5.56 (m, 1H), 5.50-5.43 (m, 1H), 4.74-4.68 (m, 1H), 2.88 (dd, *J* = 13.5, 7.3 Hz, 1H), 2.79 (dd, *J* = 13.5, 5.8 Hz, 1H), 1.61 (s, 1H), 1.59 (dd, *J* = 6.9, 1.7 Hz, 3H); ^{13}C NMR (100 MHz, Chloroform-*d*) δ 162.8 (d, *J* = 244.4 Hz), 140.5 (d, *J* = 7.3 Hz), 132.2, 129.7 (d, *J* = 8.3 Hz), 127.0, 125.2 (d, *J* = 2.9 Hz), 116.4 (d, *J* = 20.9 Hz), 113.3 (d, *J* = 21.0 Hz), 68.2, 43.5, 13.2; ^{19}F NMR (376 MHz, Chloroform-*d*) δ -113.6; HRMS (ESI) m/z calculated for $C_{11}H_{13}FO$ $[M+Na]^+$: 203.0843, found: 203.0838.

*(Z)-1-(3-methoxyphenyl)pent-3-en-2-ol (**S9**)*

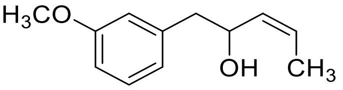

S9
(*Z*)

67% isolated yield for 2 steps, slightly yellow oil. ^{1}H NMR (400 MHz, Chloroform-*d*) δ 7.24-7.20 (m, 1H), 6.83 (d, *J* = 7.6 Hz, 1H), 6.79-6.77 (m, 2H), 5.62-5.53 (m, 1H), 5.50-5.43 (m, 1H), 4.72-4.66 (m, 1H), 3.80 (s, 3H), 2.84-2.74 (m, 2H), 1.64 (s, 1H), 1.59 (dd, *J* = 6.8, 1.7 Hz, 3H); ^{13}C NMR (100 MHz, Chloroform-*d*) δ 159.7, 139.5, 132.4, 129.4, 126.6, 121.9, 115.3, 111.8, 68.3, 55.1, 43.9, 13.2; HRMS (ESI) m/z calculated for $C_{12}H_{16}O_2$ $[M+Na]^+$: 215.1043, found: 215.1033.

*1-(naphthalen-2-yl)pent-3-en-2-ol (**S10**)*

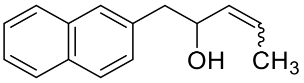

S10
Z:*E* = 16:1

50% isolated yield for 2 steps (*Z*:*E* = 16:1 determined by ^{1}H NMR analysis), yellow oil. ^{1}H NMR (400 MHz, Chloroform-*d*) δ 7.84-7.79 (m, 3H), 7.69 (s, 1H), 7.50-7.43 (m, 2H), 7.39 (dd, *J* = 8.4, 1.8 Hz, 1H), 5.74-5.49 (m, 2H), 4.83-4.78 (*Z*, m, 0.94H), 4.42-4.37 (*E*, m, 0.06H), 3.04-2.94 (m, 2H), 1.72-1.58 (m, 4H); ^{13}C NMR (100 MHz, Chloroform-*d*) δ 135.4, 133.5, 132.4, 132.2, 128.03, 127.98, 127.92, 127.6, 127.5, 126.7, 126.0, 125.4, 73.5, 68.3, 44.2, 44.0, 17.7, 13.2; HRMS (ESI) m/z calculated for $C_{15}H_{16}O$ $[M+Na]^+$: 235.1093, found: 235.1082.

*(Z)-1-([1,1'-biphenyl]-4-yl)pent-3-en-2-ol (**S11**)*

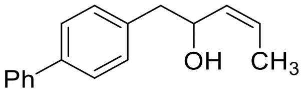

S11
(*Z*)

43% isolated yield for 2 steps, yellow oil. ^{1}H NMR (400 MHz, Chloroform-*d*) δ 7.61-7.54 (m, 4H), 7.46-7.42 (m, 2H), 7.36-7.31 (m, 3H), 5.64-5.48 (m, 2H), 4.77-4.72 (m, 1H), 2.92-2.82 (m, 2H), 1.62 (s, 1H), 1.61 (dd, *J* = 6.8, 1.6 Hz, 3H); ^{13}C NMR (100

MHz, Chloroform-*d*) δ 140.9, 139.4, 137.0, 132.5, 130.0, 128.7, 127.1, 127.0, 126.7, 68.4, 43.5, 13.2; HRMS (ESI) m/z calculated for $C_{17}H_{18}O$ $[M+Na]^+$: 261.1250, found: 261.1253.

(Z)-1-(4-(trifluoromethyl)phenyl)pent-3-en-2-ol ***(S12)***

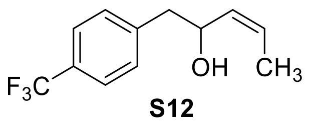

S12
(*Z*)

43% isolated yield for 2 steps, slightly yellow oil. ^{1}H NMR (400 MHz, Chloroform-*d*) δ 7.55 (d, *J* = 8.0 Hz, 2H), 7.35 (d, *J* = 7.9 Hz, 2H), 5.62-5.54 (m, 1H), 5.47-5.42 (m, 1H), 4.73-4.68 (m, 1H), 2.92 (dd, *J* = 13.5, 7.2 Hz, 1H), 2.83 (dd, *J* = 13.5, 5.8 Hz, 1H), 1.59 (s, 1H), 1.55 (dd, *J* = 6.9, 1.7 Hz, 3H); ^{13}C NMR (100 MHz, Chloroform-*d*) δ 142.1, 132.0, 129.9, 128.7 (q, *J* = 32.1 Hz), 127.2, 125.2 (q, *J* = 3.8 Hz), 124.3 (q, *J* = 270.4 Hz), 68.1, 43.5, 13.2; ^{19}F NMR (376 MHz, Chloroform-*d*) δ -62.4; HRMS (ESI) m/z calculated for $C_{12}H_{12}F_3$ $[M+H-H_2O]^+$: 213.0886, found: 213.0891.

(Z)-1-(o-tolyl)pent-3-en-2-ol ***(S13)***

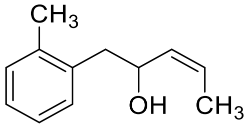

S13
(*Z*)

62% isolated yield for 2 steps, slightly yellow oil. ^{1}H NMR (400 MHz, Chloroform-*d*) δ 7.20-7.13 (m, 4H), 5.61-5.48 (m, 2H), 4.73-4.68 (m, 1H), 2.87 (dd, *J* = 13.6, 7.7 Hz, 1H), 2.81 (dd, *J* = 13.7, 5.7 Hz, 1H), 2.37 (s, 3H), 1.65 (s, 1H), 1.54 (d, *J* = 6.6 Hz, 3H); ^{13}C NMR (100 MHz, Chloroform-*d*) δ 136.6, 136.1, 132.6, 130.4, 130.3, 126.6, 126.4, 125.9, 67.5, 41.0, 19.7, 13.1; HRMS (ESI) m/z calculated for $C_{12}H_{16}O$ $[M+Na]^+$: 199.1093, found: 199.1094.

1-(2-fluorophenyl)pent-3-en-2-ol ***(S14)***

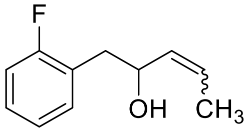

S14
Z:*E* = 7.3:1

68% isolated yield for 2 steps (*Z*:*E* = 7.3:1 determined by ^{1}H NMR analysis), slightly yellow oil. ^{1}H NMR (400 MHz, Chloroform-*d*) δ 7.25-7.18 (m, 2H), 7.09-7.00 (m, 2H), 5.69-5.44 (m, 2H), 4.78-4.72 (*Z*, m, 0.88H), 4.35-4.30 (*E*, m, 0.12H), 2.93-2.80 (m, 2H), 1.69-1.53 (m, 4H); ^{13}C NMR (100 MHz, Chloroform-*d*) δ 162.6, 160.2, 133.1, 132.3, 132.00, 131.95, 131.91, 131.87, 128.24, 128.20, 128.16, 128.12, 127.3, 126.9, 125.0, 124.9, 123.90, 123.87, 115.4, 115.3, 115.14, 115.10, 72.6, 67.4, 67.4, 37.2, 37.1, 17.6, 13.1; ^{19}F NMR (376 MHz, Chloroform-*d*) δ -117.8; HRMS (ESI) m/z calculated for $C_{11}H_{13}FO$ $[M+Na]^{+}$: 203.0843, found: 203.0839.

1-(2-methoxyphenyl)pent-3-en-2-ol ***(S15)***

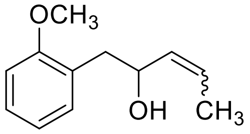

S15
Z:*E* = 19:1

65% isolated yield for 2 steps (*Z*:*E* = 19:1 determined by ^{1}H NMR analysis), slightly yellow oil. ^{1}H NMR (400 MHz, Chloroform-*d*) δ 7.25-7.20 (m, 1H), 7.15 (dd, *J* = 7.3, 1.8 Hz, 1H), 6.93-6.86 (m, 2H), 5.58-5.45 (m, 2H), 4.79-4.74 (*Z*, m, 0.95H), 4.34-4.30 (*E*, m, 0.05H), 3.85 (s, 3H), 2.89-2.80 (m, 2H), 2.03 (s, 1H), 1.70-1.58 (m, 3H); ^{13}C NMR (100 MHz, Chloroform-*d*) δ 157.6, 133.7, 133.1, 131.5, 127.8, 126.5, 126.3, 125.8, 120.6, 110.3, 72.6, 67.5, 55.3, 38.9, 17.6, 13.1; HRMS (ESI) m/z calculated for $C_{12}H_{16}O_2$ $[M+Na]^{+}$: 215.1043, found: 215.1053.

*1-(thiophen-2-yl)pent-3-en-2-ol (**S16**)*

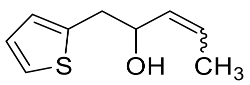

S16
Z:*E* = 13:1

66% isolated yield for 2 steps (*Z*:*E* = 13:1 determined by ^{1}H NMR analysis), yellow oil. ^{1}H NMR (400 MHz, Chloroform-*d*) δ 7.18 (dd, *J* = 5.1, 1.2 Hz, 1H), 6.96 (dd, *J* = 5.2, 3.4 Hz, 1H), 6.88 (d, *J* = 3.4 Hz, 1H), 5.76-5.44 (m, 2H), 4.71-4.66 (*Z*, m, 0.93H), 4.31-4.26 (*E*, m, 0.07H), 3.08-2.98 (m, 2H), 1.78 (s, 1H), 1.72-1.63 (m, 3H); ^{13}C NMR (100 MHz, Chloroform-*d*) δ 139.9, 132.6, 131.9, 127.7, 127.4, 126.9, 126.2, 124.3, 73.2, 68.1, 37.9, 17.7, 13.3; HRMS (ESI) m/z calculated for $C_9H_{12}OS$ $[M+Na]^+$: 191.0501, found: 191.0509.

*1-(thiophen-3-yl)pent-3-en-2-ol (**S17**)*

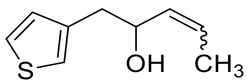

S17
Z:*E* = 16:1

65% isolated yield for 2 steps (*Z*:*E* = 16:1 determined by ^{1}H NMR analysis), yellow oil. ^{1}H NMR (400 MHz, Chloroform-*d*) δ 7.27 (dt, *J* = 5.3, 2.7 Hz, 1H), 7.06 (d, *J* = 1.7 Hz, 1H), 7.00 (dd, *J* = 4.9, 1.3 Hz, 1H), 5.73-5.45 (m, 2H), 4.71-4.65 (*Z*, m, 0.94H), 4.31-4.26 (*E*, m, 0.06H), 2.91-2.80 (m, 2H), 1.71-1.60 (m, 4H); ^{13}C NMR (100 MHz, Chloroform-*d*) δ 138.1, 132.4, 128.8, 126.8, 125.5, 122.2, 72.8, 67.7, 38.2, 17.7, 13.2; HRMS (ESI) m/z calculated for $C_9H_{12}OS$ $[M+Na]^+$: 191.0501, found: 191.0495.

*(Z)-1-(3-(trifluoromethyl)phenyl)pent-3-en-2-ol (**S18**)*

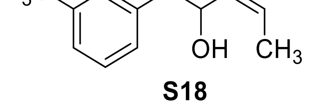

S18
(*Z*)

67% isolated yield for 2 steps, slightly yellow oil. ^{1}H NMR (400 MHz, Chloroform-*d*) δ 7.49-7.46 (m, 2H), 7.44-7.38 (m, 2H), 5.62-5.54 (m, 1H), 5.47-5.41 (m, 1H), 4.73-

4.68 (m, 1H), 2.93 (dd, *J* = 13.5, 7.2 Hz, 1H), 2.82 (dd, *J* = 13.5, 5.9 Hz, 1H), 1.62 (s, 1H), 1.53 (dd, *J* = 6.9, 1.7 Hz, 3H); ^{13}C NMR (100 MHz, Chloroform-*d*) δ 138.9, 133.0, 132.0, 130.6 (q, *J* = 31.7 Hz), 128.7, 127.2, 126.3 (q, *J* = 3.8 Hz), 124.2 (q, *J* = 270.3 Hz), 123.2 (q, *J* = 3.9 Hz), 68.1, 43.5, 13.1; ^{19}F NMR (376 MHz, Chloroform-*d*) δ -62.6; HRMS (ESI) m/z calculated for $C_{12}H_{12}F_3$ $[M+H-H_2O]^+$: 213.0886, found: 213.0883.

1-(4-(trifluoromethoxy)phenyl)pent-3-en-2-ol ***(S19)***

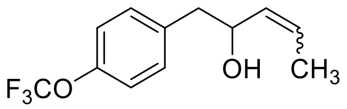

S19
Z:*E* = 3:1

70% isolated yield for 2 steps (*Z*:*E* = 3:1 determined by ^{1}H NMR analysis), slightly yellow oil. ^{1}H NMR (400 MHz, Chloroform-*d*) δ 7.26-7.23 (m, 2H), 7.15 (d, *J* = 7.9 Hz, 2H), 5.72-5.41 (m, 2H), 4.71-4.65 (*Z*, m, 0.75H), 4.31-4.25 (*E*, m, 0.25H), 2.89-2.75 (m, 2H), 1.71-1.53 (m, 4H); ^{13}C NMR (100 MHz, Chloroform-*d*) δ 147.8, 136.9, 136.7, 133.0, 132.1, 130.9, 130.8, 127.5, 127.0, 120.8, 120.5 (q, *J* = 255.2 Hz), 73.5, 68.3, 43.2, 43.0, 17.6, 13.1; ^{19}F NMR (376 MHz, Chloroform-*d*) δ -57.9; HRMS (ESI) m/z calculated for $C_{12}H_{13}F_3O_2$ $[M+Na]^+$: 269.0760, found: 269.0763.

*Synthesis of 1-(1-methyl-1H-indol-3-yl)pent-3-en-2-ol (**S20**)*

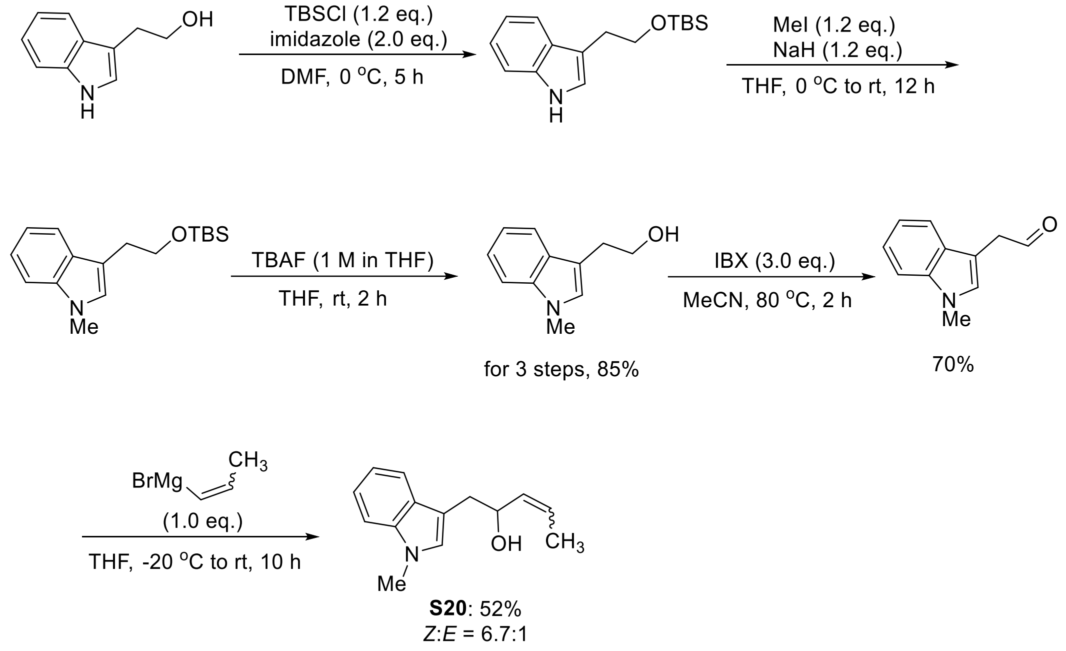

tert-Butyldimethylsilylchloride (1.809 g, 12.0 mmol) was added to a solution of tryptophol (1.612 g, 10.0 mmol) and imidazole (1.362 g, 20.0 mmol) in DMF (50 mL) at 0 °C. The ice bath was then removed and the reaction was stirred at room temperature for 5 h. The mixture was quenched with water (40 mL) and extracted with EtOAc (10 mL×3). The combined organic layers were washed with brine, and dried over Na_2SO_4. The mixture was then filtered and concentrated under reduced pressure. The residue was used directly for the next step without further purification.[13]

To a solution of the above TBS-protected tryptophol in THF (50 mL) was added NaH (400 mg, 10.0 mmol, 60% dispersion in mineral oil) at 0 °C. After being stirred at 0 °C for 15 min and then at rt for 1 h, the reaction was cooled to 0 °C, treated with MeI (700 μL, 11 mmol), and then allowed to stir at rt for additional 12 h. After the reaction was complete as monitored by TLC, saturated $NaHCO_3$ (aq., 30 mL) was added slowly. The organic layer was separated and the aqueous layer was extracted with EtOAc (10 mL×3). The combined organic layers were washed with brine (30 mL), and dried over Na_2SO_4. The mixture was then concentrated under reduced pressure. The crude product was further treated with *tetra-n*-butylammonium fluoride (15 mL, 15 mmol, 1.0 M in THF) at room temperature, and stirred for 2 h. The mixture was purified by column chromatography on silica gel (petroleum ether/ethyl acetate = 5/1) to afford the desired

product *N*-methyltryptophol (1.499 g, 85% yield for 3 steps).

To a 100 mL single-neck flask were added *N*-methyltryptophol (1.499 g, 8.5 mmol), acetonitrile (40 mL), and 2-iodoxybenzoic acid (IBX, 7.141 g, 25.5 mmol) sequentially. The mixture was heated at 80 °C for 2 h, and then cooled to room temperature. After filtration through Celite, the liquid phase was concentrated under reduced pressure.[14] The expected *N*-methyl-3-indoleacetaldehyde was obtained in 1.031g, 70% yield.

To a 100 mL three-necked flask containing a solution of *N*-methyl-3-indoleacetaldehyde (866 mg, 5.0 mmol) in dry THF (30 mL) at -20 °C was slowly added prop-1-en-1-ylmagnesium bromide (10 mL, 5.0 mmol, 0.5 M in THF). The reaction was stirred at -20 °C for 1 h, then allowed to gradually warm to room temperature and stirred for additional 9 h. After completion, the reaction was quenched with saturated NH_4Cl (aq.) carefully at 0 °C, and extracted with EtOAc (10 mL×3). The combined organic layers were washed with brine (20 mL), dried over Na_2SO_4, and concentrated in vacuo. The crude product was purified by column chromatography (eluent: petroleum ether/ethyl acetate = 15/1) to afford **S20** (559 mg, 52% yield, *Z*:*E* = 6.7:1 determined by ^{1}H NMR analysis), brown oil. ^{1}H NMR (400 MHz, Chloroform-*d*) δ 7.65 (d, *J* = 7.9 Hz, 1H), 7.33 (d, *J* = 8.2 Hz, 1H), 7.26 (t, *J* = 7.5 Hz, 1H), 7.15 (t, *J* = 7.3 Hz, 1H), 6.98 (s, 1H), 5.81-5.53 (m, 2H), 4.81-4.76 (*Z*, m, 0.87H), 4.40-4.36 (*E*, m, 0.13H), 3.78 (s, 3H), 3.02 (dd, *J* = 14.3, 4.5 Hz, 1H), 2.92 (dd, *J* = 14.5, 8.3 Hz, 1H), 1.82 (s, 1H), 1.75-1.71 (m, 3H); ^{13}C NMR (100 MHz, Chloroform-*d*) δ 137.1, 133.6, 133.0, 128.1, 127.8, 126.8, 126.4, 121.7, 119.1, 119.0, 118.90, 118.86, 110.1, 109.2, 72.3, 67.2, 33.7, 33.4, 32.6, 17.7, 13.3; HRMS (EI) m/z calculated for $C_{14}H_{17}NO$ $[M]^+$: 215.1305, found: 215.1304.

*Synthesis of 1-phenylbut-3-en-2-ol (**S21**)*[15]

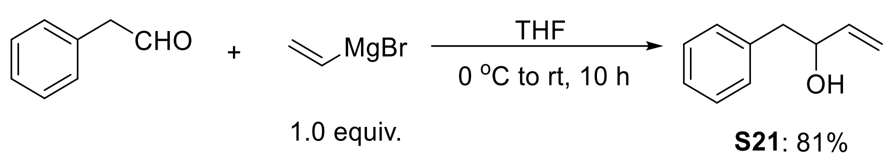

To a solution of vinylmagnesium bromide (12 mL, 12 mmol, 1.0 M in THF) in THF

(20 mL) was added 2-phenylacetaldehyde (1.201 g, 10 mmol) at 0 °C. After the mixture was stirred at 0 °C for 1 h and then at room temperature for additional 9 h, HCl (aq., 0.5 M, 10 mL) was added to the mixture and organic phase was separated. The aqueous phase was extracted with EtOAc (10 mL×3). The combined organic phase was dried over Na_2SO_4, and concentrated in vacuo. The crude product was purified by column chromatography (eluent: petroleum ether/ethyl acetate = 30/1) to afford **S21** (1.198 g, 81% yield), colorless oil. ^{1}H NMR (400 MHz, Chloroform-*d*) δ 7.35-7.31 (m, 2H), 7.27-7.23 (m, 3H), 5.98-5.90 (m, 1H), 5.26 (dt, *J* = 17.2, 1.5 Hz, 1H), 5.14 (dt, *J* = 10.5, 1.4 Hz, 1H), 4.38-4.33 (m, 1H), 2.89 (dd, *J* = 13.6, 5.2 Hz, 1H), 2.80 (dd, *J* = 13.6, 7.9 Hz, 1H), 1.77 (s, 1H); ^{13}C NMR (100 MHz, Chloroform-*d*) δ 140.1, 137.7, 129.5, 128.4, 126.5, 114.9, 73.6, 43.8; HRMS (ESI) m/z calculated for $C_{10}H_{11}$ $[M+H-H_2O]^+$: 131.0855, found: 131.0850.

*Synthesis of 3-en-2-ol (**S22-25**)*

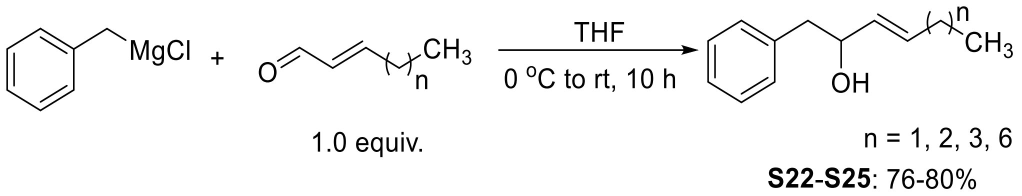

To a solution of aldehyde (5 mmol) in dry THF (20 mL) under N_2 at 0 °C was added the benzylmagnesium chloride (5 mL, 5 mmol, 1.0 M in THF). Then reaction mixture was allowed to gradually warm to room temperature, and stirred for additional 10 h. After completion, the reaction was quenched by with saturated NH_4Cl (aq.) and diluted with EtOAc (10 mL). The organic layer was separated and the aqueous layer was extracted with EtOAc (10 mL×3). The combined organic layers were washed with brine (10 mL), dried over Na_2SO_4, and concentrated in vacuo. The crude product was purified by column chromatography (eluent: petroleum ether/ethyl acetate = 40/1) gave the desired products.

*(E)-1-phenylhex-3-en-2-ol (**S22**)*

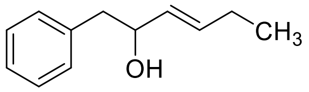

S22

76% isolated yield, colorless oil. ^{1}H NMR (400 MHz, Chloroform-*d*) δ 7.30 (t, *J* = 7.6 Hz, 2H), 7.25-7.21 (m, 3H), 5.69 (dt, *J* = 15.4, 6.2 Hz, 1H), 5.52 (dd, *J* = 15.1, 6.4 Hz, 1H), 4.31-4.26 (m, 1H), 2.85 (dd, *J* = 13.6, 5.1 Hz, 1H), 2.77 (dd, *J* = 13.5, 7.9 Hz, 1H), 2.04 (p, *J* = 7.2 Hz, 2H), 1.62 (s, 1H), 0.98 (t, *J* = 7.5 Hz, 3H); ^{13}C NMR (100 MHz, Chloroform-*d*) δ 138.0, 133.8, 130.9, 129.5, 128.4, 126.4, 73.6, 44.2, 25.2, 13.4; HRMS (ESI) m/z calculated for $C_{12}H_{16}O$ $[M+Na]^+$: 199.1093, found: 199.1089.

*(E)-1-phenylhept-3-en-2-ol (**S23**)*

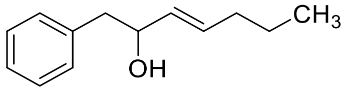

S23

78% isolated yield, colorless oil. ^{1}H NMR (400 MHz, Chloroform-*d*) δ 7.33-7.29 (m, 2H), 7.24-7.21 (m, 3H), 5.65 (dt, *J* = 14.9, 6.3 Hz, 1H), 5.56-5.50 (m, 1H), 4.33-4.28 (m, 1H), 2.88-2.76 (m, 2H), 2.01 (q, *J* = 7.1 Hz, 2H), 1.58 (s, 1H), 1.43-1.34 (m, 2H), 0.88 (t, *J* = 7.4 Hz, 3H); ^{13}C NMR (100 MHz, Chloroform-*d*) δ 138.0, 132.2, 132.1, 129.6, 128.4, 126.4, 73.6, 44.2, 34.3, 22.2, 13.6; HRMS (ESI) m/z calculated for $C_{13}H_{18}O$ $[M+Na]^+$: 213.1250, found: 213.1250.

*(E)-1-phenyloct-3-en-2-ol (**S24**)*[16]

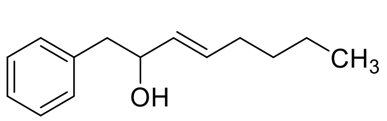

S24

77% isolated yield, colorless oil. ^{1}H NMR (400 MHz, Chloroform-*d*) δ 7.33-7.29 (m, 2H), 7.25-7.21 (m, 3H), 5.64 (dt, *J* = 15.1, 6.2 Hz, 1H), 5.56-5.50 (m, 1H), 4.32-4.28 (m, 1H), 2.88-2.76 (m, 2H), 2.03 (q, *J* = 6.9 Hz, 2H), 1.62 (s, 1H), 1.39-1.24 (m, 4H), 0.89 (t, *J* = 7.0 Hz, 3H); ^{13}C NMR (100 MHz, Chloroform-*d*) δ 138.0, 132.4, 131.9,

129.6, 128.4, 126.4, 73.6, 44.2, 31.8, 31.2, 22.1, 13.9; HRMS (ESI) m/z calculated for $C_{14}H_{19}$ $[M+H-H_2O]^+$: 187.1481, found: 187.1480.

*(E)-1-phenylundec-3-en-2-ol (**S25**)*

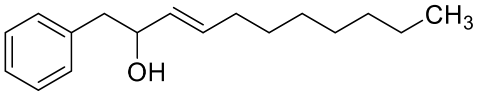

S25

80% isolated yield, colorless oil. ^{1}H NMR (400 MHz, Chloroform-*d*) δ 7.32-7.29 (m, 2H), 7.25-7.21 (m, 3H), 5.64 (dt, *J* = 15.0, 6.1 Hz, 1H), 5.55-5.49 (m, 1H), 4.33-4.27 (m, 1H), 2.87-2.76 (m, 2H), 2.02 (q, *J* = 7.1 Hz, 2H), 1.54 (s, 1H), 1.37-1.26 (m, 10H), 0.89 (t, *J* = 6.8 Hz, 3H); ^{13}C NMR (100 MHz, Chloroform-*d*) δ 138.0, 132.5, 131.8, 129.6, 128.4, 126.4, 73.6, 44.2, 32.2, 31.8, 29.1, 29.1, 29.1, 22.7, 14.1; HRMS (ESI) m/z calculated for $C_{17}H_{26}O$ $[M+Na]^+$: 269.1876, found: 269.1870.

Synthesis of 2-(prop-1-en-1-yl)-2,3-dihydro-1H-inden-2-ol

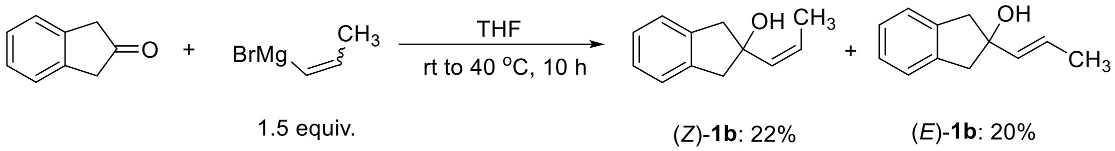

To a solution of 1,3-dihydro-2*H*-inden-2-one (660 mg, 5 mmol) in dry THF (20 mL) under N_2 at room temperature was added prop-1-en-1-ylmagnesium bromide (15 mL, 7.5 mmol, 0.5 M in THF). The reaction mixture was stirred at 40 °C for 10 h. After completion, the reaction was slowly quenched with saturated NH_4Cl (aq.), and extracted with EtOAc (10 mL×3). The organic layer was washed with brine, dried over Na_2SO_4, filtered, and concentrated in vacuo. The residue was purified by silica gel chromatography (eluent: petroleum ether/ethyl acetate = 40/1) to afford the desired products.

*(Z)-2-(prop-1-en-1-yl)-2,3-dihydro-1H-inden-2-ol ((Z)-**1b**)*

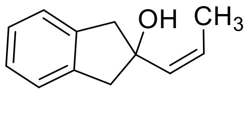

(*Z*)-**1b**

22% isolated yield, brown oil. ^{1}H NMR (400 MHz, Chloroform-*d*) δ 7.26-7.22 (m, 2H), 7.21-7.17 (m, 2H), 5.79 (dq, *J* = 11.5, 1.8 Hz, 1H), 5.63 (dq, *J* = 11.5, 7.2 Hz, 1H), 3.26 (d, *J* = 16.2 Hz, 2H), 3.19 (d, *J* = 16.2 Hz, 2H), 2.05-1.75 (m, 4H); ^{13}C NMR (100 MHz, Chloroform-*d*) δ 141.0, 135.0, 127.7, 126.6, 124.9, 81.7, 48.8, 14.5; HRMS (EI) m/z calculated for $C_{12}H_{14}O$ $[M]^{+}$: 174.1039, found: 174.1038.

*(E)-2-(prop-1-en-1-yl)-2,3-dihydro-1H-inden-2-ol ((E)-**1b**)*

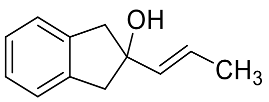

(*E*)-**1b**

20% isolated yield, brown oil. ^{1}H NMR (400 MHz, Chloroform-*d*) δ 7.24-7.20 (m, 2H), 7.19-7.16 (m, 2H), 5.89-5.79 (m, 2H), 3.17 (d, *J* = 16.2 Hz, 2H), 2.97 (d, *J* = 16.1 Hz, 2H), 1.83 (s, 1H), 1.75 (d, *J* = 4.8 Hz, 3H); ^{13}C NMR (100 MHz, Chloroform-*d*) δ 141.16, 135.67, 126.61, 124.88, 123.74, 81.85, 47.51, 17.69; HRMS (EI) m/z calculated for $C_{12}H_{14}O$ $[M]^{+}$: 174.1039, found: 174.1040.

*Synthesis of (Z)-2-(prop-1-en-1-yl)-1,2,3,4-tetrahydronaphthalen-2-ol (**S26**)*

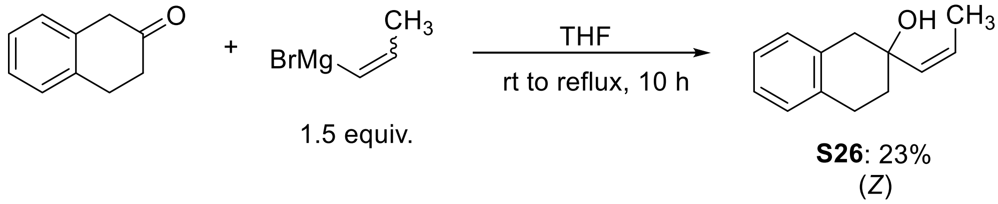

To a solution of the 3,4-dihydronaphthalen-2(1*H*)-one (730 mg, 5 mmol) in dry THF (20 mL) under N_2 at room temperature was added the prop-1-en-1-ylmagnesium bromide (15 mL, 7.5 mmol, 0.5 M in THF). Then reaction mixture was refluxed for 10 h. After completion, the reaction was quenched with saturated NH_4Cl (aq.) carefully at

0 °C, filtered and extracted with EtOAc (10 mL×3). The combined organic layers were washed with brine (10 mL), dried over Na_2SO_4, and concentrated in vacuo. The crude product was purified by column chromatography (eluent: petroleum ether/ethyl acetate = 50/1) to afford **S26** (216 mg, 23% yield), brown oil. ^{1}H NMR (400 MHz, Chloroform-*d*) δ 7.15-7.10 (m, 3H), 7.09-7.06 (m, 1H), 5.59 (dq, J = 12.0 Hz, 6.8 Hz, 1H), 5.55-5.50 (m, 1H), 3.08-2.94 (m, 3H), 2.88-2.80 (m, 1H), 2.07-1.98 (m, 2H), 1.94 (d, J = 5.6 Hz, 3H), 1.74 (s, 1H); ^{13}C NMR (100 MHz, Chloroform-*d*) δ 135.5, 135.4, 134.2, 129.6, 128.6, 127.4, 126.0, 125.8, 71.7, 43.5, 35.0, 26.3, 14.5; HRMS (EI) m/z calculated for $C_{13}H_{16}O$ $[M]^+$: 188.1196, found: 188.1194.

*Synthesis of (Z)-6-(prop-1-en-1-yl)-6,7,8,9-tetrahydro-5H-benzo[7]annulen-6-ol (**S27**)*

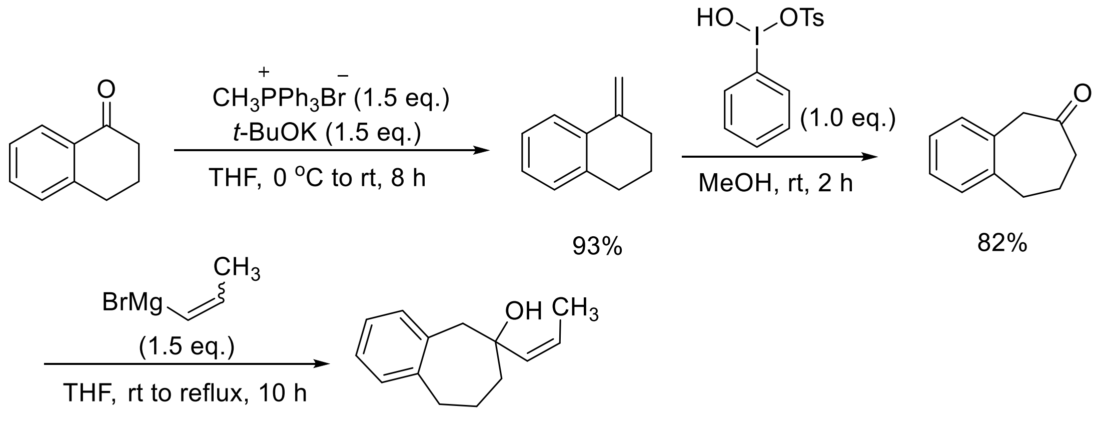

S27: 31%
(*Z*)

Methyltriphenylphosphonium bromide (5.360 g, 15 mmol) was dissolved in THF (30 mL) under an argon atmosphere. After the solution was cooled to 0 °C, potassium tert-butoxide (1.683 g, 15 mmol) was added, and the mixture was stirred for 10 min. Then 3,4-dihydronaphthalen-1(2*H*)-one (1.462 g, 10 mmol) was added, and the reaction was allowed to warm to room temperature and stir for additional 8 h. The reaction was quenched with saturated NH_4Cl (aq.), and extracted with EtOAc (10 mL×3). The combined organic layers were washed with brine (10 mL), dried over Na_2SO_4, and concentrated in vacuo. The crude product was purified by column chromatography (eluent: petroleum ether) to afford 1-methylene-1,2,3,4-tetrahydronaphthalene (1.341 g, 93% yield) as a colorless oil.

[Hydroxy(tosyloxy)iodo]benzene (2.941 g, 7.5 mmol) was added to a solution of 1-methylene-1,2,3,4-tetrahydronaphthalene (1.081 g, 7.5 mmol) in methanol (30 mL), and. The solid dissolved rapidly and exothermically, along with the solution becoming yellow. The mixture was stirred at room temperature for 2 h, then the solvent was removed in vacuo. CH_2Cl_2 and water were added, and the mixture wase extracted with CH_2Cl_2 (10 mL×3). The combined organic layers were washed with brine (10 mL), dried over Na_2SO_4, filtered and concentrated in vacuo.[17] The residue was purified by column chromatography (eluent: petroleum ether /ethyl acetate = 40:1) to afford 5,7,8,9-tetrahydro-6*H*-benzo[7]annulen-6-one (985 mg, 82% yield) as a yellow oil.

To a solution of the 5,7,8,9-tetrahydro-6*H*-benzo[7]annulen-6-one (800 mg, 5 mmol) in dry THF (20 mL) under N_2 at room temperature was added the prop-1-en-1-ylmagnesium bromide (15 mL, 7.5 mmol, 0.5 M in THF). Then reaction was refluxed for 10 h. The mixture was quenched with saturated NH_4Cl (aq.) carefully at 0 °C, filtered and extracted with EtOAc (10 mL×3). The combined organic layers were washed with brine (10 mL), dried over Na_2SO_4, filtered and concentrated in vacuo. The crude product was purified by column chromatography (eluent: petroleum ether/ethyl acetate = 50/1) to afford **S27** (313 mg, 31% yield), yellow oil. ^{1}H NMR (400 MHz, Chloroform-*d*) δ 7.18-7.09 (m, 4H), 5.48 (dq, *J* = 11.7 Hz, 6.7 Hz, 1H), 5.44-5.39 (m, 1H), 3.18 (d, *J* = 13.8 Hz, 1H), 3.06 (d, *J* = 13.8 Hz, 1H), 2.81-2.78 (m, 2H), 2.04-2.00 (m, 2H), 1.92 (d, *J* = 5.8 Hz, 3H), 1.73-1.66 (m, 2H), 1.54 (s, 1H); ^{13}C NMR (100 MHz, Chloroform-*d*) δ 143.2, 136.2, 136.0, 131.6, 128.9, 127.0, 126.3, 125.9, 72.4, 48.7, 44.9, 35.8, 23.5, 14.5; HRMS (EI) m/z calculated for $C_{14}H_{18}O$ $[M]^+$: 202.1352, found: 202.1354.

*Synthesis of 1-phenylpent-3-en-1,1-d₂-2-ol (d₂-**1a**)*

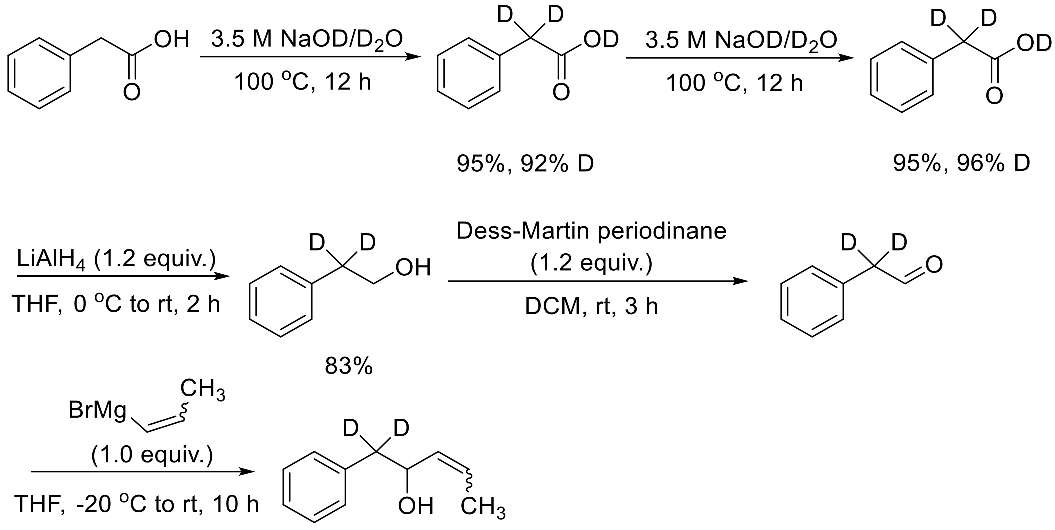

d_2-**1a**: 55%
Z:*E* = 4.6:1

To a reaction tube were added phenylacetic acid (2.723 g, 20 mmol) and NaOD (12.5 mL, 3.5 M in D_2O). The reaction was sealed and stirred at 100 °C for 12 h. After being cooled to room temperature, the reaction was acidified with 4 M aq. HCl solution. The mixture was extracted with CH_2Cl_2 (10 mL×3) and dried with Na_2SO_4. The solution was concentrated by evaporation to give 2.625 g, 95% yield of d_2-phenylacetic acid-*d* with 92% deuterium incorporation. This procedure was repeated and gave 2.493 g, 95% yield of 2-phenylacetic-d_2 acid-*d* with 96% deuterium incorporation.[18]

To an ice-cooled suspension of $LiAlH_4$ (820 mg, 21.6 mmol) in 25 mL of dry THF was dropwise added 2-phenylacetic-d_2 acid-*d* (2.487 g, 18 mmol) in 25 mL of dry THF. The hydride mixture was stirred at room temperature for 2 h, HCl (aq., 0.5 M) was added to the mixture and organic phase was separated. The aqueous phase was extracted with EtOAc (10 mL×3). The combined organic phase was dried over Na_2SO_4, and concentrated in vacuo. The crude product was purified by column chromatography (eluent: petroleum ether/ethyl acetate = 5/1) to afford d_2-2-phenylethanol (1.855 g, 83% yield), colorless oil.

To a solution of d_2-2-phenylethanol (1.861 g, 15 mmol) in CH_2Cl_2 (50 mL) was added Dess-Martin periodinane (7.634 g, 18 mmol). The reaction was stirred at room temperature for 3 h. After the alcohol was completely consumed as determined by TLC,

the mixture was quenched with saturated $Na_2S_2O_3$ (aq., 20 mL) and $NaHCO_3$ (aq., 20 mL), and extracted with CH_2Cl_2 (10 mL×3). The combined organic layers were dried over Na_2SO_4. After filtration, evaporation of the solvent afforded the crude aldehyde product which were used as the starting material in the next step without further purification.

To a 100 mL three-necked flask containing a solution of *d*$_2$-2-phenylacetaldehyde (916 mg, 7.5 mmol) in dry THF (30 mL) at -20 °C was slowly added prop-1-en-1-ylmagnesium bromide (15 mL, 7.5 mmol, 0.5 M in THF). The solution was stirred at -20 °C for 1 h, then the mixture was allowed to gradually warm to room temperature and stirred for additional 9 h. After completion, the reaction was quenched with saturated NH_4Cl (aq.) carefully at 0 ºC, filtered and extracted with EtOAc (10 mL×3). The combined organic layers were washed with brine (20 mL), dried over Na_2SO_4, and concentrated in vacuo. The crude product was purified by column chromatography (eluent: petroleum ether/ethyl acetate = 30/1) to give 1-phenylpent-3-en-1,1-*d*$_2$-2-ol with 95% deuterium incorporation. (677 mg, 55% yield, *Z*:*E* = 4.6:1 determined by ^{1}H NMR analysis), colorless oil. ^{1}H NMR (400 MHz, Chloroform-*d*) δ 7.32-7.29 (m, 2H), 7.26-7.21 (m, 3H), 5.71-5.43 (m, 2H), 4.68 (*Z*, d, *J* = 8.4 Hz, 0.82H), 4.28 (*E*, d, *J* = 6.6 Hz, 0.18H), 2.83-2.76 (m, 0.1H), 1.71-1.52 (m, 4H); ^{13}C NMR (100 MHz, Chloroform-*d*) δ 138.0, 137.8, 133.2, 132.4, 129.54, 129.51, 128.42, 128.40, 127.1, 126.6, 126.4, 73.4, 68.4, 17.7, 13.2; HRMS (ESI) m/z calculated for $C_{11}H_{12}D_2O$ [M+Na]$^+$: 187.1062, found: 187.1060.

2.2. General procedure the migratory dehydroxylative allylic arylation of 1,n-enaols

*Representative procedure for the synthesis of **3-39***

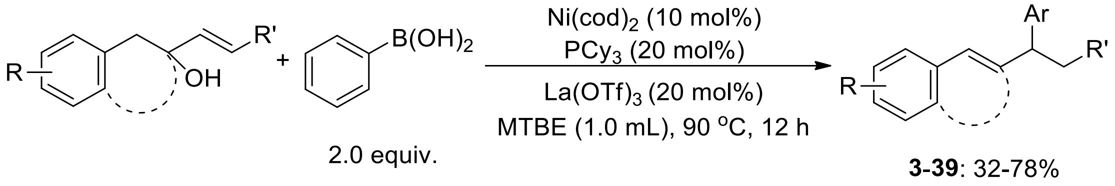

Under a nitrogen atmosphere, to a mixture of $Ni(cod)_2$ (5.5 mg, 0.02 mmol), PCy_3 (11.2 mg, 0.04 mmol), $La(OTf)_3$ (23.4 mg, 0.04 mmol), and $PhB(OH)_2$ (48.8 mg, 0.4 mmol) was added a solution of corresponding enol (0.2 mmol) in MTBE (1.0 mL). The reaction was sealed and stirred at 90 °C for 12 h. Subsequently, the reaction was cooled down to room temperature and the mixture was evaporated and purified via column chromatography on silica gel (eluent: petroleum ether/ethyl acetate) afforded desired product.

*(E)-pent-1-ene-1,3-diyldibenzene (**3**)*

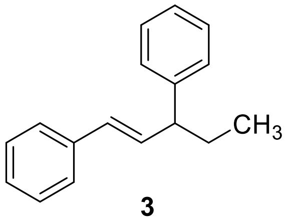

3

72% isolated yield from (*E*)-**1a** (eluent: petroleum ether), colorless oil; 73% isolated yield from (*Z*:*E* = 3:1)-**1a** (eluent: petroleum ether), colorless oil. ^{1}H NMR (400 MHz, Chloroform-*d*) δ 7.45-7.15 (m, 10H), 6.70-6.15 (m, 2H), 3.33 (q, *J* = 7.3 Hz, 1H), 1.90-1.80 (m, 2H), 0.93 (t, *J* = 7.4 Hz, 3H); ^{13}C NMR (100 MHz, Chloroform-*d*) δ 144.5, 137.6, 134.2, 129.4, 128.4, 127.7, 127.0, 126.2, 126.1, 51.0, 28.8, 12.3; HRMS (EI) m/z calculated for $C_{17}H_{18}$ $[M]^+$: 222.1403, found: 222.1400.

*(E)-1-methyl-3-(1-phenylpent-1-en-3-yl)benzene (**5**)*

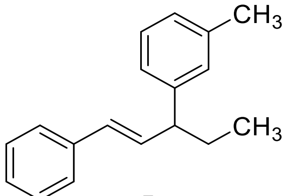

5

65% isolated yield from (*E*)-**1a** (eluent: petroleum ether), colorless oil. ^{1}H NMR (400 MHz, Chloroform-*d*) δ 7.37 (d, *J* = 7.3 Hz, 2H), 7.30 (t, *J* = 7.5 Hz, 2H), 7.25-7.16 (m, 2H), 7.11-6.96 (m, 3H), 6.55-6.07 (m, 2H), 3.29 (q, *J* = 7.4 Hz, 1H), 2.37 (s, 3H), 1.98-1.75 (m, 2H), 0.94 (t, *J* = 7.3 Hz, 3H); ^{13}C NMR (100 MHz, Chloroform-*d*) δ 144.5, 138.0, 137.7, 134.3, 129.3, 128.4, 128.3, 126.9, 126.9, 126.1, 124.6, 51.0, 28.8, 21.5, 12.3; HRMS (EI) m/z calculated for $C_{18}H_{20}$ [M]$^+$: 236.1560, found: 236.1559.

*(E)-1-methyl-4-(1-phenylpent-1-en-3-yl)benzene (**6**)*

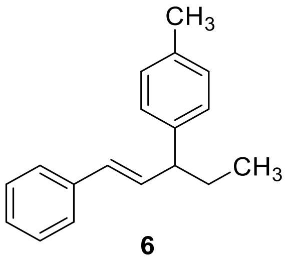

6

67% isolated yield from (*E*)-**1a** (eluent: petroleum ether), colorless oil. ^{1}H NMR (400 MHz, Chloroform-*d*) δ 7.38-7.34 (m, 2H), 7.32-7.26 (m, 2H), 7.23-7.09 (m, 5H), 6.55-5.85 (m, 2H), 3.29 (q, *J* = 7.3 Hz, 1H), 2.34 (s, 3H), 1.83 (m, 2H), 0.93 (t, *J* = 7.3 Hz, 3H); ^{13}C NMR (100 MHz, Chloroform-*d*) δ 141.5, 137.7, 135.6, 134.5, 129.3, 129.1, 128.4, 127.5, 126.9, 126.1, 50.5, 28.8, 21.0, 12.3; HRMS (EI) m/z calculated for $C_{18}H_{20}$ [M]$^+$: 236.1560, found: 236.1559.

(E)-1-(tert-butyl)-4-(1-phenylpent-1-en-3-yl)benzene (7)

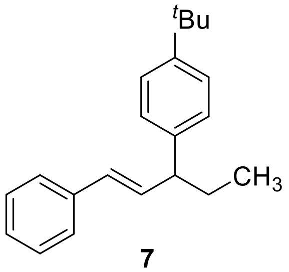

7

65% isolated yield from (*E*)-**1a** (eluent: petroleum ether), colorless oil. ^{1}H NMR (400 MHz, Chloroform-*d*) δ 7.38-7.33 (m, 4H), 7.31-7.27 (m, 2H), 7.22-7.17 (m, 3H), 6.43 (d, *J* = 15.8 Hz, 1H), 6.34 (dd, *J* = 15.8, 7.8 Hz, 1H), 3.30 (q, *J* = 7.5 Hz, 1H), 1.88-1.80 (m, 2H), 1.33 (s, 9H), 0.94 (t, *J* = 7.3 Hz, 3H); ^{13}C NMR (100 MHz, Chloroform-*d*) δ 148.9, 141.5, 137.7, 134.4, 129.3, 128.4, 127.2, 126.9, 126.1, 125.3, 50.5, 34.4, 31.4, 28.7, 12.4; HRMS (EI) m/z calculated for $C_{21}H_{26}$ $[M]^+$: 278.2029, found: 278.2029.

*(E)-1-fluoro-4-(1-phenylpent-1-en-3-yl)benzene (**8**)*

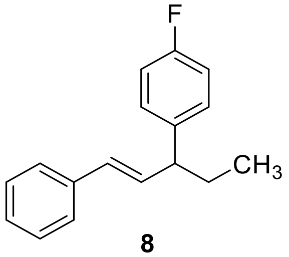

8

68% isolated yield from (*E*)-**1a** (eluent: petroleum ether), colorless oil. ^{1}H NMR (400 MHz, Chloroform-*d*) δ 7.38-7.34 (m, 2H), 7.33-7.26 (m, 2H), 7.24-7.17 (m, 3H), 7.07-6.91 (m, 2H), 6.39 (d, *J* = 15.9 Hz, 1H), 6.30 (dd, *J* = 15.8, 7.3 Hz, 1H), 3.31 (q, *J* = 7.4 Hz, 1H), 1.92-1.72 (m, 2H), 0.92 (t, *J* = 7.3 Hz, 3H); ^{13}C NMR (100 MHz, Chloroform-*d*) δ 161.4 (d, *J* = 242.4 Hz), 140.1 (d, *J* = 3.2 Hz), 137.4, 134.0, 129.6, 129.0 (d, *J* = 7.7 Hz), 128.5, 127.1, 126.1, 115.2 (d, *J* = 21.0 Hz), 50.1, 28.8, 12.2; ^{19}F NMR (376 MHz, Chloroform-*d*) δ -117.2; HRMS (EI) m/z calculated for $C_{17}H_{17}F$ $[M]^+$: 240.1309, found: 240.1312.

*(E)-1,3-dimethyl-5-(1-phenylpent-1-en-3-yl)benzene (**9**)*

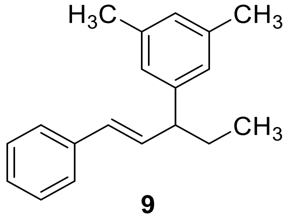

9

62% isolated yield from (*E*)-**1a** (eluent: petroleum ether), colorless oil. ^{1}H NMR (400 MHz, Chloroform-*d*) δ 7.39-7.36 (m, 2H), 7.34-7.24 (m, 2H), 7.24-7.12 (m, 1H), 6.87 (s, 3H), 6.42 (d, *J* = 15.9 Hz, 1H), 6.33 (dd, *J* = 15.8, 7.6 Hz, 1H), 3.24 (q, *J* = 7.5 Hz, 1H), 2.32 (s, 6H), 1.94-1.73 (m, 2H), 0.93 (t, *J* = 7.4 Hz, 3H); ^{13}C NMR (100 MHz, Chloroform-*d*) δ 144.5, 137.9, 137.7, 134.4, 129.2, 128.4, 127.8, 126.9, 126.1, 125.4, 51.0, 28.8, 21.4, 12.4; HRMS (EI) m/z calculated for $C_{19}H_{22}$ [M]$^{+}$: 250.1716, found: 250.1718.

*(E)-1-methoxy-4-(1-phenylpent-1-en-3-yl)benzene (**10**)*

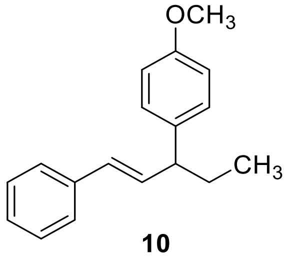

10

42% isolated yield from (*E*)-**1a** (eluent: petroleum ether/ethyl acetate = 200/1), colorless oil. ^{1}H NMR (400 MHz, Chloroform-*d*) δ 7.35-7.32 (m, 2H), 7.29-7.24 (m, 2H), 7.20-7.14 (m, 3H), 6.88-6.84 (m, 2H), 6.39-6.27 (m, 2H), 3.79 (s, 3H), 3.26 (q, *J* = 7.3 Hz, 1H), 1.85-1.73 (m, 2H), 0.90 (t, *J* = 7.4 Hz, 3H); ^{13}C NMR (100 MHz, Chloroform-*d*) δ 158.0, 137.7, 136.5, 134.6, 129.1, 128.6, 128.4, 126.9, 126.1, 113.8, 55.2, 50.0, 28.8, 12.3; HRMS (EI) m/z calculated for $C_{18}H_{20}O$ [M]$^{+}$: 252.1509, found: 252.1512.

*(E)-1-methyl-2-(3-phenylpent-1-en-1-yl)benzene (**11**)*

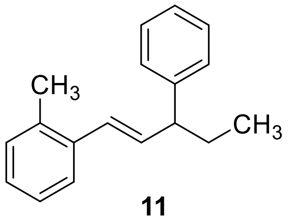

11

50% isolated yield from (*Z*)-**S13** (eluent: petroleum ether), colorless oil. ^{1}H NMR (400 MHz, Chloroform-*d*) δ 7.49-7.40 (m, 1H), 7.39-7.30 (m, 2H), 7.33-7.26 (m, 2H), 7.28-7.20 (m, 1H), 7.21-7.09 (m, 3H), 6.64 (d, *J* = 15.7 Hz, 1H), 6.23 (dd, *J* = 15.7, 8.0 Hz, 1H), 3.37 (q, *J* = 7.6 Hz, 1H), 2.35 (s, 3H), 1.91-1.83 (m, 2H), 0.97 (t, *J* = 7.4 Hz, 3H); ^{13}C NMR (101 MHz, Chloroform-*d*) δ 144.7, 136.8, 135.6, 135.1, 130.1, 128.4, 127.7, 127.4, 126.9, 126.1, 126.0, 125.6, 51.2, 28.9, 19.8, 12.3; HRMS (EI) m/z calculated for $C_{18}H_{20}$ $[M]^+$: 236.1557, found: 236.1557.

*(E)-1-methoxy-2-(3-phenylpent-1-en-1-yl)benzene (**12**)*

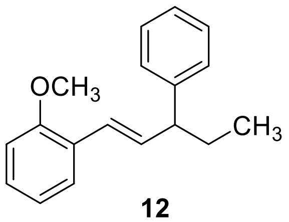

12

55% isolated yield from (*Z*:*E* = 19:1)-**S15** (eluent: petroleum ether/ethyl acetate = 200/1), colorless oil. ^{1}H NMR (400 MHz, Chloroform-*d*) δ 7.44 (dd, *J* = 7.6, 1.7 Hz, 1H), 7.35-7.25 (m, 4H), 7.24-7.13 (m, 2H), 6.94-6.82 (m, 2H), 6.78 (d, *J* = 16.4 Hz, 1H), 6.34 (dd, *J* = 16.0, 8.1 Hz, 1H), 3.84 (s, 3H), 3.35 (q, *J* = 7.6 Hz, 1H), 1.91-1.79 (m, 2H), 0.93 (t, *J* = 7.4 Hz, 3H); ^{13}C NMR (100 MHz, Chloroform-*d*) δ 156.4, 144.9, 134.6, 128.4, 128.0, 127.7, 126.6, 126.4, 126.0, 123.9, 120.6, 110.8, 55.5, 51.4, 28.9, 12.3; HRMS (EI) m/z calculated for $C_{18}H_{20}O$ $[M]^+$: 252.1509, found: 252.1508.

*(E)-1-fluoro-2-(3-phenylpent-1-en-1-yl)benzene (**13**)*

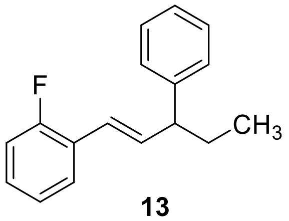

13

53% isolated yield from (*Z*:*E* = 7.3:1)-**S14** (eluent: petroleum ether), colorless oil. ^{1}H NMR (400 MHz, Chloroform-*d*) δ 7.48-7.43 (m, 1H), 7.35 (t, *J* = 7.5 Hz, 2H), 7.32-7.13 (m, 4H), 7.12-6.96 (m, 2H), 6.61 (d, *J* = 16.0 Hz, 1H), 6.44 (dd, *J* = 16.0, 8.0 Hz, 1H), 3.36 (q, *J* = 7.6 Hz, 1H), 1.93-1.82 (m, 2H), 0.95 (t, *J* = 7.4 Hz, 3H); ^{13}C NMR (100 MHz, Chloroform-*d*) δ 160.0 (d, *J* = 248.4 Hz), 144.3, 136.8 (d, *J* = 4.4 Hz), 128.5, 128.2 (d, *J* = 8.3 Hz), 127.6, 127.1 (d, *J* = 4.0 Hz), 126.2, 125.3 (d, *J* = 12.2 Hz), 123.9 (d, *J* = 3.4 Hz), 121.8 (d, *J* = 3.7 Hz), 115.6 (d, *J* = 22.3 Hz), 51.4, 28.7, 12.2; ^{19}F NMR (376 MHz, Chloroform-*d*) δ -118.7; HRMS (EI) m/z calculated for $C_{17}H_{17}F$ $[M]^{+}$: 240.1309, found: 240.1308.

*(E)-1-methyl-3-(3-phenylpent-1-en-1-yl)benzene (**14**)*

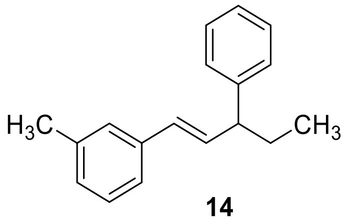

14

75% isolated yield from (*Z*:*E* = 4:1)-**S7** (eluent: petroleum ether), colorless oil. ^{1}H NMR (400 MHz, Chloroform-*d*) δ 7.39-7.33 (m, 2H), 7.31-7.28 (m, 2H), 7.27-7.18 (m, 4H), 7.09-7.02 (m, 1H), 6.51-6.23 (m, 2H), 3.35 (q, *J* = 7.3 Hz, 1H), 2.37 (s, 3H), 1.94-1.83 (m, 2H), 0.97 (t, *J* = 7.3 Hz, 3H); ^{13}C NMR (100 MHz, Chloroform-*d*) δ 144.6, 138.0, 137.6, 134.0, 129.5, 128.4, 128.3, 127.8, 127.7, 126.8, 126.1, 123.3, 51.0, 28.8, 21.4, 12.3; HRMS (EI) m/z calculated for $C_{18}H_{20}$ $[M]^{+}$: 236.1560, found: 236.1559.

*(E)-1-methoxy-3-(3-phenylpent-1-en-1-yl)benzene (**15**)*

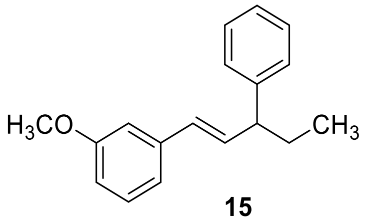

15

68% isolated yield from (*Z*)-**S9** (eluent: petroleum ether/ethyl acetate = 200/1), colorless oil. ^{1}H NMR (400 MHz, Chloroform-*d*) δ 7.36-7.32 (m, 2H), 7.28-7.20 (m, 4H), 6.97 (d, *J* = 7.6 Hz, 1H), 6.91 (s, 1H), 6.79-6.76 (m, 1H), 6.42-6.32 (m, 2H), 3.81 (s, 3H), 3.33 (q, *J* = 7.1 Hz, 1H), 1.89-1.82 (m, 2H), 0.94 (t, *J* = 7.5 Hz, 3H); ^{13}C NMR (100 MHz, Chloroform-*d*) δ 159.8, 144.4, 139.1, 134.5, 129.4, 129.3, 128.4, 127.7, 126.2, 118.8, 112.7, 111.4, 55.2, 50.9, 28.7, 12.3; HRMS (EI) m/z calculated for $C_{18}H_{20}O$ $[M]^{+}$: 252.1509, found: 252.1511.

*(E)-1-fluoro-3-(3-phenylpent-1-en-1-yl)benzene (**16**)*

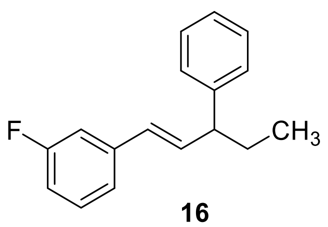

16

71% isolated yield from (*Z*)-**S8** (eluent: petroleum ether), colorless oil. ^{1}H NMR (400 MHz, Chloroform-*d*) δ 7.43-7.33 (m, 2H), 7.31-7.22 (m, 4H), 7.16-7.05 (m, 2H), 6.95-6.90 (m, 1H), 6.51-6.33 (m, 2H), 3.38-3.33 (m, 1H), 1.94-1.83 (m, 2H), 0.96 (t, *J* = 7.4 Hz, 3H); ^{13}C NMR (100 MHz, Chloroform-*d*) δ 163.1 (d, *J* = 244.8 Hz), 144.1, 140.0 (d, *J* = 7.7 Hz), 135.7, 129.8 (d, *J* = 8.5 Hz), 128.5, 128.4 (d, *J* = 1.8 Hz), 127.7, 126.3, 122.0 (d, *J* = 2.7 Hz), 113.7 (d, *J* = 21.4 Hz), 112.5 (d, *J* = 21.6 Hz), 50.9, 28.7, 12.2; ^{19}F NMR (376 MHz, Chloroform-*d*) δ -113.8; HRMS (EI) m/z calculated for $C_{17}H_{17}F$ $[M]^{+}$: 240.1309, found: 240.1309.

*(E)-1-(3-phenylpent-1-en-1-yl)-3-(trifluoromethyl)benzene (**17**)*

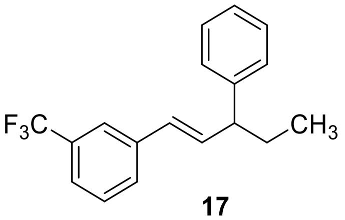

17

66% isolated yield from (*Z*)-**S18** (eluent: petroleum ether), colorless oil. ^{1}H NMR (400 MHz, Chloroform-*d*) δ 7.58 (s, 1H), 7.51-7.49 (m, 1H), 7.45-7.32 (m, 4H), 7.26-7.21 (m, 3H), 6.46-6.37 (m, 2H), 3.36-3.31 (m, 1H), 1.91-1.80 (m, 2H), 0.93 (t, *J* = 7.4 Hz, 3H); ^{13}C NMR (100 MHz, Chloroform-*d*) δ 144.0, 138.3, 136.3, 130.8 (q, *J* = 32.0 Hz), 129.3, 128.9, 128.6, 128.2, 127.7, 126.4, 124.2 (q, *J* = 271.6 Hz), 123.5 (q, *J* = 3.8 Hz), 122.7 (q, *J* = 4.0 Hz), 51.0, 28.6, 12.3; ^{19}F NMR (376 MHz, Chloroform-*d*) δ -62.7; HRMS (EI) m/z calculated for $C_{18}H_{17}F_3$ $[M]^+$: 290.1277, found: 290.1278.

*(E)-1-methyl-4-(3-phenylpent-1-en-1-yl)benzene (**18**)*

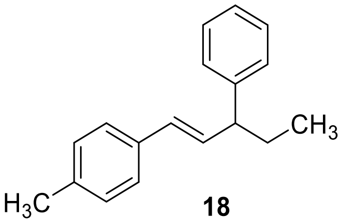

18

71% isolated yield from (*Z*:*E* = 3:1)-**S1** (eluent: petroleum ether), colorless oil. ^{1}H NMR (400 MHz, Chloroform-*d*) δ 7.37-7.31 (m, 2H), 7.29-7.20 (m, 5H), 7.11 (d, *J* = 7.9 Hz, 2H), 6.40 (d, *J* = 15.8 Hz, 1H), 6.30 (dd, *J* = 15.8, 7.5 Hz, 1H), 3.32 (q, *J* = 7.4 Hz, 1H), 2.34 (s, 3H), 1.91-1.80 (m, 2H), 0.94 (t, *J* = 7.4 Hz, 3H); ^{13}C NMR (101 MHz, Chloroform-*d*) δ 144.7, 136.7, 134.8, 133.2, 129.3, 129.1, 128.4, 127.7, 126.1, 126.0, 50.9, 28.8, 21.1, 12.3; HRMS (EI) m/z calculated for $C_{18}H_{20}$ $[M]^+$: 236.1560, found: 236.1561.

*(E)-1-(tert-butyl)-4-(3-phenylpentー1-en-1-yl)benzene (**19**)*

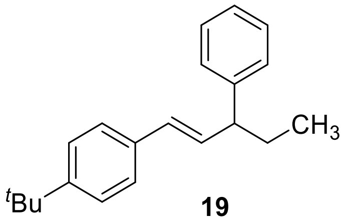

19

68% isolated yield from (*Z*:*E* = 3.5:1)-**S2** (eluent: petroleum ether), colorless oil. ^{1}H NMR (400 MHz, Chloroform-*d*) δ 7.33-7.26 (m, 6H), 7.25-7.16 (m, 3H), 6.38 (d, *J* = 15.9 Hz, 1H), 6.28 (dd, *J* = 15.8, 7.6 Hz, 1H), 3.29 (q, *J* = 7.5 Hz, 1H), 1.82 (pd, *J* = 7.4, 1.2 Hz, 2H), 1.30 (s, 9H), 0.91 (t, *J* = 7.3 Hz, 3H); ^{13}C NMR (100 MHz, Chloroform-*d*) δ 150.1, 144.7, 134.9, 133.4, 129.2, 128.4, 127.7, 126.1, 125.8, 125.4, 50.9, 34.5, 31.3, 28.8, 12.3; HRMS (EI) m/z calculated for $C_{21}H_{26}$ $[M]^+$: 278.2029, found: 278.2031.

*(E)-4-(3-phenylpent-1-en-1-yl)-1,1'-biphenyl (**20**)*

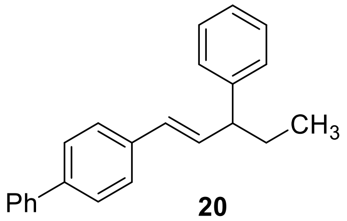

20

70% isolated yield from (*Z*)-**S11** (eluent: petroleum ether), white solid. ^{1}H NMR (400 MHz, Chloroform-*d*) δ 7.63-7.58 (m, 2H), 7.57-7.53 (m, 2H), 7.47-7.42 (m, 4H), 7.38-7.32 (m, 3H), 7.31-7.21 (m, 3H), 6.55-6.31 (m, 2H), 3.36 (q, *J* = 7.3 Hz, 1H), 1.93-1.82 (m, 2H), 0.95 (t, *J* = 7.4 Hz, 3H); ^{13}C NMR (100 MHz, Chloroform-*d*) δ 144.5, 140.8, 139.8, 136.7, 134.4, 129.0, 128.7, 128.5, 127.7, 127.16, 127.14, 126.9, 126.5, 126.2, 51.0, 28.8, 12.3; HRMS (EI) m/z calculated for $C_{23}H_{22}$ $[M]^+$: 298.1716, found: 298.1720.

*(E)-1-methoxy-4-(3-phenylpent-1-en-1-yl)benzene (**21**)*

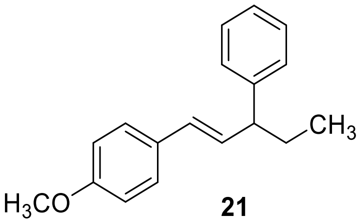

21

74% isolated yield from (*Z*:*E* = 3.5:1)-**S4** (eluent: petroleum ether/ethyl acetate = 200/1), colorless oil. ^{1}H NMR (400 MHz, Chloroform-*d*) δ 7.32-7.22 (m, 6H), 7.21-7.16 (m, 1H), 6.81 (d, *J* = 8.8 Hz, 2H), 6.34 (d, *J* = 15.8 Hz, 1H), 6.18 (dd, *J* = 15.8, 7.8 Hz, 1H), 3.78 (s, 3H), 3.28 (q, *J* = 7.5 Hz, 1H), 1.85-1.77 (m, 2H), 0.90 (t, *J* = 7.4 Hz, 3H); ^{13}C NMR (100 MHz, Chloroform-*d*) δ 158.8, 144.8, 132.1, 130.4, 128.8, 128.4, 127.7, 127.2, 126.1, 113.9, 55.3, 50.9, 28.9, 12.3; HRMS (EI) m/z calculated for $C_{18}H_{20}O$ [M]$^{+}$: 252.1509, found: 252.1507.

*(E)-1-fluoro-4-(3-phenylpent-1-en-1-yl)benzene (**22**)*

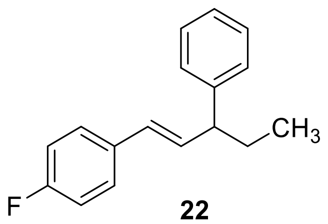

22

78% isolated yield from (*Z*:*E* = 4:1)-**S5** (eluent: petroleum ether), colorless oil. ^{1}H NMR (400 MHz, Chloroform-*d*) δ 7.39-7.30 (m, 4H), 7.29-7.20 (m, 3H), 6.99 (t, *J* = 8.7 Hz, 2H), 6.39 (d, *J* = 15.8 Hz, 1H), 6.27 (dd, *J* = 15.8, 7.6 Hz, 1H), 3.32 (q, *J* = 7.5 Hz, 1H), 1.90-1.81 (m, 2H), 0.94 (t, *J* = 7.4 Hz, 3H); ^{13}C NMR (100 MHz, Chloroform-*d*) δ 162.0 (d, *J* = 245.8 Hz), 144.4, 134.0, 133.8 (d, *J* = 3.1 Hz), 128.5, 128.3, 127.7, 127.5 (d, *J* = 7.8 Hz), 126.2, 115.3 (d, *J* = 21.4 Hz), 50.9, 28.8, 12.3; ^{19}F NMR (376 MHz, Chloroform-*d*) δ -115.5; HRMS (EI) m/z calculated for $C_{17}H_{17}F$ [M]$^{+}$: 240.1309, found: 240.1307.

*(E)-1-chloro-4-(3-phenylpent-1-en-1-yl)benzene (**23**)*

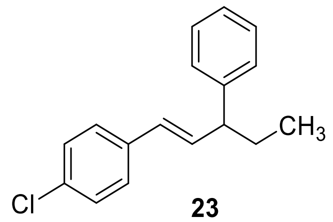

23

32% isolated yield from (*Z*:*E* = 3.8:1)-**S3** (eluent: petroleum ether), colorless oil. ^{1}H NMR (400 MHz, Chloroform-*d*) δ 7.35-7.31 (m, 2H), 7.30-7.18 (m, 7H), 6.43-6.25 (m, 2H), 3.31 (q, *J* = 7.1 Hz, 1H), 1.89-1.78 (m, 2H), 0.92 (t, *J* = 7.4 Hz, 3H); ^{13}C NMR (100 MHz, Chloroform-*d*) δ 144.2, 136.1, 135.0, 132.5, 128.6, 128.5, 128.2, 127.7, 127.3, 126.3, 51.0, 28.7, 12.3; HRMS (EI) m/z calculated for $C_{17}H_{17}{}^{35}Cl$ $[M]^+$: 256.1013, found: 256.1014.

*(E)-1-(3-phenylpent-1-en-1-yl)-4-(trifluoromethyl)benzene (**24**)*

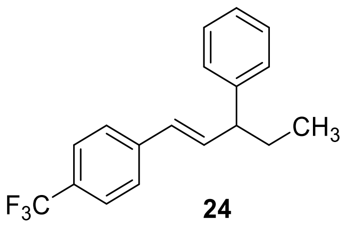

24

65% isolated yield from (*Z*)-**S12** (eluent: petroleum ether), colorless oil. ^{1}H NMR (400 MHz, Chloroform-*d*) δ 7.53 (d, *J* = 8.2 Hz, 2H), 7.43 (d, *J* = 8.2 Hz, 2H), 7.37-7.32 (m, 2H), 7.26-7.21 (m, 3H), 6.48-6.39 (m, 2H), 3.34 (q, *J* = 6.8 Hz, 1H), 1.91-1.80 (m, 2H), 0.92 (t, *J* = 7.4 Hz, 3H); ^{13}C NMR (100 MHz, Chloroform-*d*) δ 143.9, 141.1, 137.0, 128.6, 128.5 (q, *J* = 32.1 Hz), 128.2, 127.7, 126.4, 126.2, 125.4 (q, *J* = 3.9 Hz), 124.3 (q, *J* = 270.0 Hz), 51.0, 28.6, 12.2; ^{19}F NMR (376 MHz, Chloroform-*d*) δ -62.4; HRMS (EI) m/z calculated for $C_{18}H_{17}F_3$ $[M]^+$: 290.1277, found: 290.1280.

*(E)-1-(3-phenylpent-1-en-1-yl)-4-(trifluoromethoxy)benzene (**25**)*

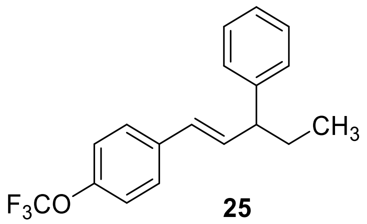

25

69% isolated yield from (*Z*:*E* = 3:1)-**S19** (eluent: petroleum ether), colorless oil. ^{1}H NMR (400 MHz, Chloroform-*d*) δ 7.38-7.31 (m, 4H), 7.28-7.21 (m, 3H), 7.15 (d, *J* = 8.3 Hz, 2H), 6.43-6.31 (m, 2H), 3.34 (q, *J* = 7.3 Hz, 1H), 1.90-1.81 (m, 2H), 0.94 (t, *J* = 7.3 Hz, 3H); ^{13}C NMR (100 MHz, Chloroform-*d*) δ 148.1, 144.2, 136.4, 135.4, 128.5, 128.0, 127.7, 127.3, 126.3, 121.0, 120.5 (q, *J* = 255.7 Hz), 51.0, 28.7, 12.2; ^{19}F NMR (376 MHz, Chloroform-*d*) δ -57.9; HRMS (EI) m/z calculated for $C_{18}H_{17}F_3O$ $[M]^+$: 306.1226, found: 306.1227.

*(E)-1-(3-phenylpent-1-en-1-yl)naphthalene (**26**)*

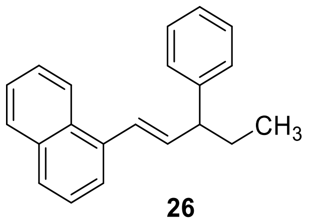

26

70% isolated yield from (*Z*)-**S6** (eluent: petroleum ether), slightly yellow oil. ^{1}H NMR (400 MHz, Chloroform-*d*) δ 8.15-8.10 (m, 1H), 7.89-7.85 (m, 1H), 7.78 (d, *J* = 8.2 Hz, 1H), 7.60 (d, *J* = 7.1 Hz, 1H), 7.56-7.48 (m, 2H), 7.45 (dd, *J* = 8.2, 7.2 Hz, 1H), 7.41-7.33 (m, 4H), 7.30-7.25 (m, 1H), 7.18 (d, *J* = 15.5 Hz, 1H), 6.40 (dd, *J* = 15.6, 7.9 Hz, 1H), 3.50 (q, *J* = 7.8 Hz, 1H), 2.01-1.90 (m, 2H), 1.03 (t, *J* = 7.3 Hz, 3H); ^{13}C NMR (100 MHz, Chloroform-*d*) δ 144.5, 137.5, 135.5, 133.6, 131.2, 128.5, 128.5, 127.7, 127.4, 126.8, 126.2, 125.8, 125.62, 125.60, 123.9, 123.7, 51.3, 29.0, 12.4; HRMS (EI) m/z calculated for $C_{21}H_{20}$ $[M]^+$: 272.1560, found: 272.1560.

*(E)-2-(3-phenylpent-1-en-1-yl)naphthalene (**27**)*

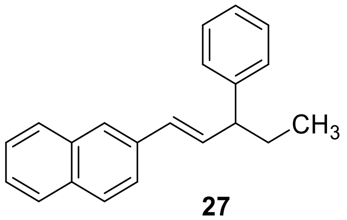

27

78% isolated yield from (*Z*:*E* = 16:1)-**S10** (eluent: petroleum ether), slightly yellow oil. ^{1}H NMR (400 MHz, Chloroform-*d*) δ 7.80-7.76 (m, 3H), 7.70 (s, 1H), 7.60 (dd, *J* = 8.6, 1.7 Hz, 1H), 7.49-7.40 (m, 2H), 7.38-7.34 (m, 2H), 7.33-7.29 (m, 2H), 7.27-7.22 (m, 1H), 6.59 (d, *J* = 15.9 Hz, 1H), 6.49 (dd, *J* = 15.8, 7.5 Hz, 1H), 3.39 (q, *J* = 7.4 Hz, 1H), 1.96-1.85 (m, 2H), 0.97 (t, *J* = 7.3 Hz, 3H); ^{13}C NMR (100 MHz, Chloroform-*d*) δ 144.5, 135.1, 134.7, 133.6, 132.7, 129.5, 128.5, 128.0, 127.8, 127.7, 127.6, 126.2, 126.1, 125.7, 125.5, 123.6, 51.1, 28.8, 12.3; HRMS (EI) m/z calculated for $C_{21}H_{20}$ $[M]^+$: 272.1560, found: 272.1560.

*(E)-1-methyl-3-(3-phenylpent-1-en-1-yl)-1H-indole (**28**)*

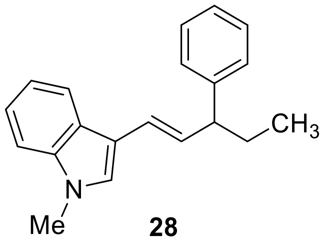

28

48% isolated yield from (*Z*:*E* = 6.7:1)-**S20** (eluent: petroleum ether/ethyl acetate = 150/1), yellow oil. ^{1}H NMR (400 MHz, Chloroform-*d*) δ 7.84 (d, *J* = 8.0 Hz, 1H), 7.36-7.19 (m, 7H), 7.17-7.13 (m, 1H), 7.05 (s, 1H), 6.56 (d, *J* = 16.0 Hz, 1H), 6.29 (dd, *J* = 16.0, 7.8 Hz, 1H), 3.74 (s, 3H), 3.33 (q, *J* = 7.5 Hz, 1H), 1.91-1.83 (m, 2H), 0.95 (t, *J* = 7.3 Hz, 3H); ^{13}C NMR (100 MHz, Chloroform-*d*) δ 145.4, 137.5, 130.6, 128.4, 127.7, 127.2, 126.2, 125.9, 121.9, 121.8, 120.1, 119.6, 113.8, 109.3, 51.5, 32.7, 29.2, 12.4; HRMS (EI) m/z calculated for $C_{20}H_{21}N$ $[M]^+$: 275.1669, found: 275.1665.

*(E)-2-(3-phenylpent-1-en-1-yl)thiophene (**29**)*

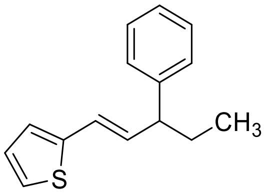

29

51% isolated yield from (*Z*:*E* = 13:1)-**S16** (eluent: petroleum ether), yellow oil. ^{1}H NMR (400 MHz, Chloroform-*d*) δ 7.35-7.31 (m, 2H), 7.25-7.20 (m, 3H), 7.09 (d, *J* = 5.1 Hz, 1H), 6.93 (dd, *J* = 5.1, 3.5 Hz, 1H), 6.88 (d, *J* = 3.0 Hz, 1H), 6.51 (d, *J* = 15.7 Hz, 1H), 6.20 (dd, *J* = 15.7, 7.7 Hz, 1H), 3.28 (q, *J* = 6.9, 6.4 Hz, 1H), 1.88-1.77 (m, 2H), 0.92 (t, *J* = 7.4 Hz, 3H); ^{13}C NMR (100 MHz, Chloroform-*d*) δ 144.1, 142.8, 134.1, 128.5, 127.7, 127.2, 126.2, 124.7, 123.3, 122.7, 50.8, 28.7, 12.3; HRMS (EI) m/z calculated for $C_{15}H_{16}S$ $[M]^{+}$: 228.0967, found: 228.0969.

*(E)-3-(3-phenylpent-1-en-1-yl)thiophene (**30**)*

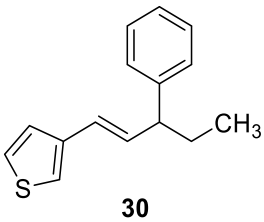

30

60% isolated yield from (*Z*:*E* = 16:1)-**S17** (eluent: petroleum ether), yellow oil. ^{1}H NMR (400 MHz, Chloroform-*d*) δ 7.34-7.30 (m, 2H), 7.25-7.17 (m, 5H), 7.07 (d, *J* = 2.2 Hz, 1H), 6.41 (d, *J* = 15.8 Hz, 1H), 6.20 (dd, *J* = 15.8, 7.7 Hz, 1H), 3.28 (q, *J* = 7.5 Hz, 1H), 1.88-1.77 (m, 2H), 0.92 (t, *J* = 7.3 Hz, 3H); ^{13}C NMR (100 MHz, Chloroform-*d*) δ 144.5, 140.2, 134.2, 128.4, 127.7, 126.2, 125.8, 125.0, 123.7, 120.9, 50.8, 28.7, 12.3; HRMS (EI) m/z calculated for $C_{15}H_{16}S$ $[M]^{+}$: 228.0967, found: 228.0968.

*(E)-but-1-ene-1,3-diyldibenzene (**31**)*

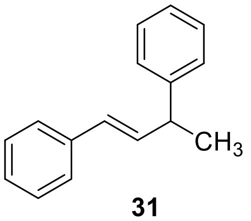

31

63% isolated yield from **S21** (eluent: petroleum ether), colorless oil. ^{1}H NMR (400 MHz, Chloroform-*d*) δ 7.35-7.32 (m, 2H), 7.31-7.25 (m, 6H), 7.22-7.15 (m, 2H), 6.44-6.34 (m, 2H), 3.66-3.60 (m, 1H), 1.46 (d, *J* = 6.9 Hz, 3H); ^{13}C NMR (100 MHz, Chloroform-*d*) δ 145.6, 137.6, 135.2, 128.51, 128.47, 127.3, 127.0, 126.2, 126.1, 42.6, 21.2; HRMS (EI) m/z calculated for $C_{16}H_{16}$ $[M]^+$: 208.1247, found: 208.1245.

*(E)-hex-1-ene-1,3-diyldibenzene (**32**)*

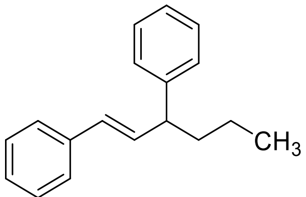

32

67% isolated yield from **S22** (eluent: petroleum ether), colorless oil. ^{1}H NMR (400 MHz, Chloroform-*d*) δ 7.41-7.27 (m, 8H), 7.26-7.20 (m, 2H), 6.46-6.34 (m, 2H), 3.47 (q, *J* = 7.3 Hz, 1H), 1.87-1.78 (m, 2H), 1.46-1.28 (m, 2H), 0.96 (t, *J* = 7.3 Hz, 3H); ^{13}C NMR (101 MHz, Chloroform-*d*) δ 144.7, 137.6, 134.4, 129.2, 128.5, 128.4, 127.6, 127.0, 126.1, 126.1, 48.9, 38.1, 20.7, 14.0; HRMS (EI) m/z calculated for $C_{18}H_{20}$ $[M]^+$: 236.1560, found: 236.1562.

*(E)-hept-1-ene-1,3-diyldibenzene (**33**)*

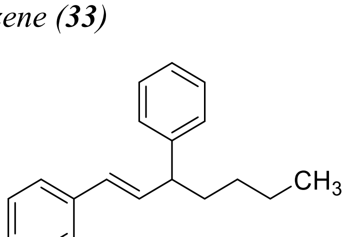

33

62% isolated yield from **S23** (eluent: petroleum ether), colorless oil. ^{1}H NMR (400

MHz, Chloroform-*d*) δ 7.38-7.26 (m, 8H), 7.25-7.18 (m, 2H), 6.44-6.32 (m, 2H), 3.41 (q, *J* = 7.3 Hz, 1H), 1.87-1.78 (m, 2H), 1.41-1.22 (m, 4H), 0.90 (t, *J* = 7.3 Hz, 3H); ^{13}C NMR (100 MHz, Chloroform-*d*) δ 144.8, 137.6, 134.5, 129.2, 128.46, 128.44, 127.6, 127.0, 126.14, 126.12, 49.2, 35.6, 29.8, 22.7, 14.0; HRMS (EI) m/z calculated for $C_{19}H_{22}$ $[M]^+$: 250.1716, found: 250.1719.

*(E)-oct-1-ene-1,3-diyldibenzene (**34**)*

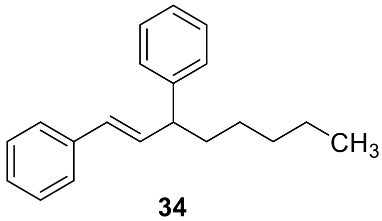

34

61% isolated yield from **S24** (eluent: petroleum ether), colorless oil. ^{1}H NMR (400 MHz, Chloroform-*d*) δ 7.39-7.25 (m, 8H), 7.25-7.18 (m, 2H), 6.44-6.32 (m, 2H), 3.42 (q, *J* = 7.3 Hz, 1H), 1.84-1.79 (m, 2H), 1.41-1.24 (m, 6H), 0.90-0.87 (m, 3H); ^{13}C NMR (100 MHz, Chloroform-*d*) δ 144.7, 137.6, 134.5, 129.2, 128.45, 128.43, 127.6, 127.0, 126.13, 126.11, 49.2, 35.9, 31.8, 27.3, 22.6, 14.1; HRMS (EI) m/z calculated for $C_{20}H_{24}$ $[M]^+$: 264.1873, found: 264.1875.

*(E)-undec-1-ene-1,3-diyldibenzene (**35**)*

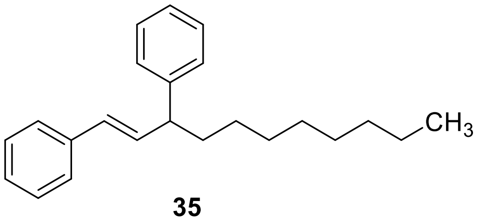

35

55% isolated yield from **S25** (eluent: petroleum ether), colorless oil. ^{1}H NMR (400 MHz, Chloroform-*d*) δ 7.37-7.24 (m, 8H), 7.23-7.17 (m, 2H), 6.42-6.31 (m, 2H), 3.41 (q, *J* = 7.3 Hz, 1H), 1.83-1.77 (m, 2H), 1.39-1.19 (m, 12H), 0.88 (t, *J* = 6.8 Hz, 3H); ^{13}C NMR (100 MHz, Chloroform-*d*) δ 144.8, 137.6, 134.5, 129.2, 128.45, 128.43, 127.6, 127.0, 126.13, 126.11, 49.2, 35.9, 31.9, 29.6, 29.5, 29.3, 27.6, 22.7, 14.1; HRMS (EI) m/z calculated for $C_{23}H_{30}$ $[M]^+$: 306.2342, found: 306.2344.

*2-(1-phenylpropyl)-1H-indene (**37**)*

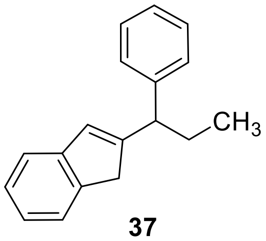

37

59% isolated yield from (*E*)-**1b** (eluent: petroleum ether), colorless oil; 58% isolated yield from (*Z*)-**1b** (eluent: petroleum ether), colorless oil. ^{1}H NMR (400 MHz, Chloroform-*d*) δ 7.34-7.28 (m, 4H), 7.27-7.19 (m, 4H), 7.10 (td, *J* = 7.3, 1.2 Hz, 1H), 6.67 (s, 1H), 3.64 (t, *J* = 7.6 Hz, 1H), 3.22 (s, 2H), 2.17-2.07 (m, 1H), 2.01-1.90 (m, 1H), 0.94 (t, *J* = 7.3 Hz, 3H); ^{13}C NMR (100 MHz, Chloroform-*d*) δ 153.4, 145.2, 144.2, 143.2, 128.4, 127.9, 126.2, 126.1, 123.8, 123.4, 120.3, 49.7, 40.0, 27.7, 12.6; HRMS (EI) m/z calculated for $C_{18}H_{18}$ [M]$^+$: 234.1403, found: 234.1403.

*3-(1-phenylpropyl)-1,2-dihydronaphthalene (**38**)*

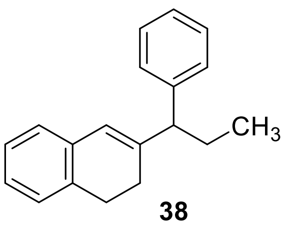

38

56% isolated yield from (*Z*)-**S26** (eluent: petroleum ether), colorless oil. ^{1}H NMR (400 MHz, Chloroform-*d*) δ 7.32-7.26 (m, 4H), 7.23-7.14 (m, 2H), 7.13-7.05 (m, 3H), 6.44 (s, 1H), 3.29 (t, *J* = 7.5 Hz, 1H), 2.74-2.66 (m, 2H), 2.10 (td, *J* = 8.0, 4.0 Hz, 2H), 2.00 (dt, *J* = 13.3, 7.2 Hz, 1H), 1.86 (dt, *J* = 13.4, 7.4 Hz, 1H), 0.93 (t, *J* = 7.3 Hz, 3H); ^{13}C NMR (100 MHz, Chloroform-*d*) δ 144.2, 143.5, 134.8, 134.7, 128.2, 128.1, 127.1, 126.4, 126.3, 126.2, 125.7, 122.4, 54.3, 28.3, 25.9, 25.5, 12.5; HRMS (EI) m/z calculated for $C_{19}H_{20}$ [M]$^+$: 248.1560, found: 248.1560.

*8-(1-phenylpropyl)-6,7-dihydro-5H-benzo[7]annulene (**39**)*

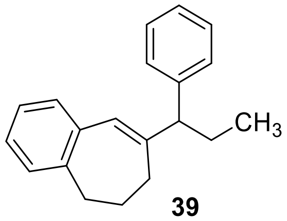

39

50% isolated yield from (*Z*)-**S27** (eluent: petroleum ether), colorless oil. ^{1}H NMR (400 MHz, Chloroform-*d*) δ 7.32-7.28 (m, 4H), 7.24-7.16 (m, 3H), 7.11-7.07 (m, 2H), 6.53 (s, 1H), 3.31 (t, *J* = 7.6 Hz, 1H), 2.67-2.60 (m, 2H), 2.09-2.01 (m, 2H), 2.00-1.87 (m, 2H), 1.86-1.79 (m, 2H), 0.96 (t, *J* = 7.3 Hz, 3H); ^{13}C NMR (100 MHz, Chloroform-*d*) δ 146.6, 144.2, 141.1, 137.6, 129.8, 128.9, 128.1, 128.0, 126.1, 126.0, 125.8, 125.8, 56.5, 34.4, 30.8, 30.7, 25.8, 12.6; HRMS (EI) m/z calculated for $C_{20}H_{22}$ $[M]^{+}$: 262.1716, found: 262.1719.

2.3. Regio- and stereoconvergent reactions

a) Reactivity comparison of (E)- and (Z)-enols

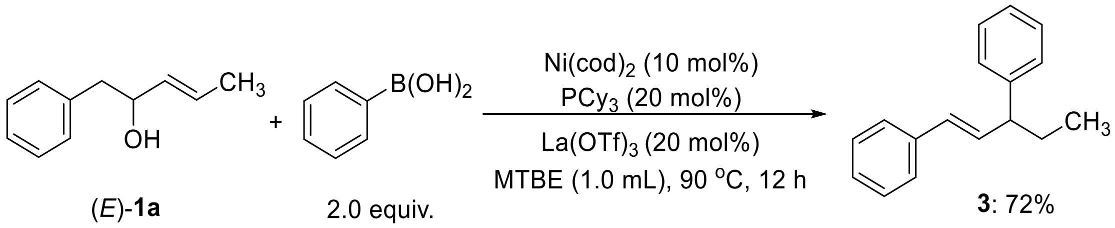

Under a nitrogen atmosphere, to a mixture of $Ni(cod)_2$ (5.5 mg, 0.02 mmol), PCy_3 (11.2 mg, 0.04 mmol), $La(OTf)_3$ (23.4 mg, 0.04 mmol), and $PhB(OH)_2$ (48.8 mg, 0.4 mmol) was added a solution of enol (*E*)-**1a** (32.4 mg, 0.2 mmol) in MTBE (1.0 mL). The reaction was sealed and stirred at 90 °C for 12 h. Subsequently, the reaction was cooled down to room temperature and the mixture was evaporated and purified via column chromatography on silica gel (eluent: petroleum ether) afforded **3** (32.0 mg, 72%): colorless oil.

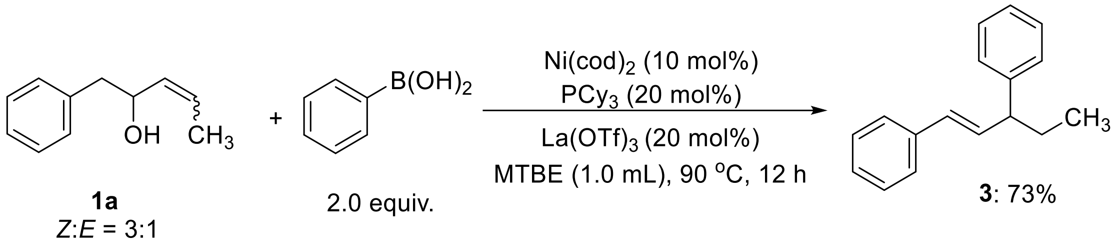

Under a nitrogen atmosphere, to a mixture of $Ni(cod)_2$ (5.5 mg, 0.02 mmol), PCy_3 (11.2 mg, 0.04 mmol), $La(OTf)_3$ (23.4 mg, 0.04 mmol), and $PhB(OH)_2$ (48.8 mg, 0.4 mmol) was added a solution of enol (*Z:E* = 3:1)-**1a** (32.4 mg, 0.2 mmol) in MTBE (1.0 mL). The reaction was sealed and stirred at 90 °C for 12 h. Subsequently, the reaction was cooled down to room temperature and the mixture was evaporated and purified via column chromatography on silica gel (eluent: petroleum ether) afforded **3** (32.5 mg, 73%): colorless oil.

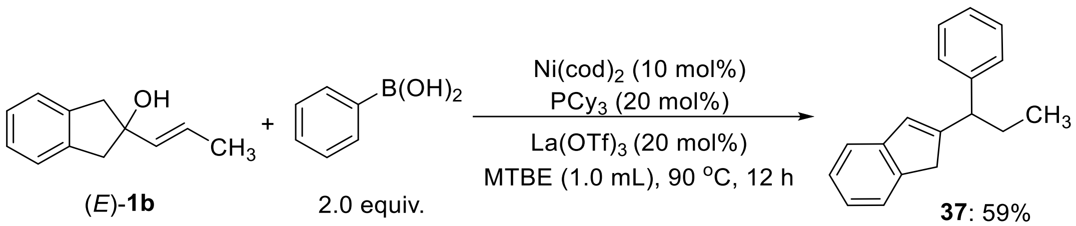

Under a nitrogen atmosphere, to a mixture of Ni(cod)$_2$ (5.5 mg, 0.02 mmol), PCy$_3$ (11.2 mg, 0.04 mmol), La(OTf)$_3$ (23.4 mg, 0.04 mmol), and PhB(OH)$_2$ (48.8 mg, 0.4 mmol) was added a solution of enol (*E*)-**1b** (34.8 mg, 0.2 mmol) in MTBE (1.0 mL). The reaction was sealed and stirred at 90 ºC for 12 h. Subsequently, the reaction was cooled down to room temperature and the mixture was evaporated and purified via column chromatography on silica gel (eluent: petroleum ether) afforded **37** (27.6 mg, 59%): colorless oil.

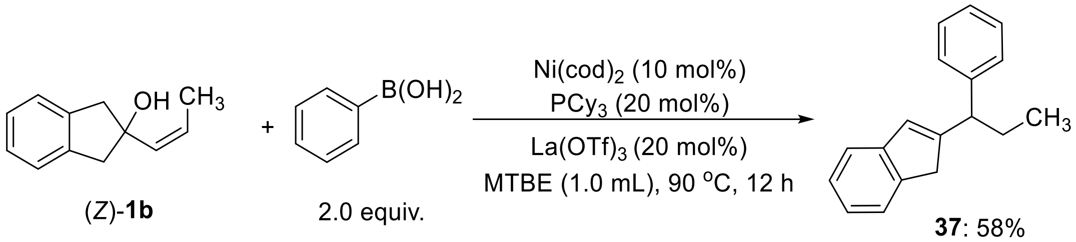

Under a nitrogen atmosphere, to a mixture of Ni(cod)$_2$ (5.5 mg, 0.02 mmol), PCy$_3$ (11.2 mg, 0.04 mmol), La(OTf)$_3$ (23.4 mg, 0.04 mmol), and PhB(OH)$_2$ (48.8 mg, 0.4 mmol) was added a solution of enol (*Z*)-**1b** (34.8 mg, 0.2 mmol) in MTBE (1.0 mL). The reaction was sealed and stirred at 90 ºC for 12 h. Subsequently, the reaction was cooled down to room temperature and the mixture was evaporated and purified via column chromatography on silica gel (eluent: petroleum ether) afforded **37** (27.1 mg, 58%): colorless oil.

b) Reaction of Enols with OH group or olefin at different positions

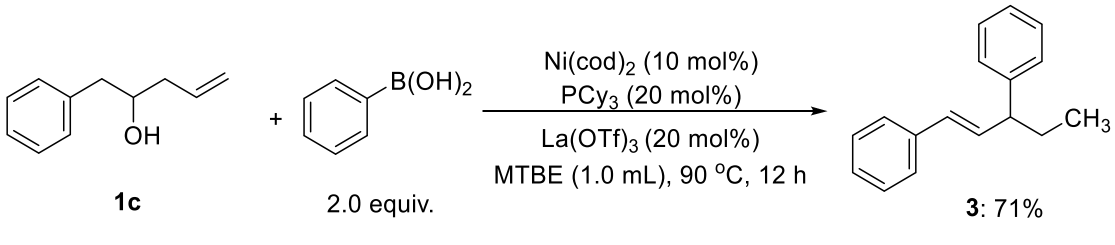

Under a nitrogen atmosphere, to a mixture of $Ni(cod)_2$ (5.5 mg, 0.02 mmol), PCy_3 (11.2 mg, 0.04 mmol), $La(OTf)_3$ (23.4 mg, 0.04 mmol), and $PhB(OH)_2$ (48.8 mg, 0.4 mmol) was added a solution of enol **1c** (32.4 mg, 0.2 mmol) in MTBE (1.0 mL). The reaction was sealed and stirred at 90 °C for 12 h. Subsequently, the reaction was cooled down to room temperature and the mixture was evaporated and purified via column chromatography on silica gel (eluent: petroleum ether) afforded **3** (31.5 mg, 71%): colorless oil.

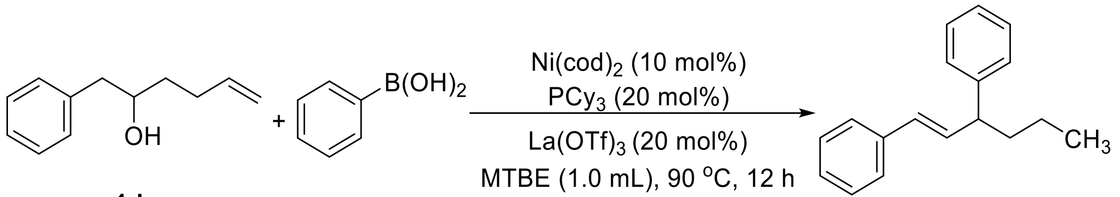

1d 2.0 equiv. **32**: 38%

Under a nitrogen atmosphere, to a mixture of $Ni(cod)_2$ (5.5 mg, 0.02 mmol), PCy_3 (11.2 mg, 0.04 mmol), $La(OTf)_3$ (23.4 mg, 0.04 mmol), and $PhB(OH)_2$ (48.8 mg, 0.4 mmol) was added a solution of enol **1d** (35.3 mg, 0.2 mmol) in MTBE (1.0 mL). The reaction was sealed and stirred at 90 °C for 12 h. Subsequently, the reaction was cooled down to room temperature and the mixture was evaporated and purified via column chromatography on silica gel (eluent: petroleum ether) afforded **32** (17.9 mg, 38%): colorless oil.

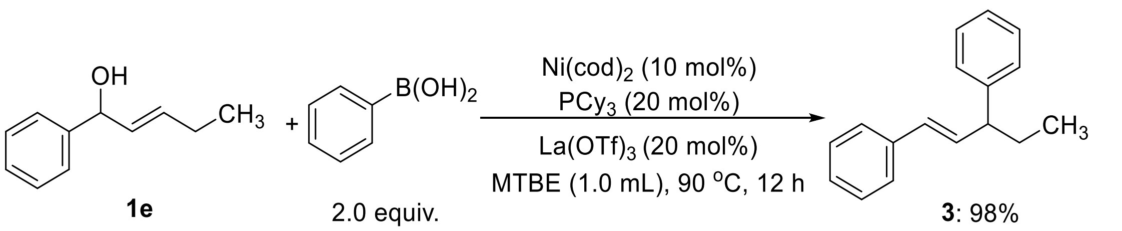

1e 2.0 equiv. **3**: 98%

Under a nitrogen atmosphere, to a mixture of $Ni(cod)_2$ (5.5 mg, 0.02 mmol), PCy_3 (11.2 mg, 0.04 mmol), $La(OTf)_3$ (23.4 mg, 0.04 mmol), and $PhB(OH)_2$ (48.8 mg, 0.4 mmol) was added a solution of enol **1e** (32.4 mg, 0.2 mmol) in MTBE (1.0 mL). The reaction was sealed and stirred at 90 °C for 12 h. Subsequently, the reaction was cooled down to room temperature and the mixture was evaporated and purified via column

chromatography on silica gel (eluent: petroleum ether) afforded **3** (43.6 mg, 98%): colorless oil.

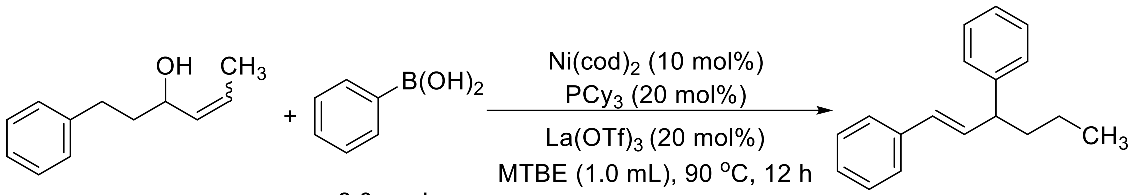

1f
Z:*E* = 4.9:1

32: 42%

Under a nitrogen atmosphere, to a mixture of Ni(cod)$_2$ (5.5 mg, 0.02 mmol), PCy$_3$ (11.2 mg, 0.04 mmol), La(OTf)$_3$ (23.4 mg, 0.04 mmol), and PhB(OH)$_2$ (48.8 mg, 0.4 mmol) was added a solution of enol **1f** (*Z*:*E* = 4.9:1, 35.3 mg, 0.2 mmol) in MTBE (1.0 mL). The reaction was sealed and stirred at 90 °C for 12 h. Subsequently, the mixture was cooled down to room temperature and then concentrated under reduced pressure. The yield of **32** was determined by ^{1}H NMR analysis using dibromomethane as the internal standard.

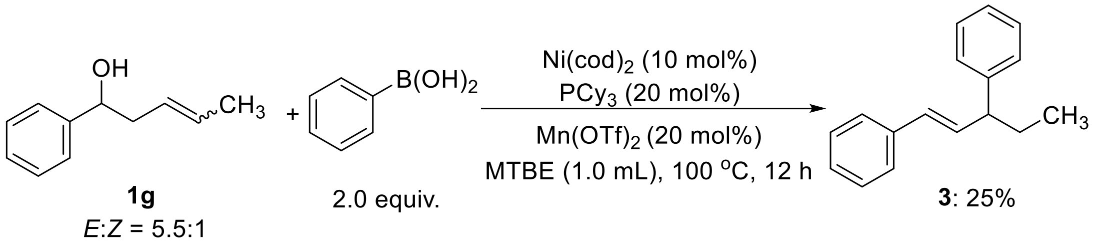

1g
E:*Z* = 5.5:1

3: 25%

Under a nitrogen atmosphere, to a mixture of Ni(cod)$_2$ (5.5 mg, 0.02 mmol), PCy$_3$ (11.2 mg, 0.04 mmol), Mn(OTf)$_2$ (14.1 mg, 0.04 mmol), and PhB(OH)$_2$ (48.8 mg, 0.4 mmol) was added a solution of enol **1g** (*E*:*Z* = 5.5:1, 32.4 mg, 0.2 mmol) in MTBE (1.0 mL). The reaction was sealed and stirred at 100 °C for 12 h. Subsequently, the reaction was cooled down to room temperature and the mixture was evaporated and purified via column chromatography on silica gel (eluent: petroleum ether) afforded **3** (11.1 mg, 25%): colorless oil.

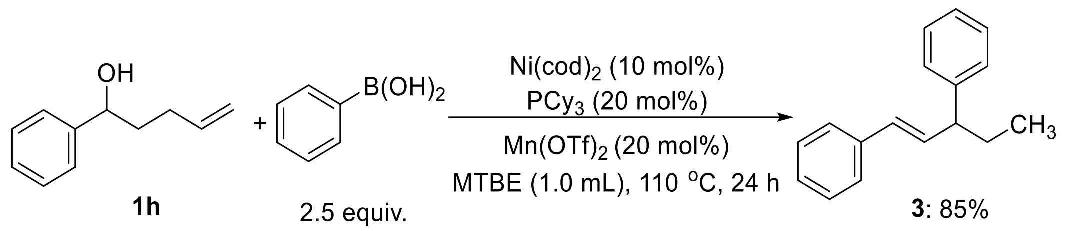

Under a nitrogen atmosphere, to a mixture of $Ni(cod)_2$ (8.3 mg, 0.03 mmol), PCy_3 (16.8 mg, 0.06 mmol), $Mn(OTf)_2$ (21.2 mg, 0.06 mmol), and $PhB(OH)_2$ (91.4 mg, 0.75 mmol) was added a solution of enol **1h** (48.7 mg, 0.3 mmol) in MTBE (1.0 mL). The reaction was sealed and stirred at 110 °C for 24 h. Subsequently, the reaction was cooled down to room temperature and the mixture was evaporated and purified via column chromatography on silica gel (eluent: petroleum ether) afforded **3** (56.6 mg, 85%): colorless oil.

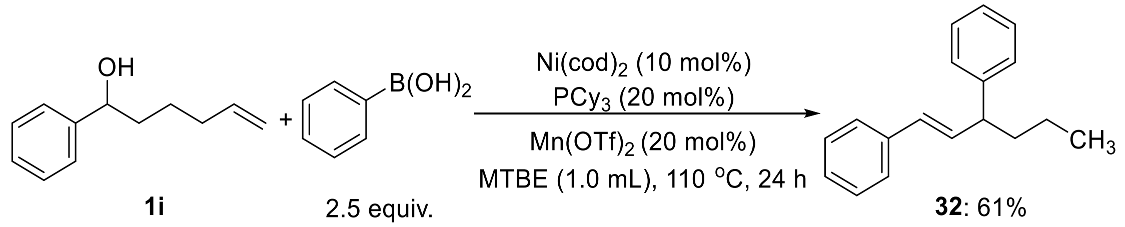

Under a nitrogen atmosphere, to a mixture of $Ni(cod)_2$ (8.3 mg, 0.03 mmol), PCy_3 (16.8 mg, 0.06 mmol), $Mn(OTf)_2$ (21.2 mg, 0.06 mmol), and $PhB(OH)_2$ (91.4 mg, 0.75 mmol) was added a solution of enol **1i** (52.9 mg, 0.3 mmol) in MTBE (1.0 mL). The reaction was sealed and stirred at 110 °C for 24 h. Subsequently, the reaction was cooled down to room temperature and the mixture was evaporated and purified via column chromatography on silica gel (eluent: petroleum ether) afforded **32** (43.2 mg, 61%): colorless oil.

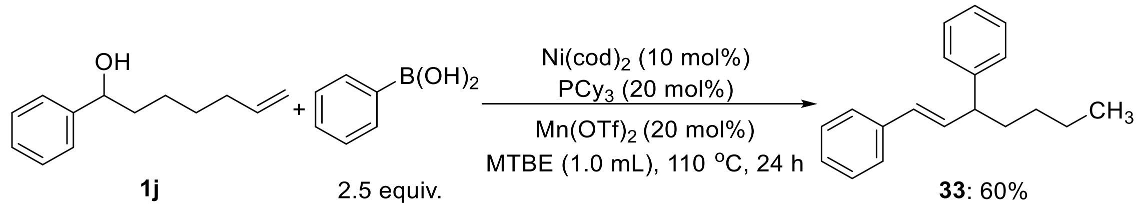

Under a nitrogen atmosphere, to a mixture of $Ni(cod)_2$ (8.3 mg, 0.03 mmol), PCy_3

(16.8 mg, 0.06 mmol), $Mn(OTf)_2$ (21.2 mg, 0.06 mmol), and $PhB(OH)_2$ (91.4 mg, 0.75 mmol) was added a solution of enol **1j** (57.1 mg, 0.3 mmol) in MTBE (1.0 mL). The reaction was sealed and stirred at 110 °C for 24 h. Subsequently, the reaction was cooled down to room temperature and the mixture was evaporated and purified via column chromatography on silica gel (eluent: petroleum ether) afforded **33** (44.5 mg, 60%): colorless oil.

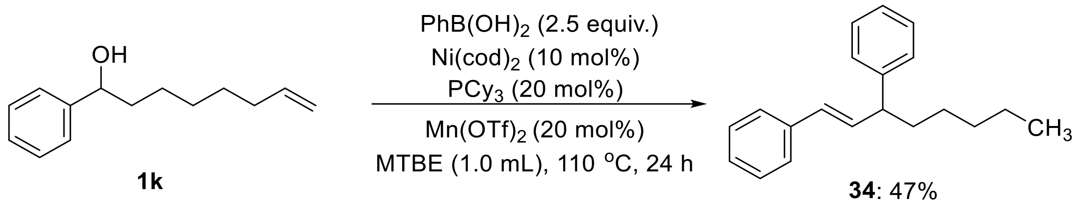

Under a nitrogen atmosphere, to a mixture of $Ni(cod)_2$ (8.3 mg, 0.03 mmol), PCy_3 (16.8 mg, 0.06 mmol), $Mn(OTf)_2$ (21.2 mg, 0.06 mmol), and $PhB(OH)_2$ (91.4 mg, 0.75 mmol) was added a solution of enol **1k** (61.3 mg, 0.3 mmol) in MTBE (1.0 mL). The reaction was sealed and stirred at 110 °C for 24 h. Subsequently, the reaction was cooled down to room temperature and the mixture was evaporated and purified via column chromatography on silica gel (eluent: petroleum ether) afforded **34** (37.2 mg, 47%): colorless oil.

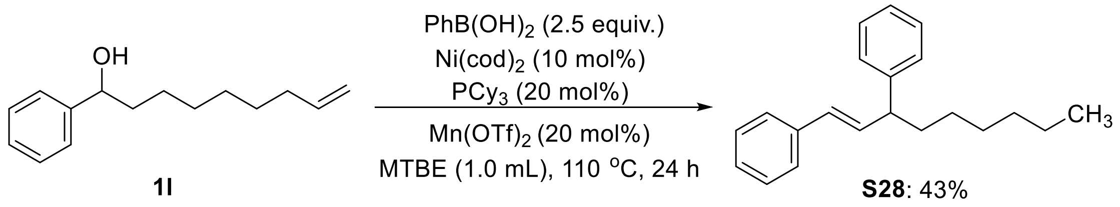

Under a nitrogen atmosphere, to a mixture of $Ni(cod)_2$ (8.3 mg, 0.03 mmol), PCy_3 (16.8 mg, 0.06 mmol), $Mn(OTf)_2$ (21.2 mg, 0.06 mmol), and $PhB(OH)_2$ (91.4 mg, 0.75 mmol) was added a solution of enol **1l** (65.5 mg, 0.3 mmol) in MTBE (1.0 mL). The reaction was sealed and stirred at 110 °C for 24 h. Subsequently, the reaction was cooled down to room temperature and the mixture was evaporated and purified via column chromatography on silica gel (eluent: petroleum ether) afforded **S28** (35.9 mg, 43%):

colorless oil. ^{1}H NMR (400 MHz, Chloroform-*d*) δ 7.35-7.23 (m, 8H), 7.22-7.16 (m, 2H), 6.41-6.29 (m, 2H), 3.39 (q, J = 7.3 Hz, 1H), 1.79 (q, J = 7.4 Hz, 2H), 1.37-1.24 (m, 8H), 0.86 (t, J = 6.7 Hz, 3H); ^{13}C NMR (100 MHz, Chloroform-*d*) δ 144.8, 137.6, 134.5, 129.2, 128.5, 128.4, 127.6, 127.0, 126.1, 126.1, 49.2, 35.9, 31.8, 29.3, 27.6, 22.6, 14.1; HRMS (EI) m/z calculated for $C_{21}H_{26}$ $[M]^+$: 278.2029, found: 278.2030.

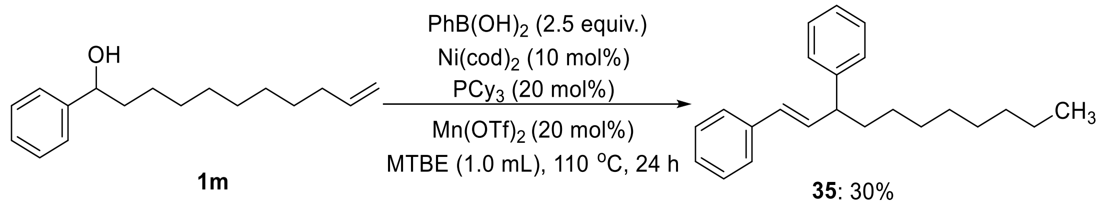

Under a nitrogen atmosphere, to a mixture of Ni(cod)$_2$ (8.3 mg, 0.03 mmol), PCy$_3$ (16.8 mg, 0.06 mmol), Mn(OTf)$_2$ (21.2 mg, 0.06 mmol), and PhB(OH)$_2$ (91.4 mg, 0.75 mmol) was added a solution of enol **1m** (73.9 mg, 0.3 mmol) in MTBE (1.0 mL). The reaction was sealed and stirred at 110 °C for 24 h. Subsequently, the reaction was cooled down to room temperature and the mixture was evaporated and purified via column chromatography on silica gel (eluent: petroleum ether) afforded **35** (27.2 mg, 30%): colorless oil.

c) Reaction of the mixture of isomers (1:1:1:1:1:1)

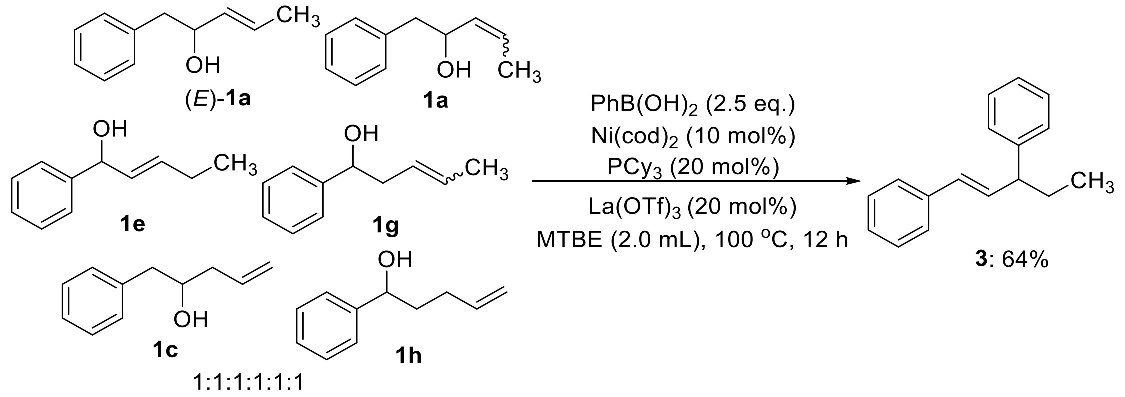

Under a nitrogen atmosphere, to a mixture of Ni(cod)$_2$ (16.5 mg, 0.06 mmol), PCy$_3$ (33.7 mg, 0.12 mmol), La(OTf)$_3$ (70.3 mg, 0.12 mmol), and PhB(OH)$_2$ (182.9 mg, 1.5 mmol) was added a solution of enol (*E*)-**1a** (16.2 mg, 0.1 mmol), **1a** (*Z*:*E* = 3:1, 16.2

mg, 0.1 mmol), **1c** (16.2 mg, 0.1 mmol), **1e** (16.2 mg, 0.1 mmol), **1g** (*E*:*Z* = 5.5:1, 16.2 mg, 0.1 mmol), **1h** (16.2 mg, 0.1 mmol) in MTBE (2.0 mL). The reaction was sealed and stirred at 100 °C for 12 h. Subsequently, the reaction was cooled down to room temperature and the mixture was evaporated and purified via column chromatography on silica gel (eluent: petroleum ether) afforded **3** (85.1 mg, 64%): colorless oil.

*d) (E)-(3-ethyl-5-methylhexa-1,4-dien-1-yl)benzene **(3-alkenyl)***

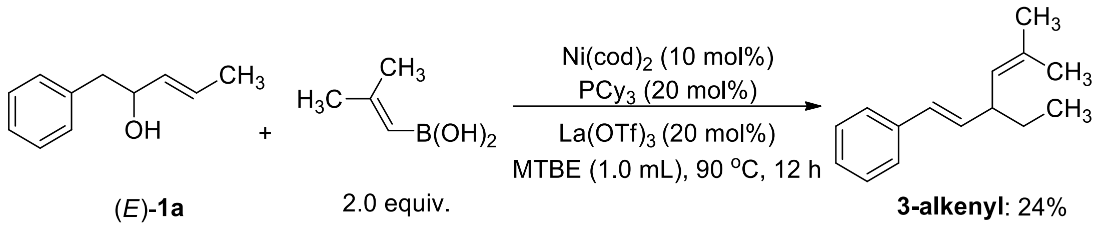

Under a nitrogen atmosphere, to a mixture of Ni(cod)$_2$ (5.5 mg, 0.02 mmol), PCy$_3$ (11.2 mg, 0.04 mmol), La(OTf)$_3$ (23.4 mg, 0.04 mmol), and (2-methylprop-1-en-1-yl)boronic acid (40.0 mg, 0.4 mmol) was added a solution of enol (*E*)-**1a** (32.4 mg, 0.2 mmol) in MTBE (1.0 mL). The reaction was sealed and stirred at 90 °C for 12 h. Subsequently, the reaction was cooled down to room temperature and the mixture was evaporated and purified via column chromatography on silica gel (eluent: petroleum ether) afforded **3-alkenyl** (9.7 mg, 24%): colorless oil; ^{1}H NMR (400 MHz, Chloroform-*d*) δ 7.34 (d, *J* = 6.8 Hz, 2H), 7.29-7.26 (m, 2H), 7.17 (t, *J* = 7.2 Hz, 1H), 6.33 (d, *J* = 15.9 Hz, 1H), 6.08 (dd, *J* = 15.9, 7.3 Hz, 1H), 5.03 (d, *J* = 9.0 Hz, 1H), 2.99-2.91 (m, 1H), 1.74 (s, 3H), 1.66 (s, 3H), 1.54-1.36 (m, 2H), 0.89 (t, *J* = 7.4 Hz, 3H); ^{13}C NMR (100 MHz, Chloroform-*d*) δ 138.0, 134.2, 132.0, 128.4, 128.2, 127.1, 126.7, 126.0, 43.5, 28.8, 25.9, 18.2, 11.8; HRMS (EI) m/z calculated for $C_{15}H_{20}$ $[M]^+$: 200.1560, found: 200.1562.

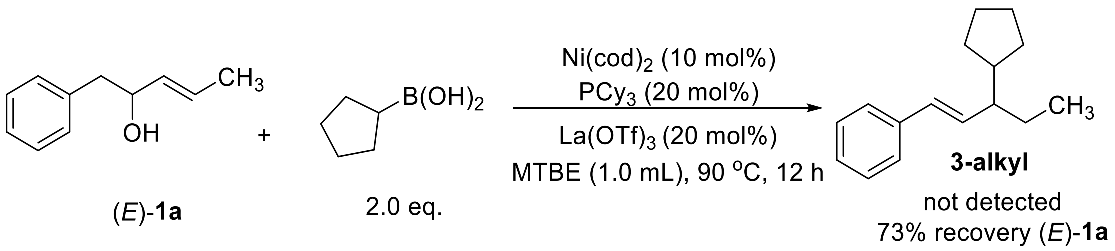

Under a nitrogen atmosphere, to a mixture of Ni(cod)$_2$ (5.5 mg, 0.02 mmol), PCy$_3$ (11.2 mg, 0.04 mmol), La(OTf)$_3$ (23.4 mg, 0.04 mmol), and cyclopentylboronic acid (45.6 mg, 0.4 mmol) was added a solution of enol (*E*)-**1a** (32.4 mg, 0.2 mmol) in MTBE (1.0 mL). The reaction was sealed and stirred at 90 °C for 12 h. Subsequently, the reaction was cooled down to room temperature and then concentrated under reduced pressure. **3-alkyl** was not detected from the reaction as confirmed by ^{1}H NMR. The recovery of (*E*)-**1a** was determined by ^{1}H NMR analysis using dibromomethane as the internal standard.

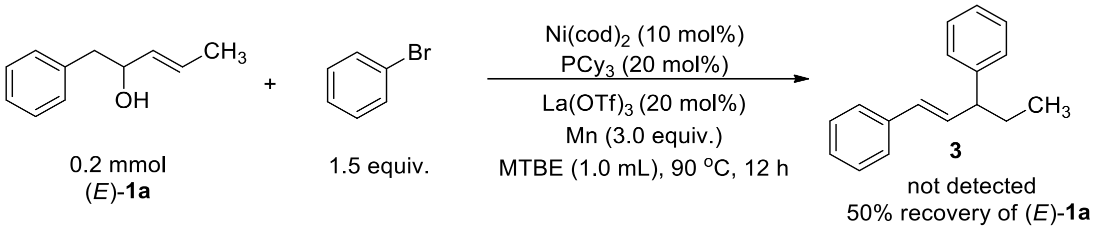

Under a nitrogen atmosphere, to a mixture of Ni(cod)$_2$ (5.5 mg, 0.02 mmol), PCy$_3$ (11.2 mg, 0.04 mmol), La(OTf)$_3$ (23.4 mg, 0.04 mmol), Mn (33.0 mg, 0.6 mmol) and bromobenzene (47.1 mg, 0.3 mmol) was added a solution of enol (*E*)-**1a** (32.4 mg, 0.2 mmol) in MTBE (1.0 mL). The reaction was sealed and stirred at 90 °C for 12 h. Subsequently, the reaction was cooled down to room temperature and then concentrated under reduced pressure. Desired product **3** was not detected from the reaction as confirmed by ^{1}H NMR. The recovery of (*E*)-**1a** was determined by ^{1}H NMR analysis using dibromomethane as the internal standard.

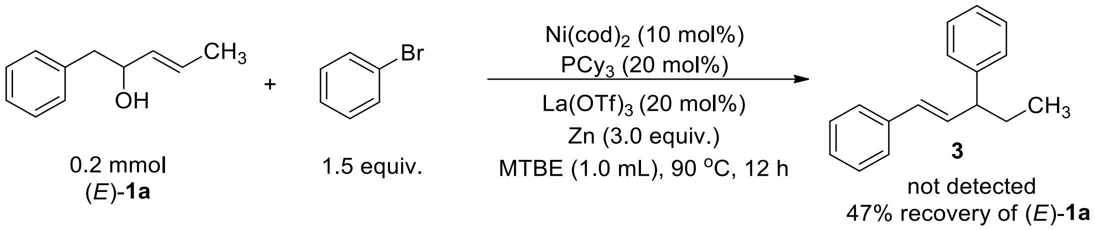

Under a nitrogen atmosphere, to a mixture of Ni(cod)$_2$ (5.5 mg, 0.02 mmol), PCy$_3$ (11.2 mg, 0.04 mmol), La(OTf)$_3$ (23.4 mg, 0.04 mmol), Zn (39.2 mg, 0.6 mmol) and bromobenzene (47.1 mg, 0.3 mmol) was added a solution of enol (*E*)-**1a** (32.4 mg, 0.2

mmol) in MTBE (1.0 mL). The reaction was sealed and stirred at 90 °C for 12 h. Subsequently, the reaction was cooled down to room temperature and then concentrated under reduced pressure. Desired product **3** was not observed from the reaction as confirmed by ^{1}H NMR. The recovery of (*E*)-**1a** was determined by ^{1}H NMR analysis using dibromomethane as the internal standard.

e) Preliminary attempts for asymmetric migratory allylic arylation

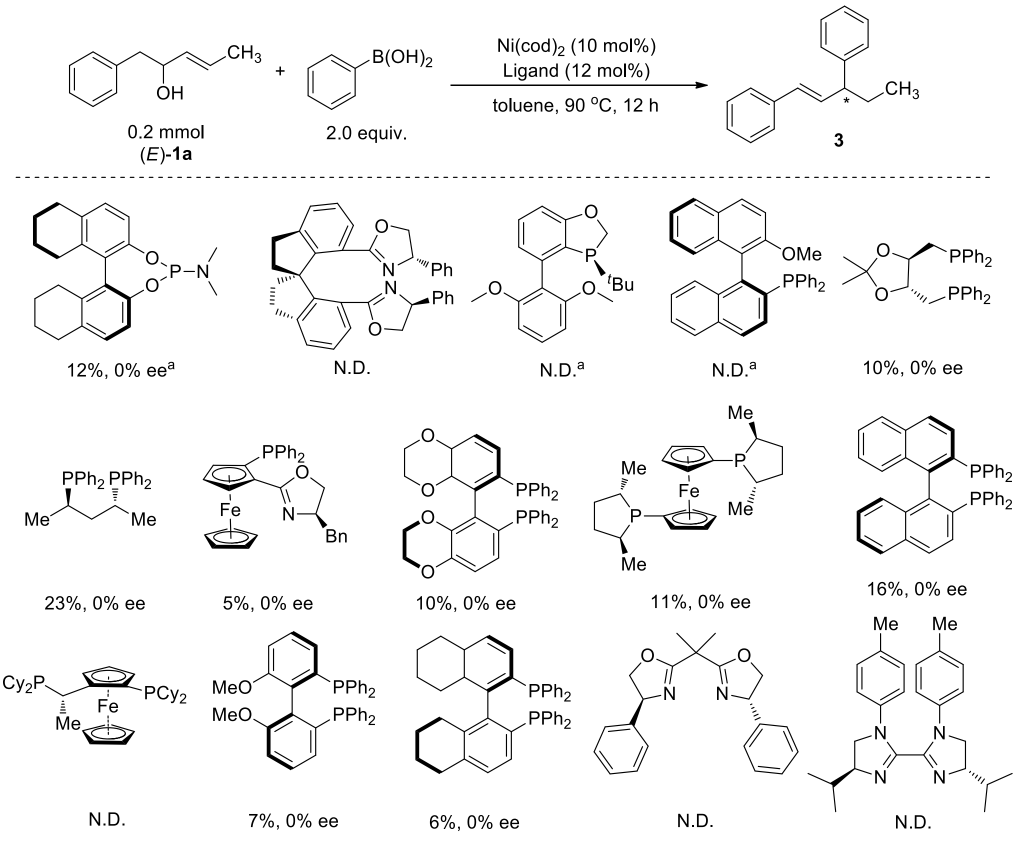

Reaction conditions: (*E*)-**1a** (0.2 mmol), $PhB(OH)_2$ (0.4 mmol), $Ni(cod)_2$ (10 mol %), ligand (12 mol %), toluene (1 mL) at 90 °C for 12 h. The yield was determined by ^{1}H NMR using CH_2Br_2 as an internal standard. Enantiomric excess (*ee*) was determined by chiral high performance liquid chromatography (HPLC). N.D. = not detected. [a]Ligand: 20 mol%.

Supplementary Figure 1. Preliminary attempts for asymmetric migratory allylic arylation of (*E*)-**1a**

Further investigation of asymmetric migratory allylic arylation was conducted with enols **1o** and **1h**. Good enantioselective controls can be achieved with chiral bidentate nitrogen ligands **L1** and **L2** respectively (91:9 er for 31, 82:18 er for 3), but with low yields in both reactions.

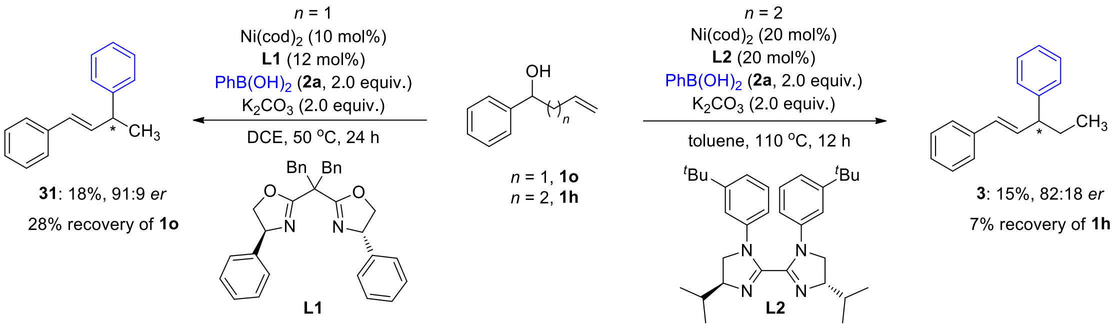

Supplementary Figure 2. Preliminary attempts for asymmetric migratory allylic arylation of **1o** and **1h**

1. *Asymmetric migratory allylic arylation of* ***1o***

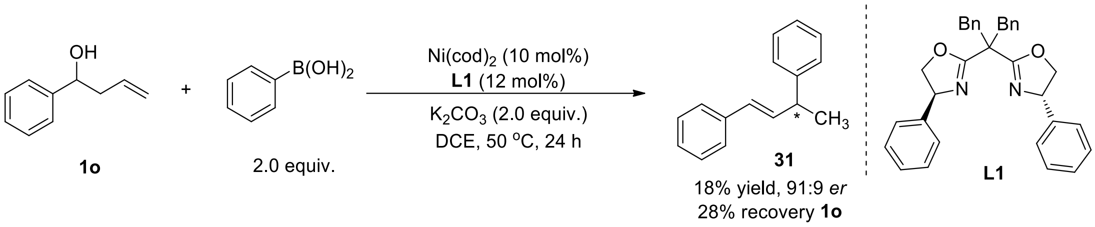

Under a nitrogen atmosphere, to a mixture of Ni(cod)$_2$ (5.5 mg, 0.02 mmol), **L1** (11.7 mg, 0.024 mmol), K_2CO_3 (55.3 mg, 0.4 mmol), and PhB(OH)$_2$ (48.8 mg, 0.4 mmol) was added a solution of enol **1o** (29.6 mg, 0.2 mmol) in DCE (1.0 mL). The reaction was sealed and stirred at 50 °C for 24 h. Subsequently, the reaction was cooled down to room temperature and the mixture was evaporated and purified via column chromatography on silica gel (eluent: petroleum ether) afforded **31** (7.6 mg, 18%, 91:9 *er*): colorless oil; HPLC conditions: CHIRALPAK OJ-H column, 25 °C, wavelength = 250 nm, *n*-Hexane/*i*-PrOH = 99:1, flow rate = 0.5 mL/min, *t* = 34.92 min for major isomer, *t* = 31.73 min for minor isomer.

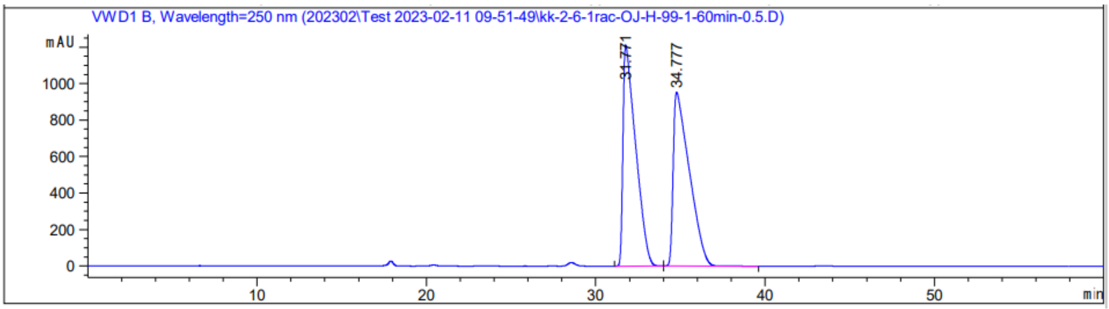

Peak #	RetTime [min]	Type	Width [min]	Area [mAU*s]	Height [mAU]	Area %
1	31.771	BB	0.7521	6.27242e4	1211.26526	49.9620
2	34.777	BB	0.9977	6.28197e4	952.20166	50.0380

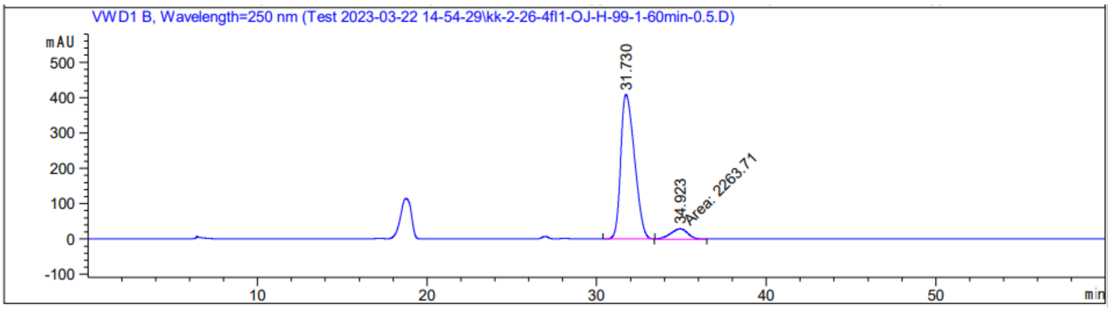

Peak #	RetTime [min]	Type	Width [min]	Area [mAU*s]	Height [mAU]	Area %
1	31.730	BB	0.8968	2.30488e4	409.38373	91.0569
2	34.923	MM	1.2435	2263.70776	30.33951	8.9431

2. *Asymmetric migratory allylic arylation of* ***1h***

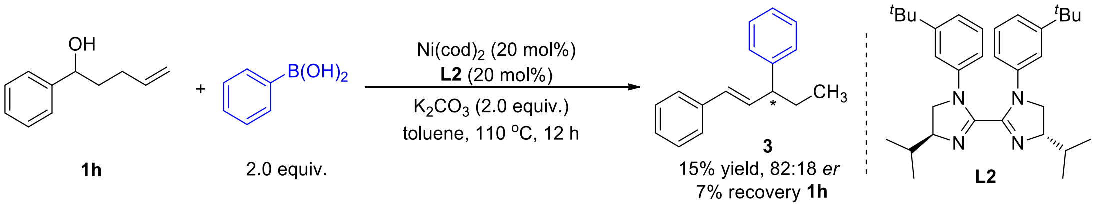

Under a nitrogen atmosphere, to a mixture of Ni(cod)$_2$ (11.0 mg, 0.04 mmol), **L2** (19.5 mg, 0.04 mmol), K_2CO_3 (55.3 mg, 0.4 mmol), and PhB(OH)$_2$ (48.8 mg, 0.4 mmol) was added a solution of enol **1h** (32.4 mg, 0.2 mmol) in toluene (1.5 mL). The reaction was sealed and stirred at 110 °C for 12 h. Subsequently, the reaction was cooled down to room temperature and the mixture was evaporated and purified via column chromatography on silica gel (eluent: petroleum ether) afforded **3** (6.6 mg, 15%, 82:18 *er*): colorless oil; HPLC conditions: CHIRALPAK OJ-H column, 25 °C, wavelength = 250 nm, *n*-Hexane/*i*-PrOH = 99:1, flow rate = 0.5 mL/min, *t* = 35.06 min for major isomer, *t* = 24.13 min for minor isomer.

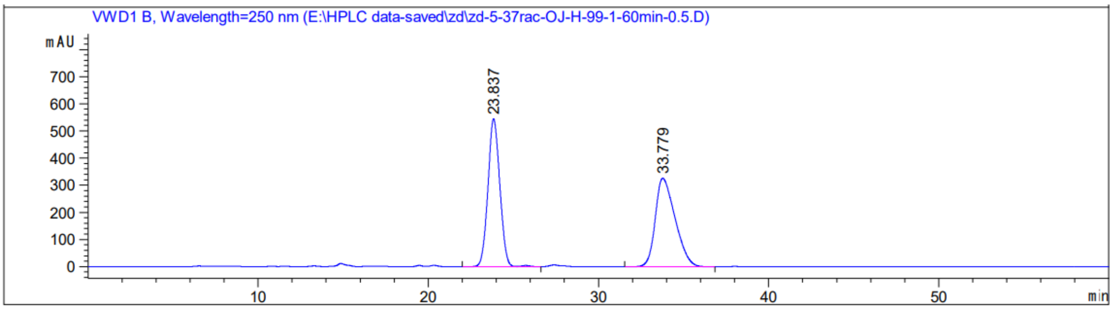

Peak #	RetTime [min]	Type	Width [min]	Area [mAU*s]	Height [mAU]	Area %
1	23.837	BV R	0.7376	2.63889e4	545.32037	50.0910
2	33.779	BB	1.2377	2.62930e4	325.72687	49.9090

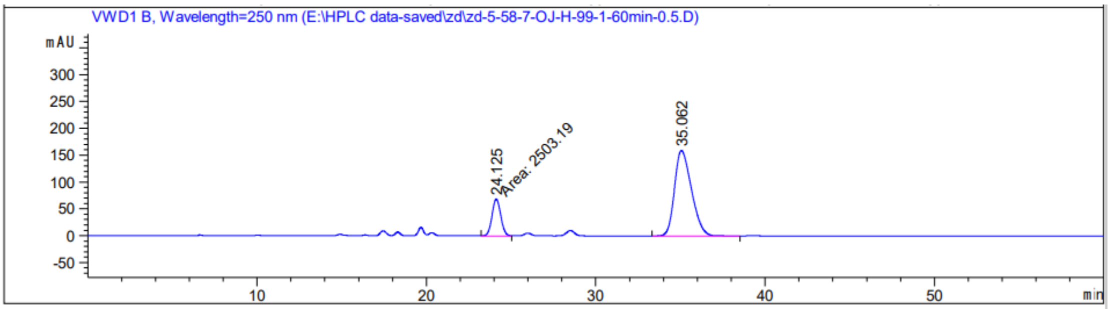

Peak #	RetTime [min]	Type	Width [min]	Area [mAU*s]	Height [mAU]	Area %
1	24.125	MF	0.6035	2503.18872	69.12406	18.4856
2	35.062	BB	1.0731	1.10381e4	159.16289	81.5144

2.4. Mechanistic studies

Control experiments

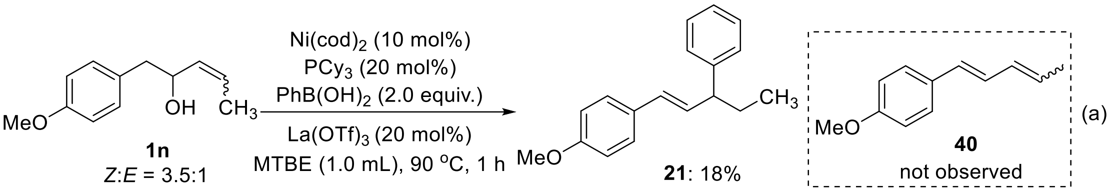

Under a nitrogen atmosphere, to a mixture of Ni(cod)$_2$ (5.5 mg, 0.02 mmol), PCy$_3$ (11.2 mg, 0.04 mmol), La(OTf)$_3$ (23.4 mg, 0.04 mmol), and PhB(OH)$_2$ (48.8 mg, 0.4 mmol) was added a solution of **1n** (*Z*:*E* = 3.5:1, 32.8 mg, 0.2 mmol) in MTBE (1.0 mL). The reaction was sealed and stirred at 90 °C for 1 h. Subsequently, the reaction was cooled down to room temperature and the mixture was evaporated and purified via column chromatography on silica gel (eluent: petroleum ether/ethyl acetate = 200/1) afforded **21** (9.0 mg, 18%): colorless oil.

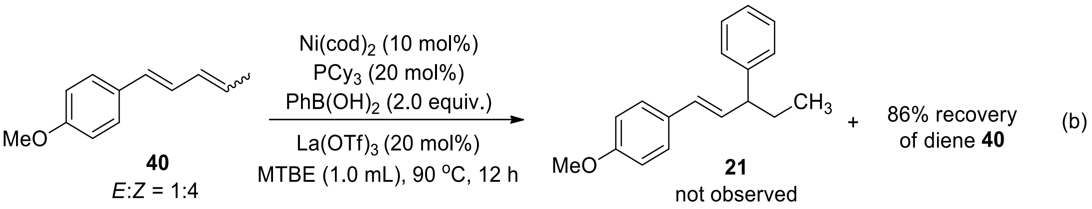

Under a nitrogen atmosphere, to a mixture of Ni(cod)$_2$ (5.5 mg, 0.02 mmol), PCy$_3$ (11.2 mg, 0.04 mmol), La(OTf)$_3$ (23.4 mg, 0.04 mmol), and PhB(OH)$_2$ (48.8 mg, 0.4 mmol) was added a solution of **40** (*E*:*Z* = 4:1, 34.8 mg, 0.2 mmol) in MTBE (1.0 mL). The reaction was sealed and stirred at 90 °C for 12 h. The mixture was cooled down to room temperature and then concentrated under reduced pressure. **21** was not observed from the reaction as confirmed by ^{1}H NMR. The yield of **40** was determined by ^{1}H NMR analysis using dibromomethane as the internal standard.

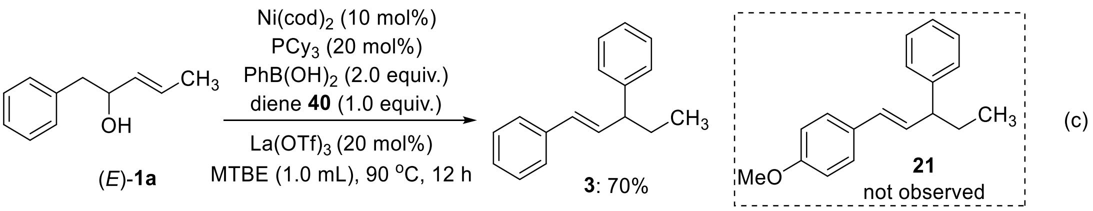

Under a nitrogen atmosphere, to a mixture of Ni(cod)$_2$ (5.5 mg, 0.02 mmol), PCy$_3$ (11.2 mg, 0.04 mmol), La(OTf)$_3$ (23.4 mg, 0.04 mmol), and PhB(OH)$_2$ (48.8 mg, 0.4 mmol) was added a solution of (*E*)-**1a** (32.4 mg, 0.2 mmol) and diene **40** (34.8 mg, 0.2 mmol) in MTBE (1.0 mL). The reaction was sealed and stirred at 90 °C for 12 h. The mixture was cooled down to room temperature and then concentrated under reduced pressure. **21** was not observed from the reaction as confirmed by ^{1}H NMR. The yield of **3** was determined by ^{1}H NMR analysis using dibromomethane as the internal standard.

Deuterium labeling experiments

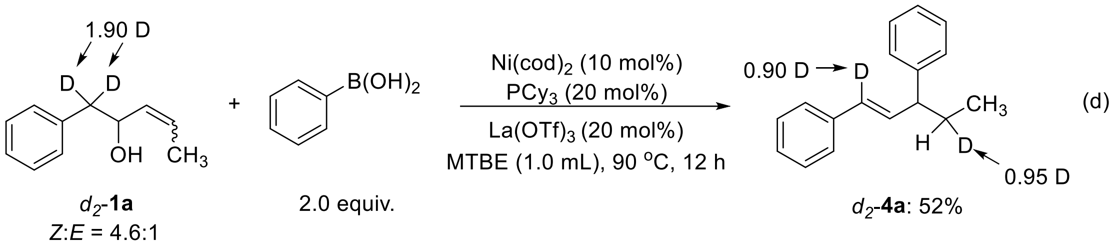

Under a nitrogen atmosphere, to a mixture of Ni(cod)$_2$ (5.5 mg, 0.02 mmol), PCy$_3$ (11.2 mg, 0.04 mmol), La(OTf)$_3$ (23.4 mg, 0.04 mmol), and PhB(OH)$_2$ (48.8 mg, 0.4 mmol) was added a solution of *d*$_2$-**1a** (*Z*:*E* = 4.6:1, 32.8 mg, 0.2 mmol) in MTBE (1.0 mL). The reaction was sealed and stirred at 90 °C for 12 h. Subsequently, the reaction was cooled down to room temperature and the reaction mixture was evaporated and purified via column chromatography on silica gel (eluent: petroleum ether) afforded *d*$_2$-**4a** (23.3 mg, 52%): colorless oil. ^{1}H NMR (400 MHz, Chloroform-*d*) δ 7.35-7.27 (m, 5H), 7.25-7.16 (m, 5H), 6.42-6.30 (m, 1.10H), 3.30 (t, *J* = 7.5 Hz, 1H), 1.87-1.78 (m, 1.05H), 0.90 (d, *J* = 7.2 Hz, 3H); ^{13}C NMR (100 MHz, Chloroform-*d*) δ 144.5, 137.5, 134.1, 129.4, 128.4, 127.7, 127.0, 126.2, 126.1, 50.8, 28.8, 28.6, 28.4, 28.2, 12.2; HRMS (EI) m/z calculated for $C_{17}H_{16}D_2$ [M]$^+$: 224.1529, found: 224.1527. The deuterium incorporation in *d*$_2$-**4a** was determined by ^{1}H NMR analysis.

Intermolecular cross-over reaction

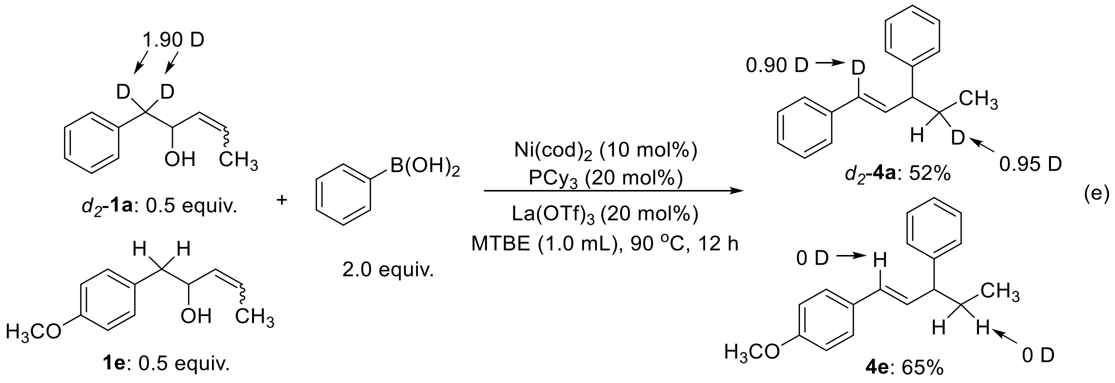

Under a nitrogen atmosphere, to a mixture of $Ni(cod)_2$ (5.5 mg, 0.02 mmol), PCy_3 (11.2 mg, 0.04 mmol), $La(OTf)_3$ (23.4 mg, 0.04 mmol), and $PhB(OH)_2$ (48.8 mg, 0.4 mmol) was added a solution of *d*$_2$-**1a** (*Z*:*E* = 4.6:1, 16.4 mg, 0.1 mmol) and **1e** (*Z*:*E* = 3.5:1, 19.2 mg, 0.1 mmol) in MTBE (1.0 mL). The reaction was sealed and stirred at 90 °C for 12 h. Subsequently, the reaction was cooled down to room temperature and the mixture was evaporated and purified via column chromatography on silica gel (eluent: petroleum ether to petroleum ether/ethyl acetate = 200/1) afforded *d*$_2$-**4a** (11.6 mg, 52%): colorless oil, **4e** (16.4 mg, 65%): colorless oil. No intermolecular H/D exchange during the reaction as confirmed by ^{1}H NMR.

Comparison of reactivity of ***1h*** *and* ***1h-var*** *on the olefin migration*

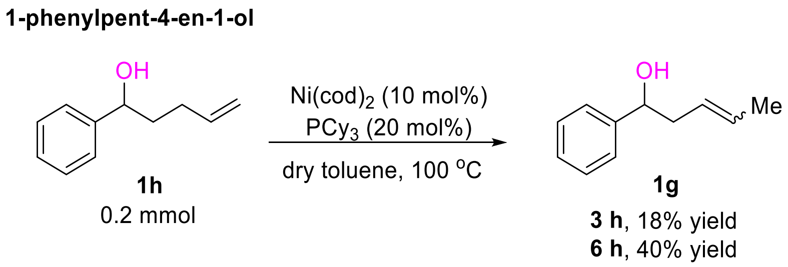

Under a nitrogen atmosphere, to a mixture of $Ni(cod)_2$ (5.5 mg, 0.02 mmol), PCy_3 (11.2 mg, 0.04 mmol) was added a solution of **1h** (32.4 mg, 0.2 mmol) in dry toluene (1.0 mL). The reaction was sealed and stirred at 100 °C for 3 h and 6 h respectively. The mixture was cooled down to room temperature and then concentrated under reduced pressure. The yield of **1g** was determined by ^{1}H NMR analysis using dibromomethane as the internal standard.

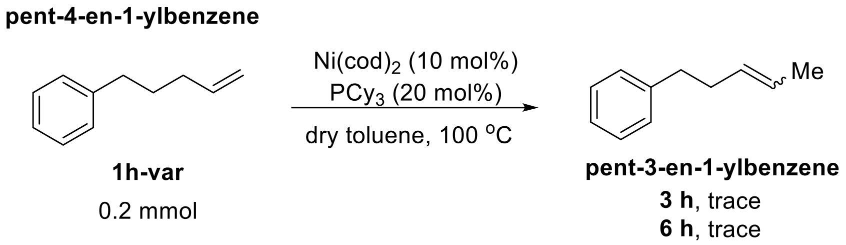

Under a nitrogen atmosphere, to a mixture of Ni(cod)$_2$ (5.5 mg, 0.02 mmol), PCy$_3$ (11.2 mg, 0.04 mmol) was added a solution of **1h-var** (29.2 mg, 0.2 mmol) in dry toluene (1.0 mL). The reaction was sealed and stirred at 100 °C for 3 h and 6 h respectively. The mixture was cooled down to room temperature and then concentrated under reduced pressure. The yield of **pent-3-en-1-ylbenzene** was determined by ^{1}H NMR analysis using dibromomethane as the internal standard.

3. Supplementary Notes

3.1. Computational Details

In order to get deeper insight into the 1,4-hydride transfer process of two stereoisomers of **1a**, density functional theory (DFT) calculations were carried out using the Gaussian 09 software package, revision D.01.[19,20] The stationary structures were optimized using PBE0 method and combined basis set. That is, LANL2DZ for Ni atom, and 6-31G(d) basis set for all the other atoms.[21] Truhlar and coworkers' SMD solvation model was employed to consider the solvent effect of tert-Butyl methyl ether.[22] The geometry optimizations were performed without symmetry constraints, and the nature of the extrema was checked by analytical frequency calculations. The intrinsic reaction coordinate (IRC)[23] pathways have been traced to verify two desired minima connected by the transition states.

3.2. Supplementary Figures

^{1}H NMR and ^{13}C NMR spectra for compounds

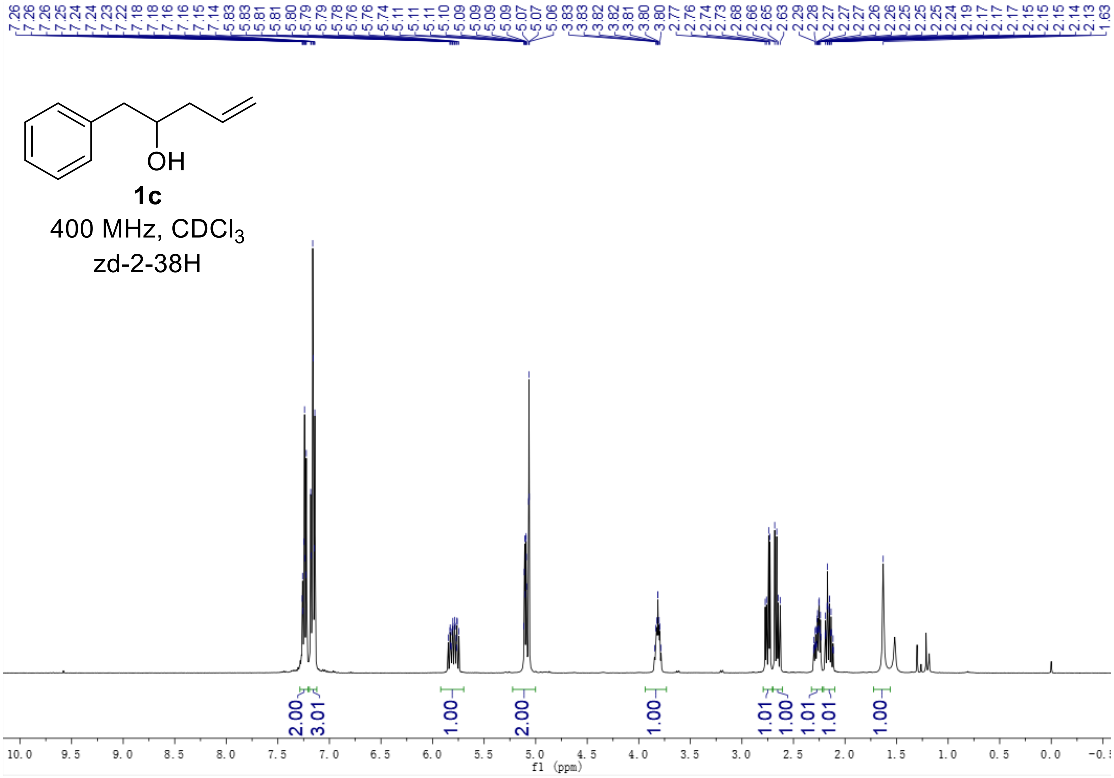

Supplementary Figure 3. ^{1}H NMR (400 MHz, $CDCl_3$, 25 °C) spectra for **1c**

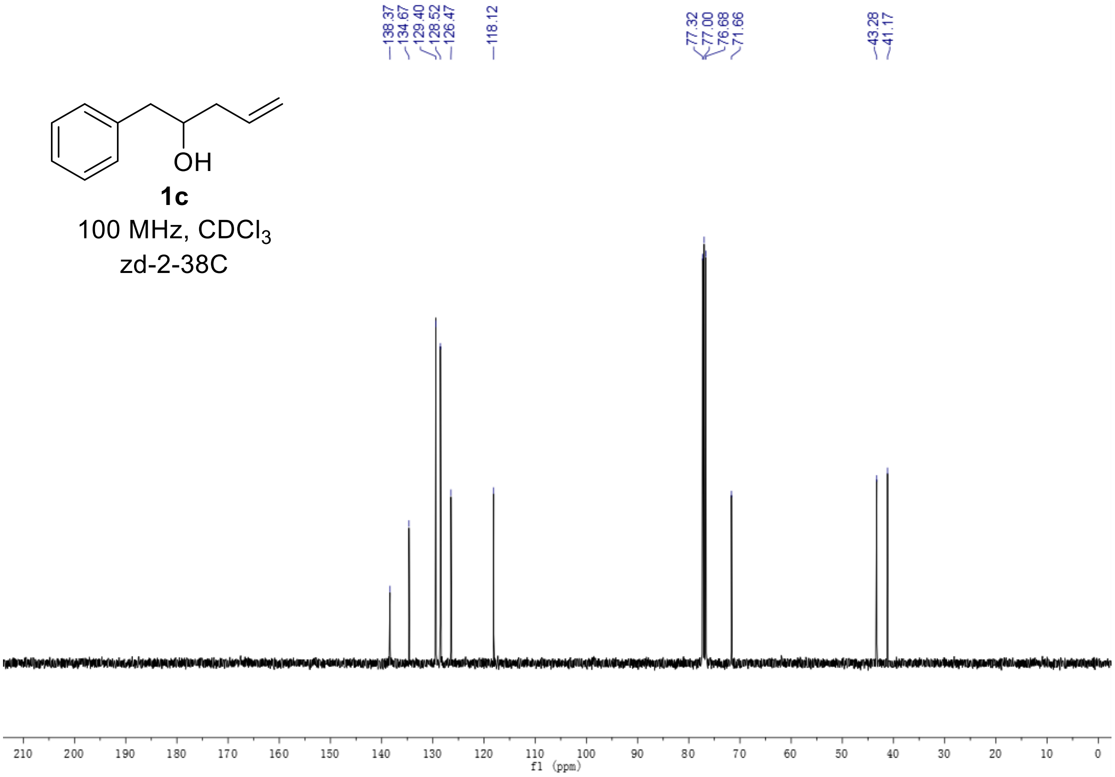

Supplementary Figure 4. ^{13}C NMR (100 MHz, $CDCl_3$, 25 °C) spectra for **1c**

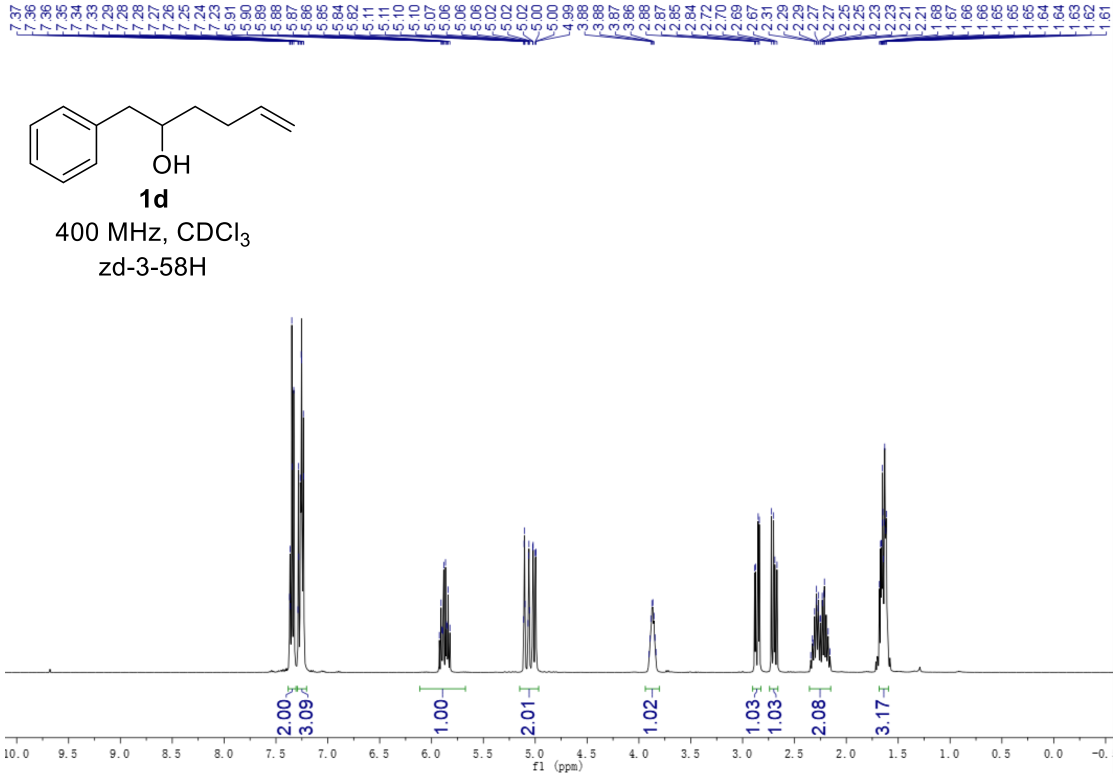

Supplementary Figure 5. ^{1}H NMR (400 MHz, $CDCl_3$, 25 °C) spectra for **1d**

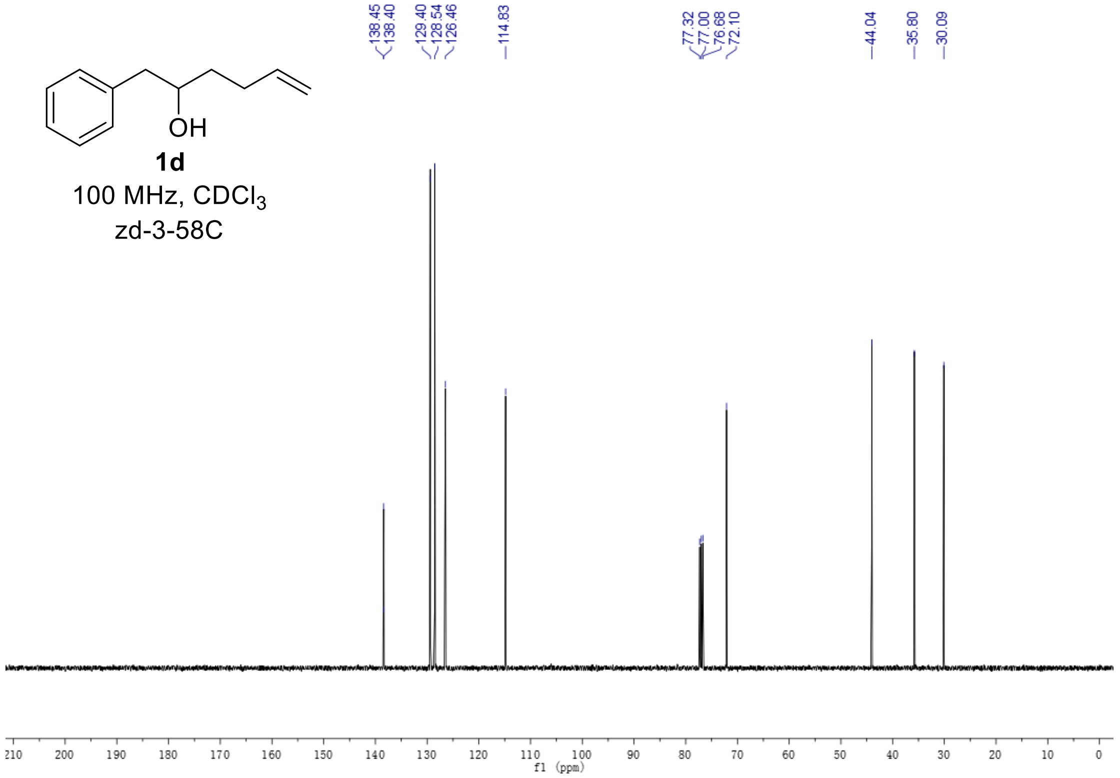

Supplementary Figure 6. ^{13}C NMR (100 MHz, $CDCl_3$, 25 °C) spectra for **1d**

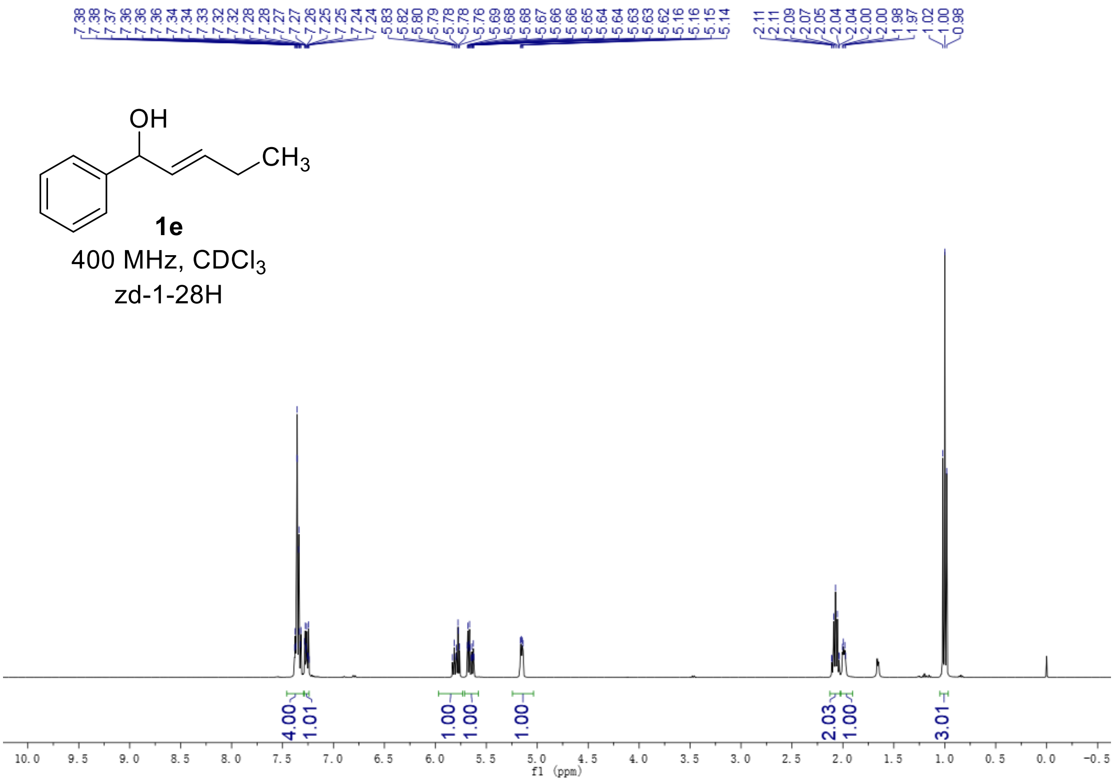

Supplementary Figure 7. ^{1}H NMR (400 MHz, $CDCl_3$, 25 °C) spectra for **1e**

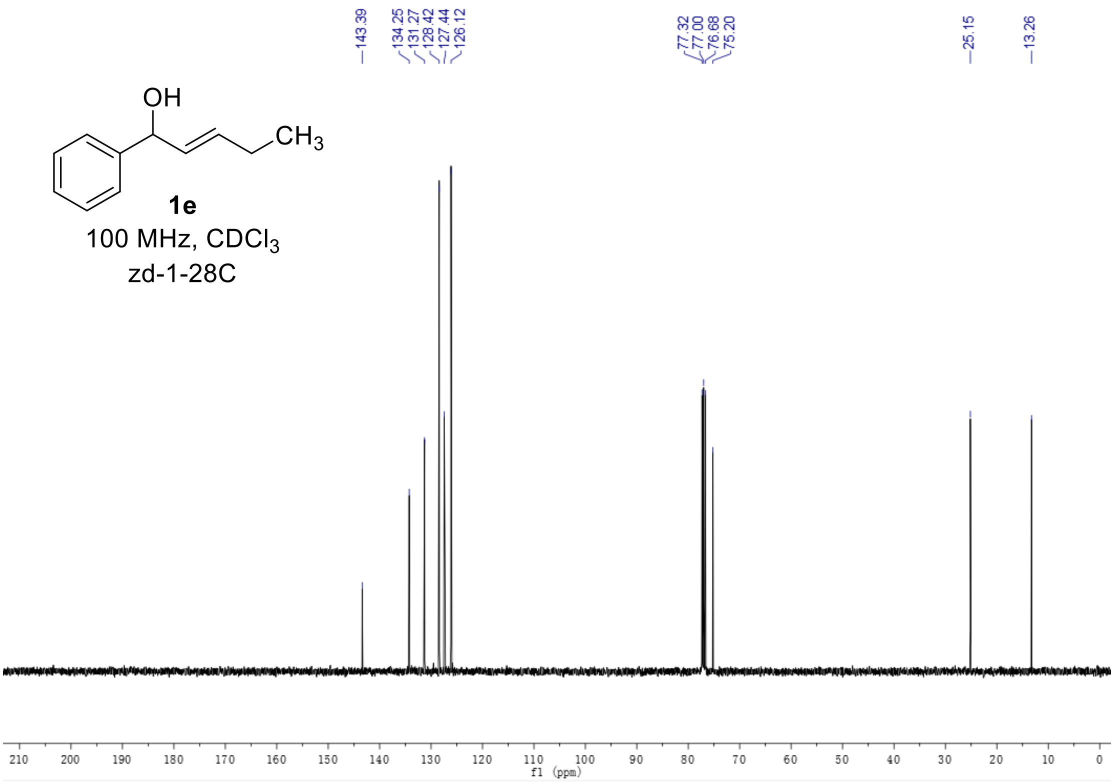

Supplementary Figure 8. ^{13}C NMR (100 MHz, $CDCl_3$, 25 °C) spectra for **1e**

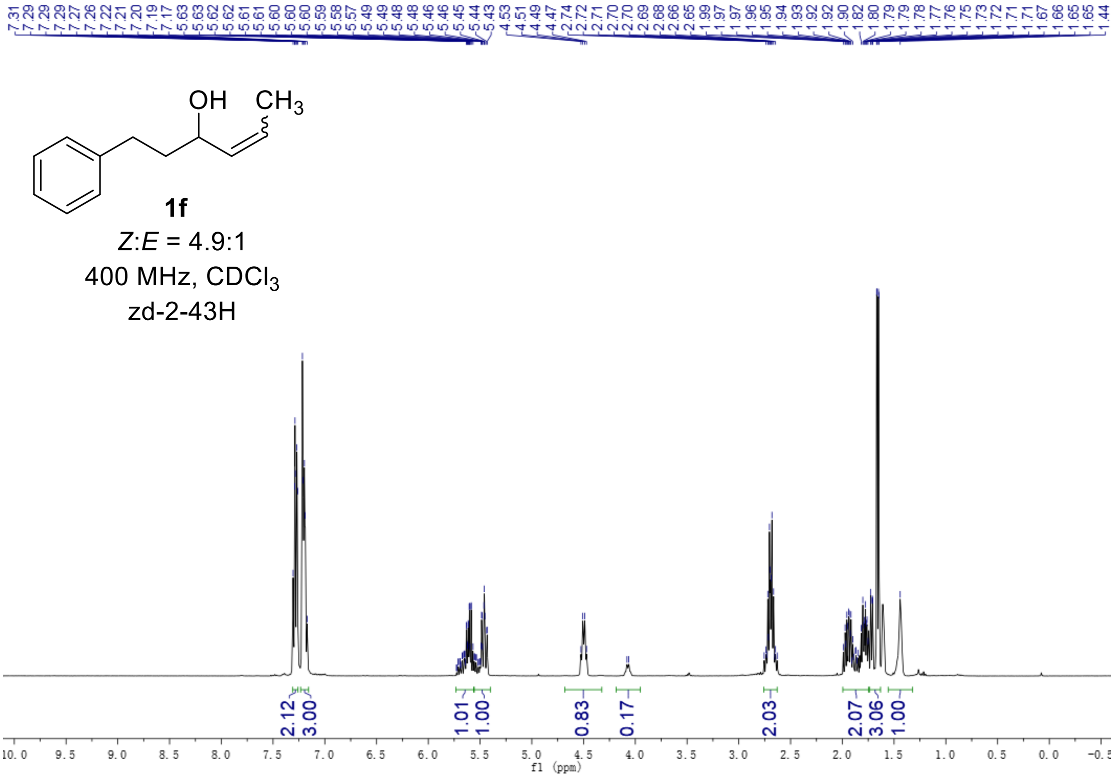

Supplementary Figure 9. ^{1}H NMR (400 MHz, $CDCl_3$, 25 °C) spectra for **1f**

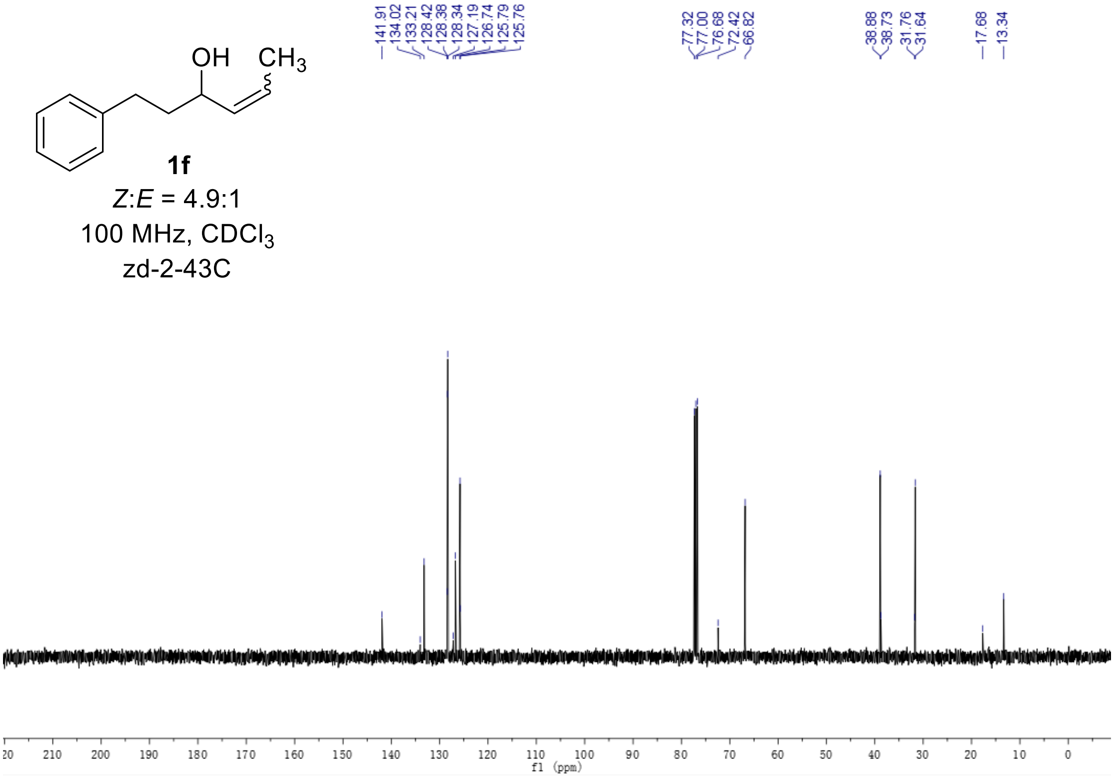

Supplementary Figure 10. ^{13}C NMR (100 MHz, $CDCl_3$, 25 °C) spectra for **1f**

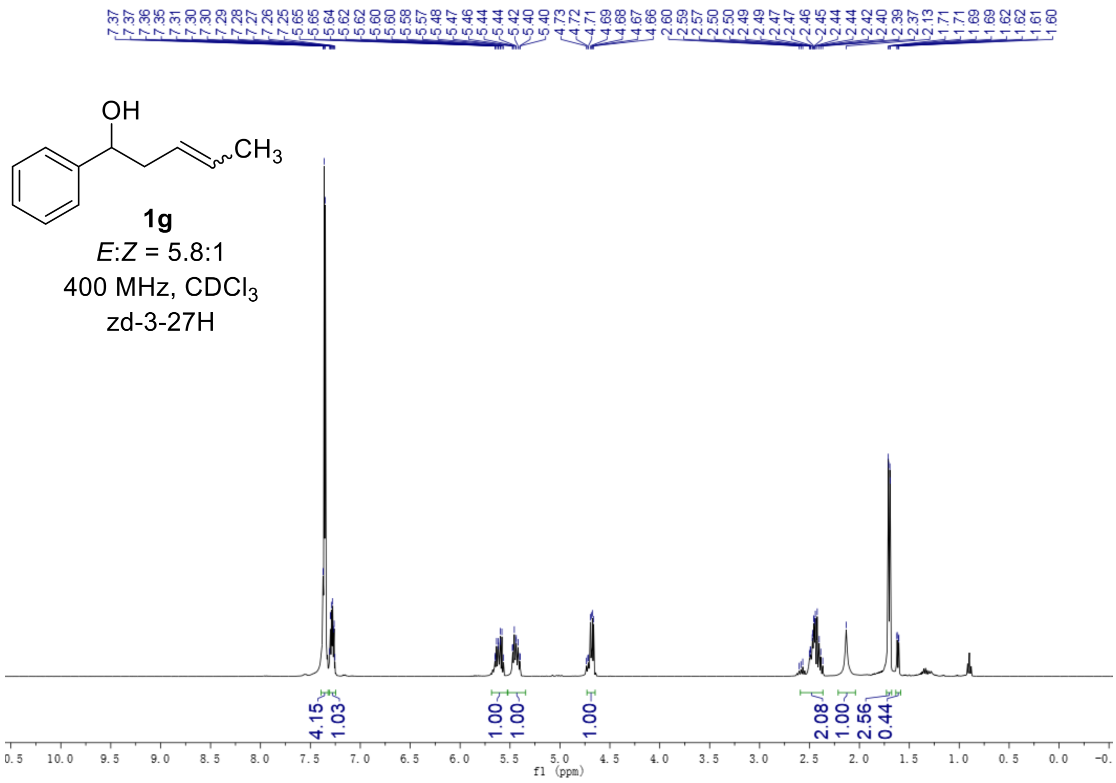

Supplementary Figure 11. ^{1}H NMR (400 MHz, $CDCl_3$, 25 °C) spectra for **1g**

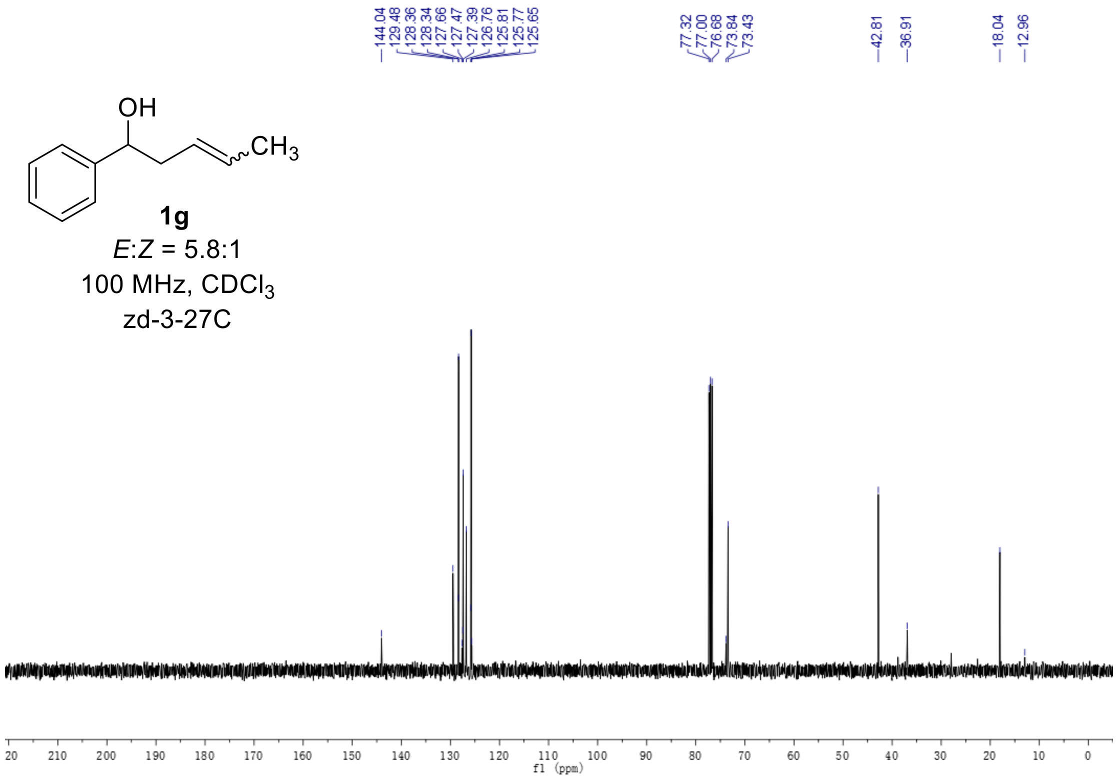

Supplementary Figure 12. ^{13}C NMR (100 MHz, $CDCl_3$, 25 °C) spectra for **1g**

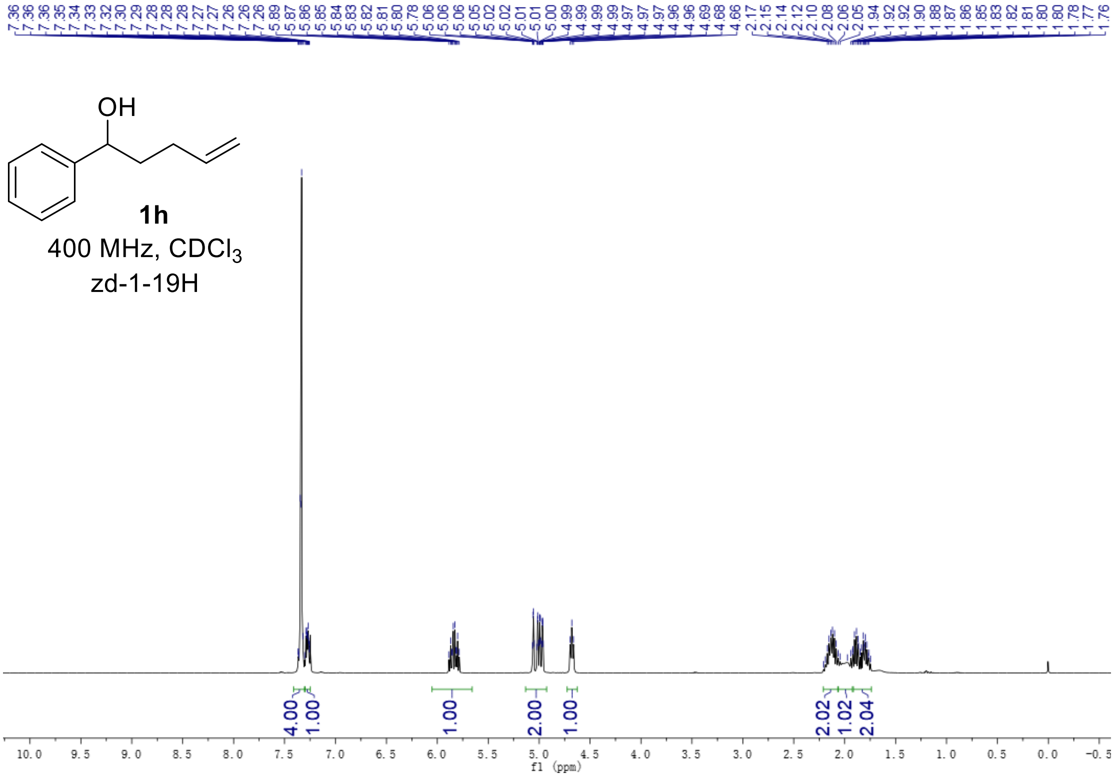

Supplementary Figure 13. ^{1}H NMR (400 MHz, $CDCl_3$, 25 °C) spectra for **1h**

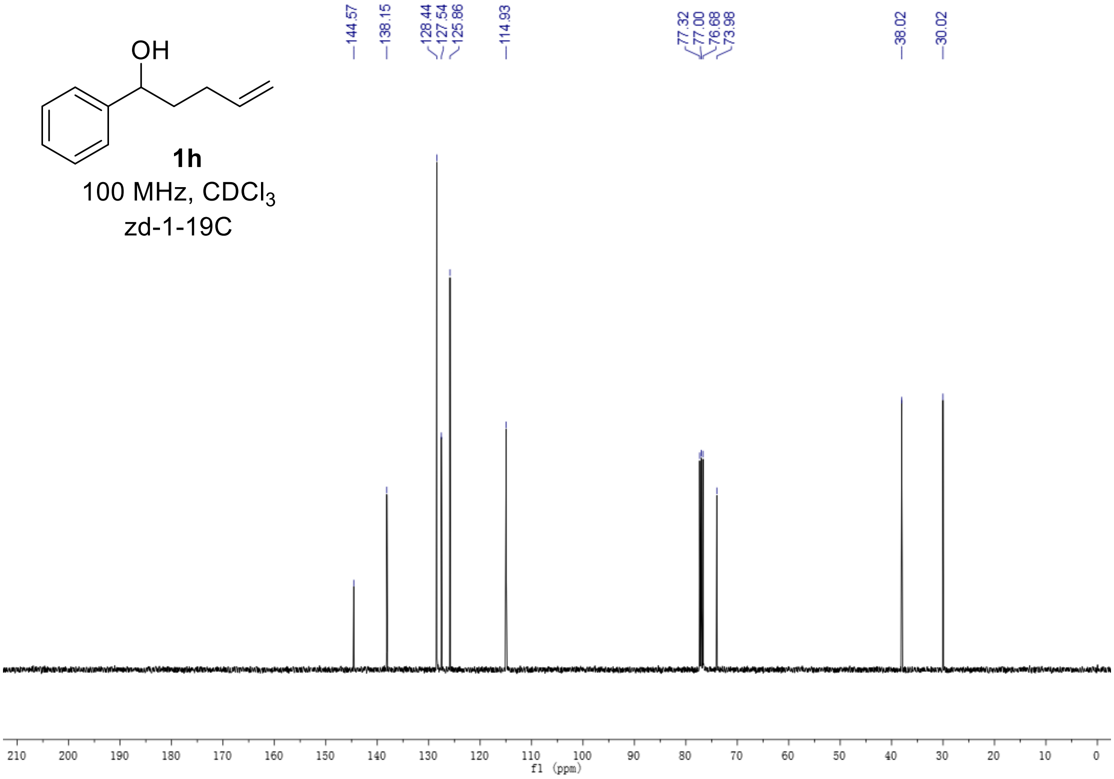

Supplementary Figure 14. ^{13}C NMR (100 MHz, $CDCl_3$, 25 °C) spectra for **1h**

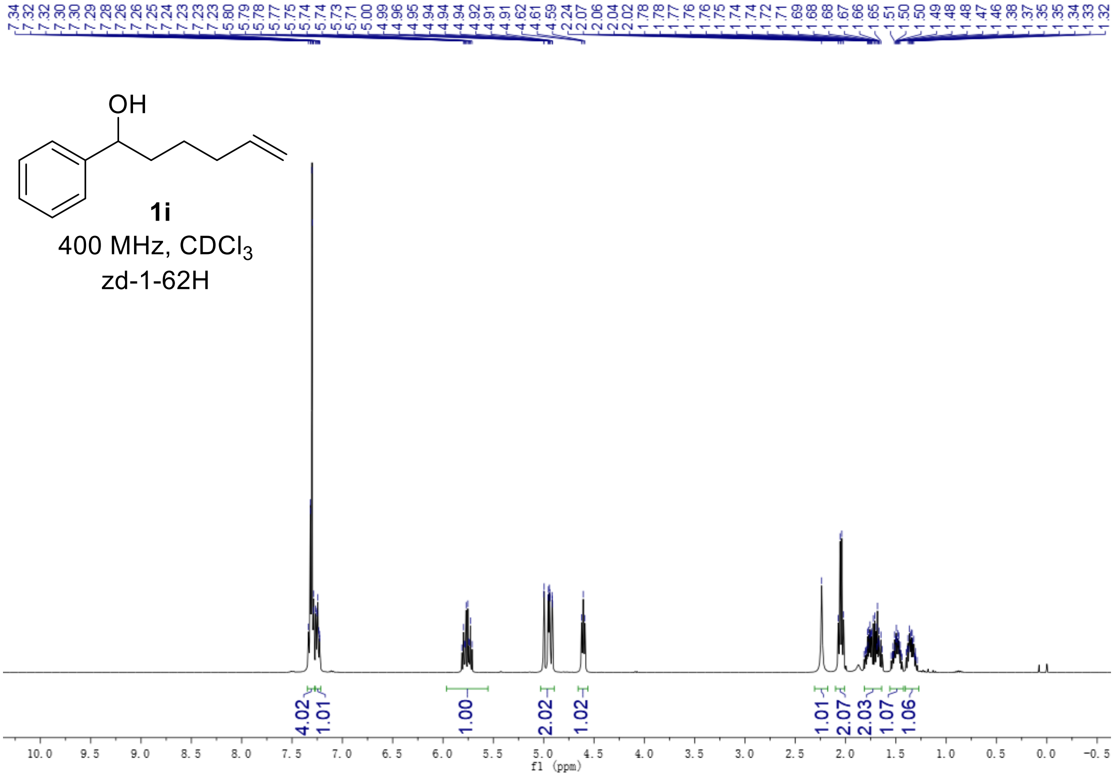

Supplementary Figure 15. ^{1}H NMR (400 MHz, $CDCl_3$, 25 °C) spectra for **1i**

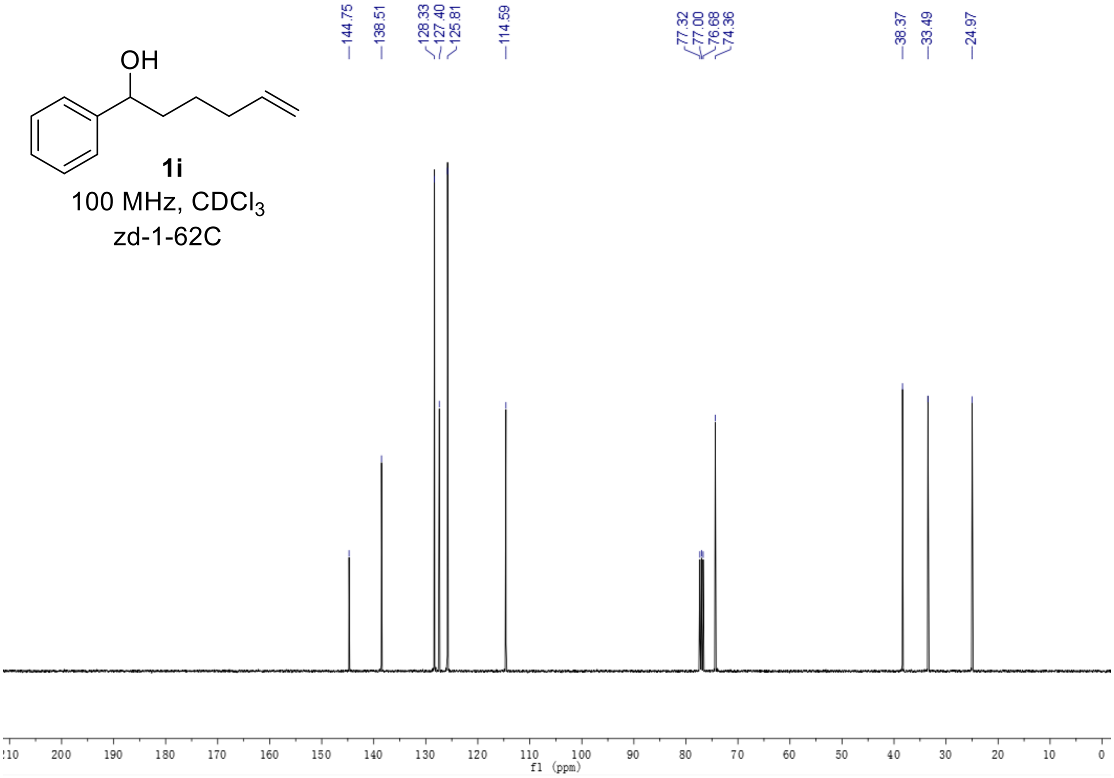

Supplementary Figure 16. ^{13}C NMR (100 MHz, $CDCl_3$, 25 °C) spectra for **1i**

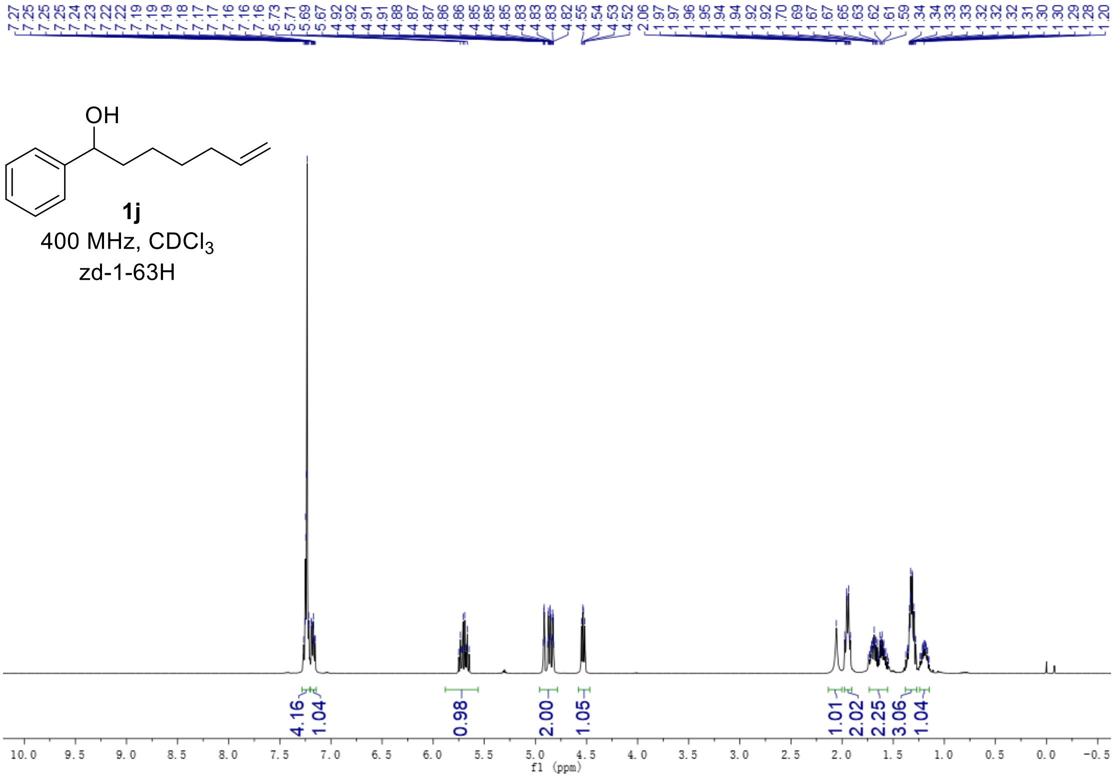

Supplementary Figure 17. ^{1}H NMR (400 MHz, $CDCl_3$, 25 °C) spectra for **1j**

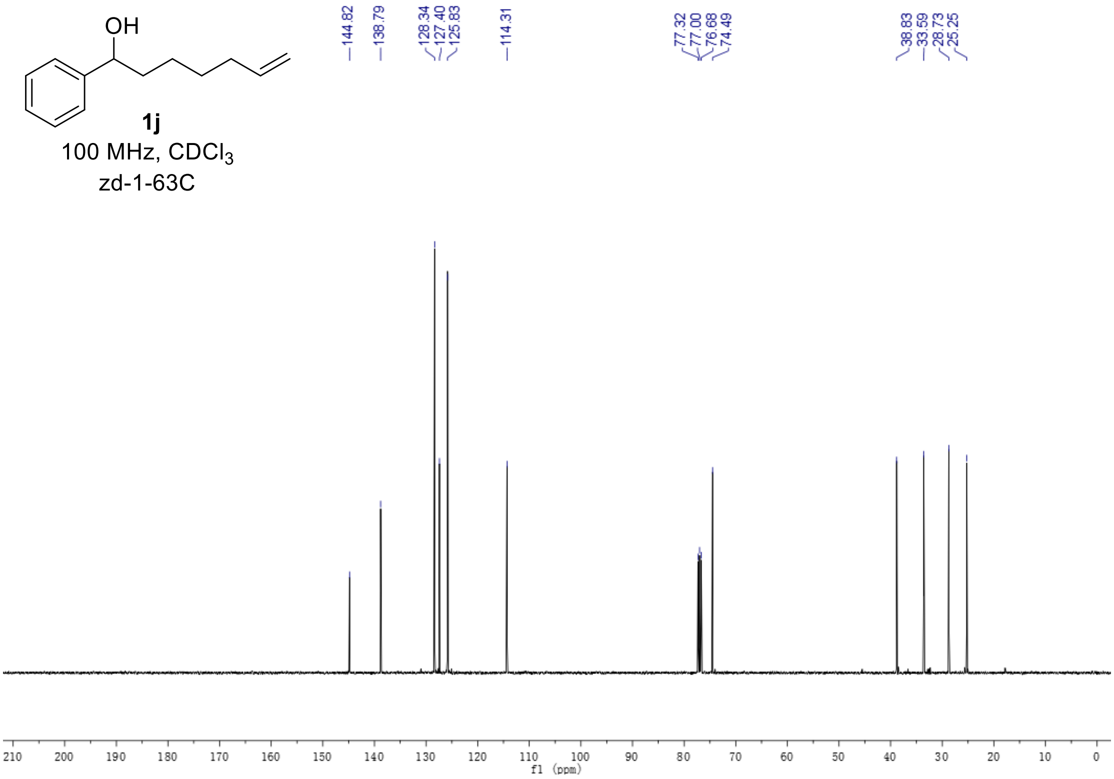

Supplementary Figure 18. ^{13}C NMR (100 MHz, $CDCl_3$, 25 °C) spectra for **1j**

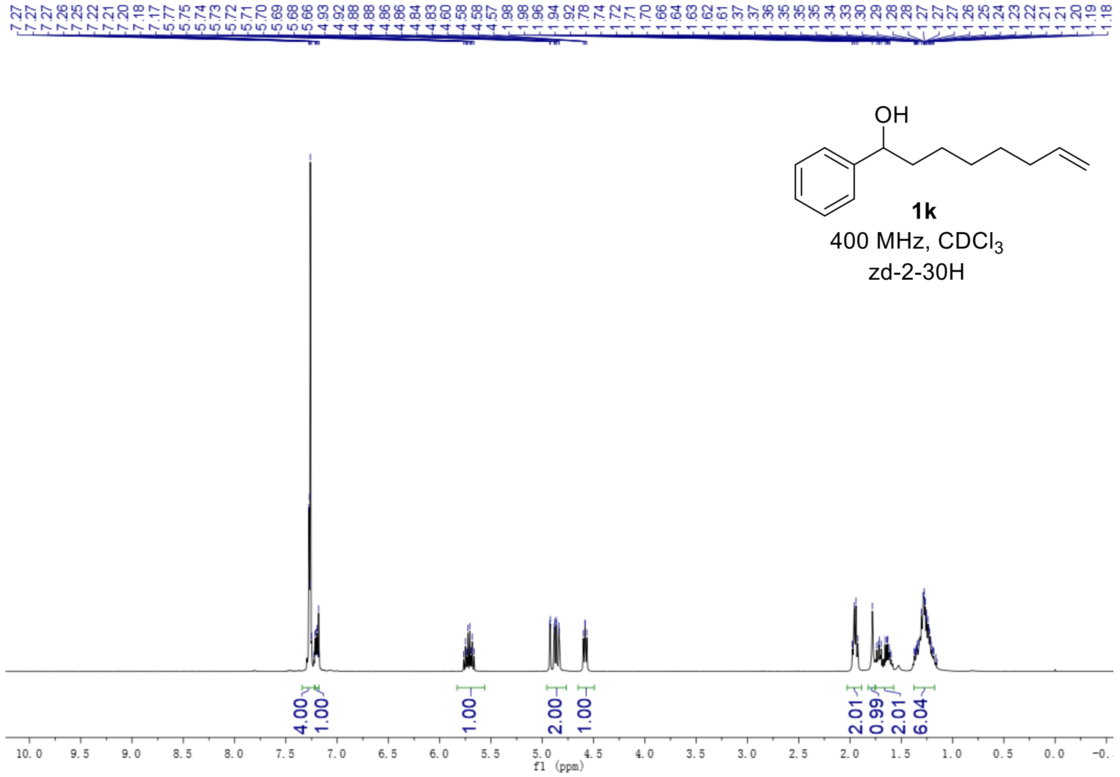

Supplementary Figure 19. ^{1}H NMR (400 MHz, $CDCl_3$, 25 °C) spectra for **1k**

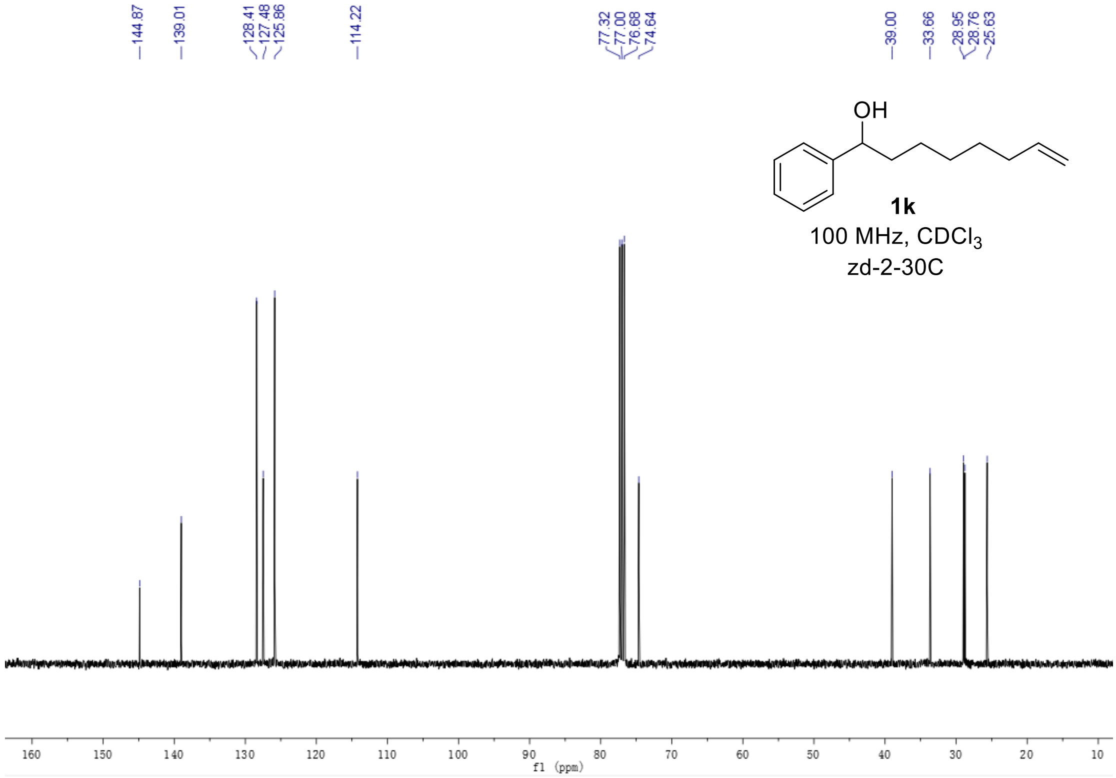

Supplementary Figure 20. ^{13}C NMR (100 MHz, $CDCl_3$, 25 °C) spectra for **1k**

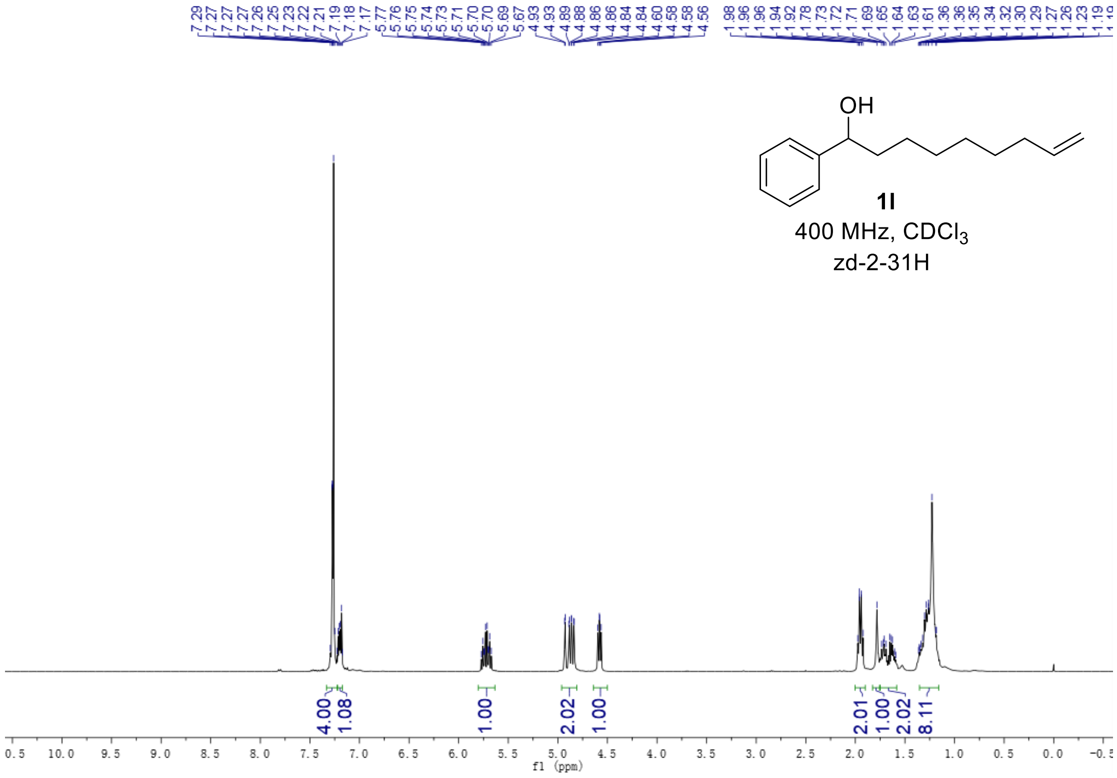

Supplementary Figure 21. ^{1}H NMR (400 MHz, $CDCl_3$, 25 °C) spectra for **1l**

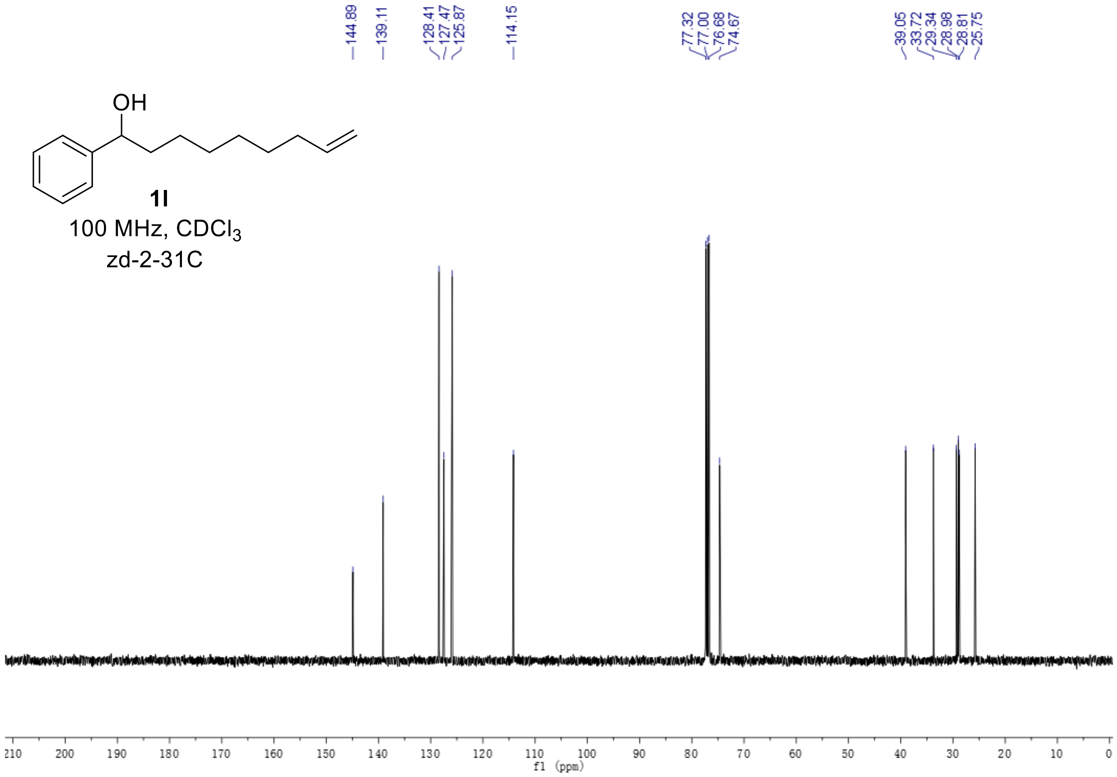

Supplementary Figure 22. ^{13}C NMR (100 MHz, $CDCl_3$, 25 °C) spectra for **1l**

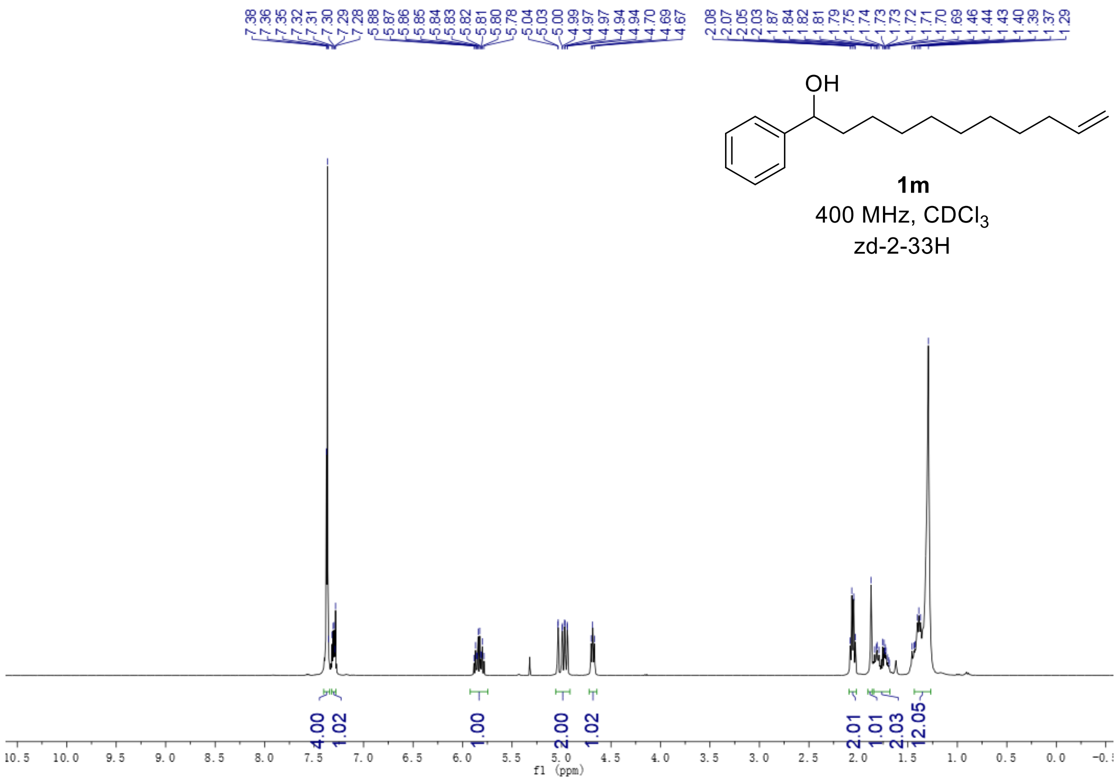

Supplementary Figure 23. ^{1}H NMR (400 MHz, $CDCl_3$, 25 °C) spectra for **1m**

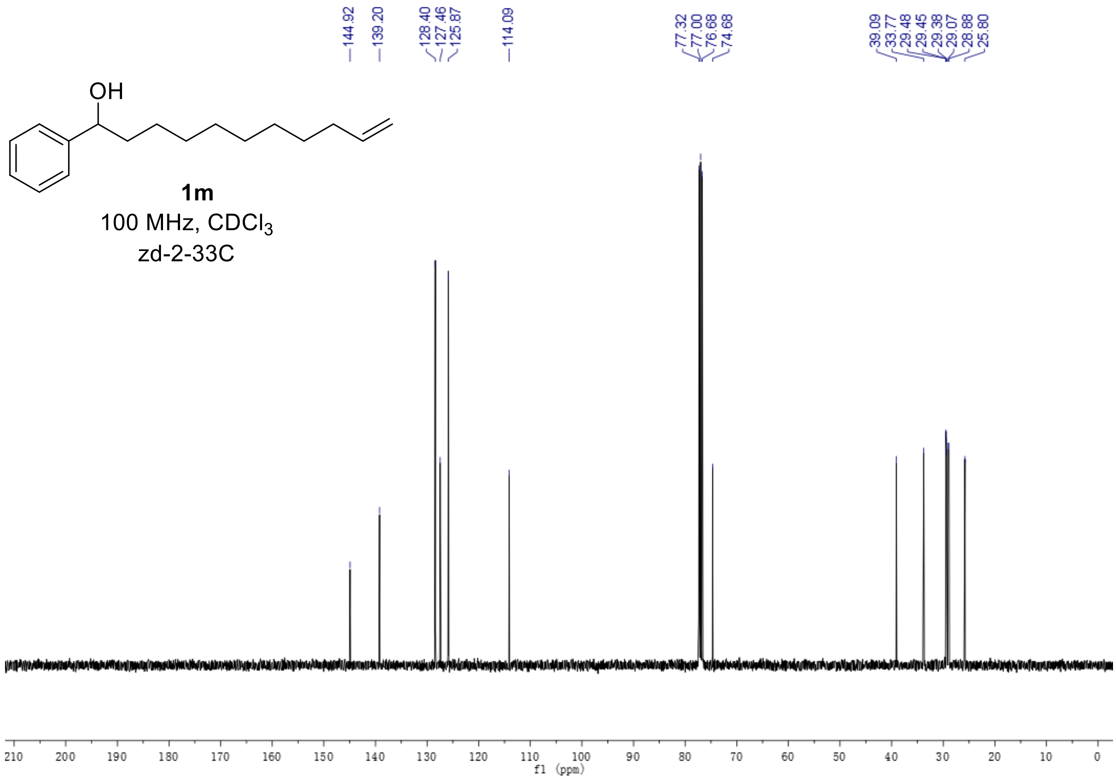

Supplementary Figure 24. ^{13}C NMR (100 MHz, $CDCl_3$, 25 °C) spectra for **1m**

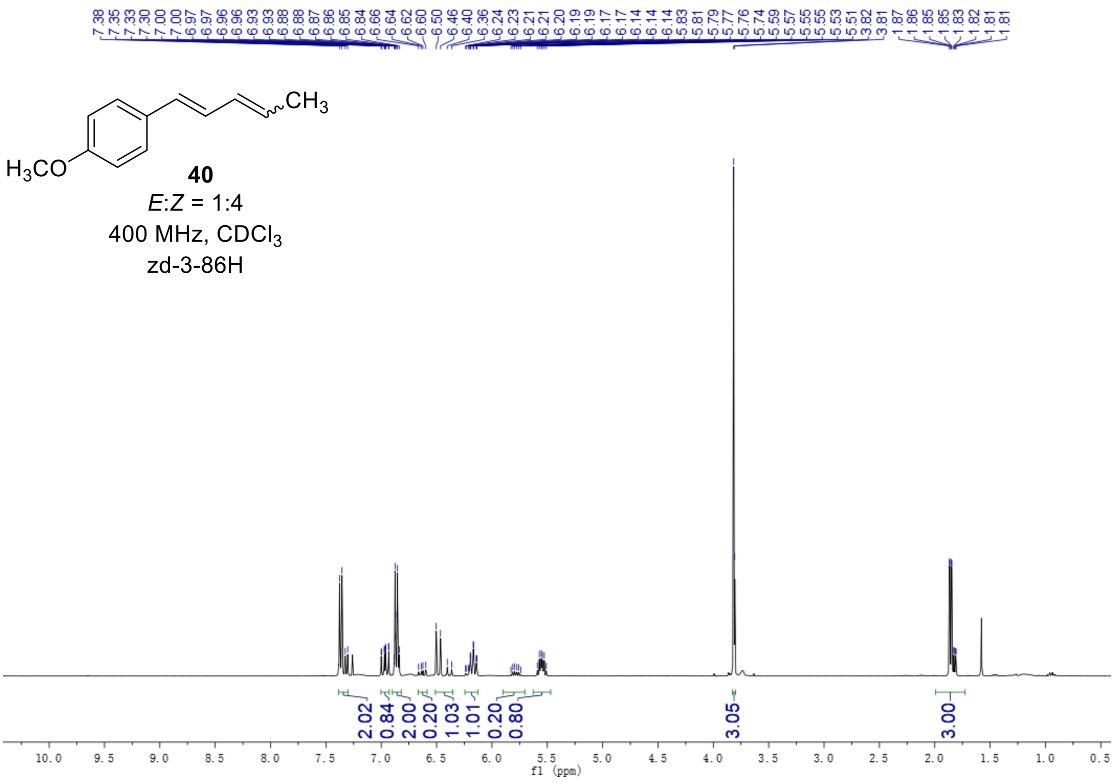

Supplementary Figure 25. ^{1}H NMR (400 MHz, $CDCl_3$, 25 °C) spectra for **40**

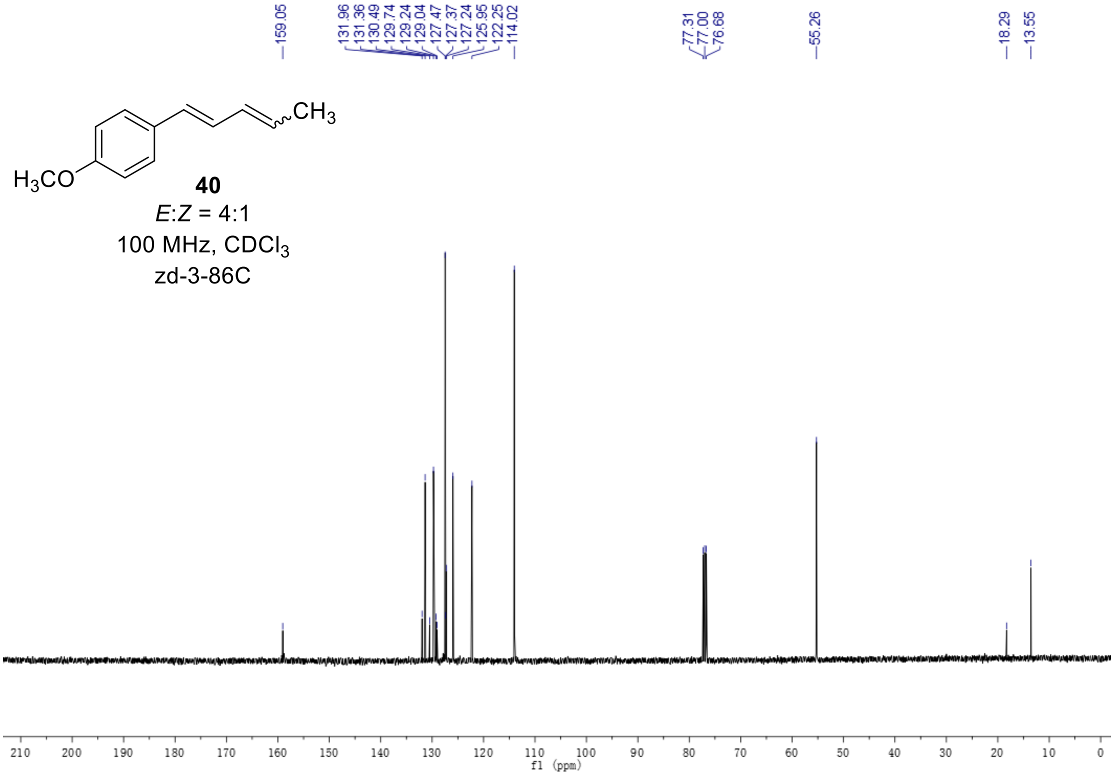

Supplementary Figure 26. ^{13}C NMR (100 MHz, $CDCl_3$, 25 °C) spectra for **40**

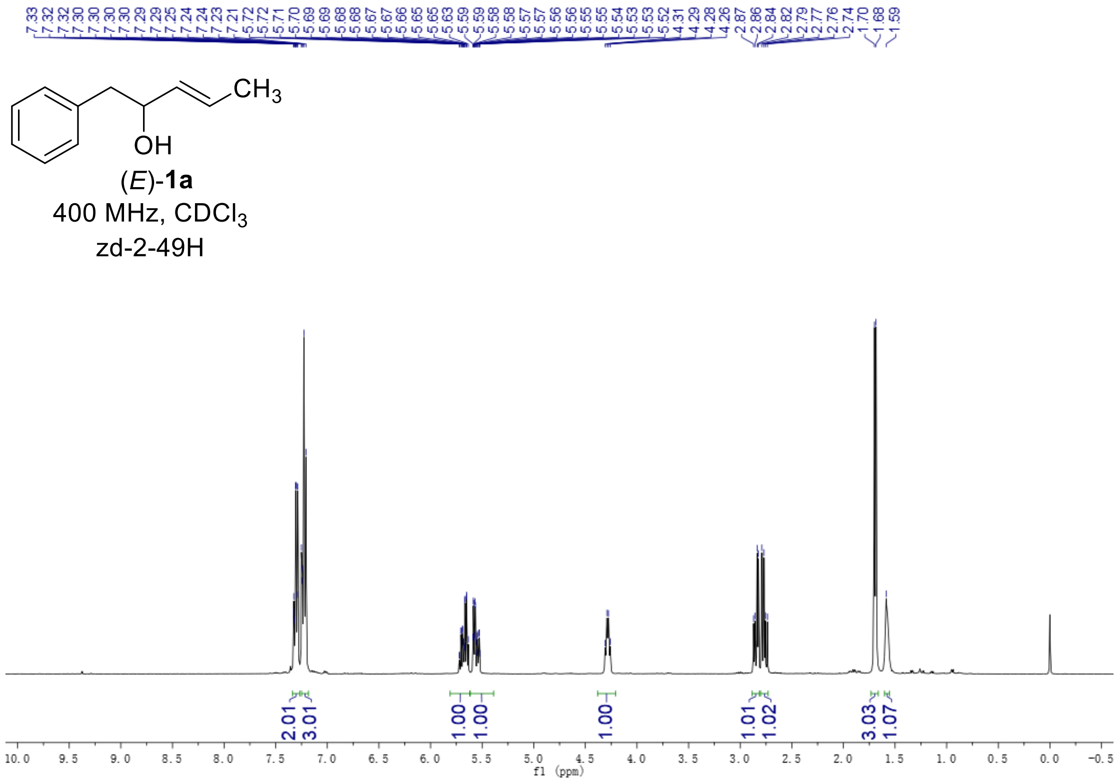

Supplementary Figure 27. ^{1}H NMR (400 MHz, $CDCl_3$, 25 °C) spectra for (*E*)-**1a**

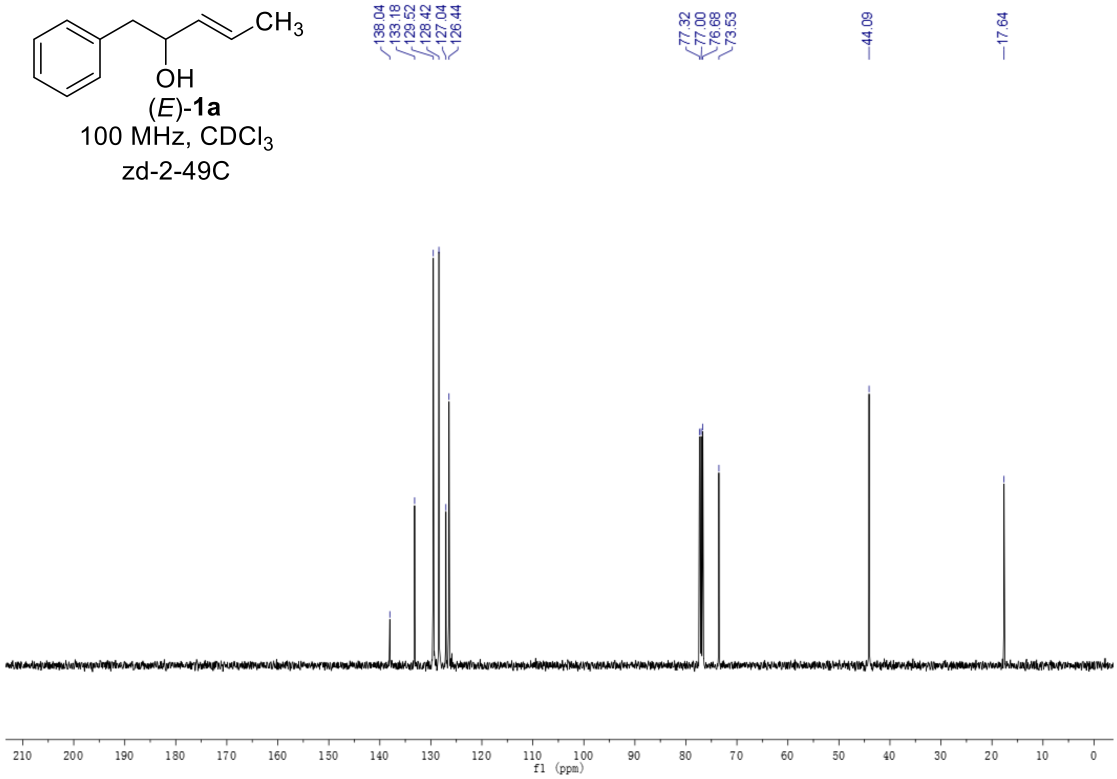

Supplementary Figure 28. ^{13}C NMR (100 MHz, $CDCl_3$, 25 °C) spectra for (*E*)-**1a**

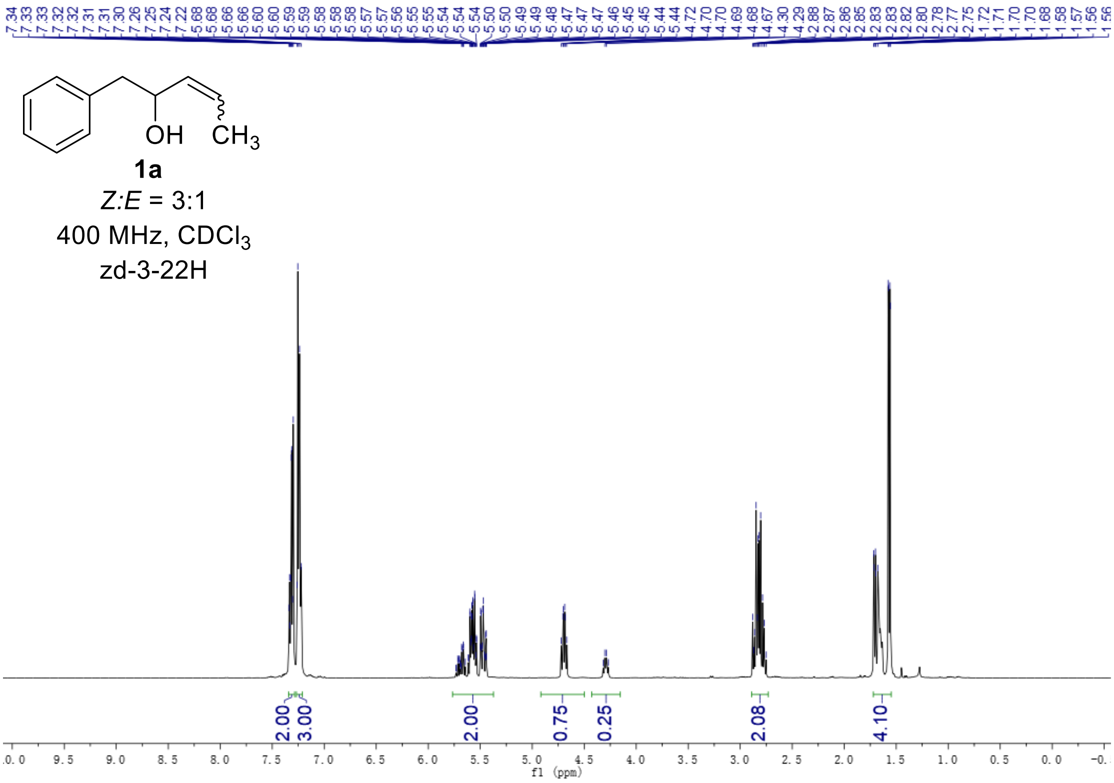

Supplementary Figure 29. ^{1}H NMR (400 MHz, $CDCl_3$, 25 °C) spectra for **1a**

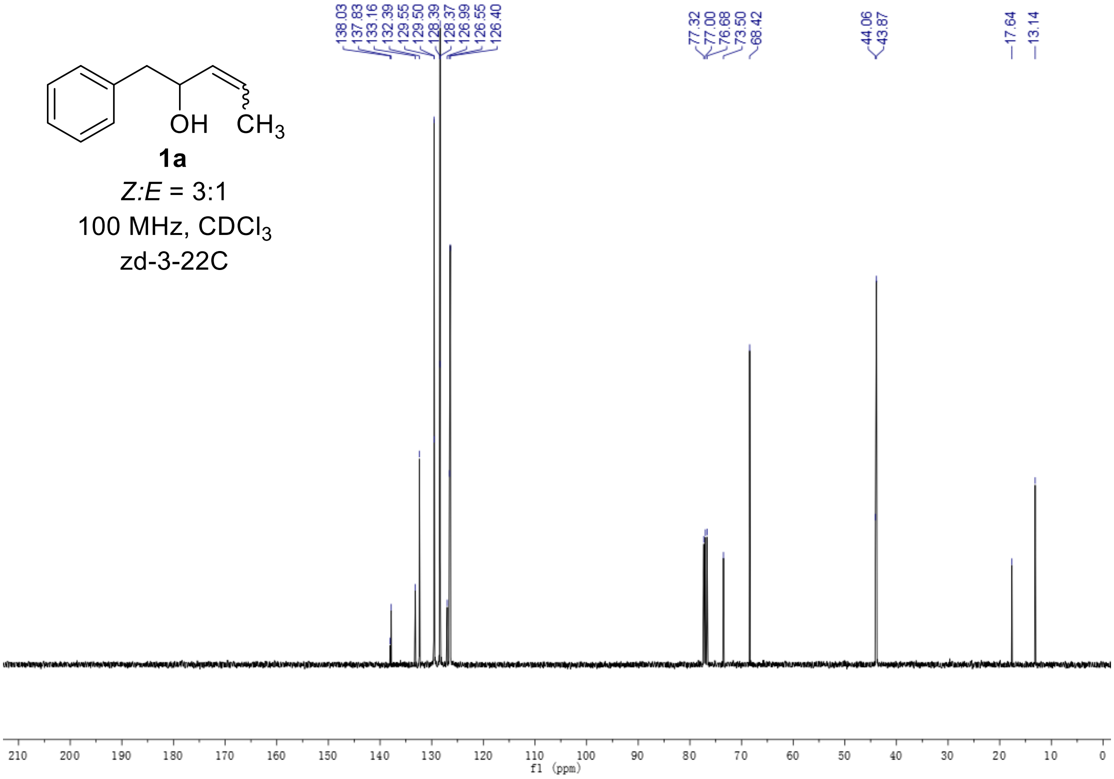

Supplementary Figure 30. ^{13}C NMR (100 MHz, $CDCl_3$, 25 °C) spectra for **1a**

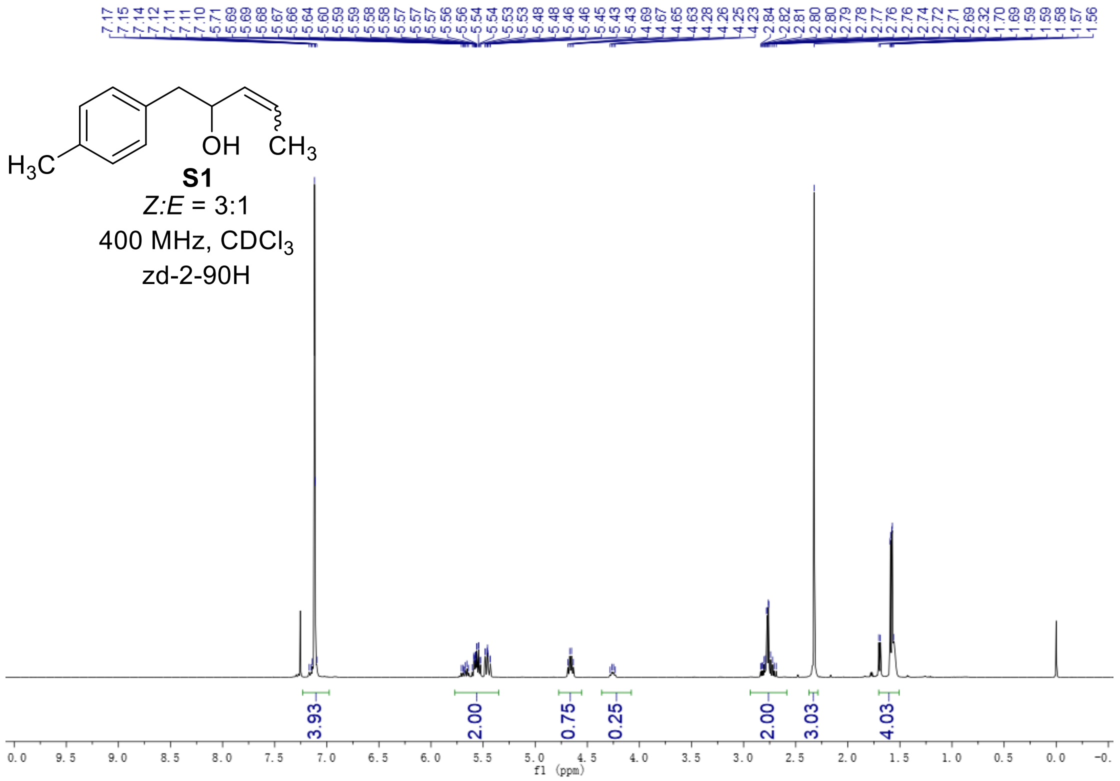

Supplementary Figure 31. ^{1}H NMR (400 MHz, $CDCl_3$, 25 °C) spectra for **S1**

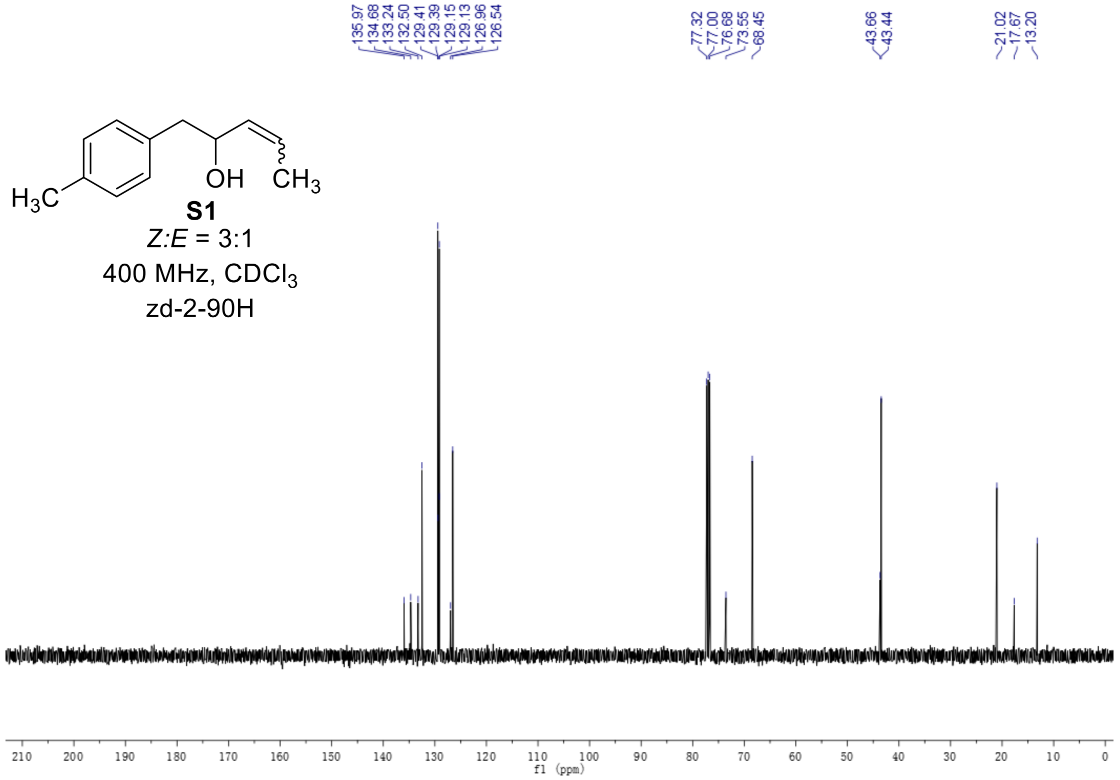

Supplementary Figure 32. ^{13}C NMR (100 MHz, $CDCl_3$, 25 °C) spectra for **S1**

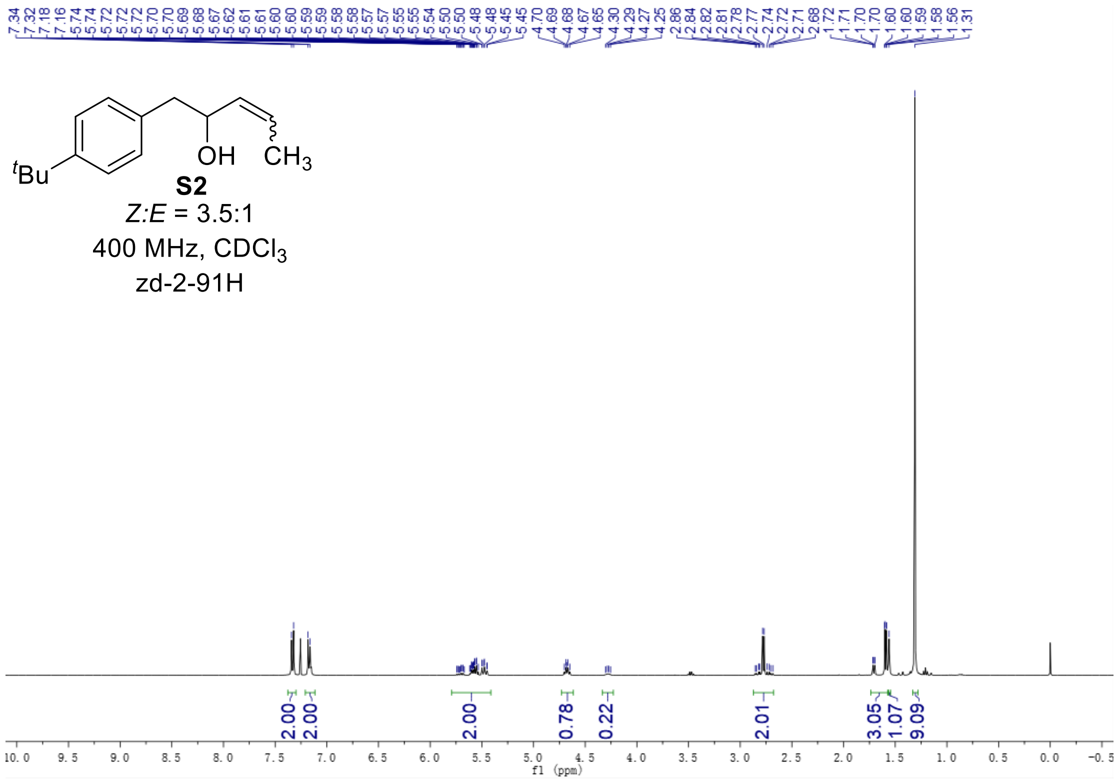

Supplementary Figure 33. ${}^{1}H$ NMR (400 MHz, $CDCl_3$, 25 °C) spectra for **S2**

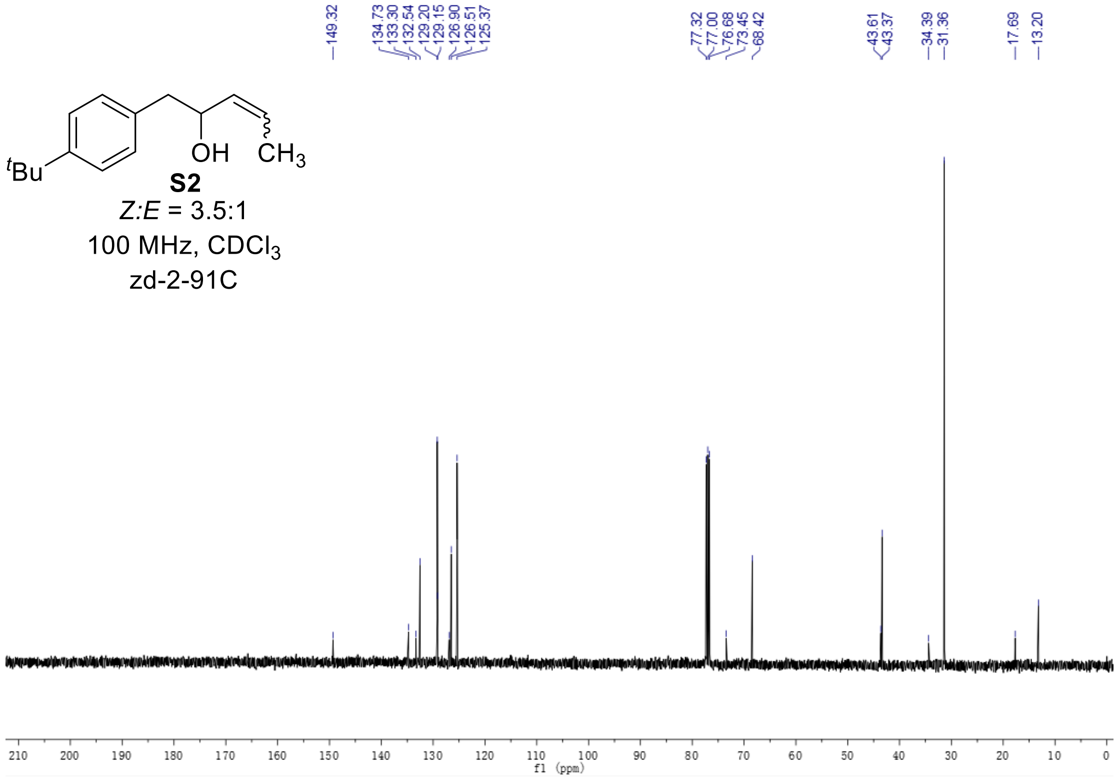

Supplementary Figure 34. ${}^{13}C$ NMR (100 MHz, $CDCl_3$, 25 °C) spectra for **S2**

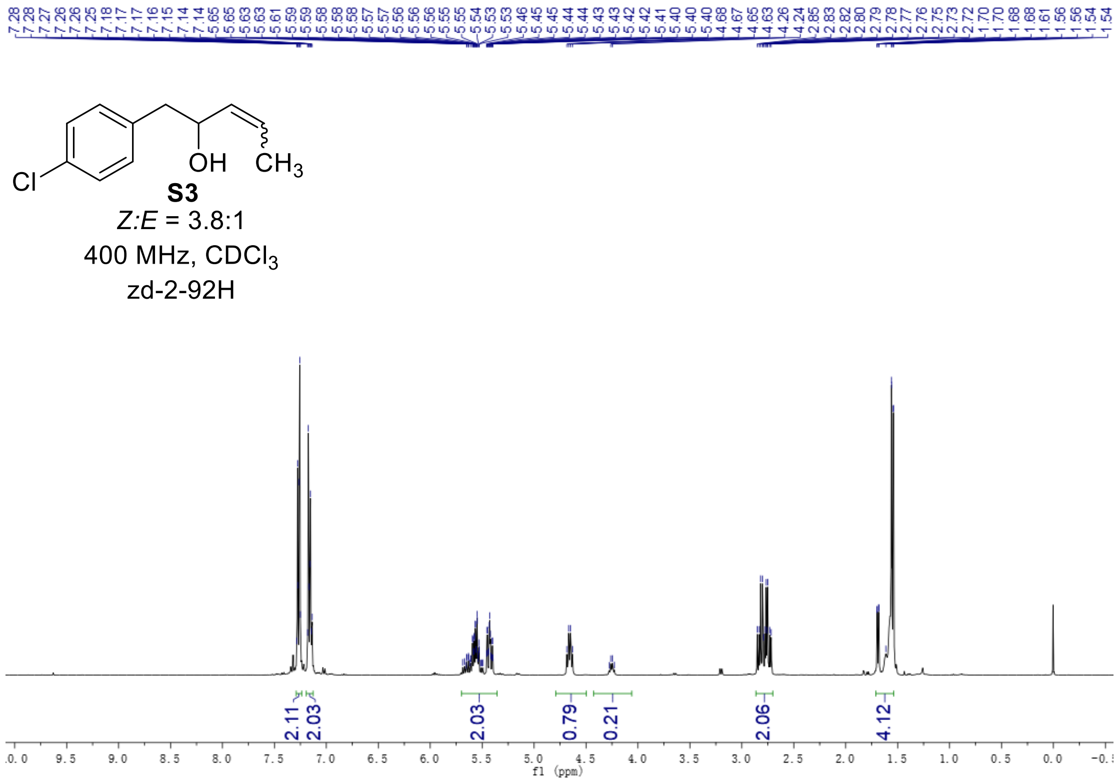

Supplementary Figure 35. ^{1}H NMR (400 MHz, $CDCl_3$, 25 °C) spectra for **S3**

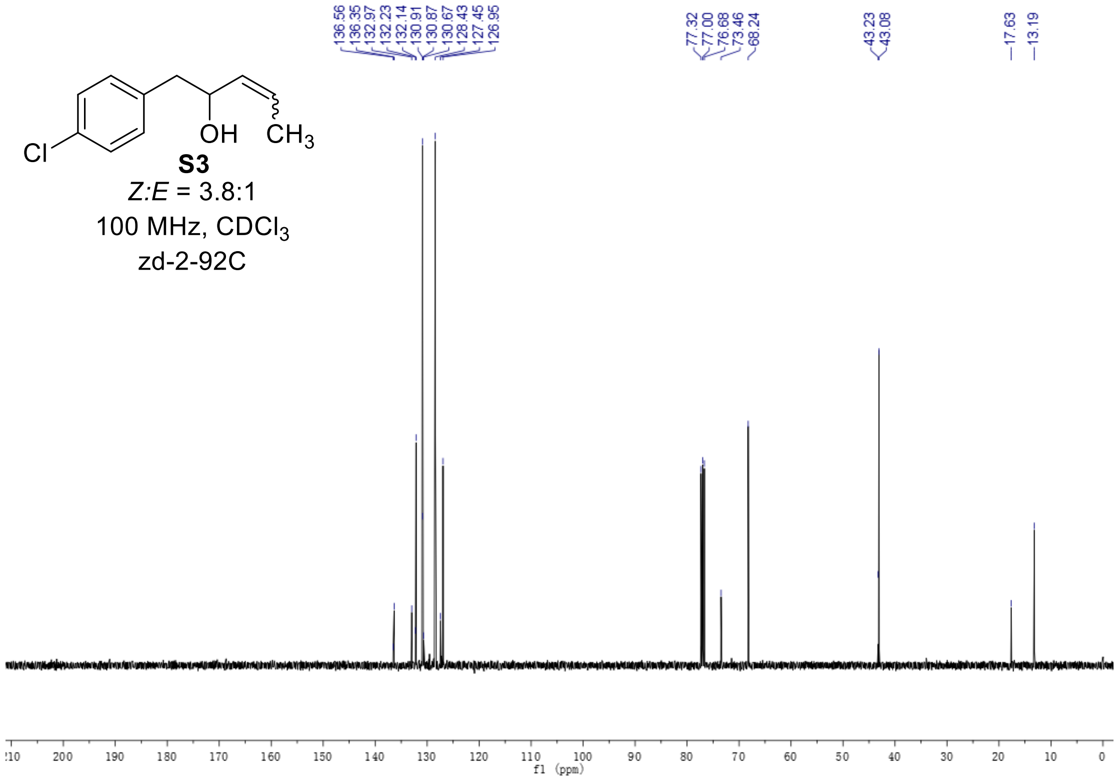

Supplementary Figure 36. ^{13}C NMR (100 MHz, $CDCl_3$, 25 °C) spectra for **S3**

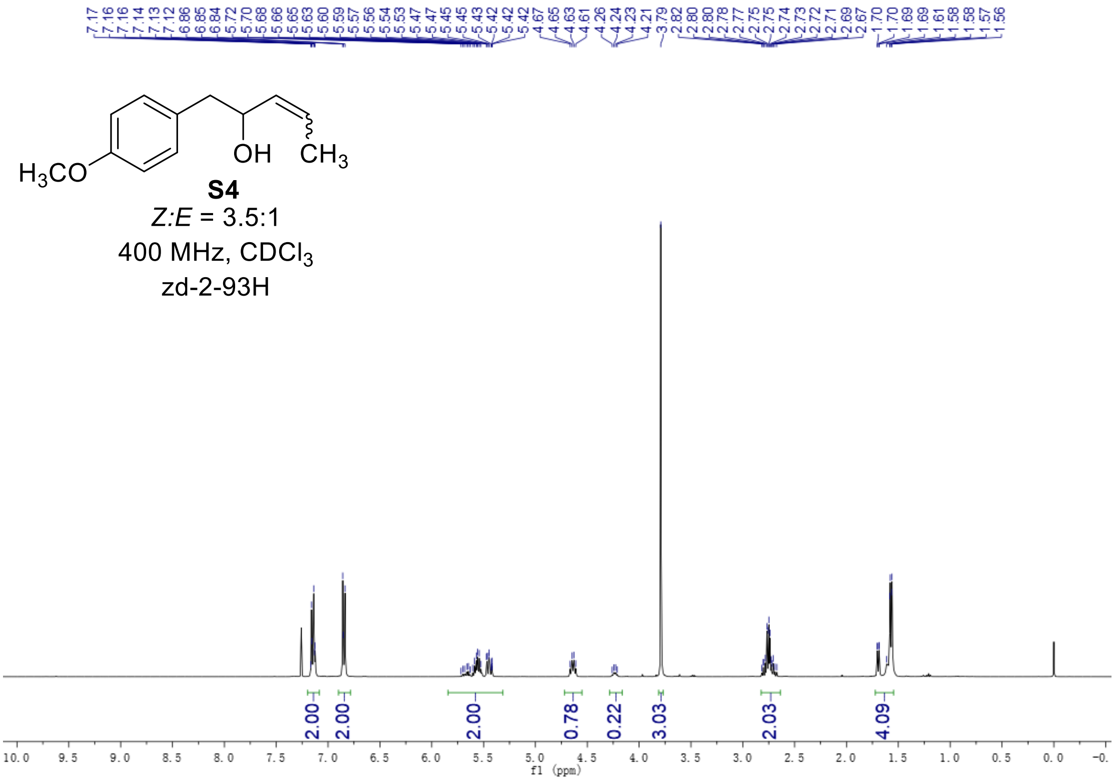

Supplementary Figure 37. ^{1}H NMR (400 MHz, $CDCl_3$, 25 °C) spectra for **S4**

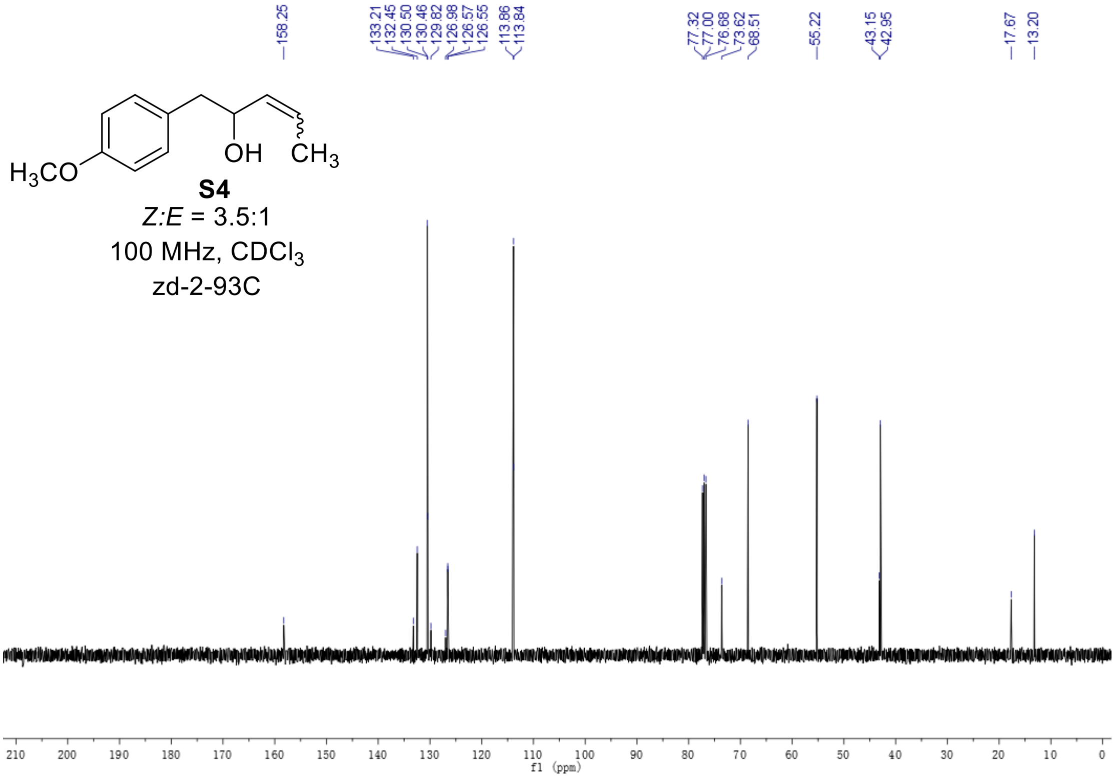

Supplementary Figure 38. ^{13}C NMR (100 MHz, $CDCl_3$, 25 °C) spectra for **S4**

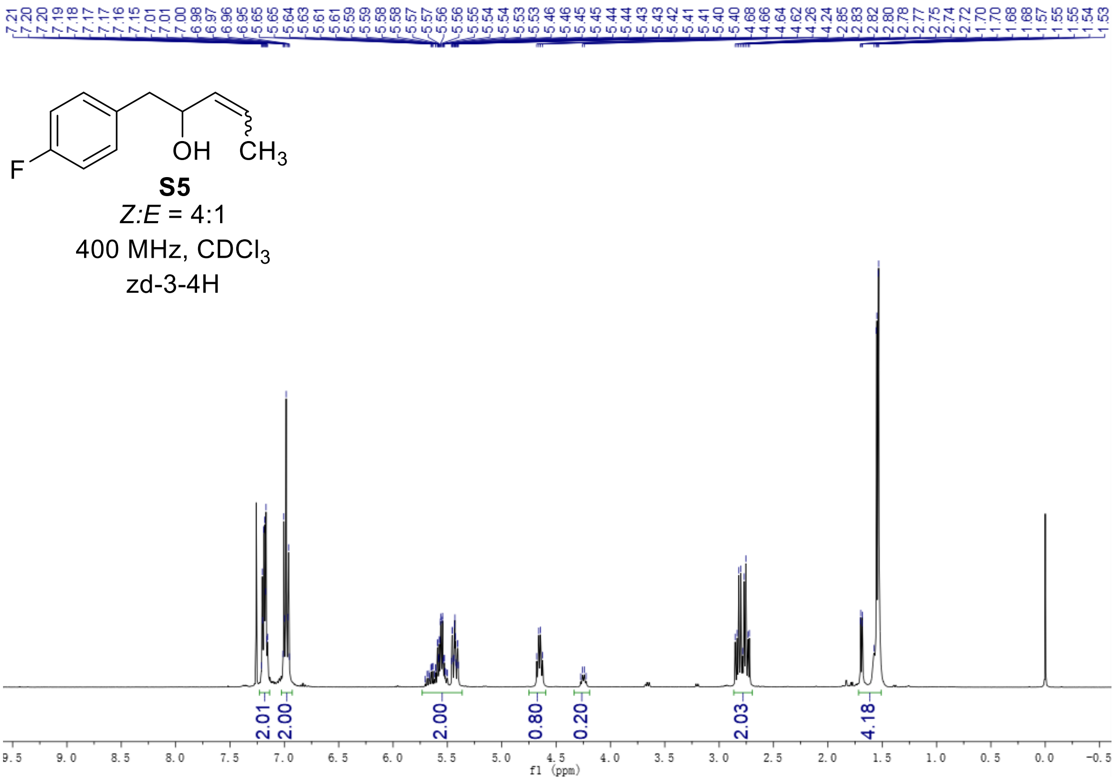

Supplementary Figure 39. ^{1}H NMR (400 MHz, $CDCl_3$, 25 °C) spectra for **S5**

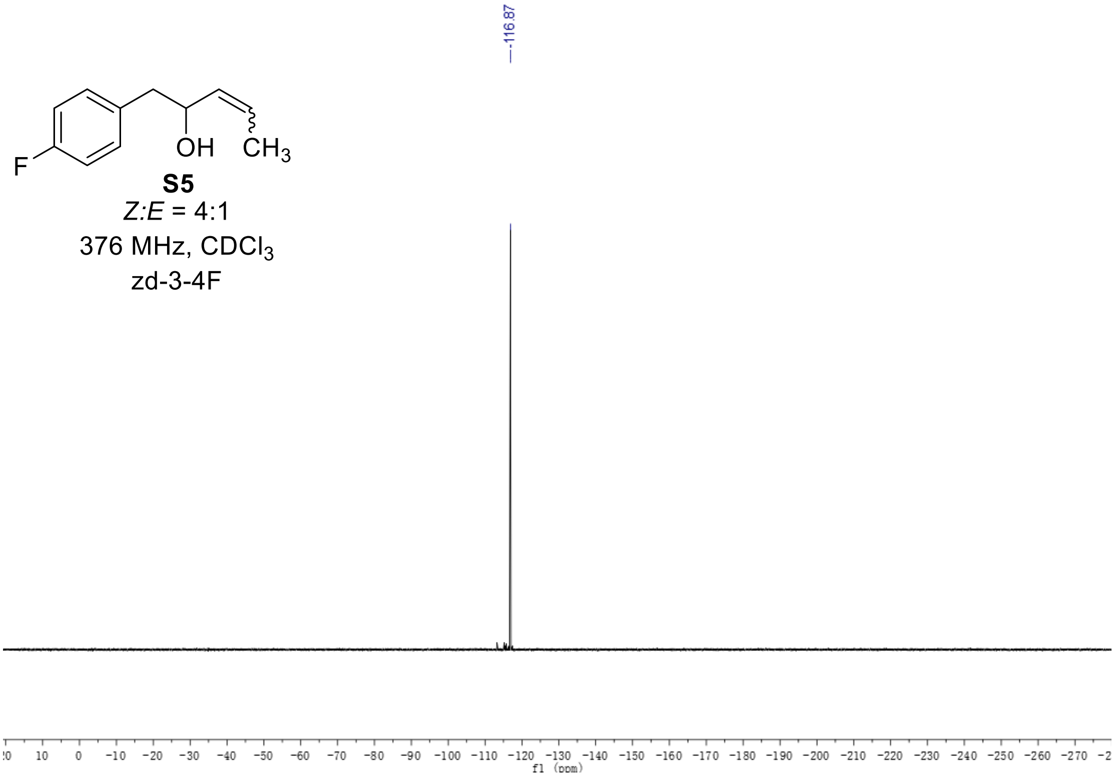

Supplementary Figure 40. ^{19}F NMR (376 MHz, $CDCl_3$, 25 °C) spectra for **S5**

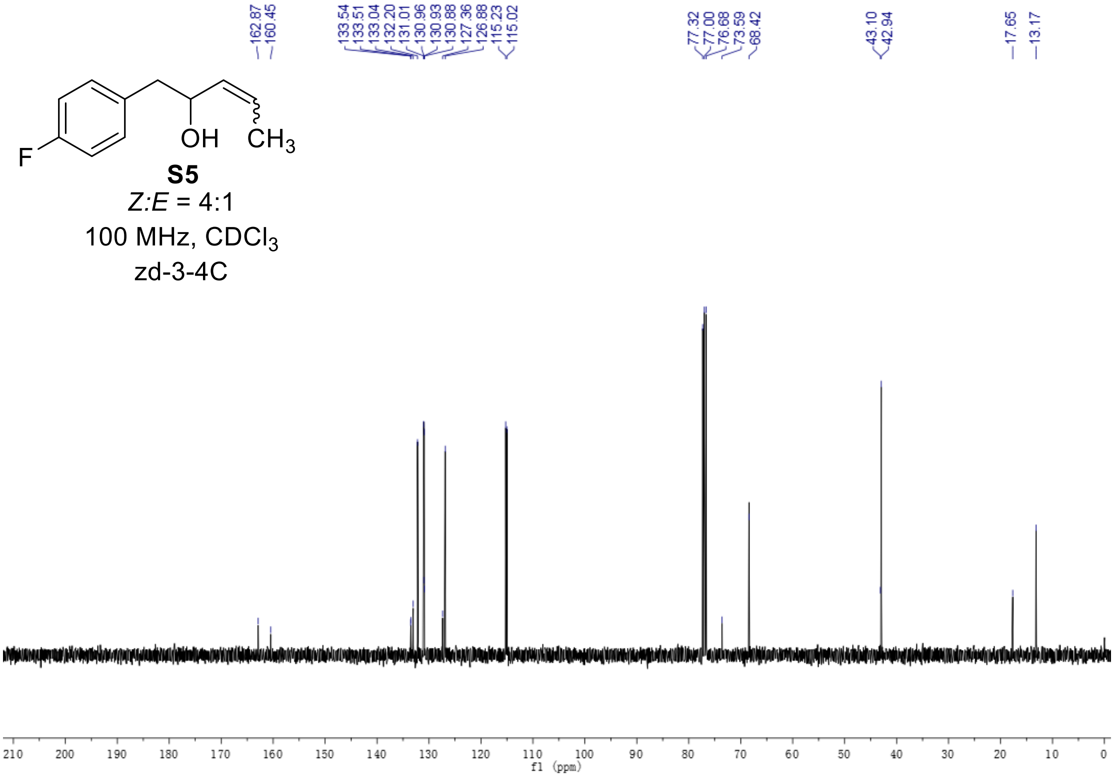

Supplementary Figure 41. ^{13}C NMR (100 MHz, $CDCl_3$, 25 °C) spectra for **S5**

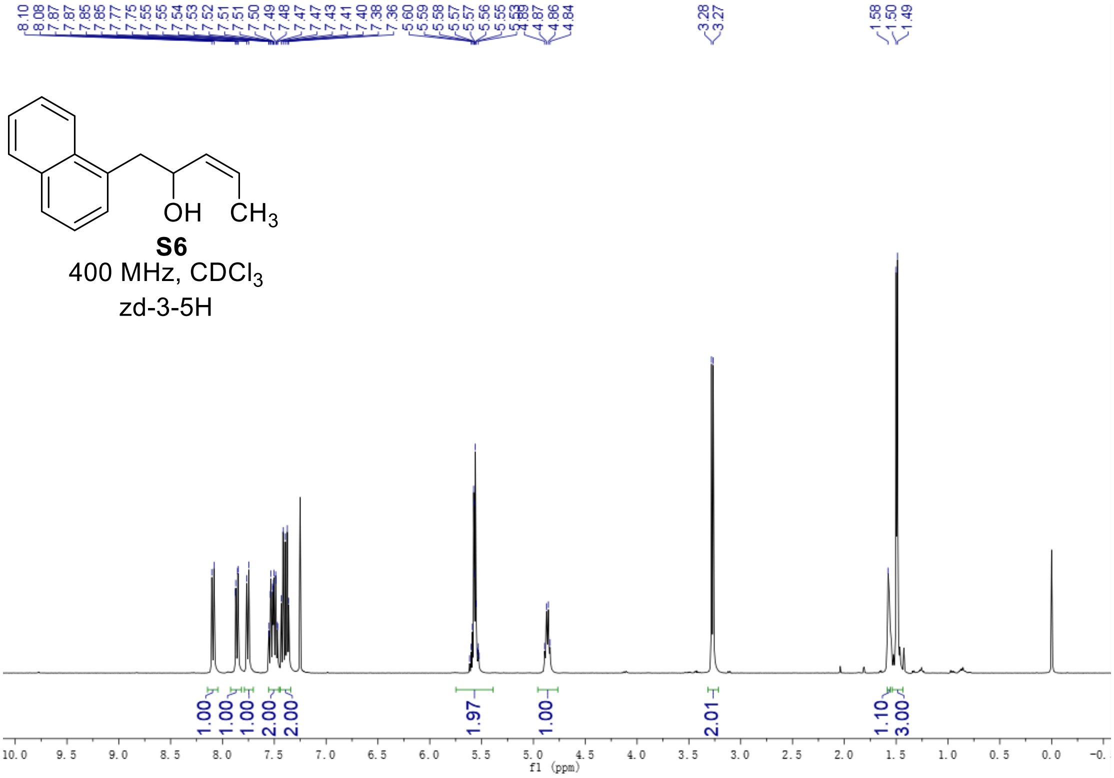

Supplementary Figure 42. ^{1}H NMR (400 MHz, $CDCl_3$, 25 °C) spectra for **S6**

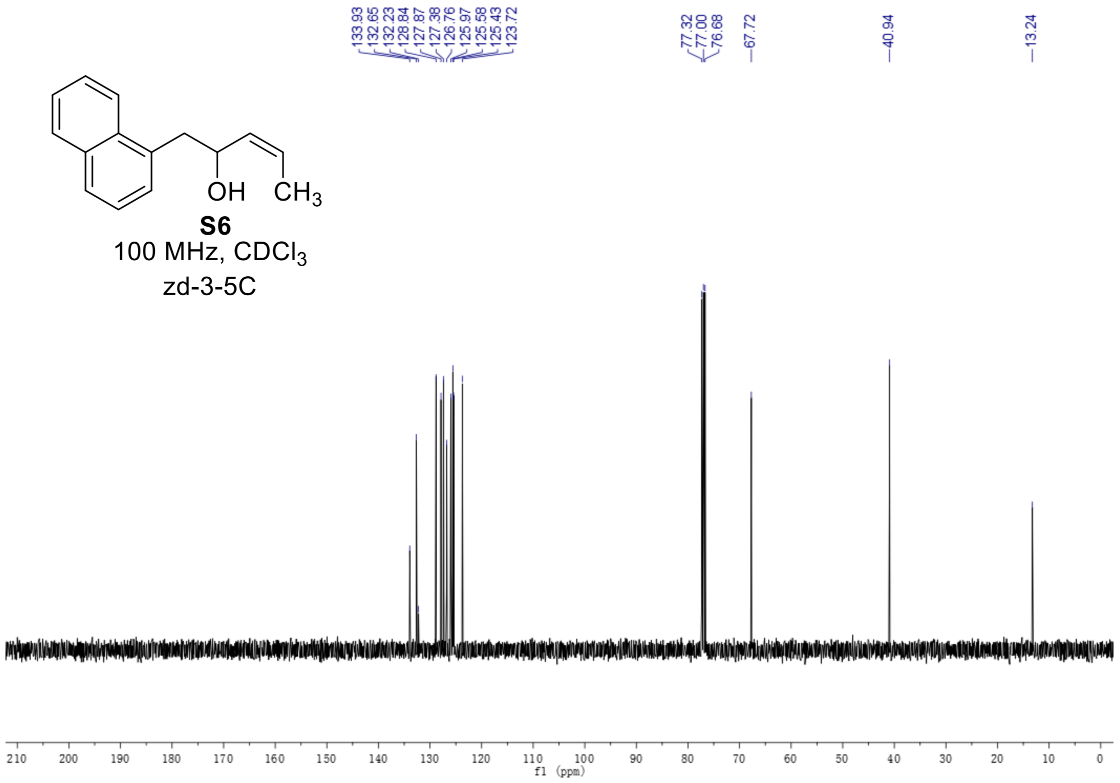

Supplementary Figure 43. ^{13}C NMR (100 MHz, $CDCl_3$, 25 °C) spectra for **S6**

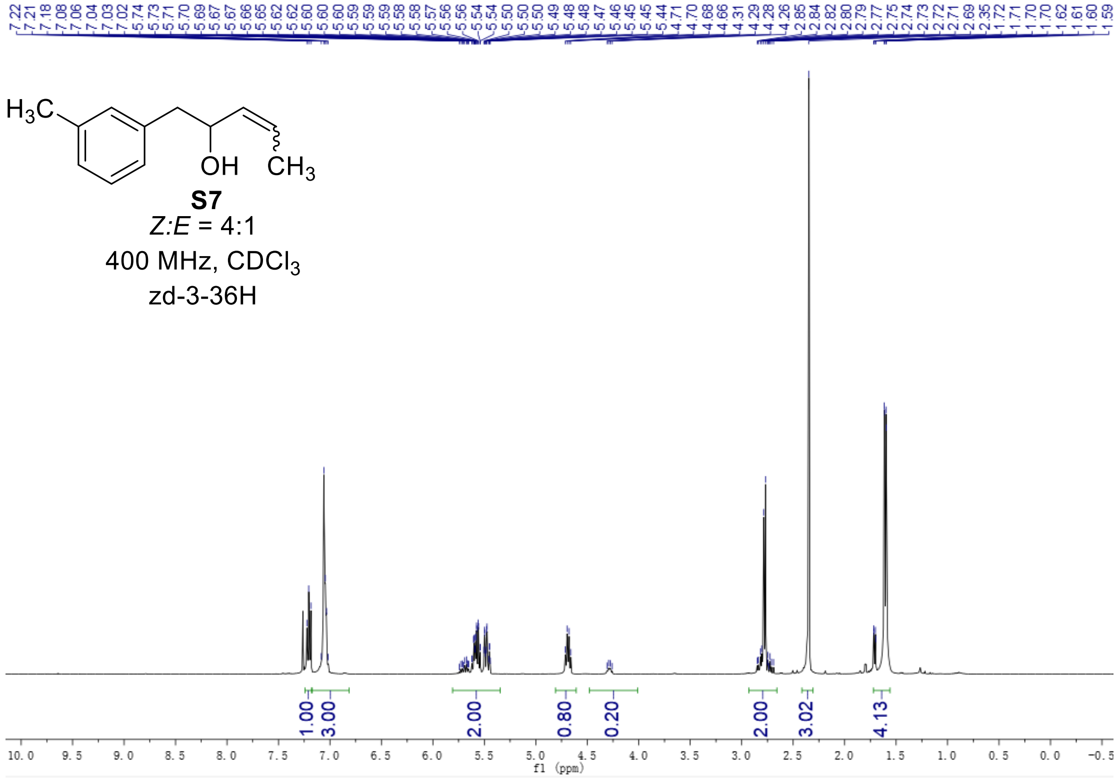

Supplementary Figure 44. ^{1}H NMR (400 MHz, $CDCl_3$, 25 °C) spectra for **S7**

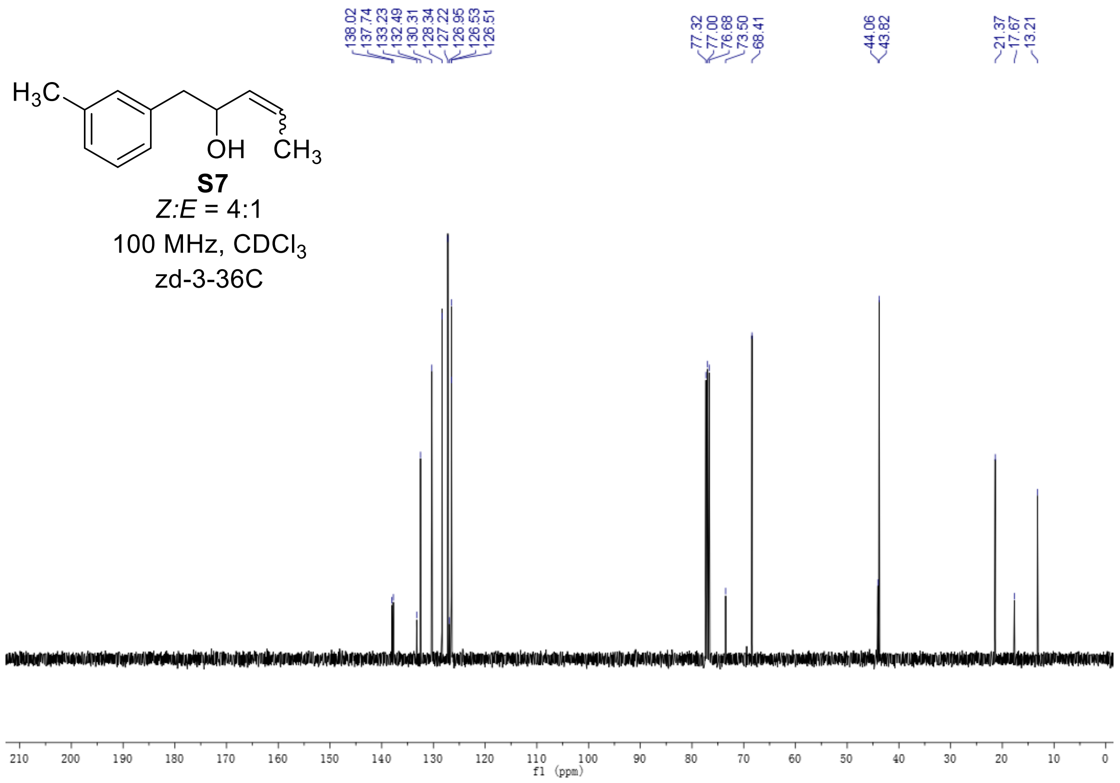

Supplementary Figure 45. ^{13}C NMR (100 MHz, $CDCl_3$, 25 °C) spectra for **S7**

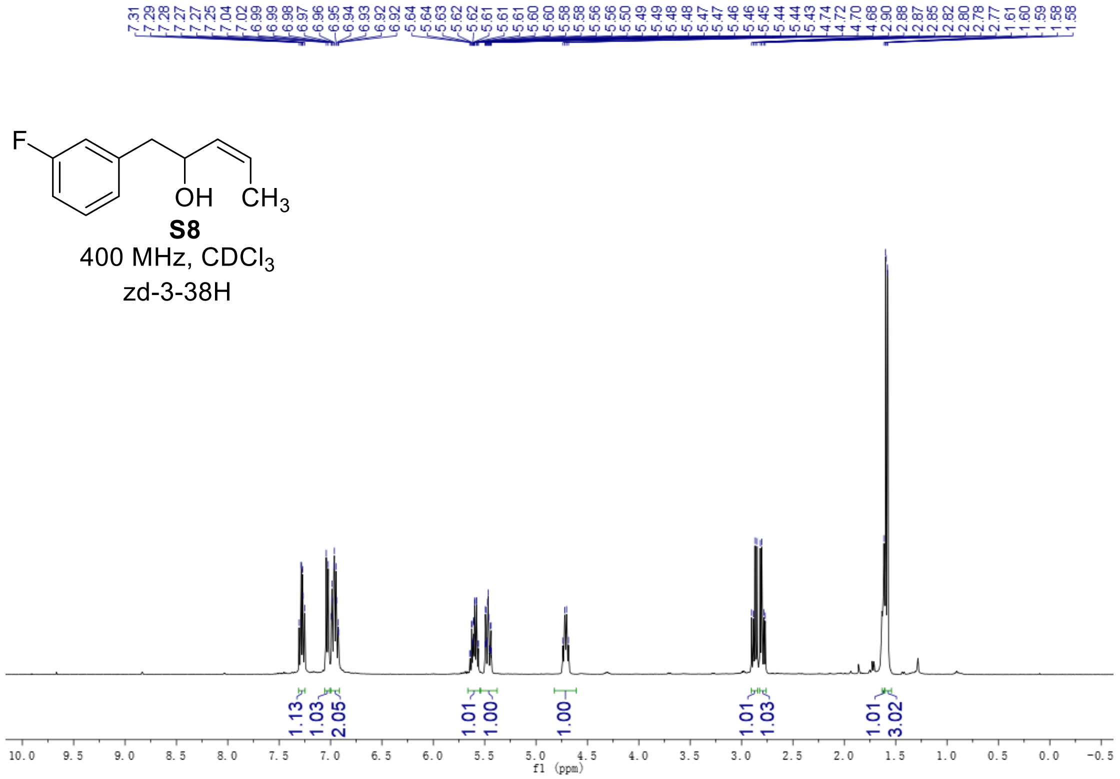

Supplementary Figure 46. ^{1}H NMR (400 MHz, $CDCl_3$, 25 °C) spectra for **S8**

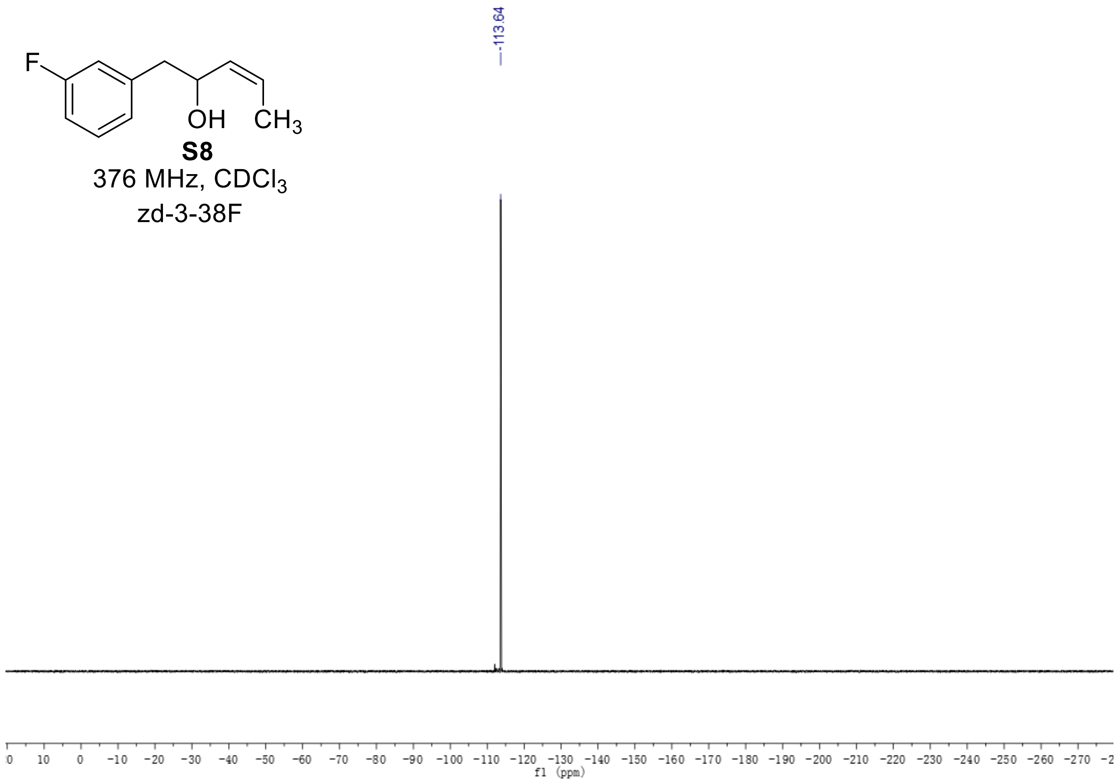

Supplementary Figure 47. ^{19}F NMR (376 MHz, $CDCl_3$, 25 °C) spectra for **S8**

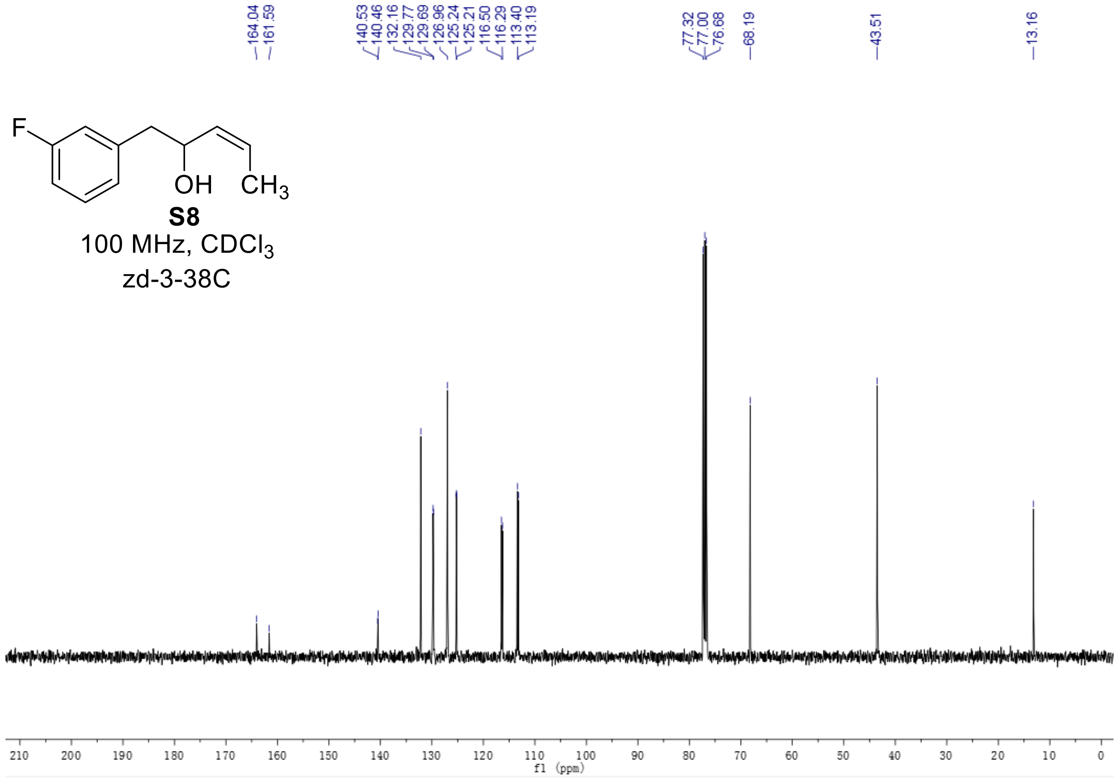

Supplementary Figure 48. ^{13}C NMR (100 MHz, $CDCl_3$, 25 °C) spectra for **S8**

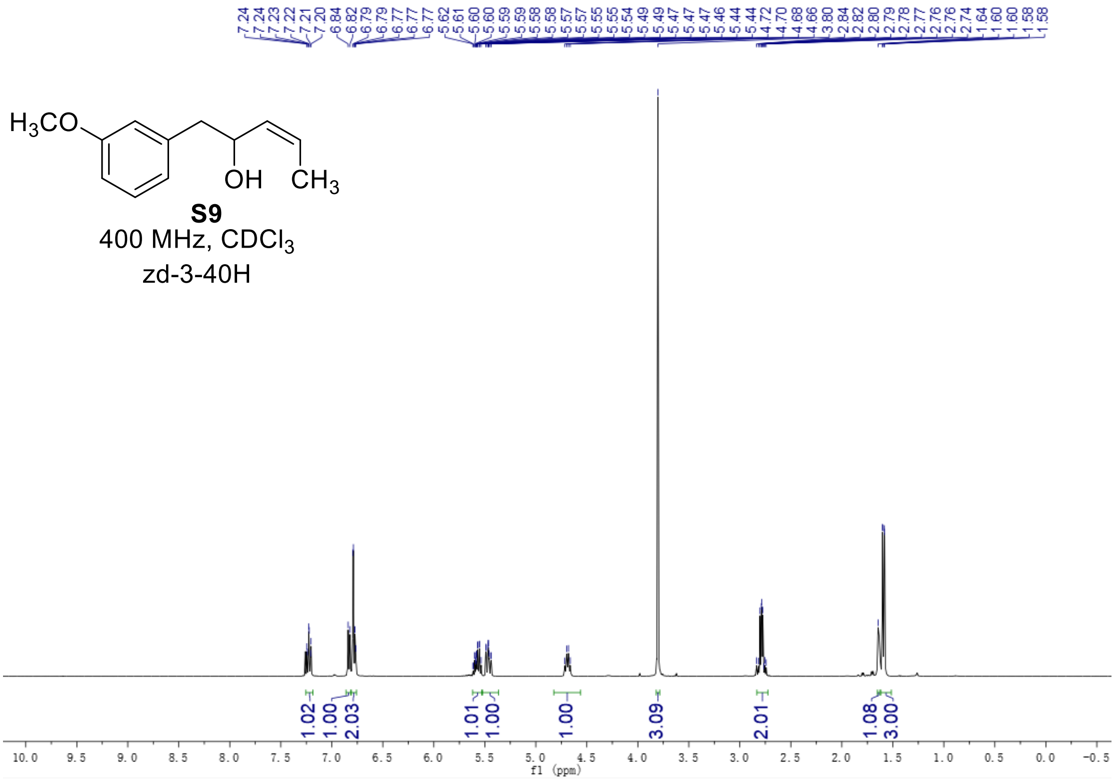

Supplementary Figure 49. ^{1}H NMR (400 MHz, $CDCl_3$, 25 °C) spectra for **S9**

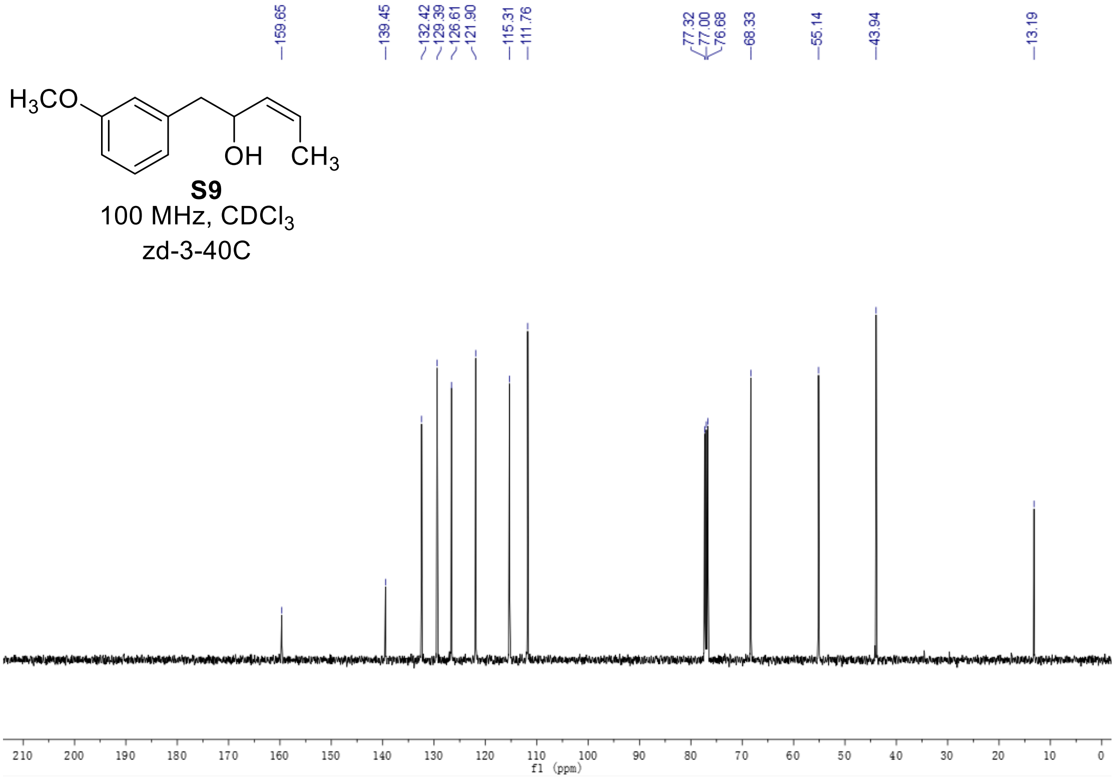

Supplementary Figure 50. ^{13}C NMR (100 MHz, $CDCl_3$, 25 °C) spectra for **S9**

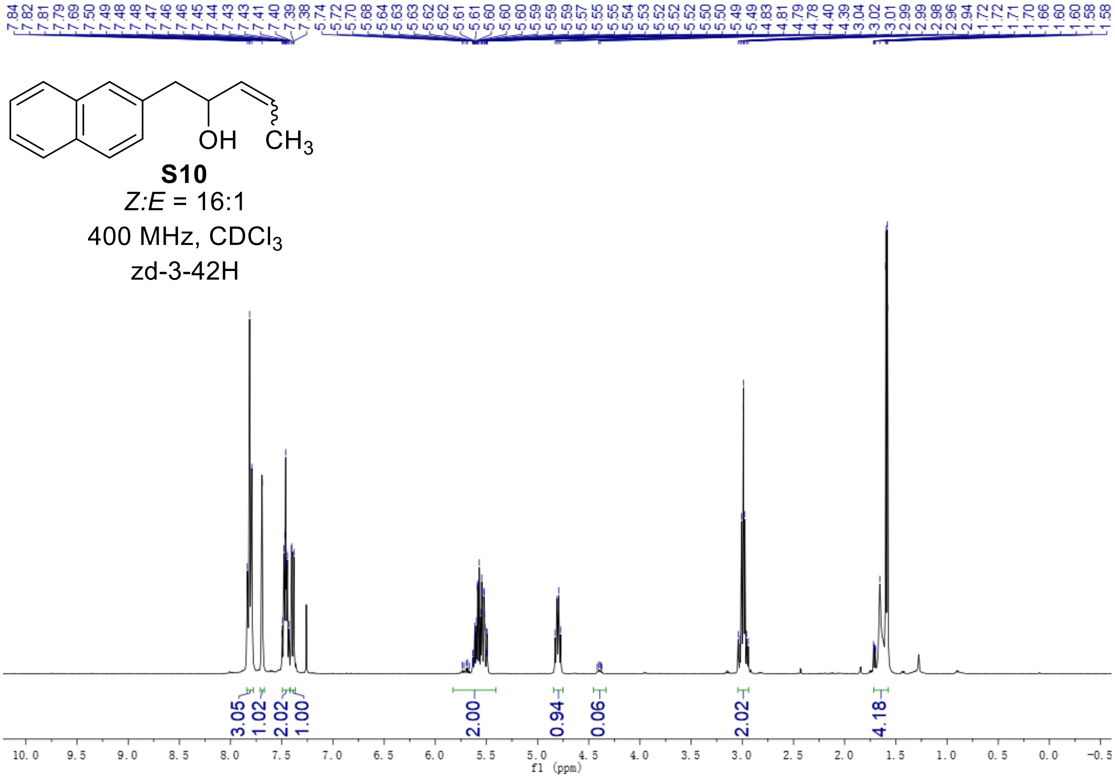

Supplementary Figure 51. ^{1}H NMR (400 MHz, $CDCl_3$, 25 °C) spectra for **S10**

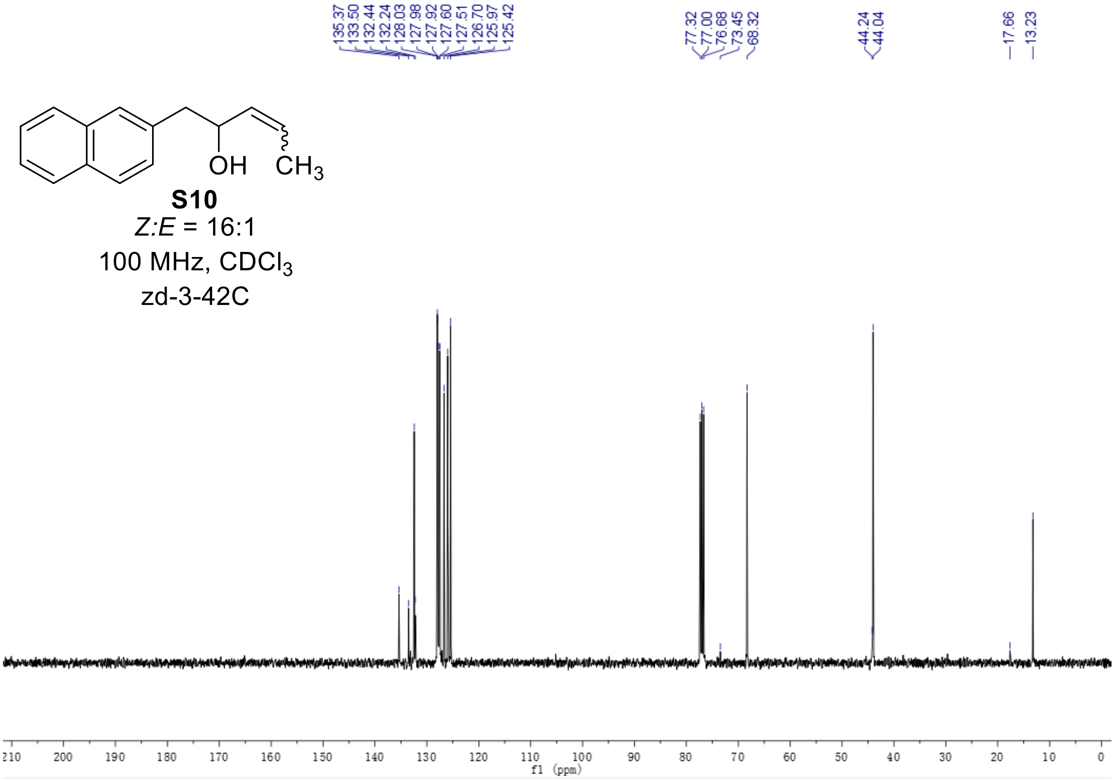

Supplementary Figure 52. ^{13}C NMR (100 MHz, $CDCl_3$, 25 °C) spectra for **S10**

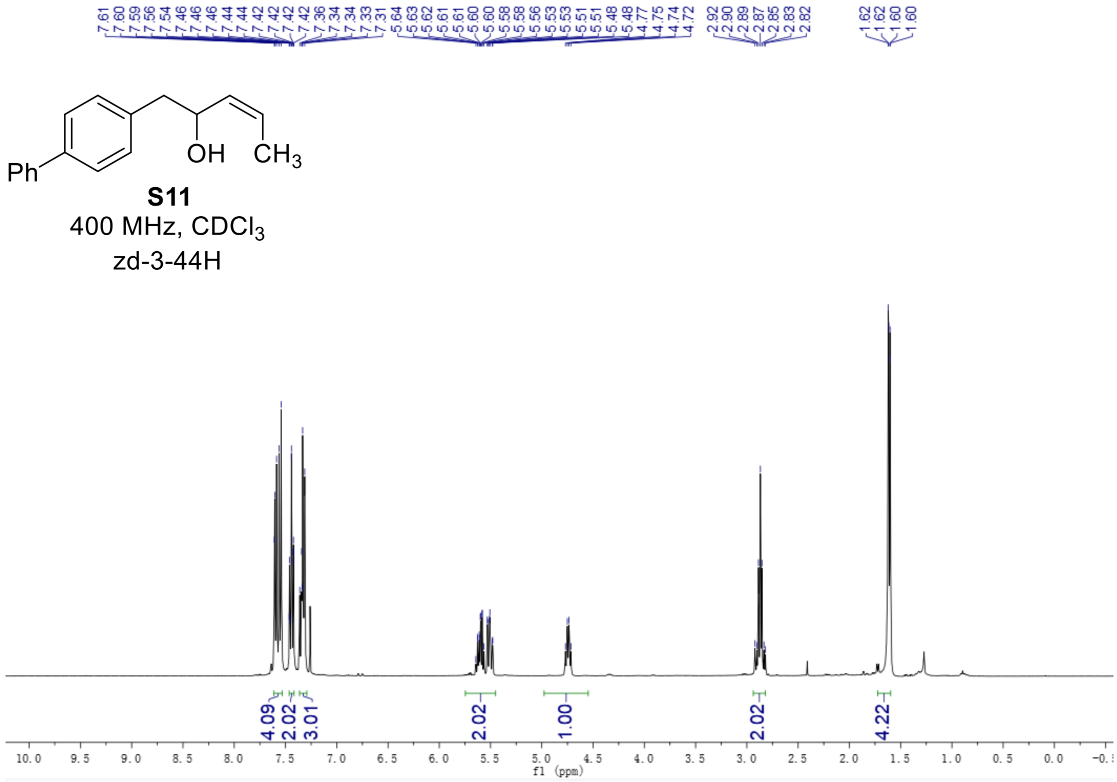

Supplementary Figure 53. ^{1}H NMR (400 MHz, $CDCl_3$, 25 °C) spectra for **S11**

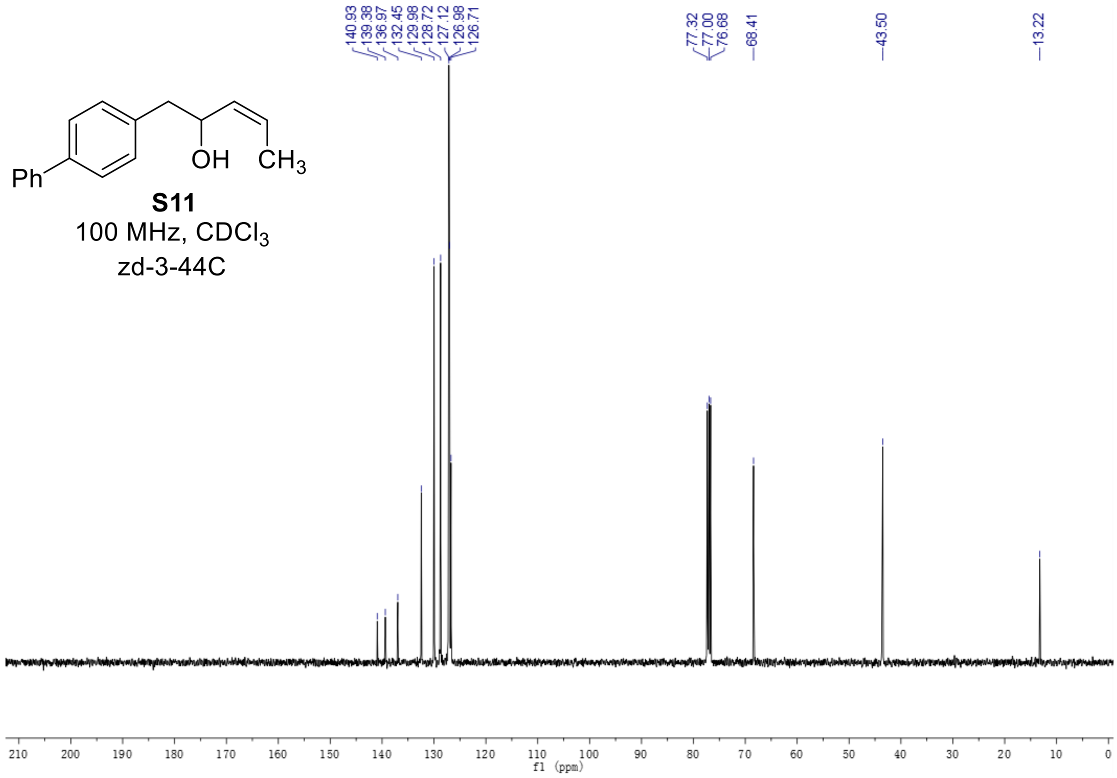

Supplementary Figure 54. ^{13}C NMR (100 MHz, $CDCl_3$, 25 °C) spectra for **S11**

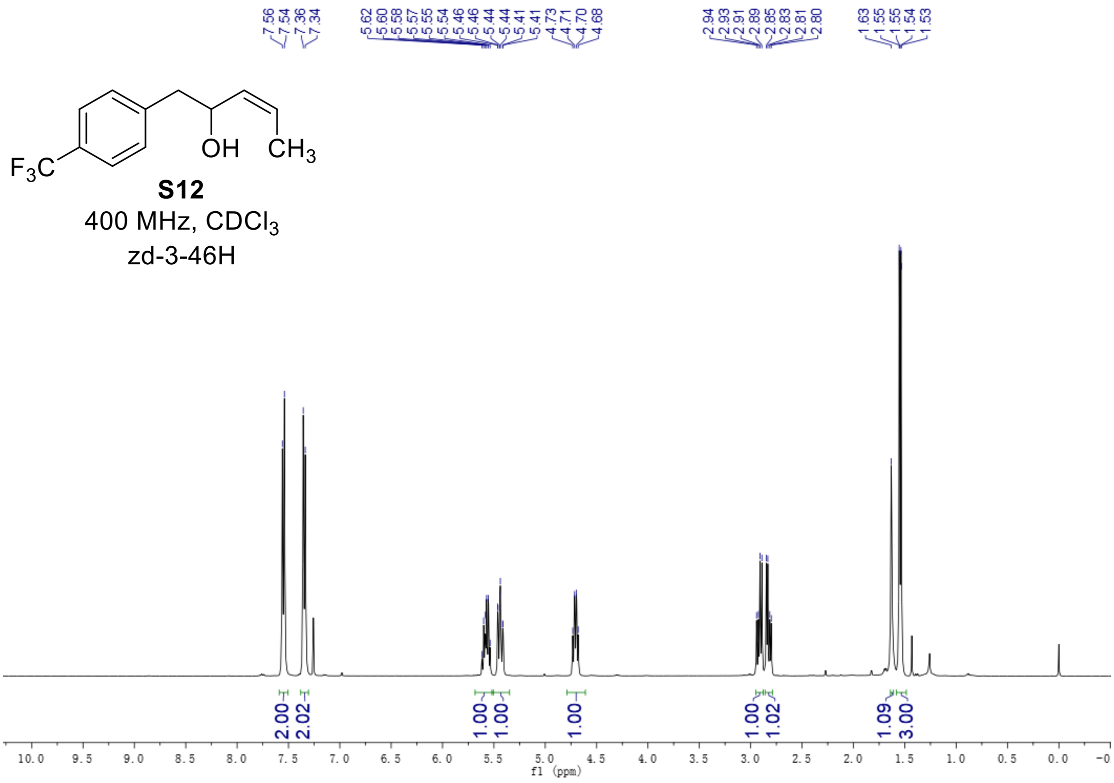

Supplementary Figure 55. ^{1}H NMR (400 MHz, $CDCl_3$, 25 °C) spectra for **S12**

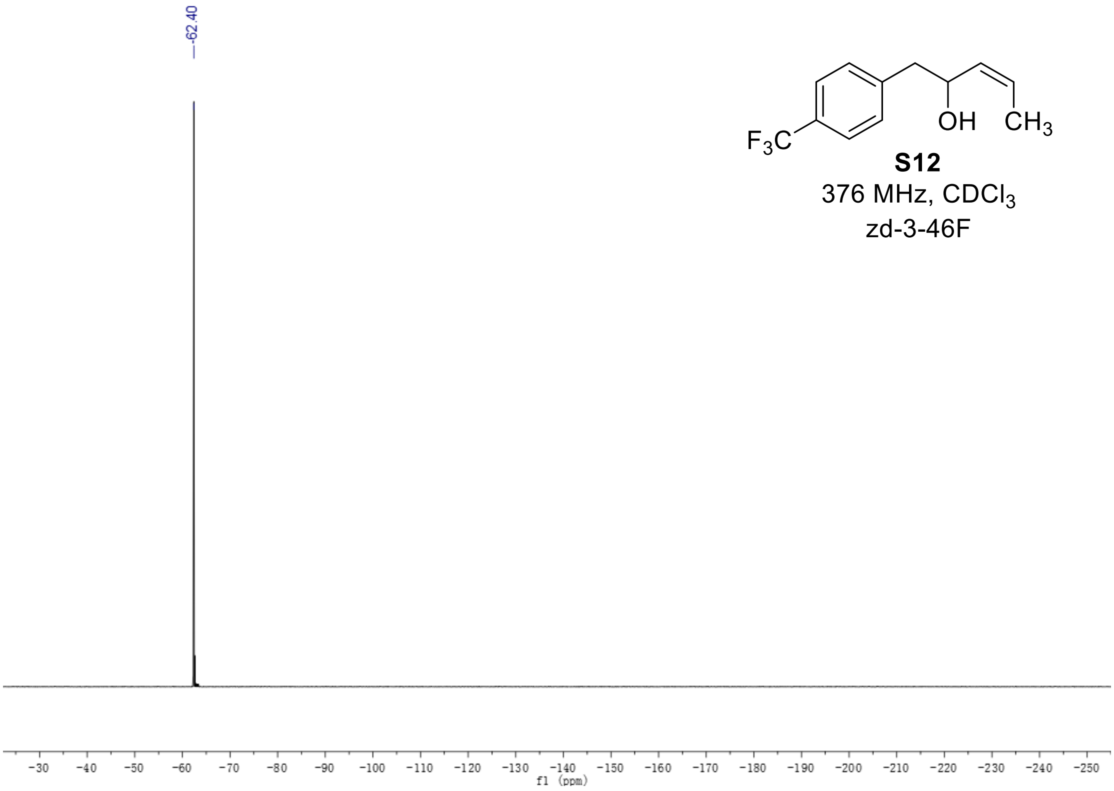

Supplementary Figure 56. ^{19}F NMR (376 MHz, $CDCl_3$, 25 °C) spectra for **S12**

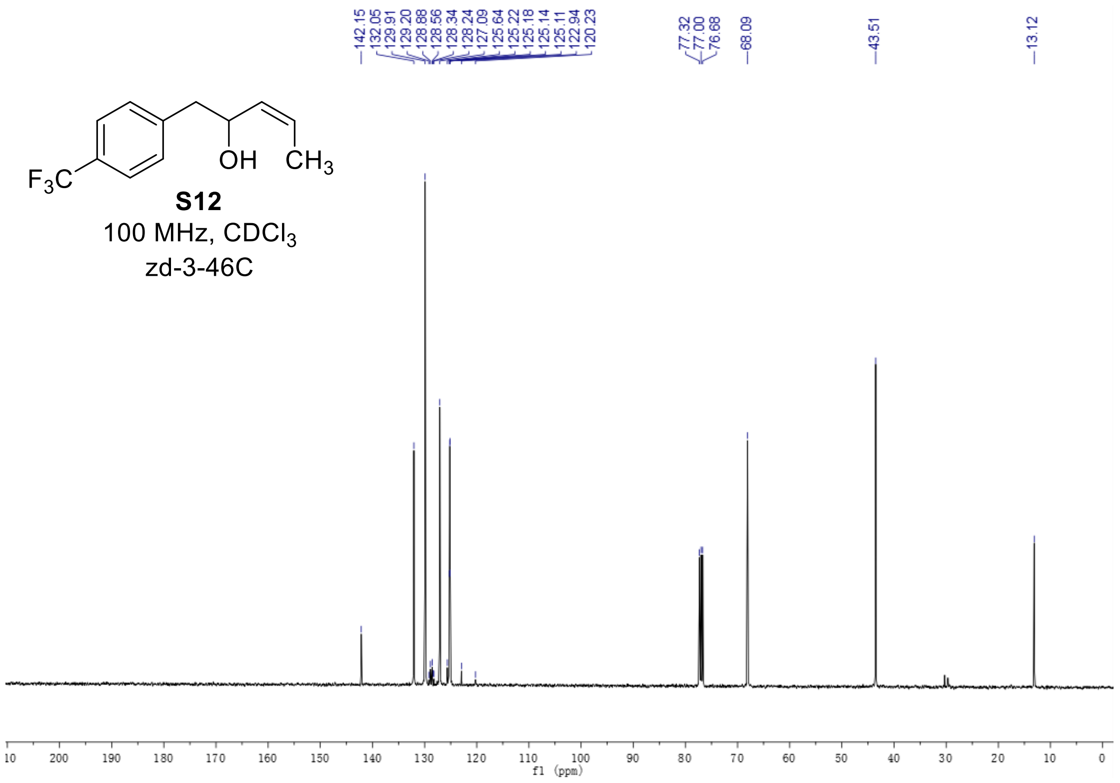

Supplementary Figure 57. ^{13}C NMR (100 MHz, $CDCl_3$, 25 °C) spectra for **S12**

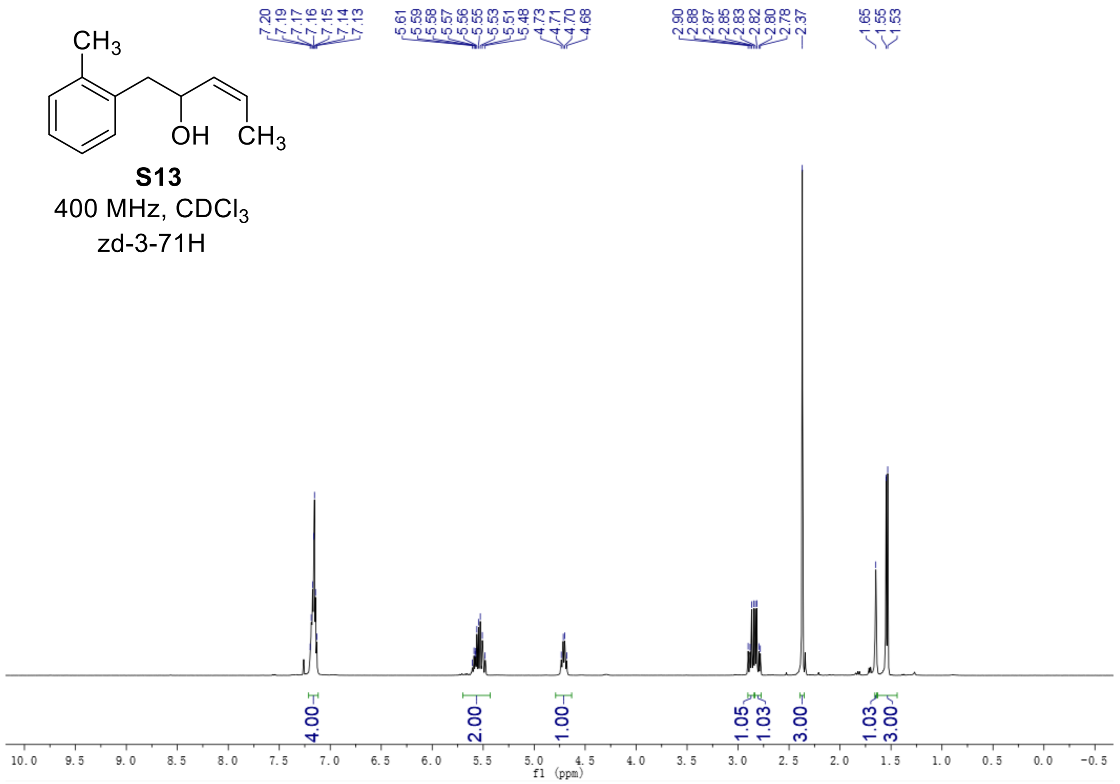

Supplementary Figure 58. ^{1}H NMR (400 MHz, $CDCl_3$, 25 °C) spectra for **S13**

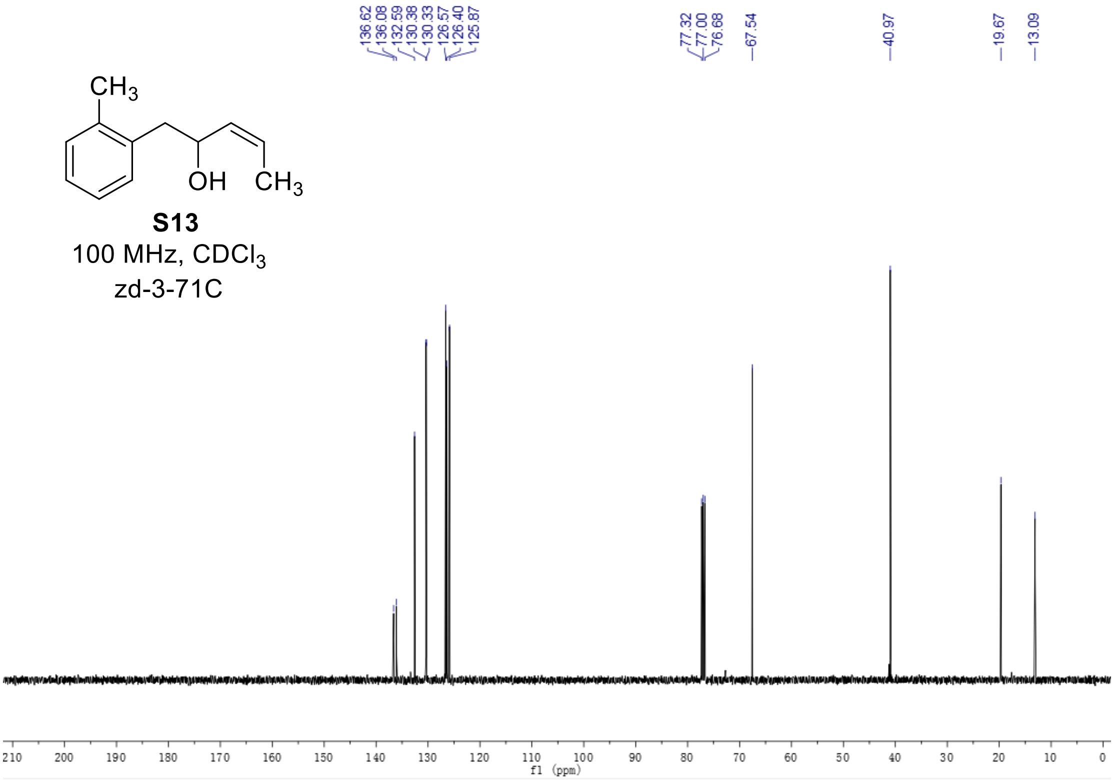

Supplementary Figure 59. ^{13}C NMR (100 MHz, $CDCl_3$, 25 °C) spectra for **S13**

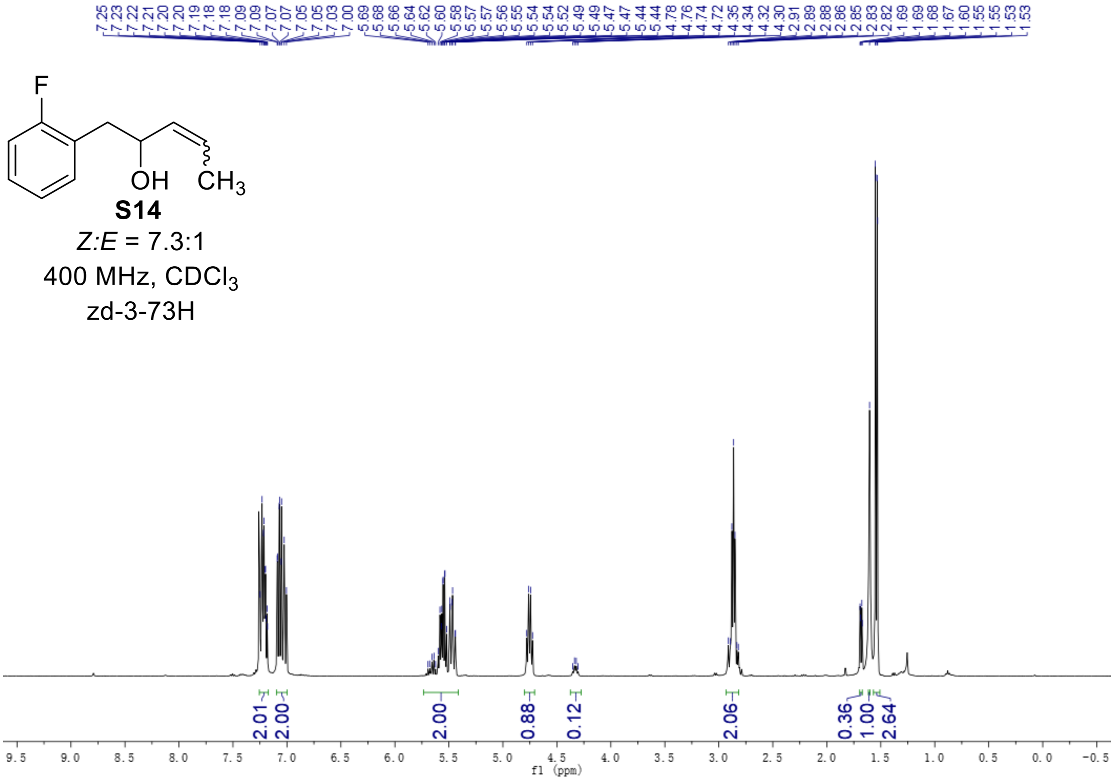

Supplementary Figure 60. ^{1}H NMR (400 MHz, $CDCl_3$, 25 °C) spectra for **S14**

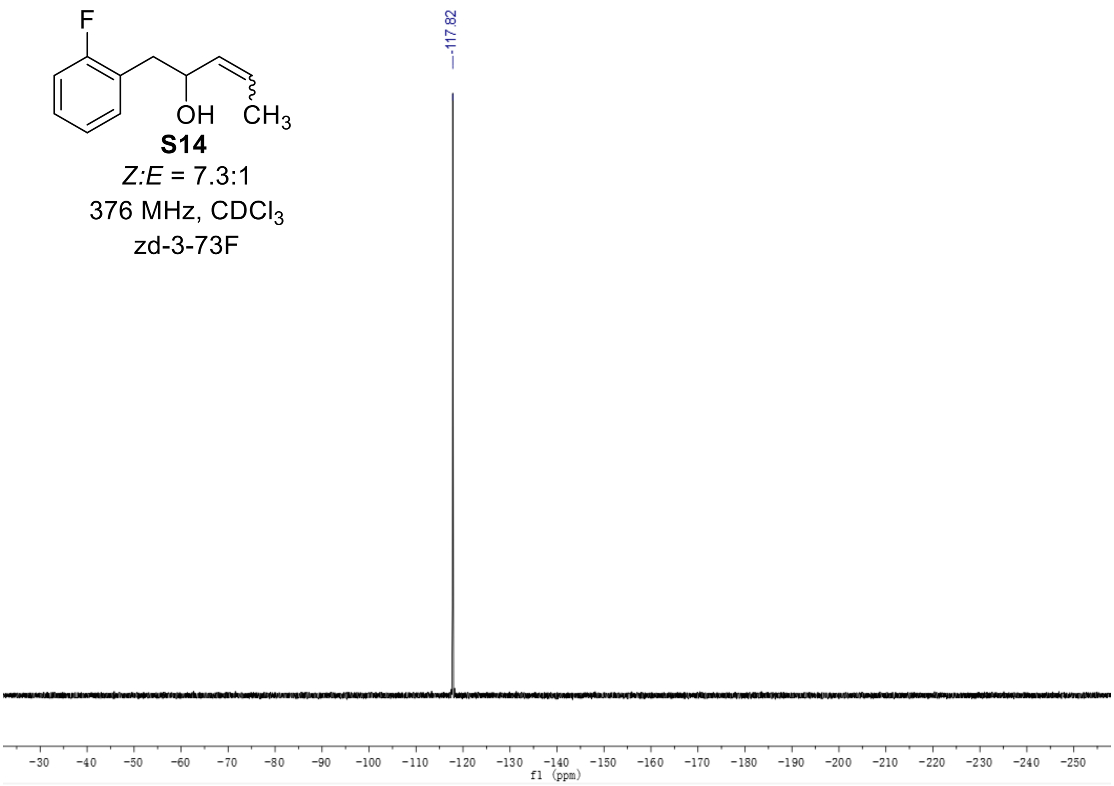

Supplementary Figure 61. ^{19}F NMR (376 MHz, $CDCl_3$, 25 °C) spectra for **S14**

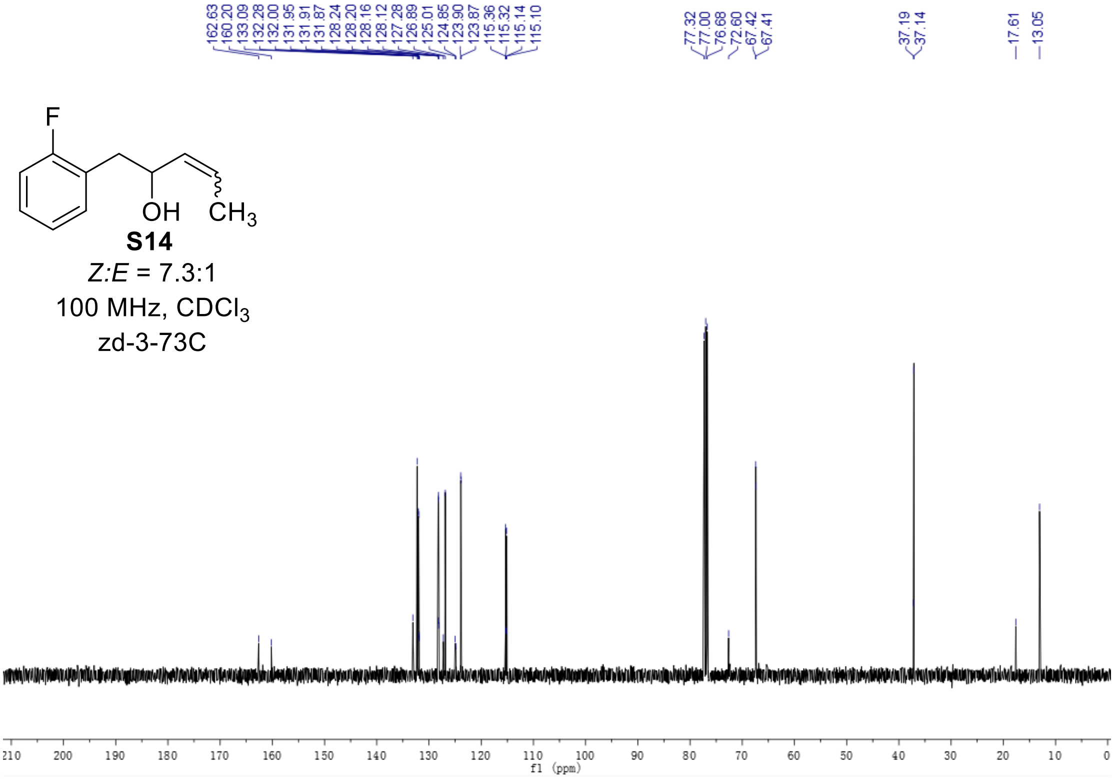

Supplementary Figure 62. ^{13}C NMR (100 MHz, $CDCl_3$, 25 °C) spectra for **S14**

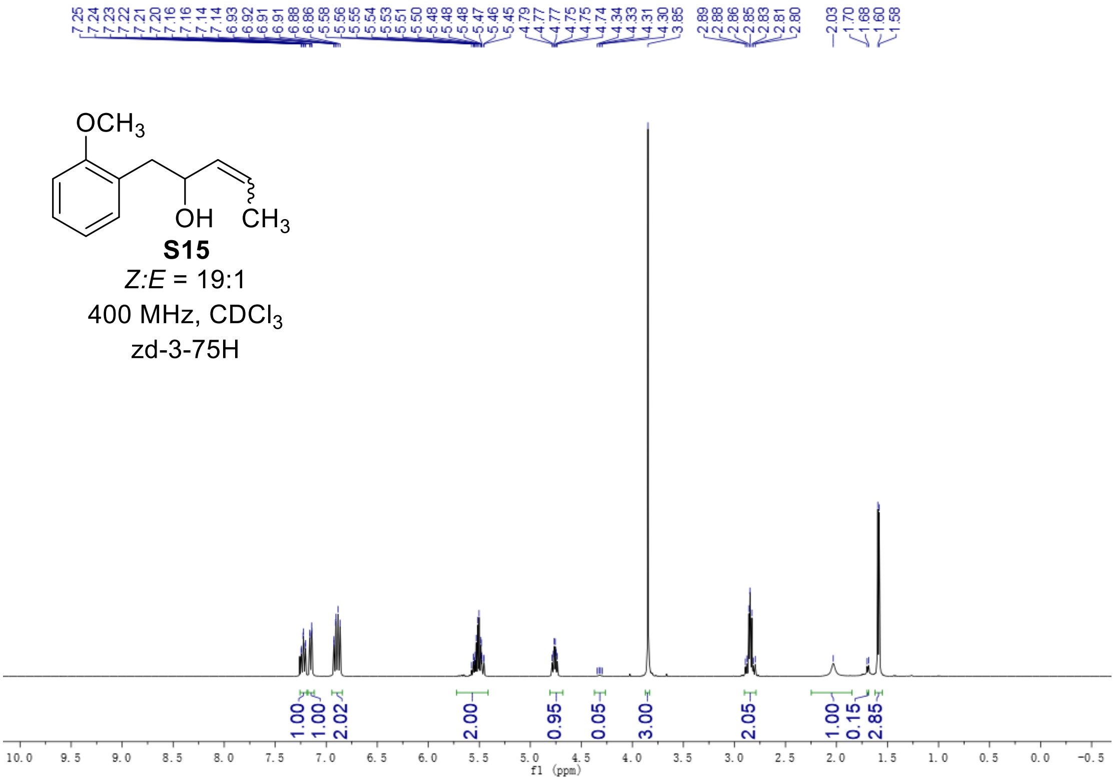

Supplementary Figure 63. ^{1}H NMR (400 MHz, $CDCl_3$, 25 °C) spectra for **S15**

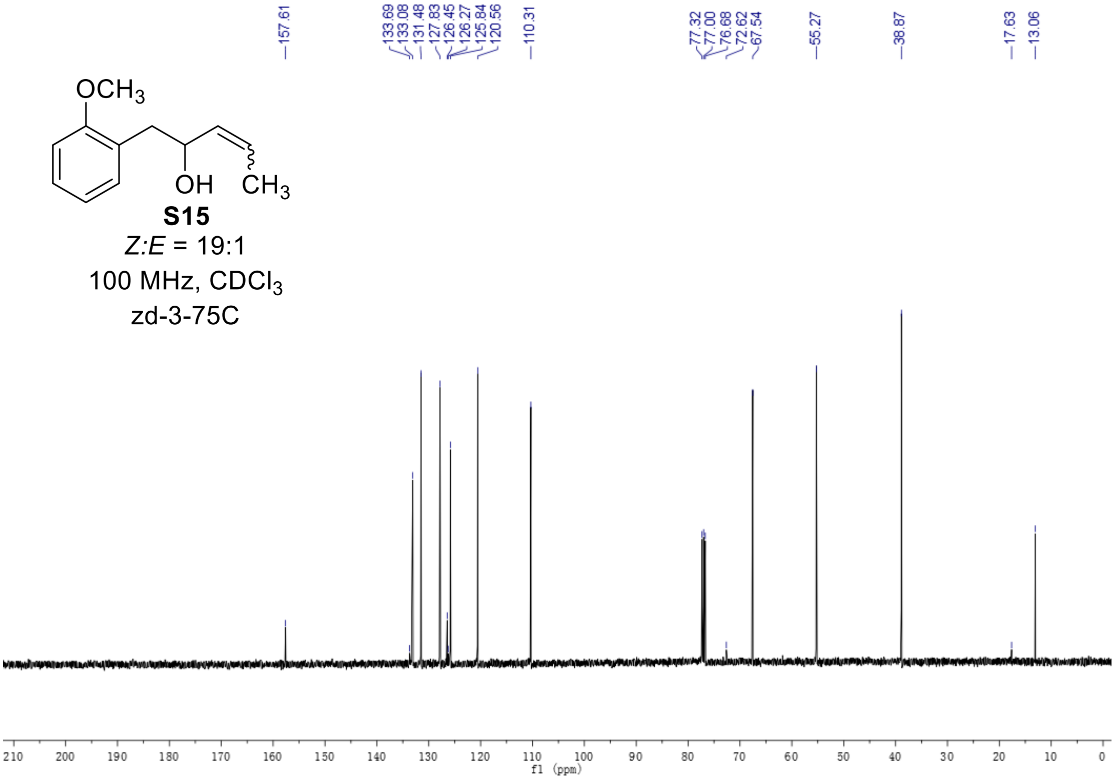

Supplementary Figure 64. ^{13}C NMR (100 MHz, $CDCl_3$, 25 °C) spectra for **S15**

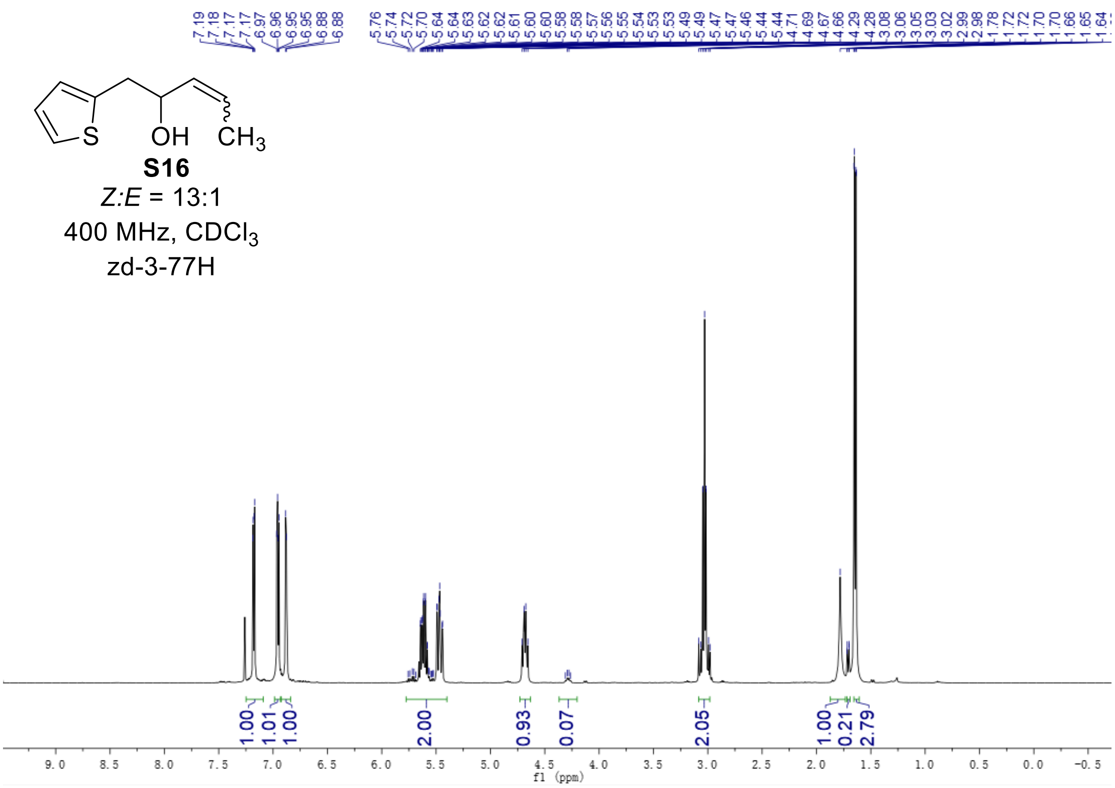

Supplementary Figure 65. ^{1}H NMR (400 MHz, $CDCl_3$, 25 °C) spectra for **S16**

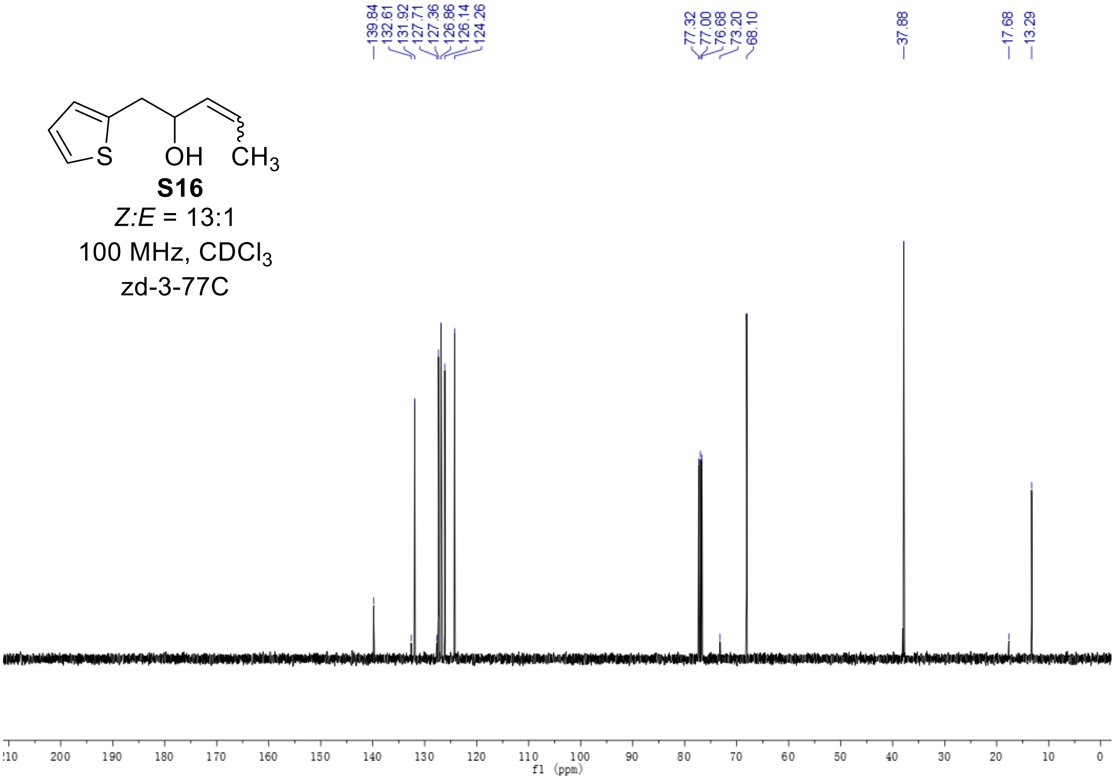

Supplementary Figure 66. ^{13}C NMR (100 MHz, $CDCl_3$, 25 °C) spectra for **S16**

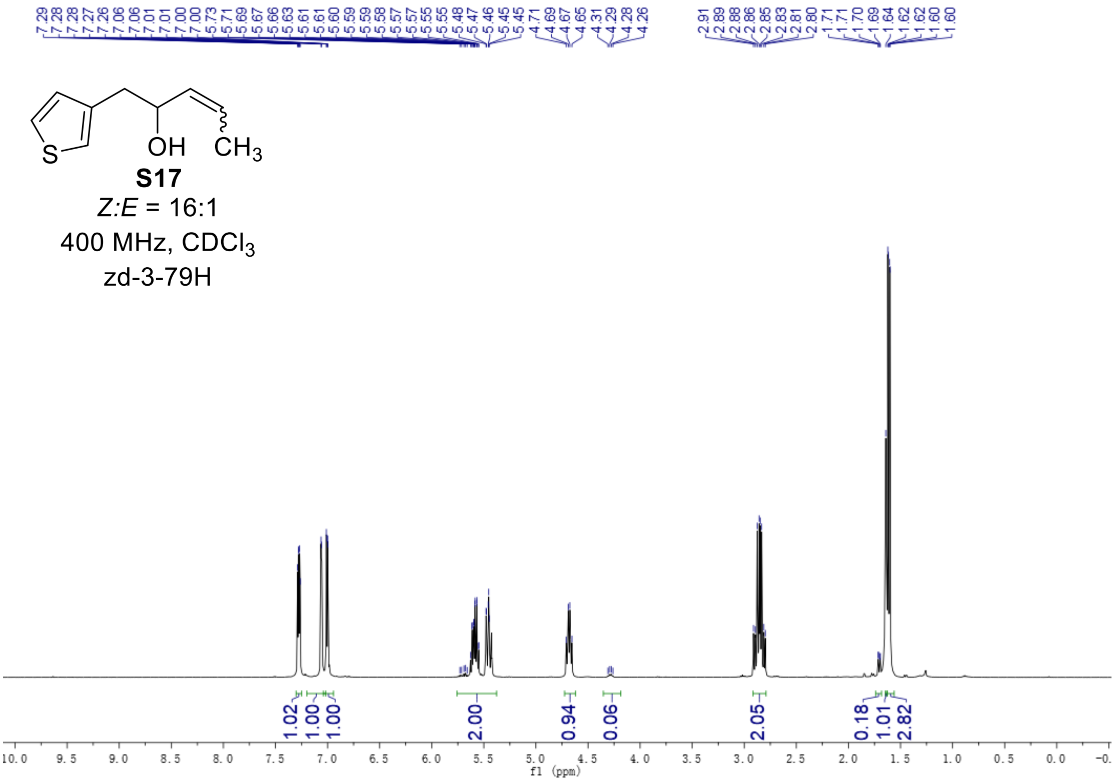

Supplementary Figure 67. ^{1}H NMR (400 MHz, $CDCl_3$, 25 °C) spectra for **S17**

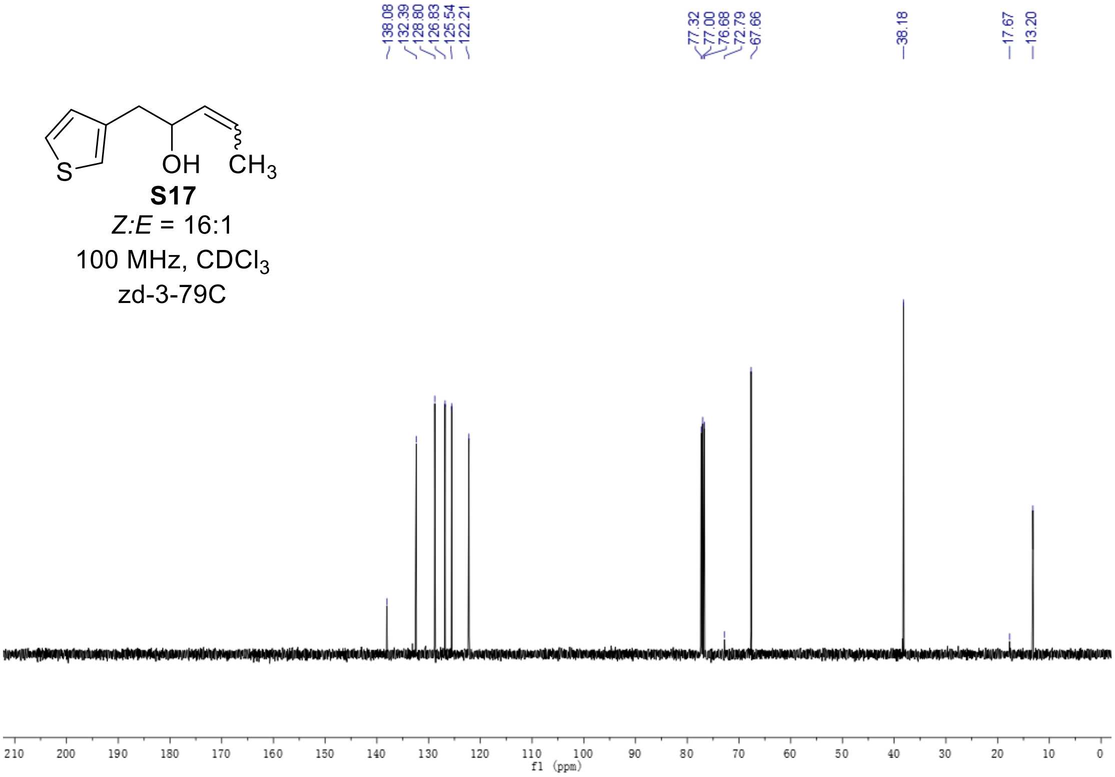

Supplementary Figure 68. ^{13}C NMR (100 MHz, $CDCl_3$, 25 °C) spectra for **S17**

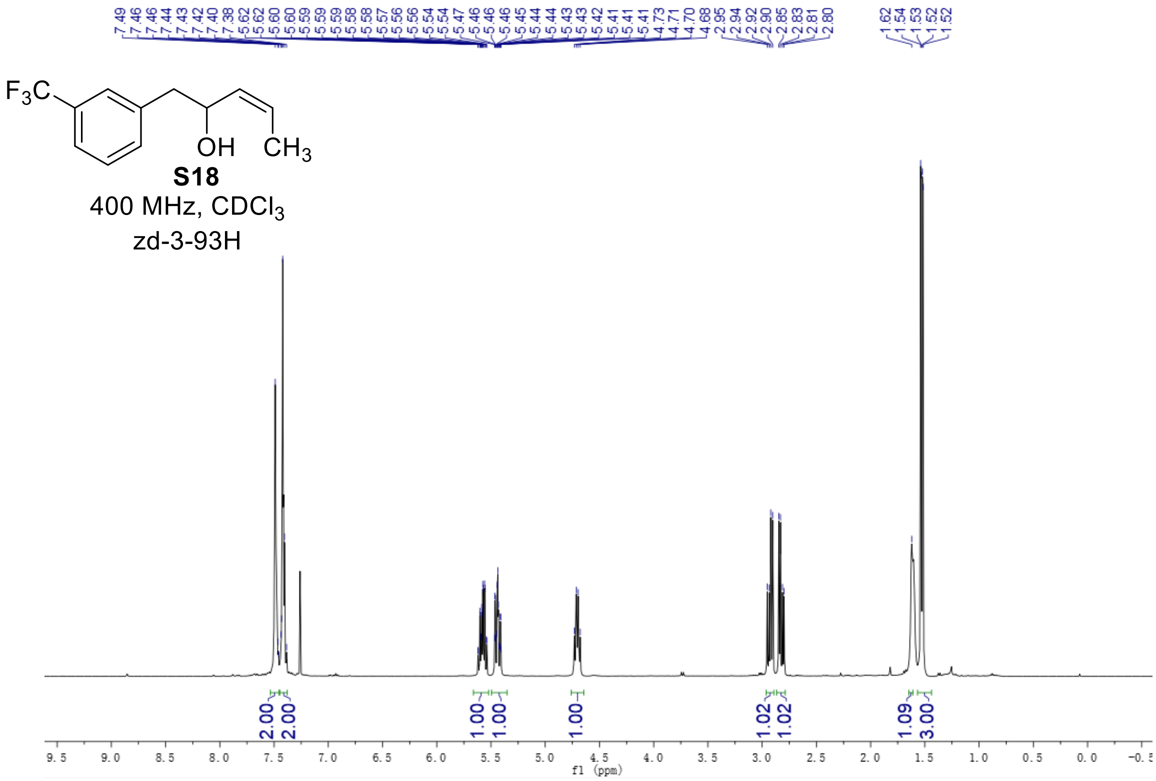

Supplementary Figure 69. ^{1}H NMR (400 MHz, $CDCl_3$, 25 °C) spectra for **S18**

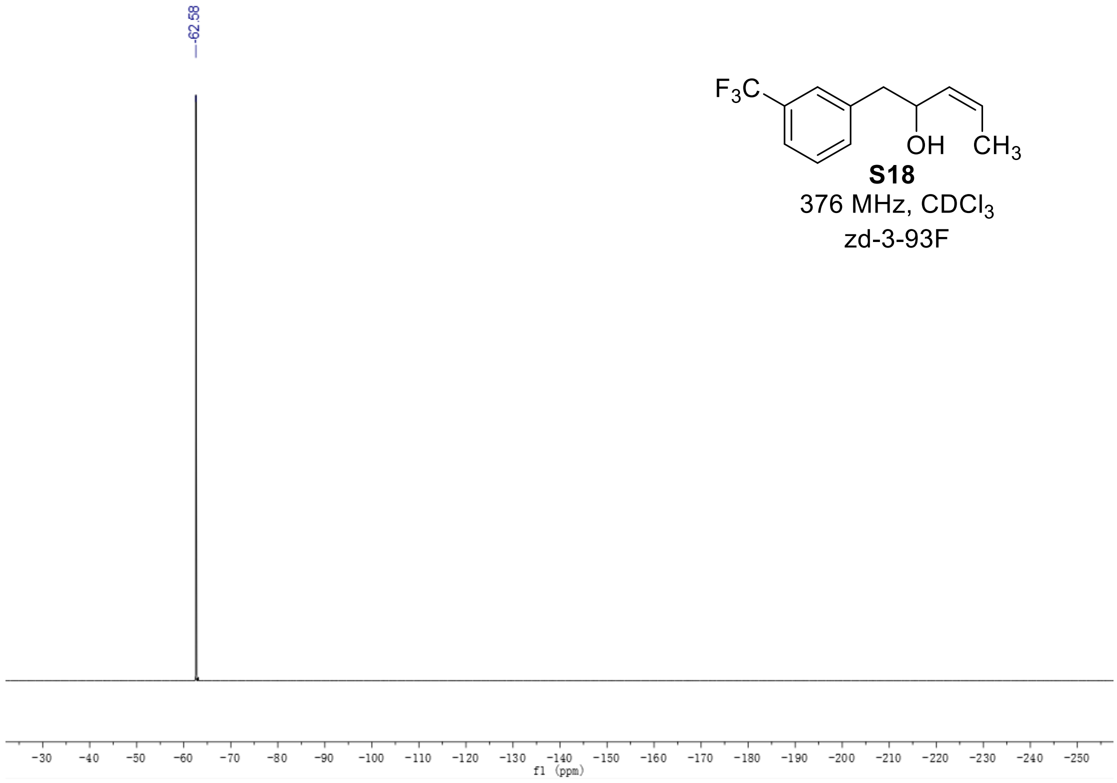

Supplementary Figure 70. ^{19}F NMR (376 MHz, $CDCl_3$, 25 °C) spectra for **S8**

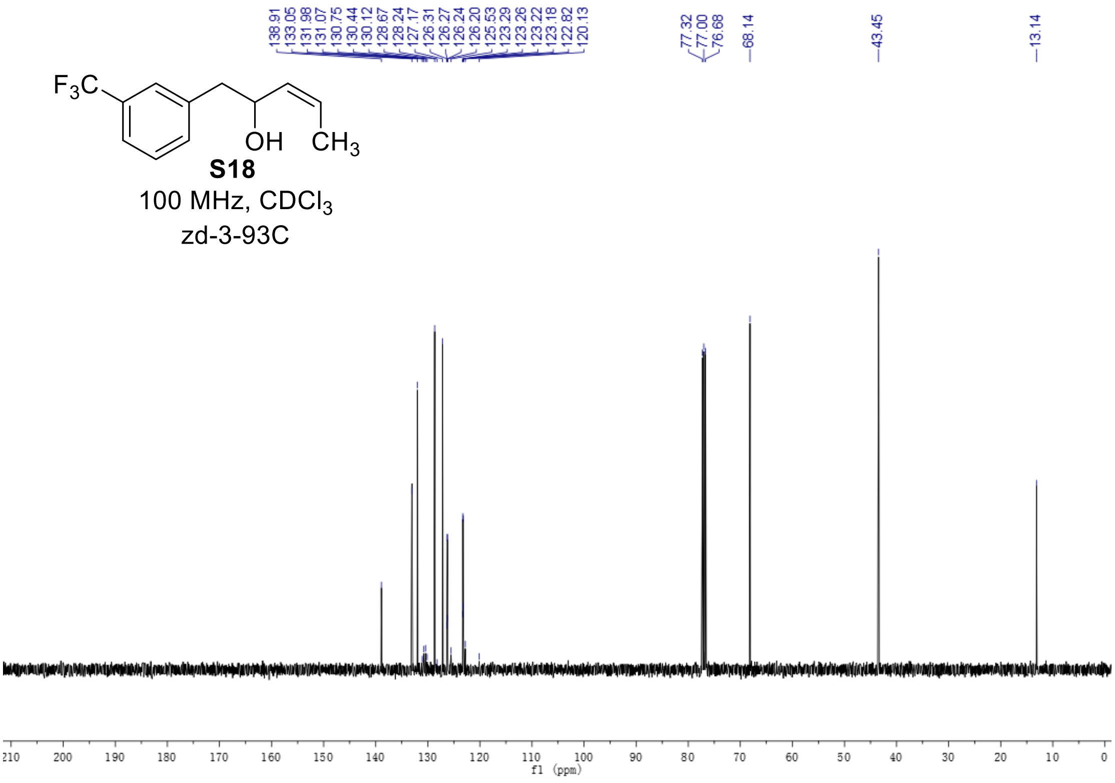

Supplementary Figure 71. ^{13}C NMR (100 MHz, $CDCl_3$, 25 °C) spectra for **S18**

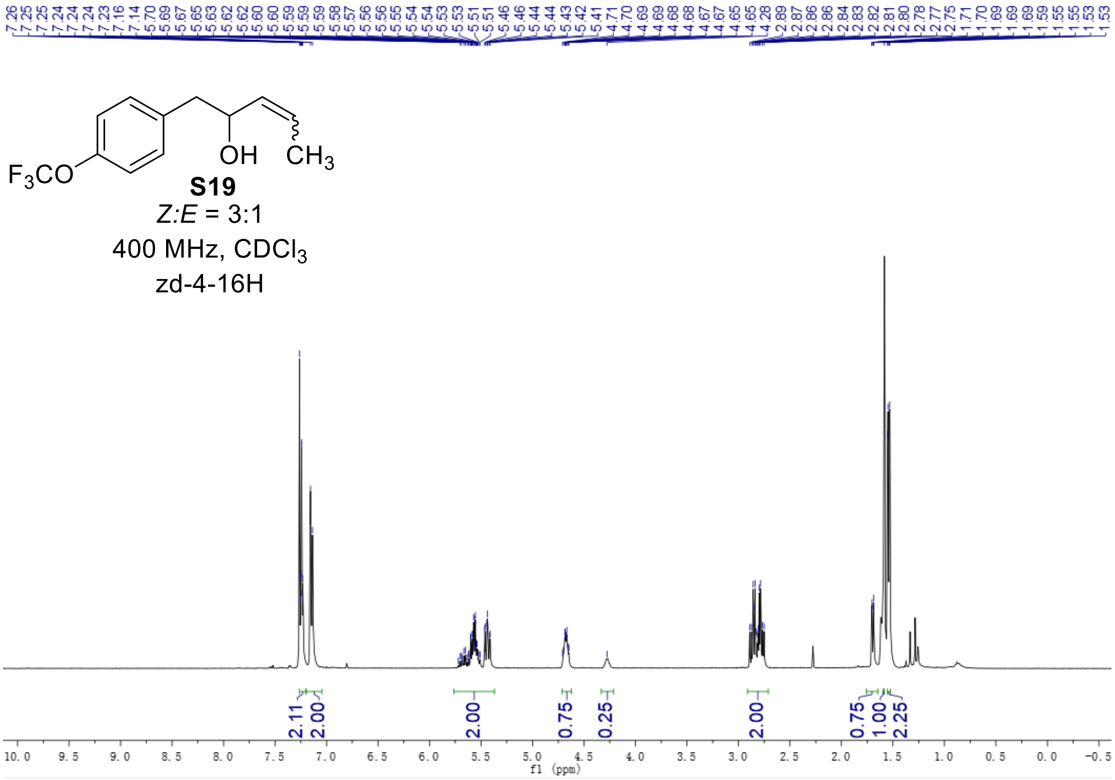

Supplementary Figure 72. ^{1}H NMR (400 MHz, $CDCl_3$, 25 °C) spectra for **S19**

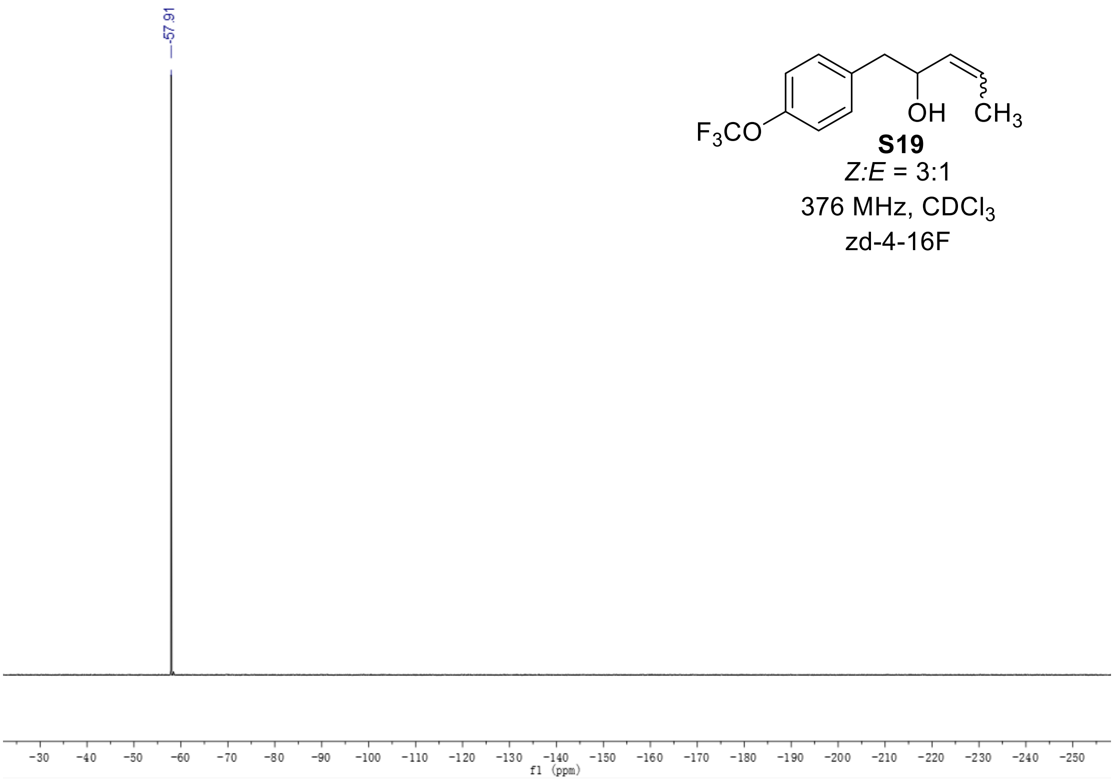

Supplementary Figure 73. ^{19}F NMR (376 MHz, $CDCl_3$, 25 °C) spectra for **S19**

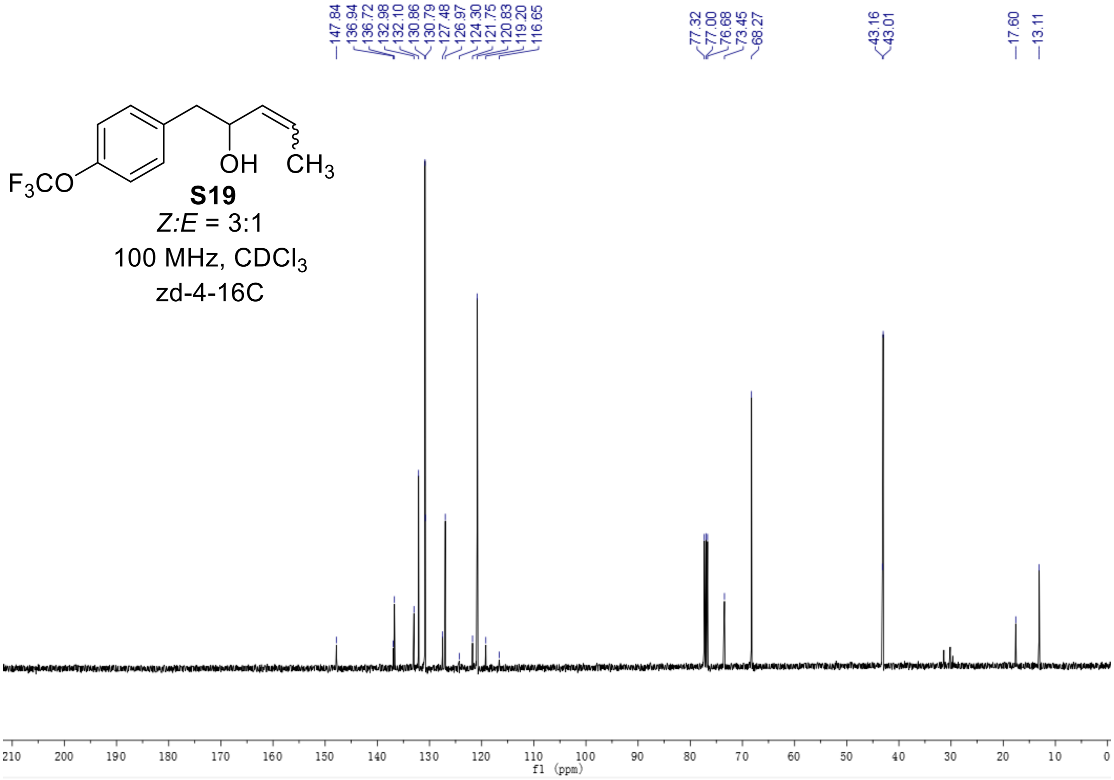

Supplementary Figure 74. ^{13}C NMR (100 MHz, $CDCl_3$, 25 °C) spectra for **S19**

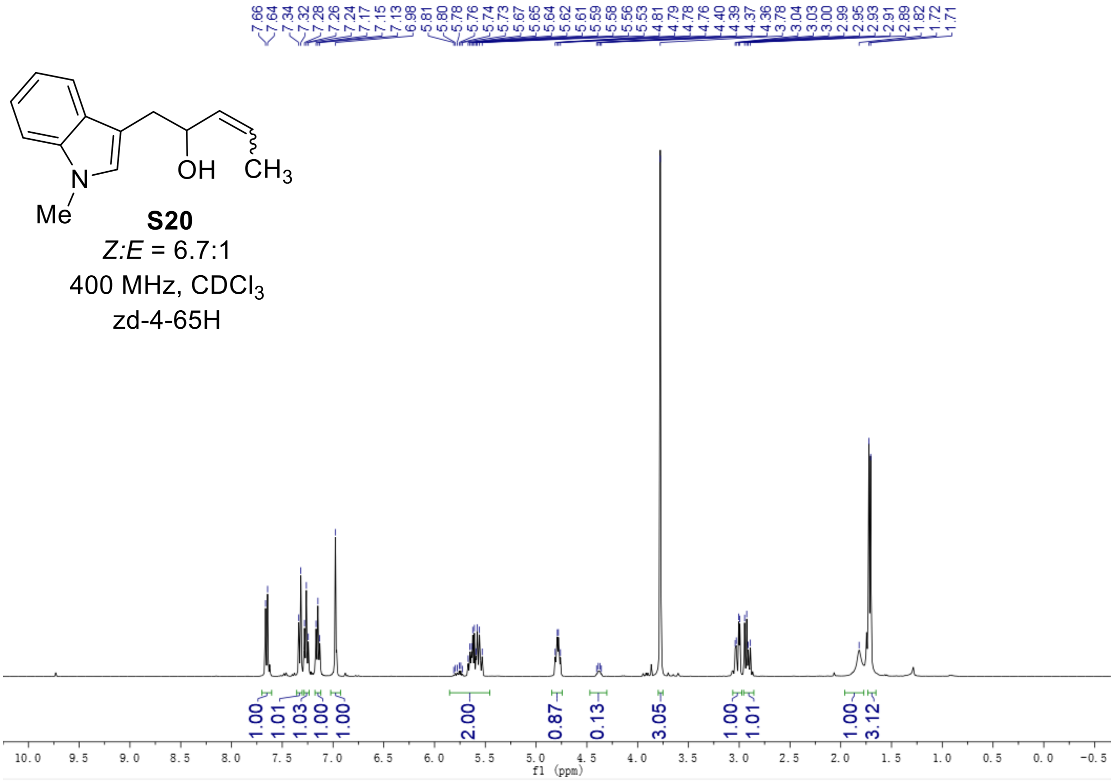

Supplementary Figure 75. ^{1}H NMR (400 MHz, $CDCl_3$, 25 °C) spectra for **S20**

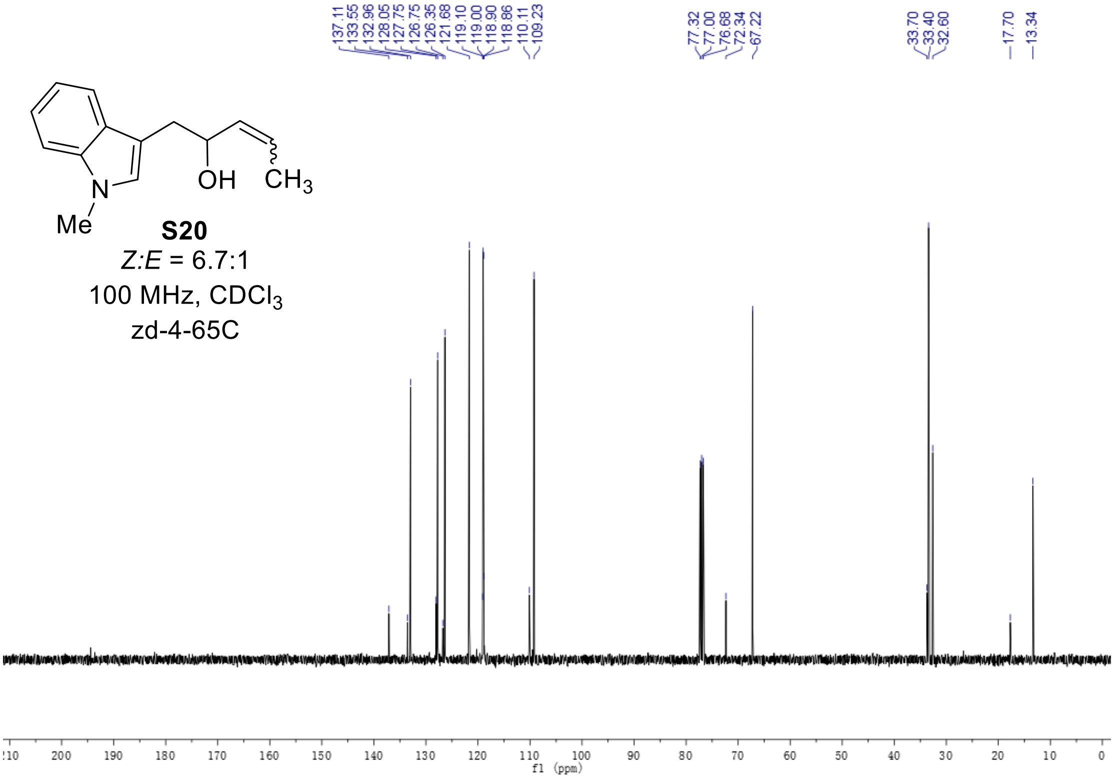

Supplementary Figure 76. ^{13}C NMR (100 MHz, $CDCl_3$, 25 °C) spectra for **S20**

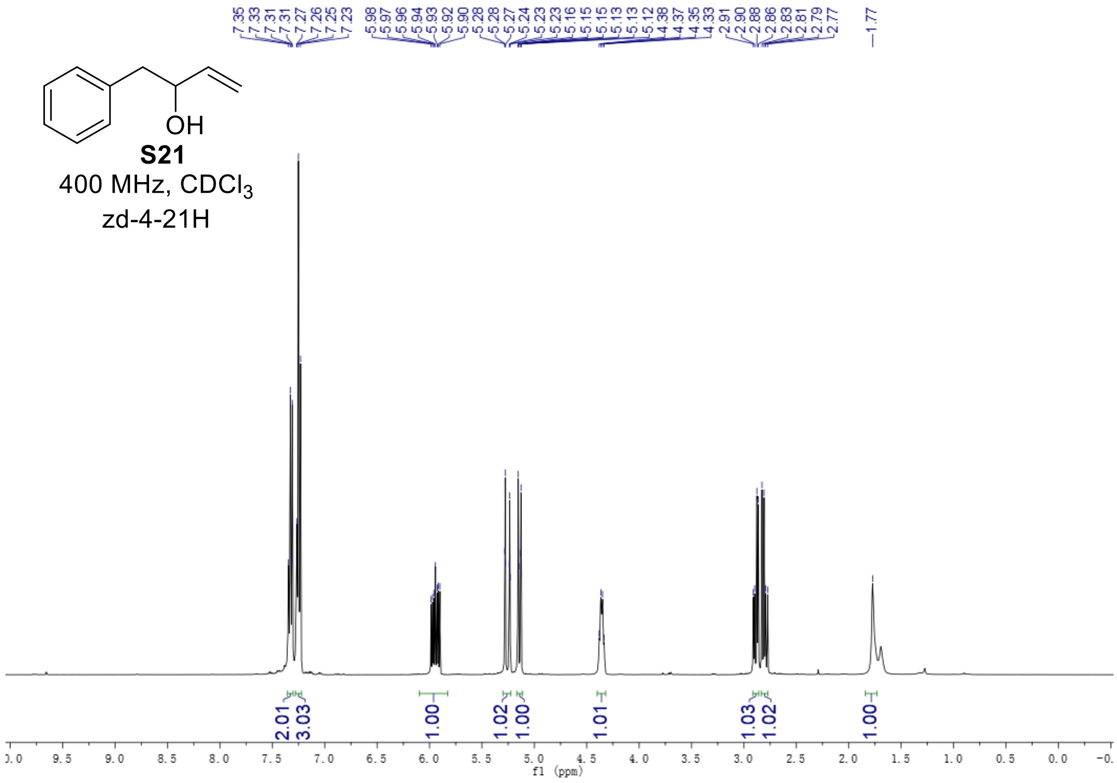

Supplementary Figure 77. ^{1}H NMR (400 MHz, $CDCl_3$, 25 °C) spectra for **S21**

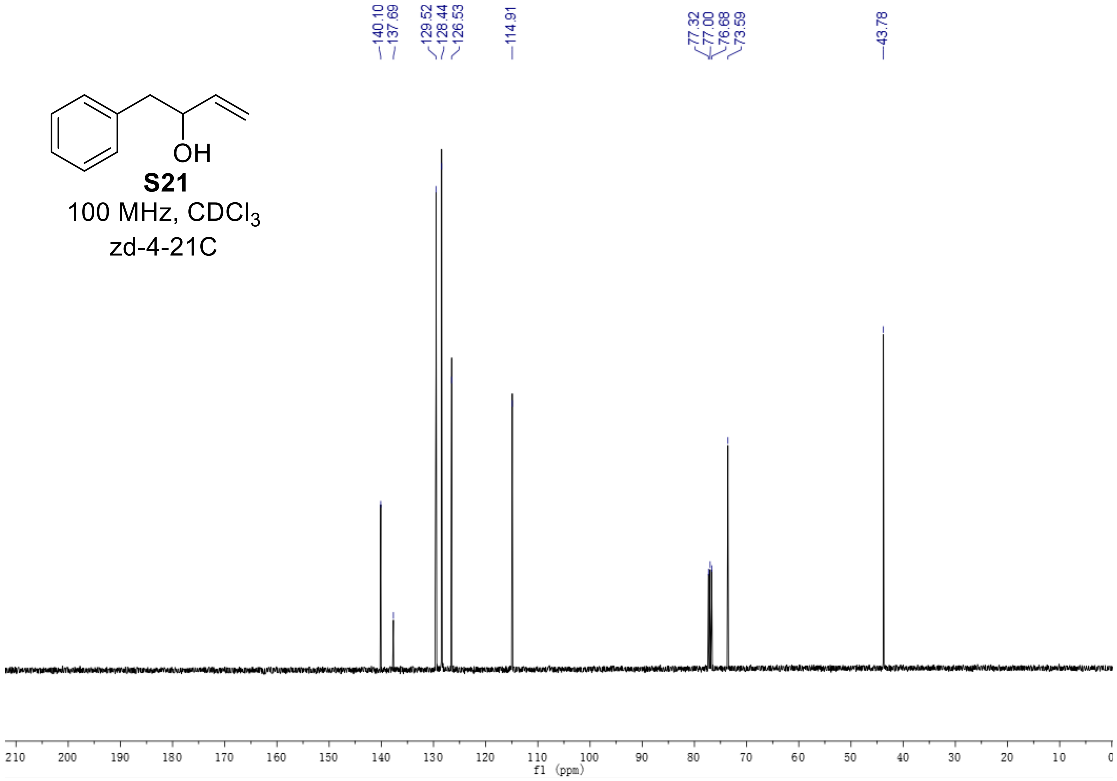

Supplementary Figure 78. ^{13}C NMR (100 MHz, $CDCl_3$, 25 °C) spectra for **S21**

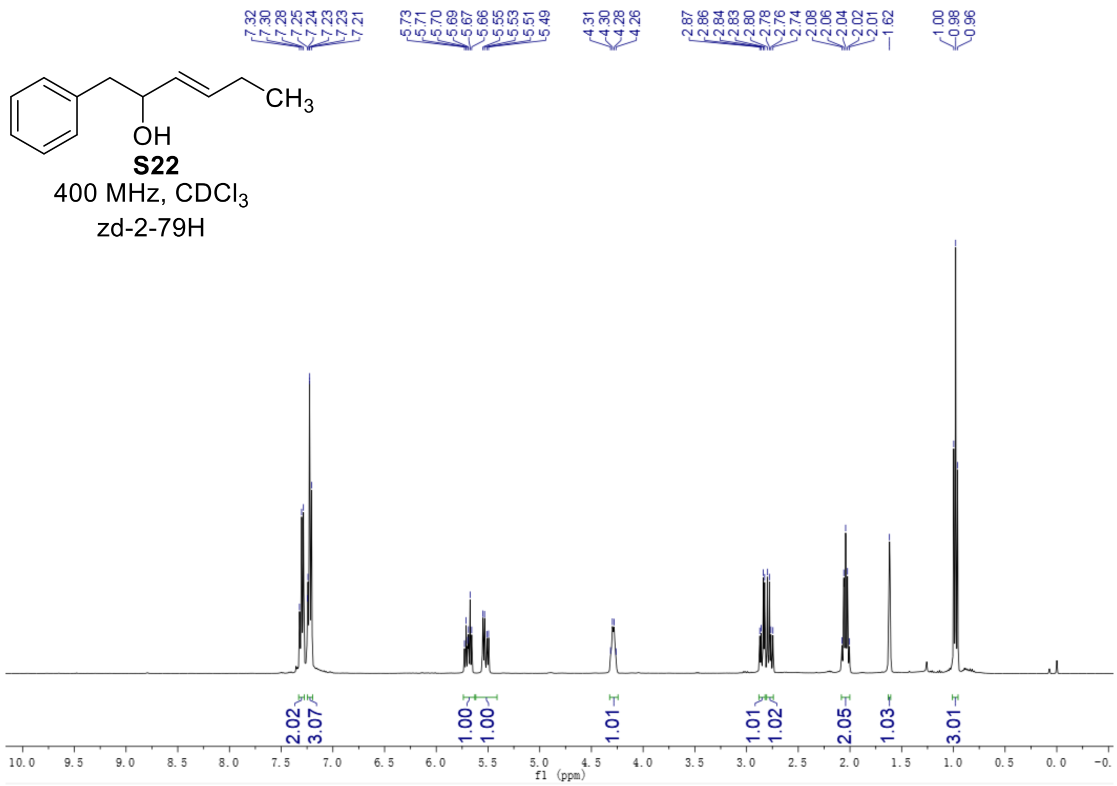

Supplementary Figure 79. ^{1}H NMR (400 MHz, $CDCl_3$, 25 °C) spectra for **S22**

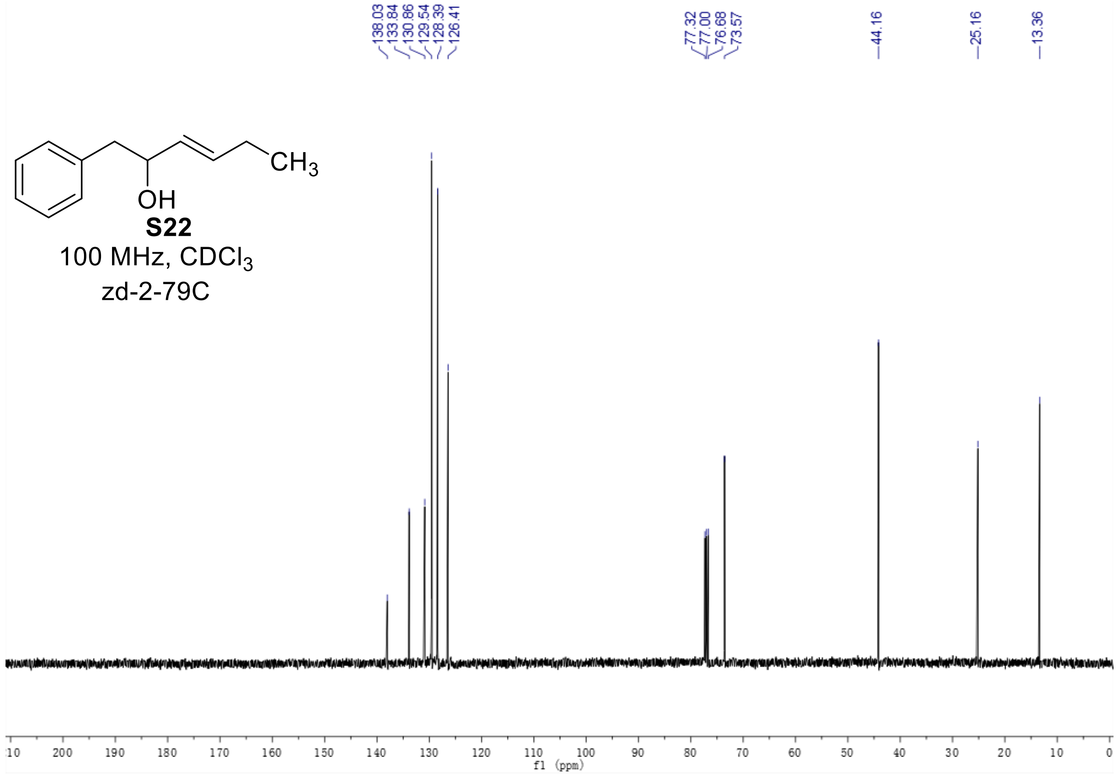

Supplementary Figure 80. ^{13}C NMR (100 MHz, $CDCl_3$, 25 °C) spectra for **S22**

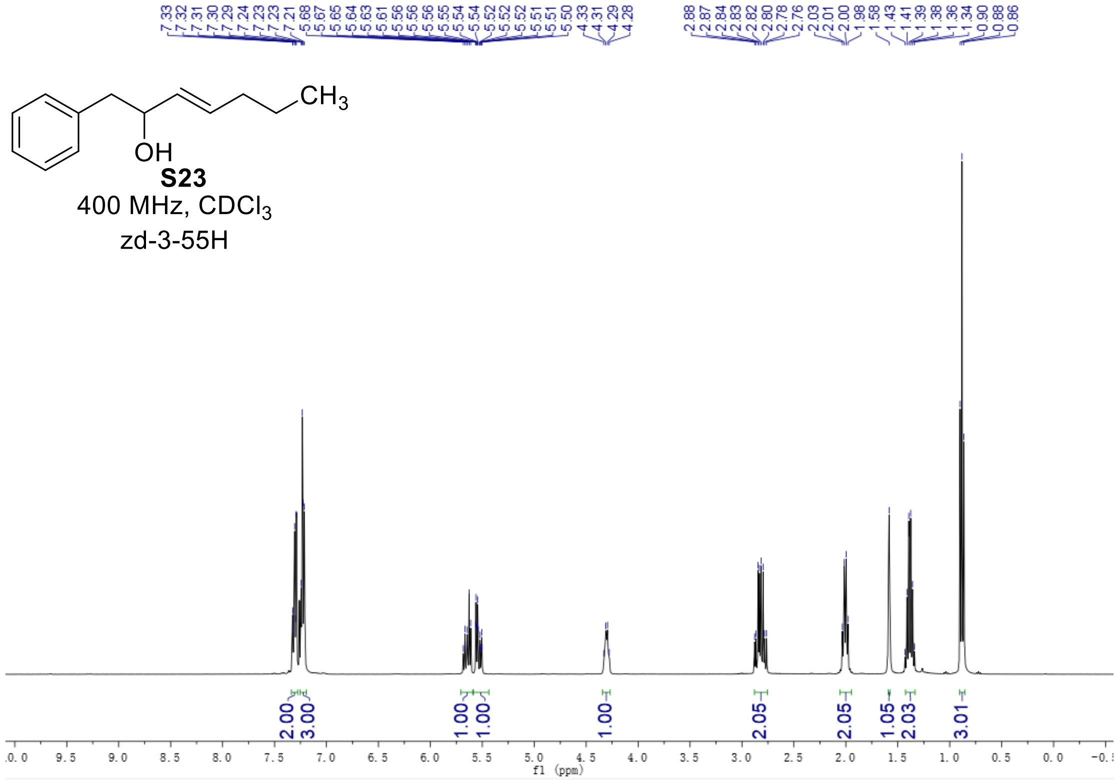

Supplementary Figure 81. ^{1}H NMR (400 MHz, $CDCl_3$, 25 °C) spectra for **S23**

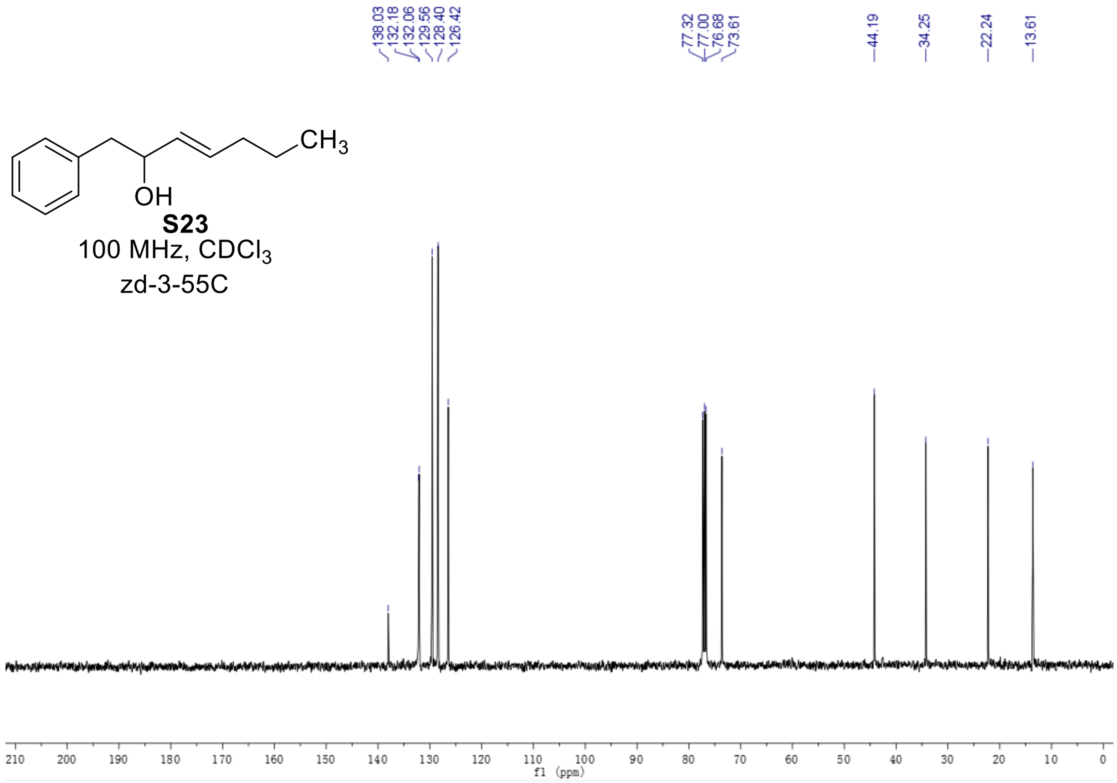

Supplementary Figure 82. ^{13}C NMR (100 MHz, $CDCl_3$, 25 °C) spectra for **S23**

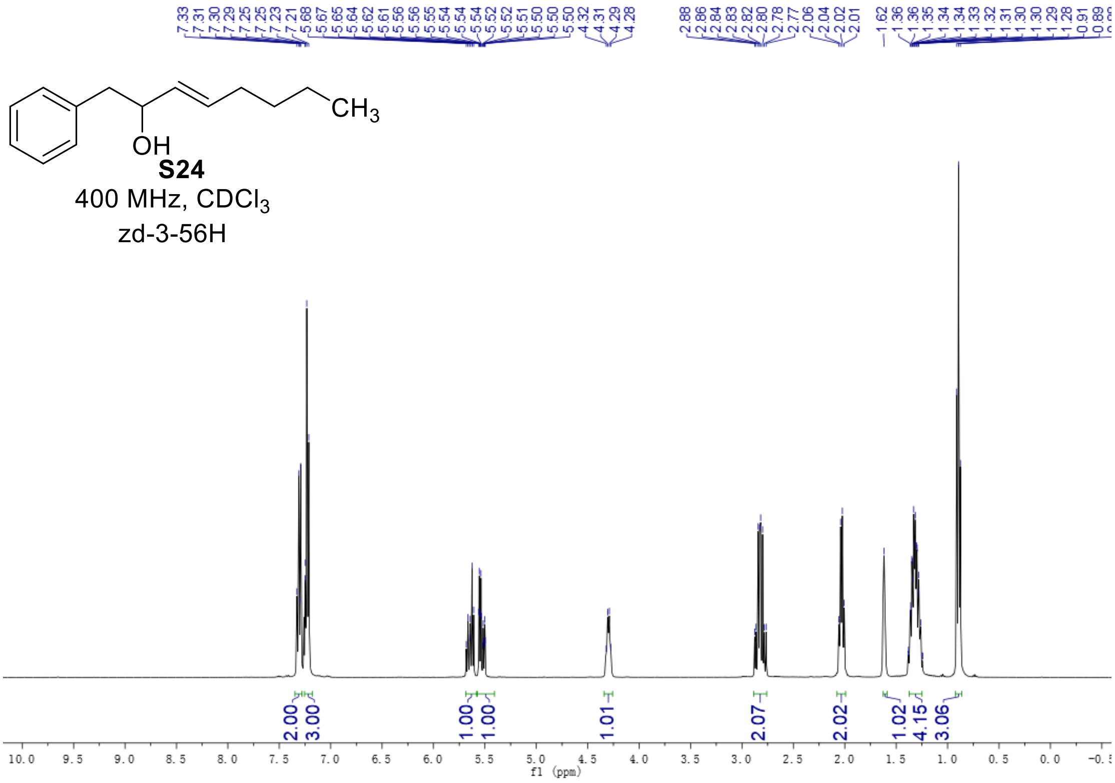

Supplementary Figure 83. ^{1}H NMR (400 MHz, $CDCl_3$, 25 °C) spectra for **S24**

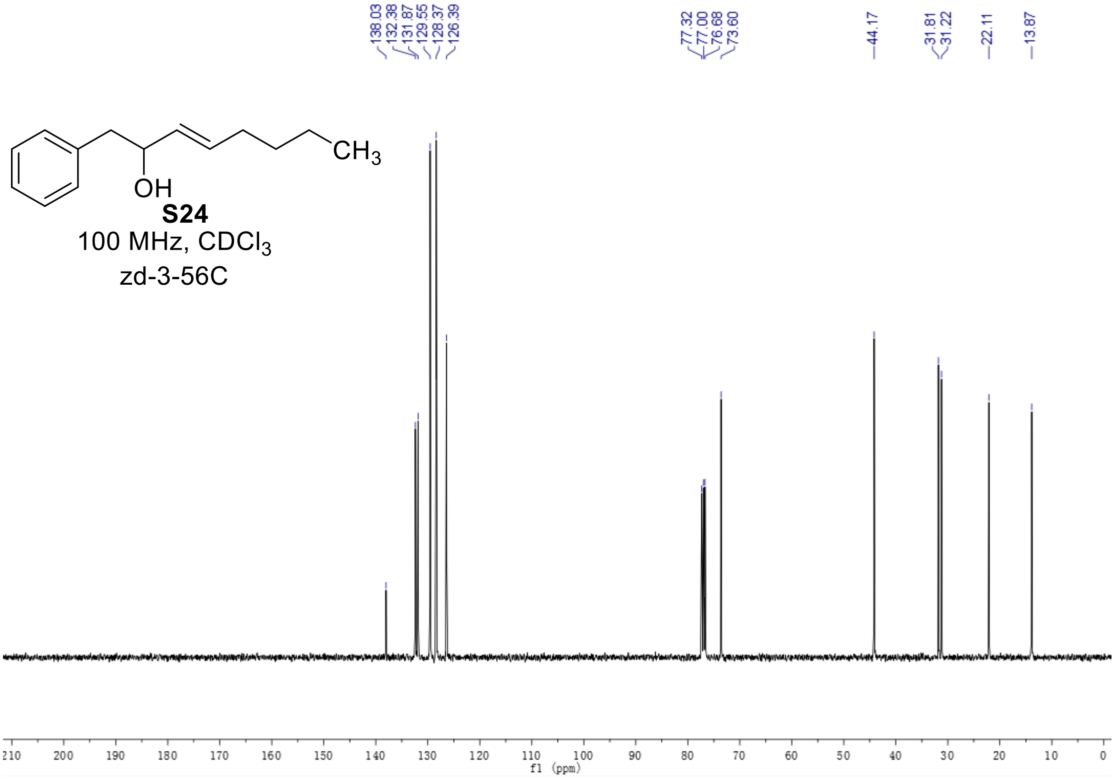

Supplementary Figure 84. ^{13}C NMR (100 MHz, $CDCl_3$, 25 °C) spectra for **S24**

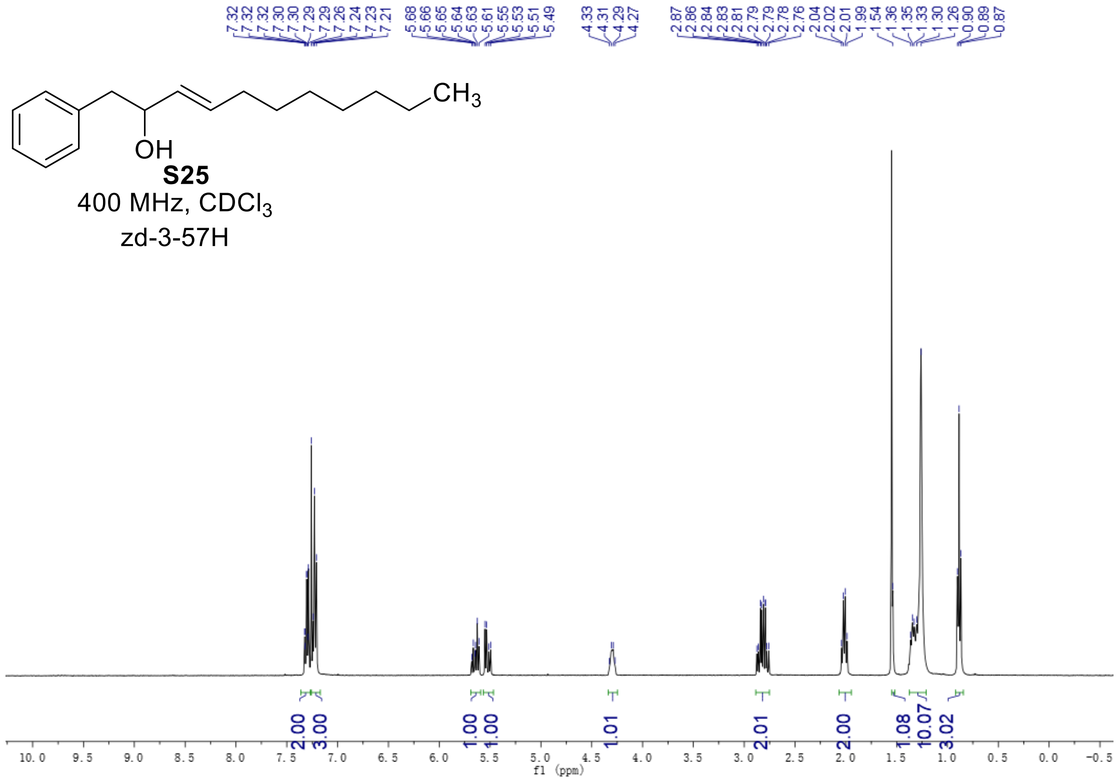

Supplementary Figure 85. ^{1}H NMR (400 MHz, $CDCl_3$, 25 °C) spectra for **S25**

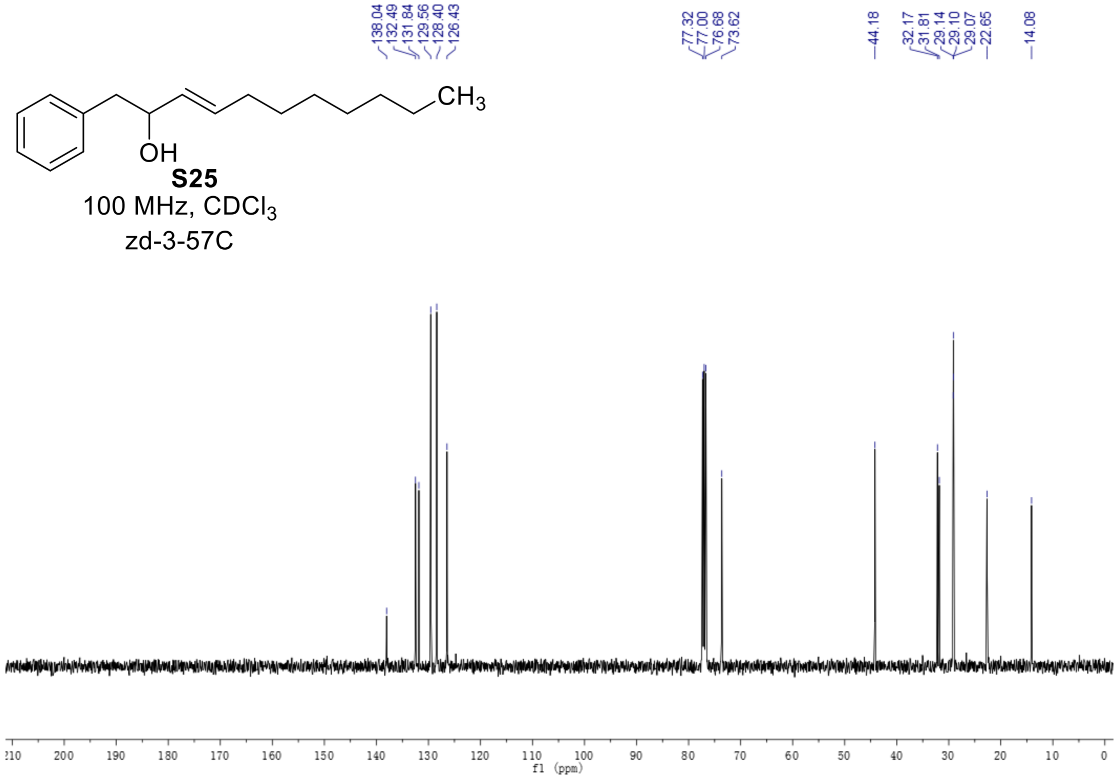

Supplementary Figure 86. ^{13}C NMR (100 MHz, $CDCl_3$, 25 °C) spectra for **S25**

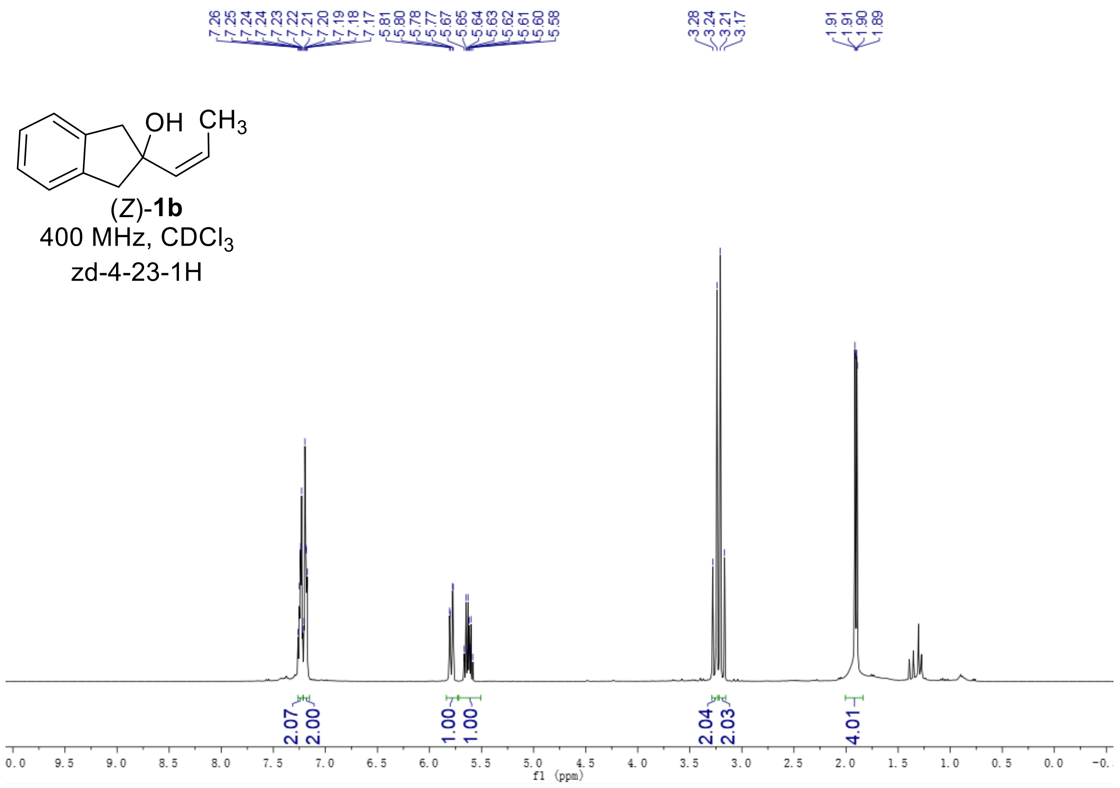

Supplementary Figure 87. ^{1}H NMR (400 MHz, $CDCl_3$, 25 °C) spectra for (*Z*)-**1b**

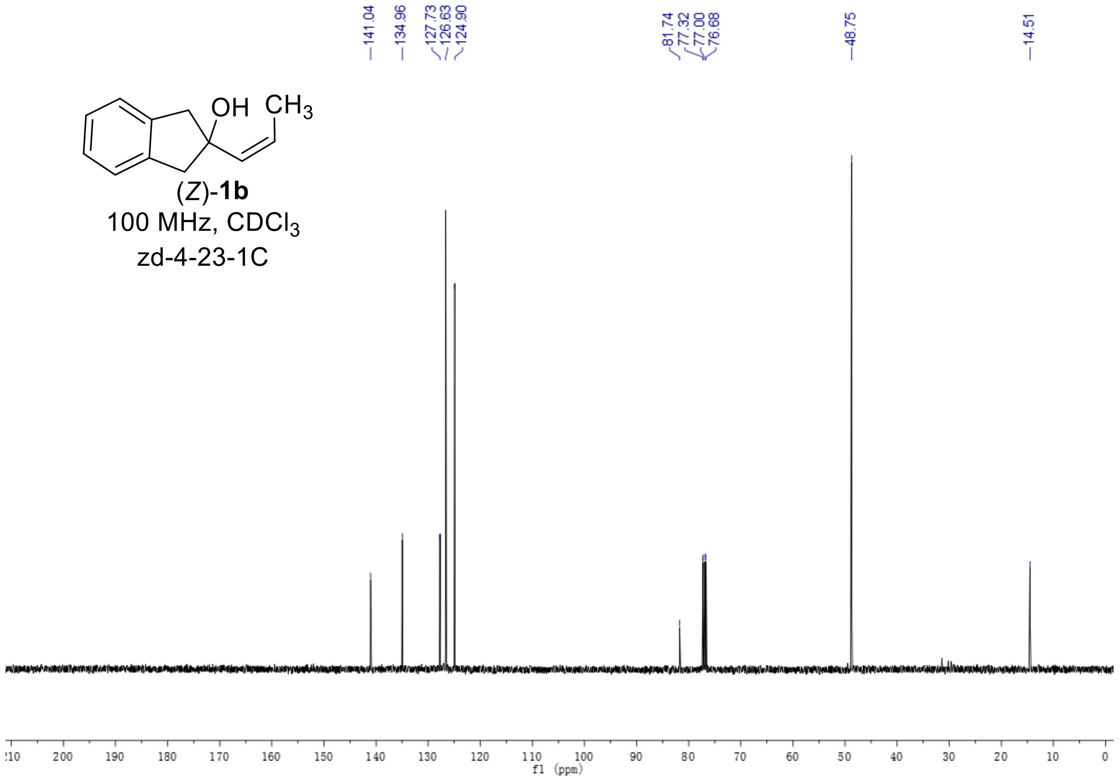

Supplementary Figure 88. ^{13}C NMR (100 MHz, $CDCl_3$, 25 °C) spectra for (*Z*)-**1b**

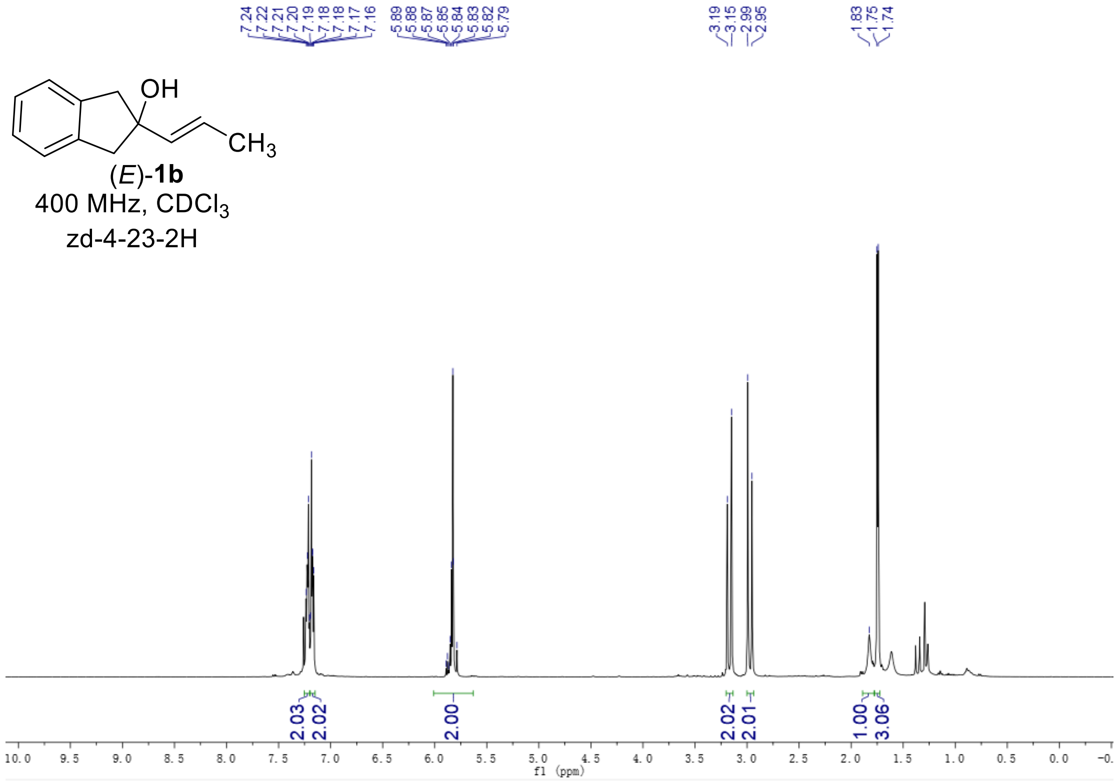

Supplementary Figure 89. ^{1}H NMR (400 MHz, $CDCl_3$, 25 °C) spectra for (*E*)**-1b**

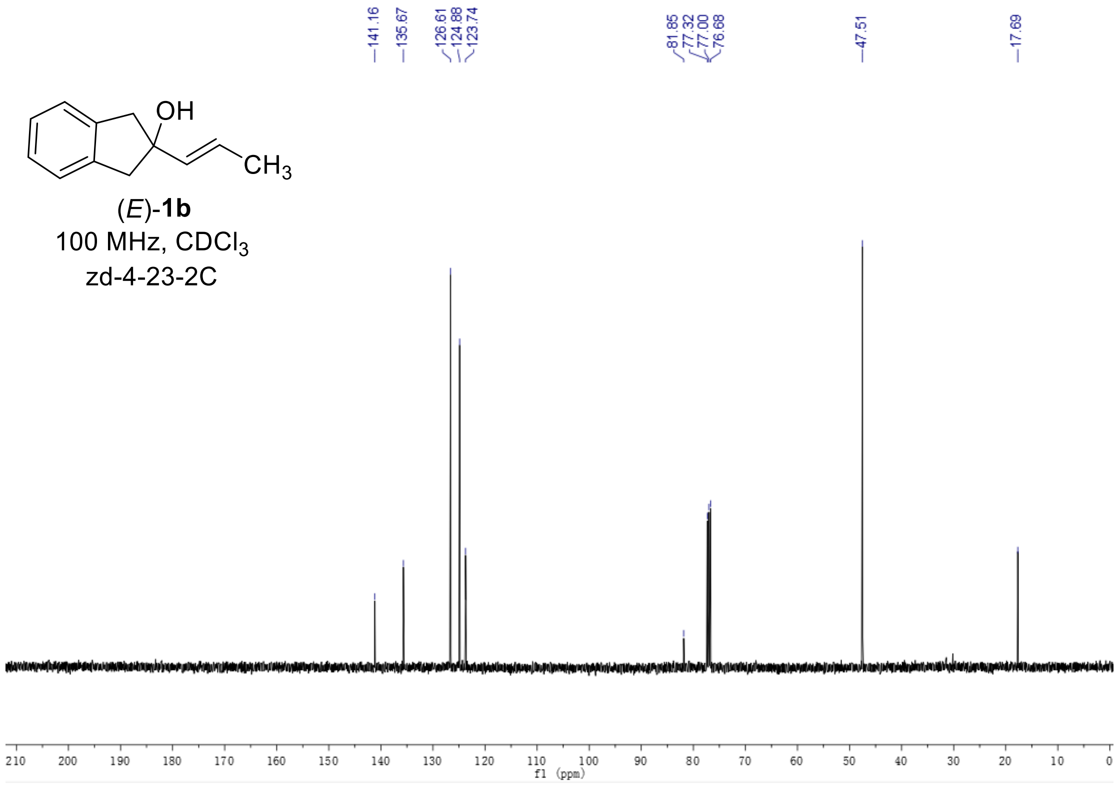

Supplementary Figure 90. ^{13}C NMR (100 MHz, $CDCl_3$, 25 °C) spectra for (*E*)**-1b**

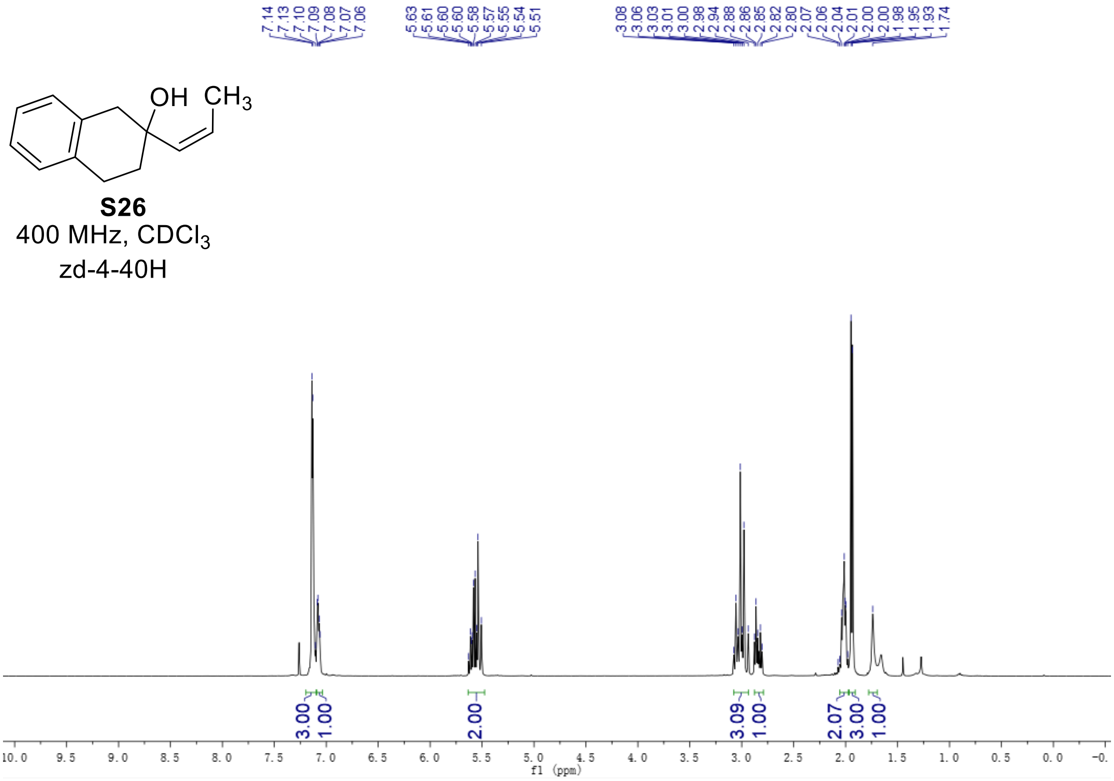

Supplementary Figure 91. ^{1}H NMR (400 MHz, $CDCl_3$, 25 °C) spectra for **S26**

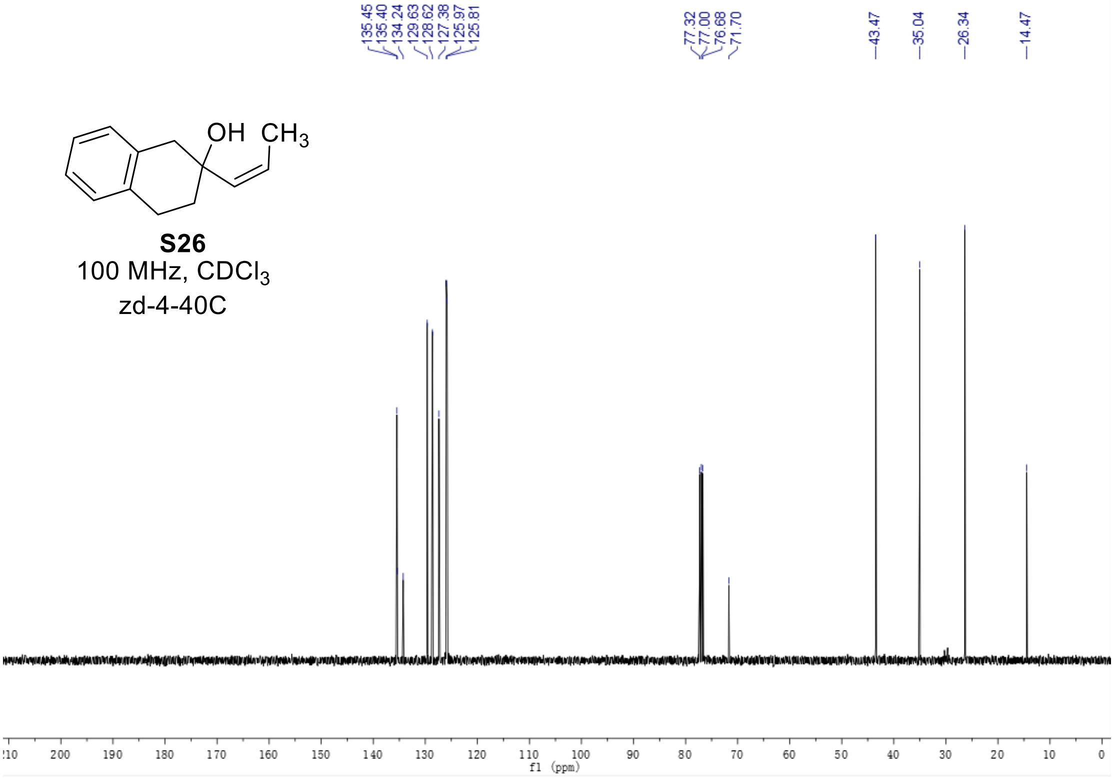

Supplementary Figure 92. ^{13}C NMR (100 MHz, $CDCl_3$, 25 °C) spectra for **S26**

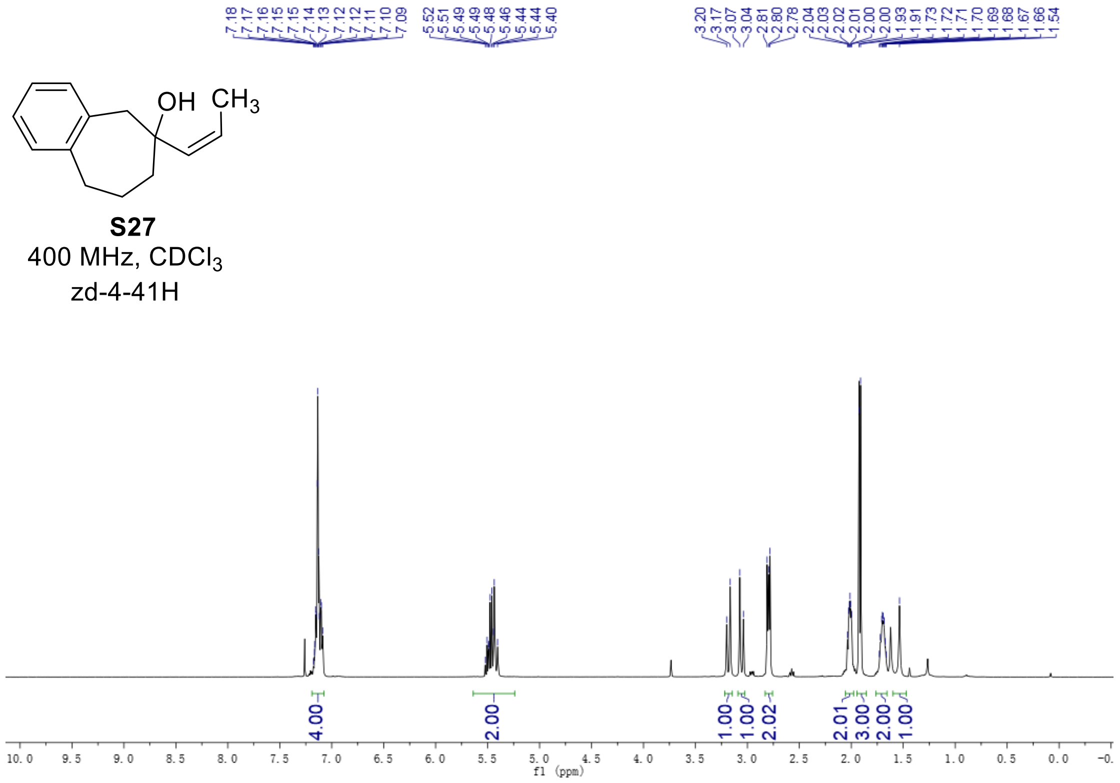

Supplementary Figure 93. ^{1}H NMR (400 MHz, $CDCl_3$, 25 °C) spectra for **S27**

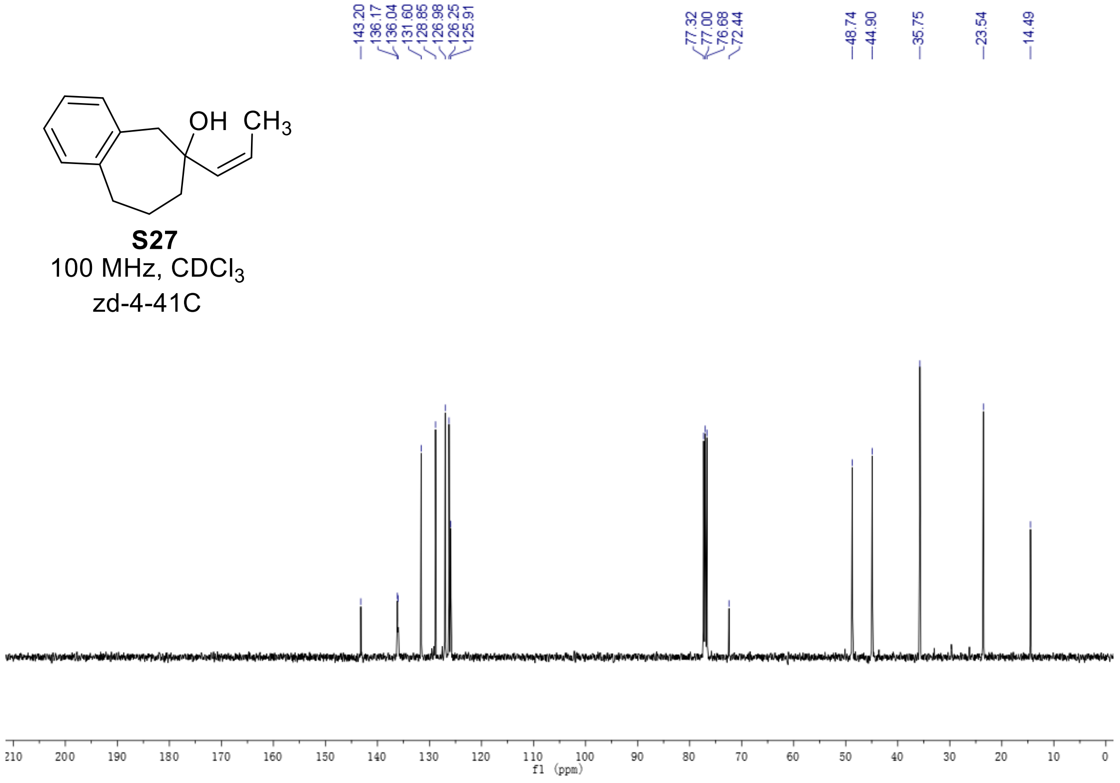

Supplementary Figure 94. ^{13}C NMR (100 MHz, $CDCl_3$, 25 °C) spectra for **S27**

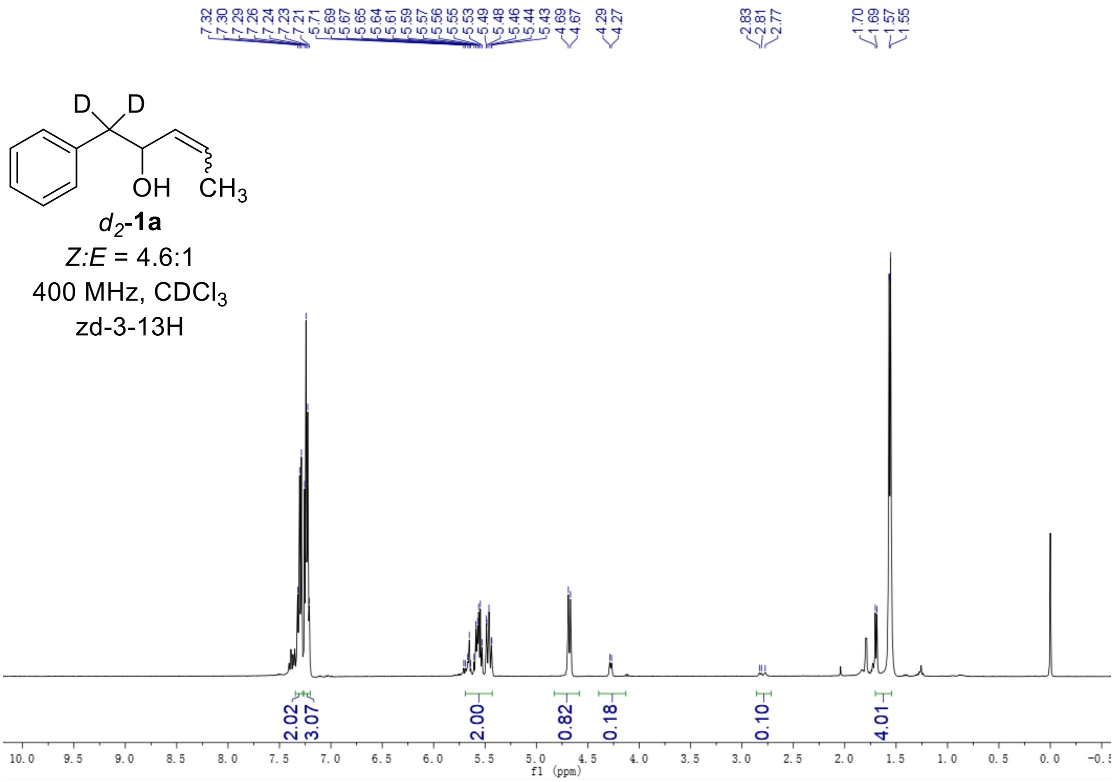

Supplementary Figure 95. ^{1}H NMR (400 MHz, $CDCl_3$, 25 °C) spectra for d_2-**1a**

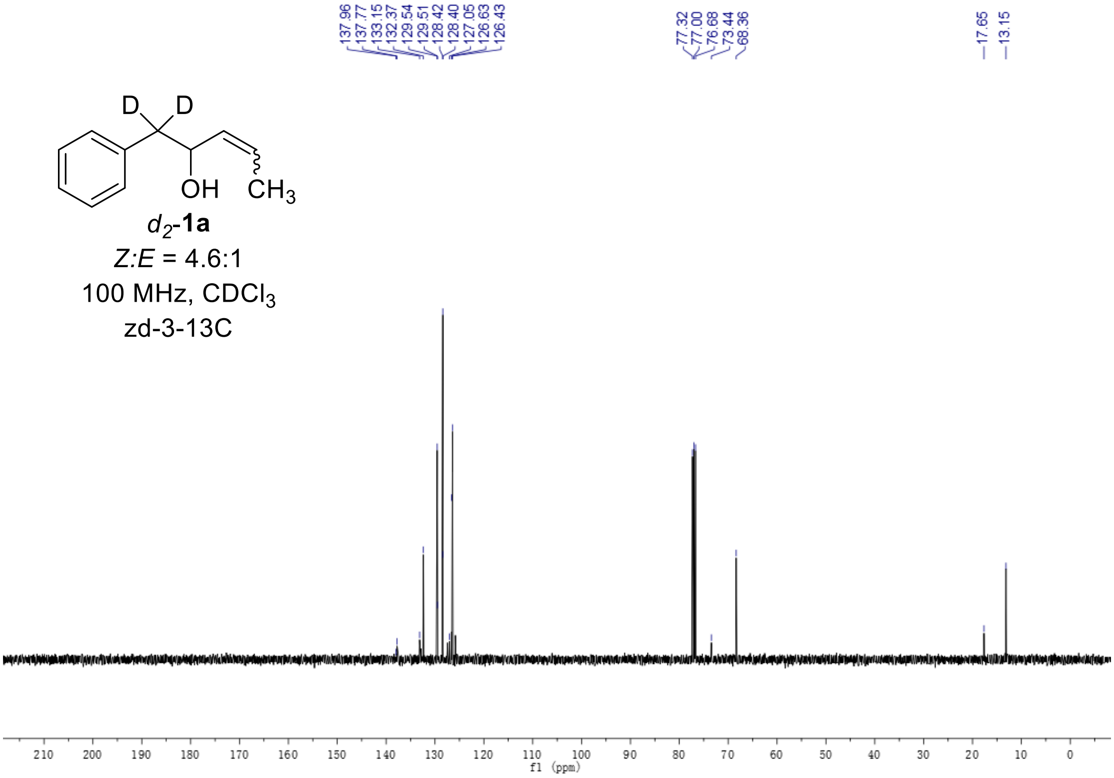

Supplementary Figure 96. ^{13}C NMR (100 MHz, $CDCl_3$, 25 °C) spectra for d_2-**1a**

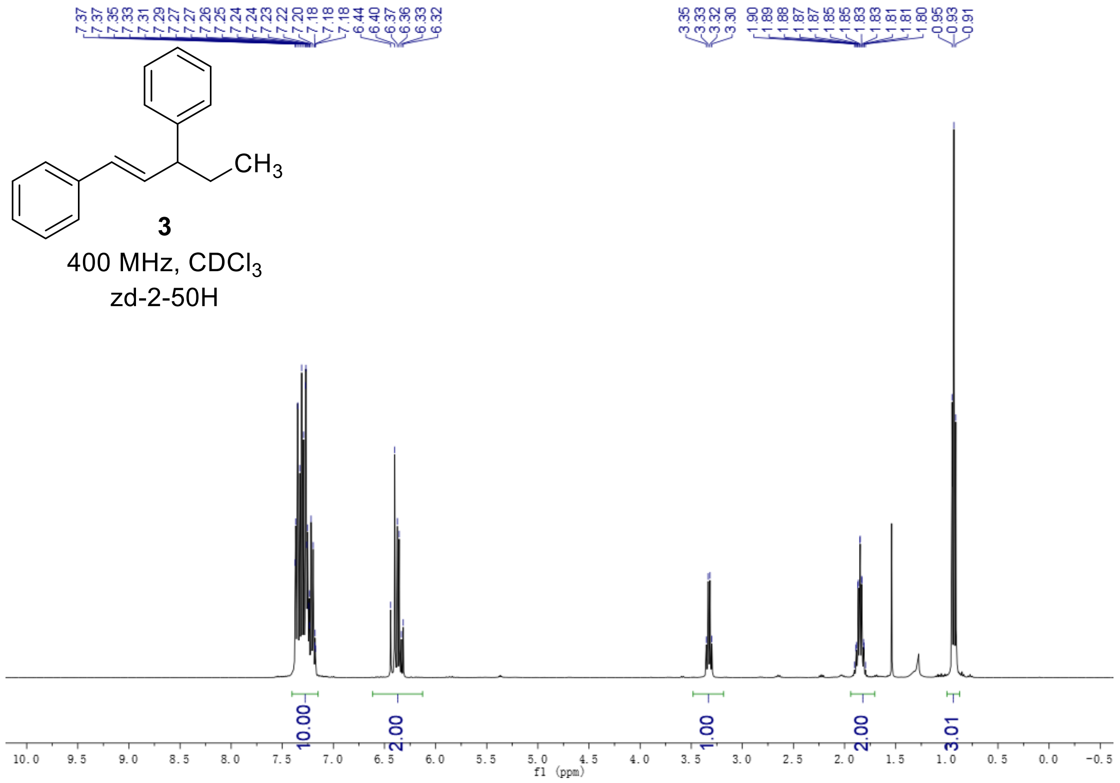

Supplementary Figure 97. ^{1}H NMR (400 MHz, $CDCl_3$, 25 °C) spectra for **3**

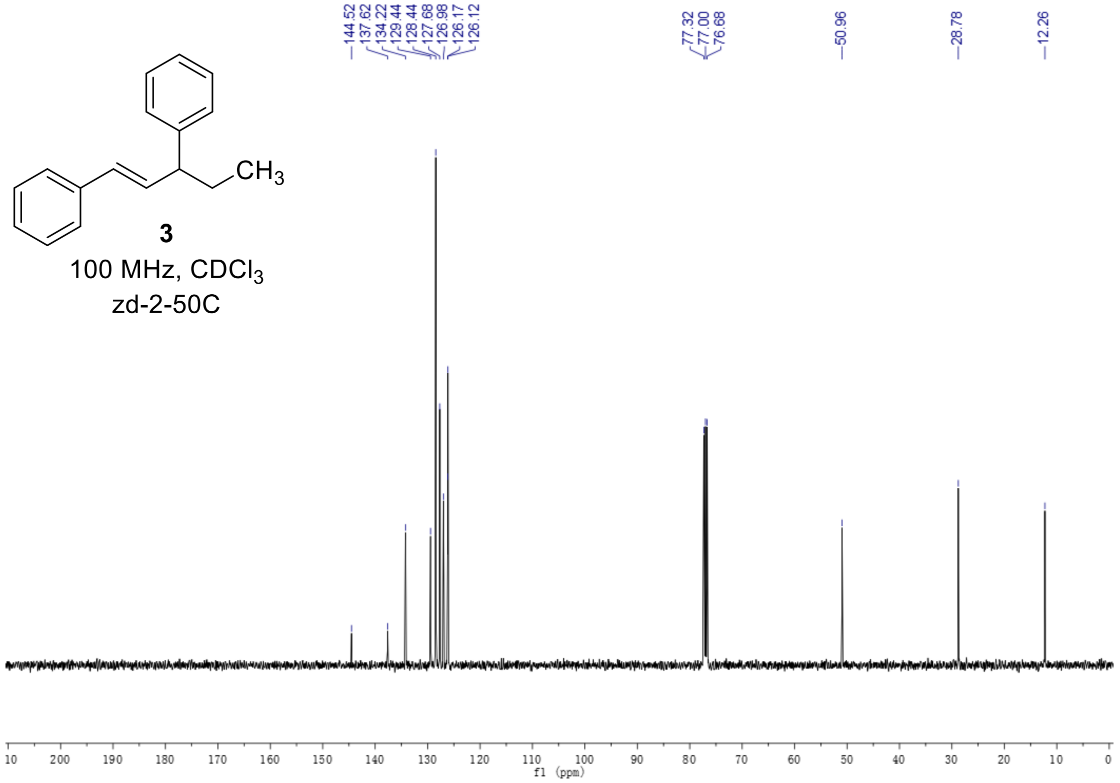

Supplementary Figure 98. ^{13}C NMR (100 MHz, $CDCl_3$, 25 °C) spectra for **3**

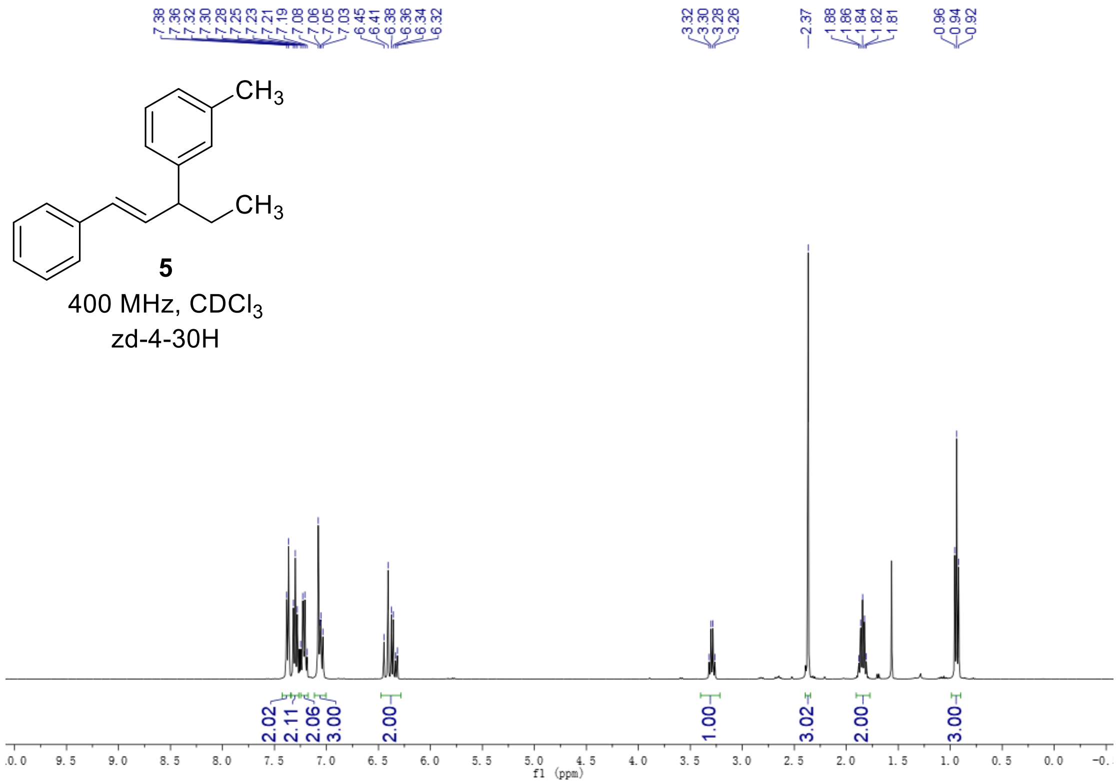

Supplementary Figure 99. ^{1}H NMR (400 MHz, $CDCl_3$, 25 °C) spectra for **5**

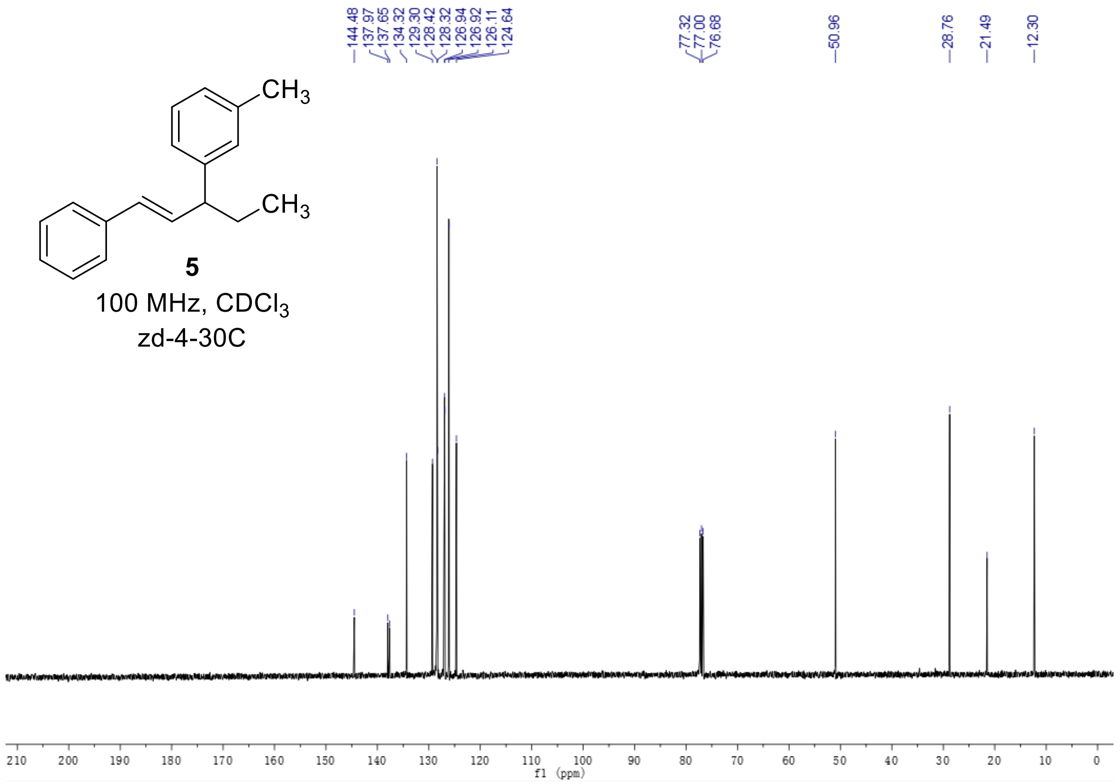

Supplementary Figure 100. ^{13}C NMR (100 MHz, $CDCl_3$, 25 °C) spectra for **5**

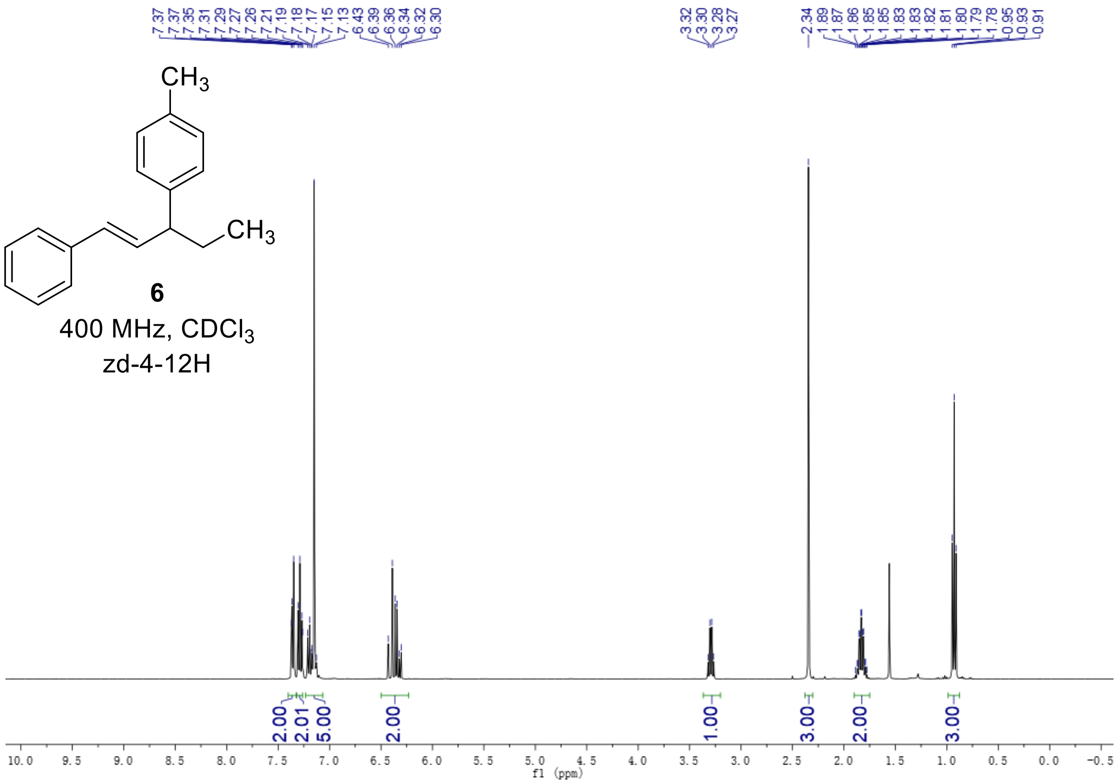

Supplementary Figure 101. ^{1}H NMR (400 MHz, $CDCl_3$, 25 °C) spectra for **6**

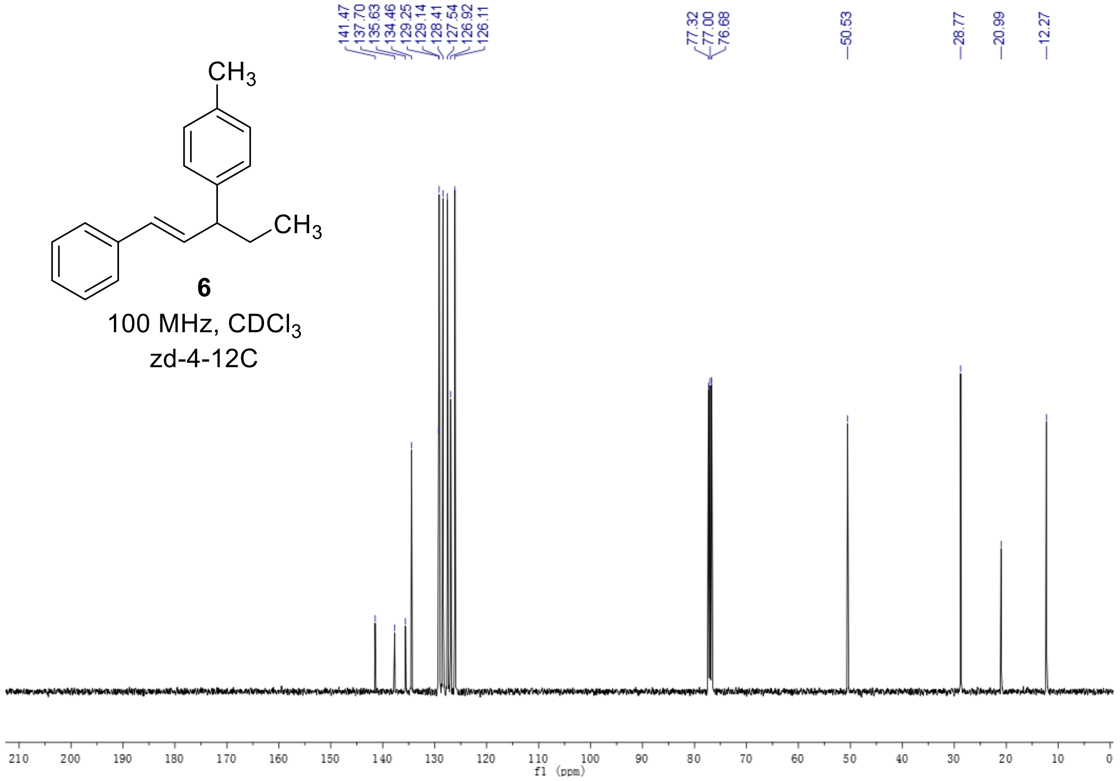

Supplementary Figure 102. ^{13}C NMR (100 MHz, $CDCl_3$, 25 °C) spectra for **6**

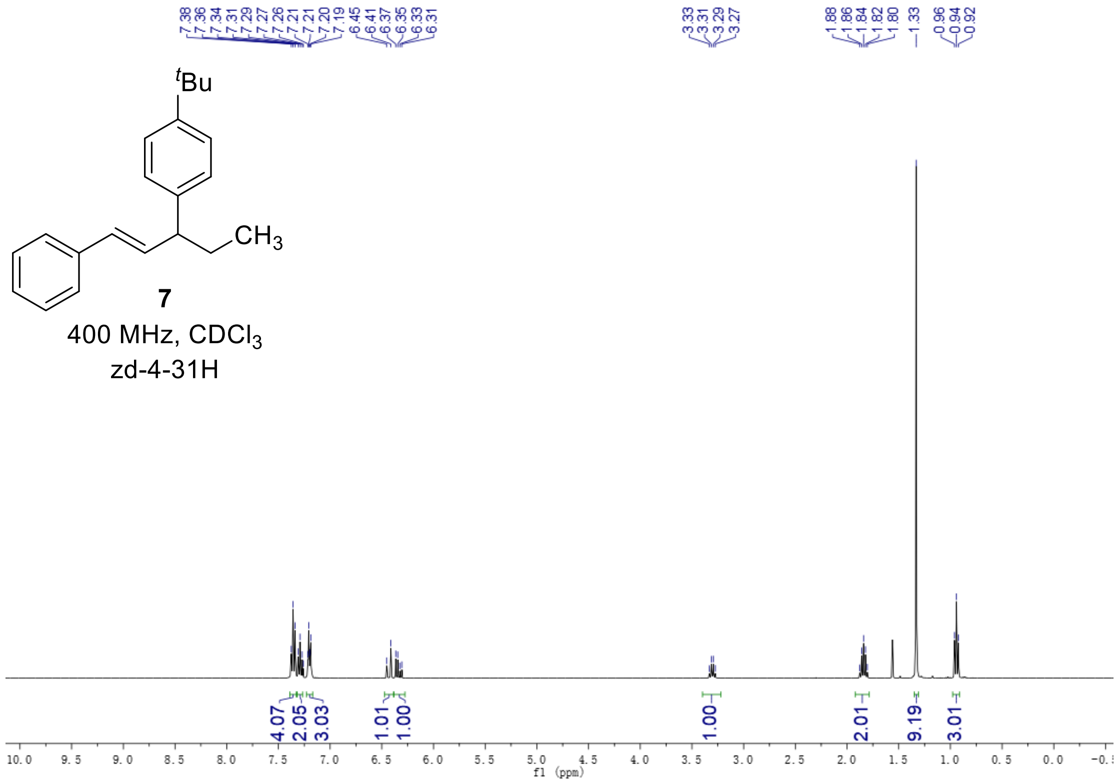

Supplementary Figure 103. ^{1}H NMR (400 MHz, $CDCl_3$, 25 °C) spectra for **7**

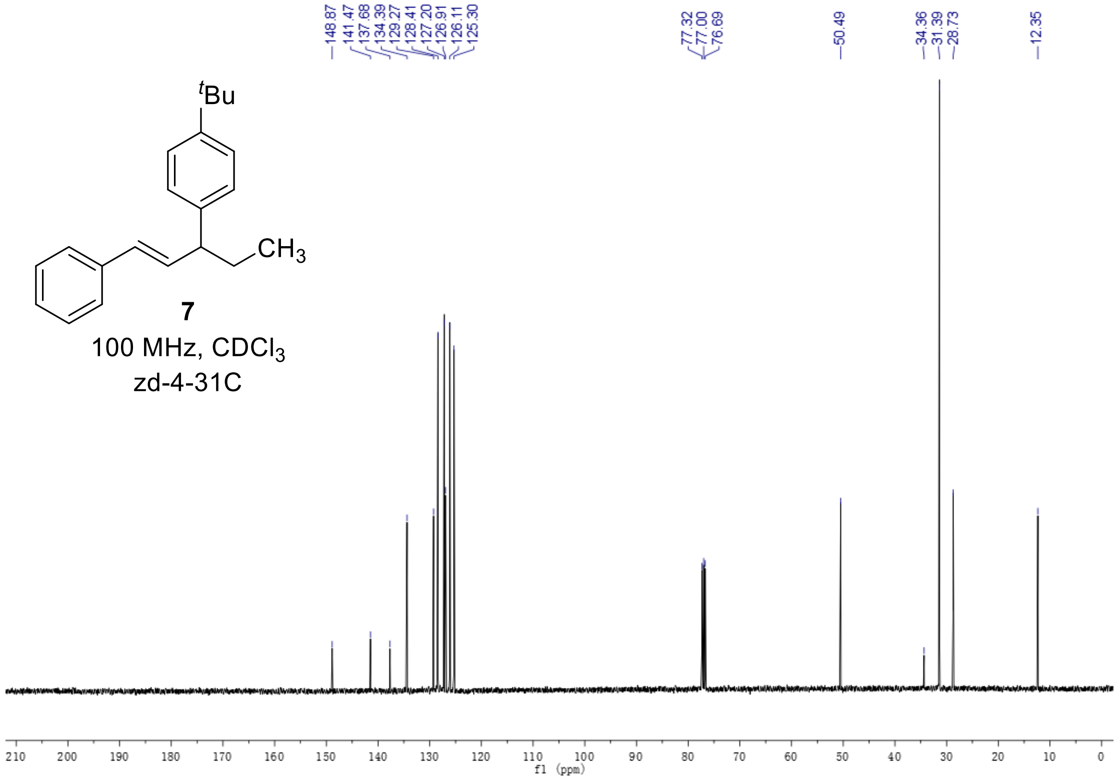

Supplementary Figure 104. ^{13}C NMR (100 MHz, $CDCl_3$, 25 °C) spectra for **7**

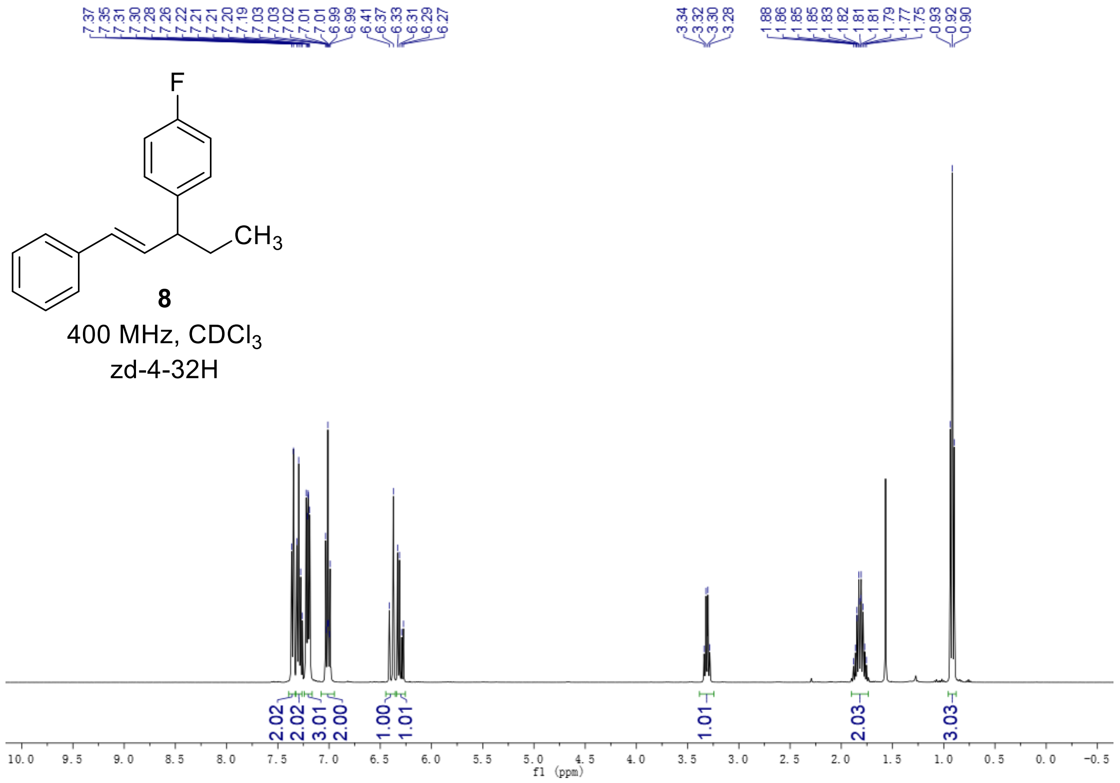

Supplementary Figure 105. ^{1}H NMR (400 MHz, $CDCl_3$, 25 °C) spectra for **8**

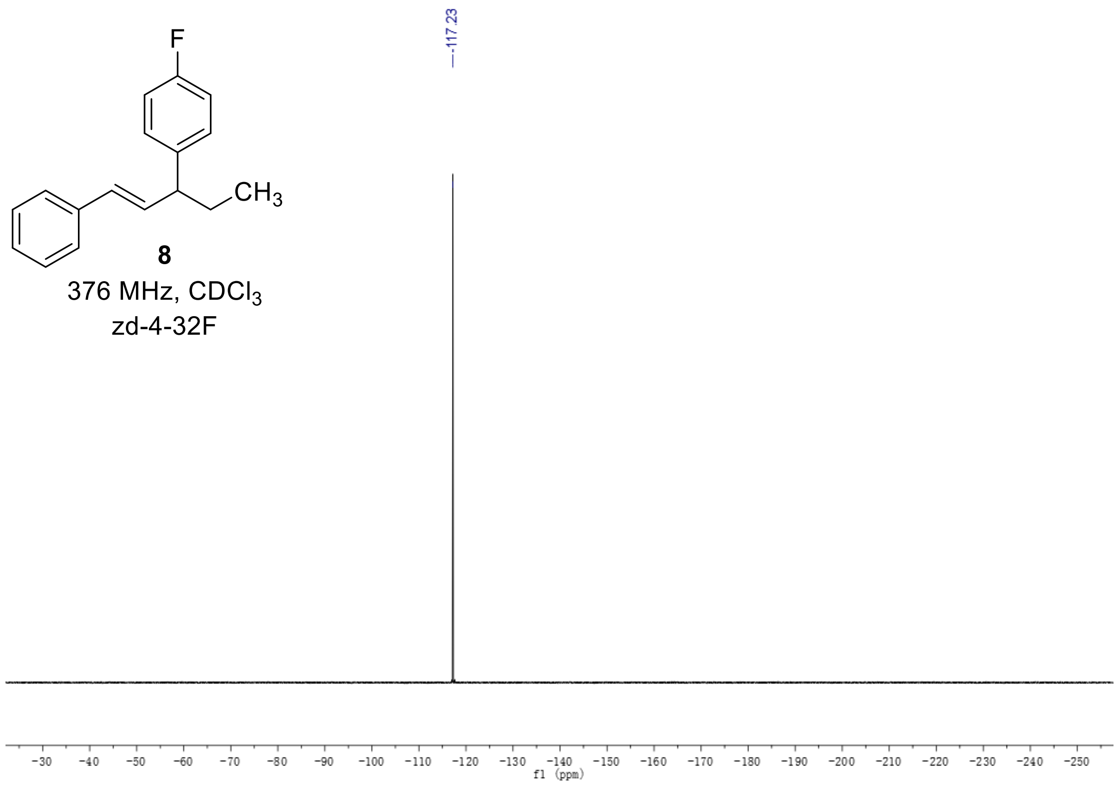

Supplementary Figure 106. ^{19}F NMR (376 MHz, $CDCl_3$, 25 °C) spectra for **8**

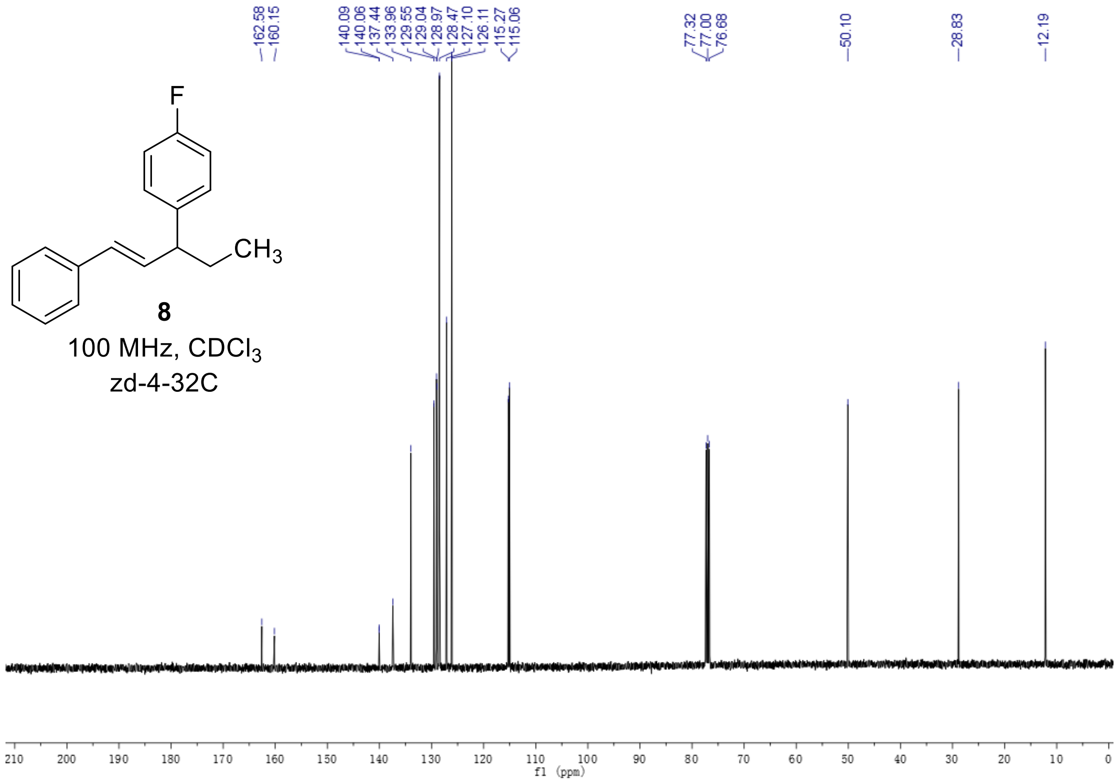

Supplementary Figure 107. ^{13}C NMR (100 MHz, $CDCl_3$, 25 °C) spectra for **8**

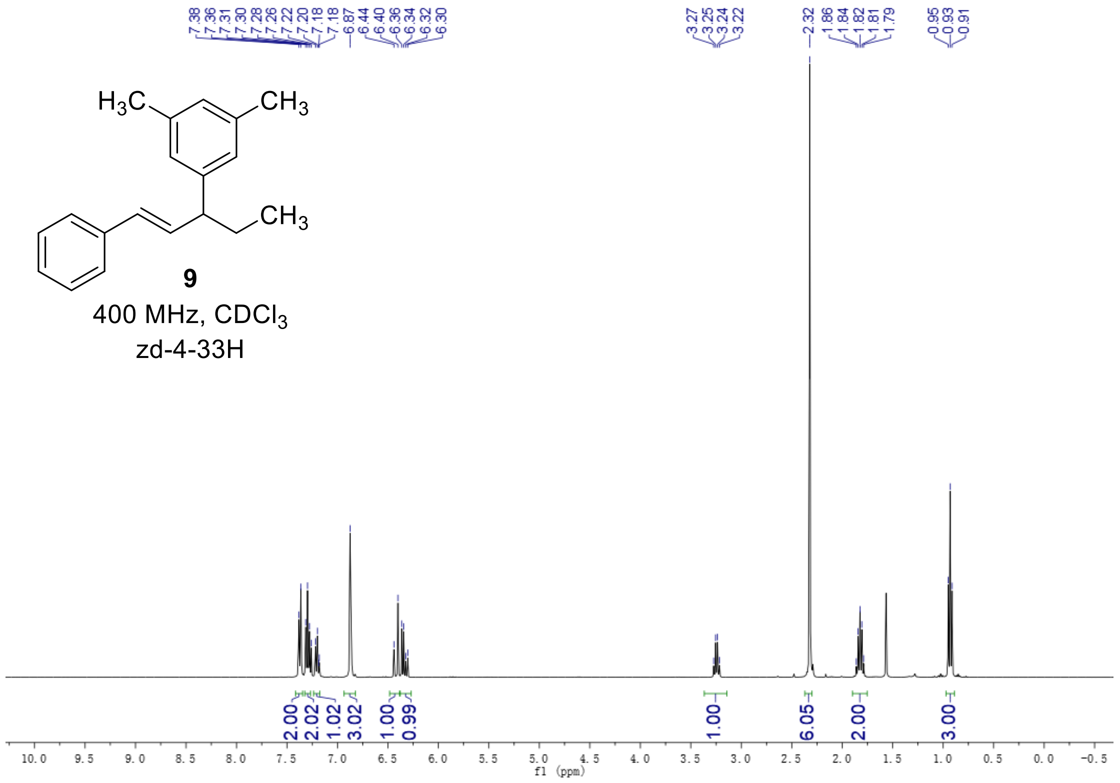

Supplementary Figure 108. ^{1}H NMR (400 MHz, $CDCl_3$, 25 °C) spectra for **9**

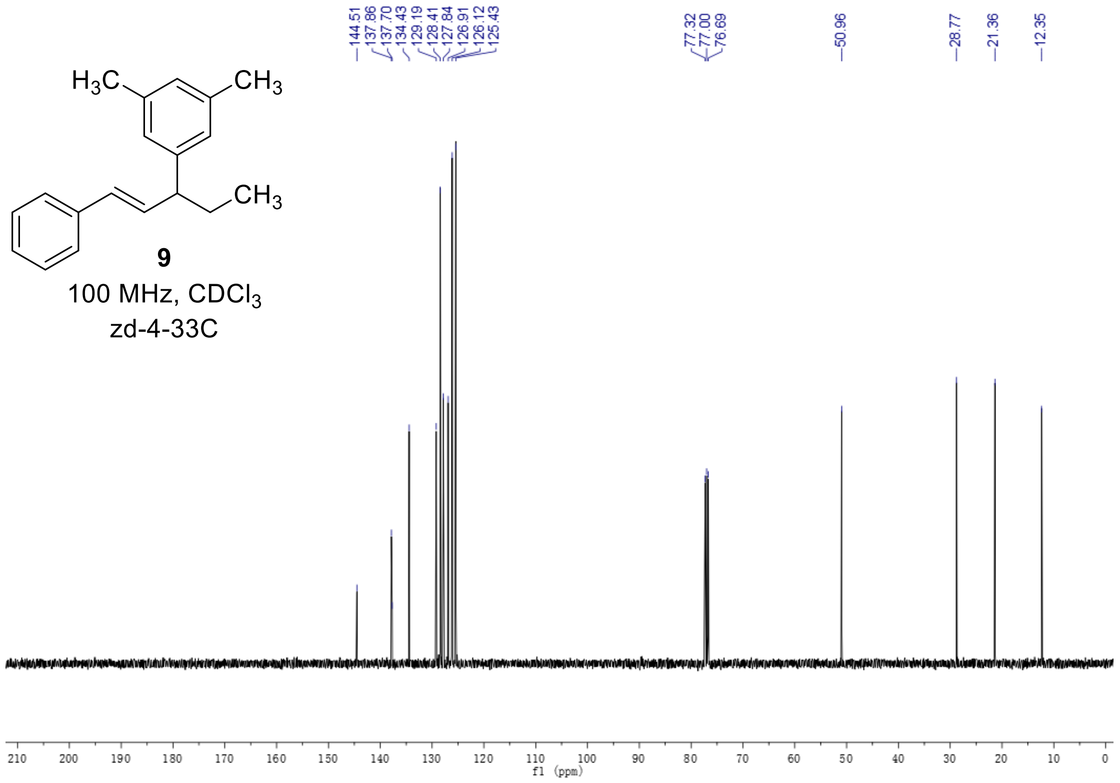

Supplementary Figure 109. ^{13}C NMR (100 MHz, $CDCl_3$, 25 °C) spectra for **9**

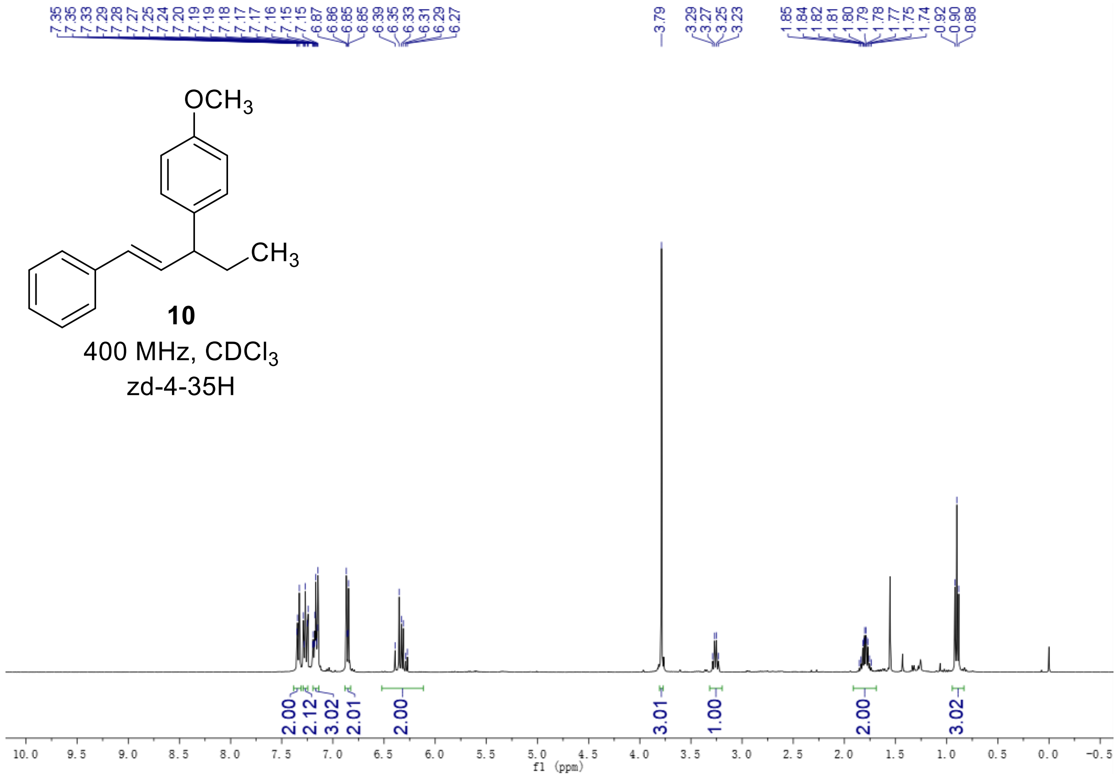

Supplementary Figure 110. ^{1}H NMR (400 MHz, $CDCl_3$, 25 °C) spectra for **10**

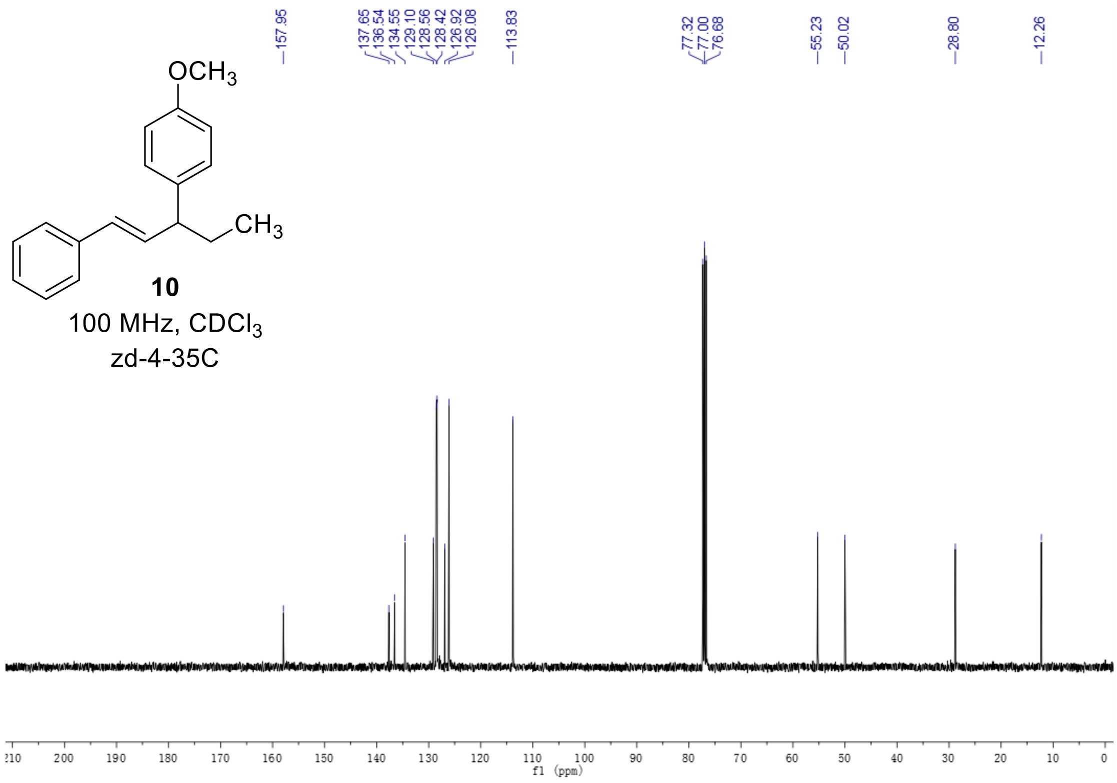

Supplementary Figure 111. ^{13}C NMR (100 MHz, $CDCl_3$, 25 °C) spectra for **10**

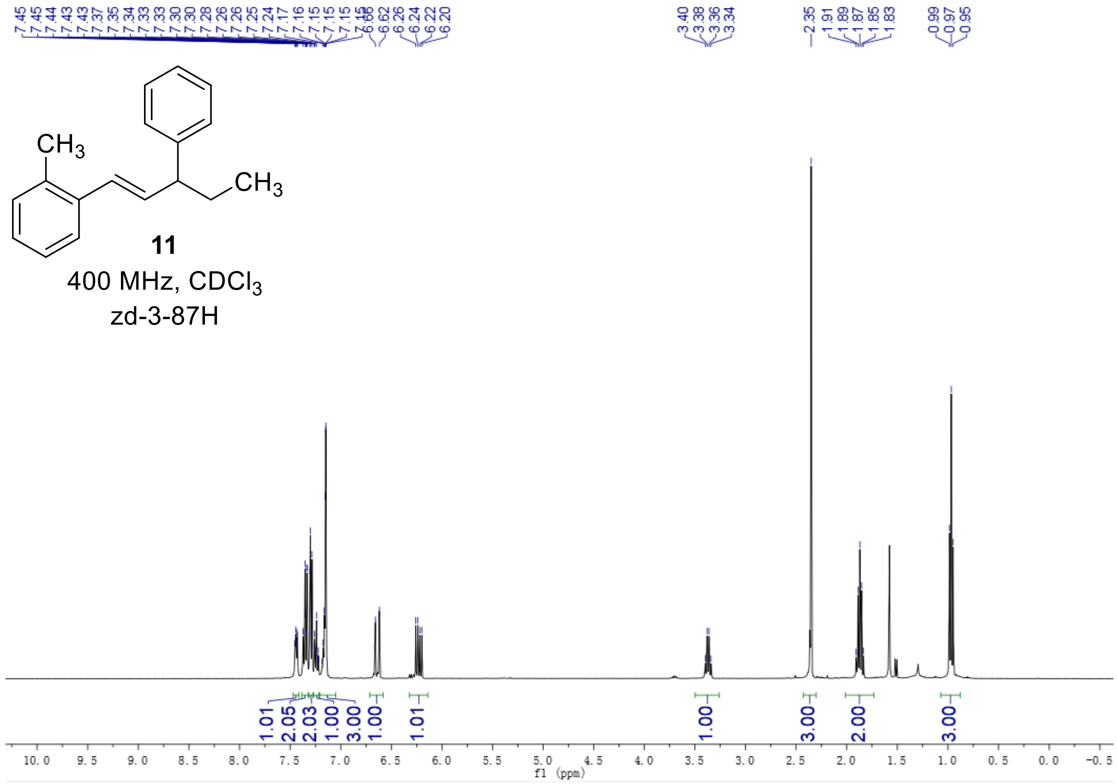

Supplementary Figure 112. ^{1}H NMR (400 MHz, $CDCl_3$, 25 °C) spectra for **11**

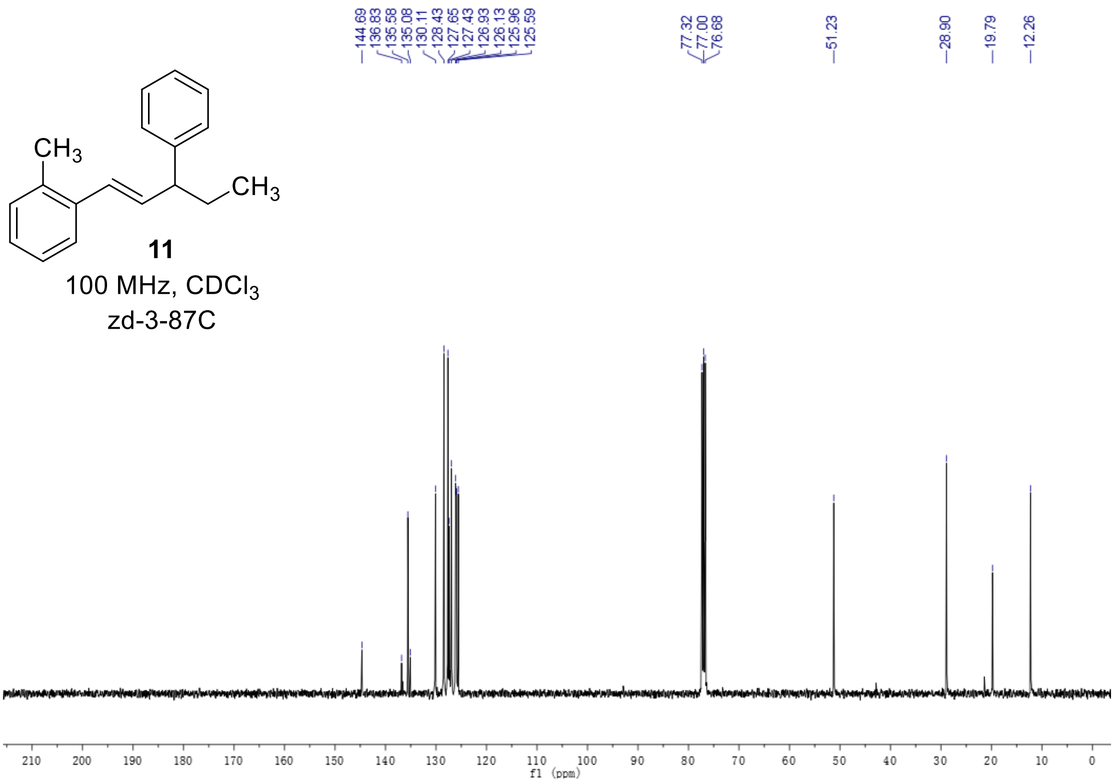

Supplementary Figure 113. ^{13}C NMR (100 MHz, $CDCl_3$, 25 °C) spectra for **11**

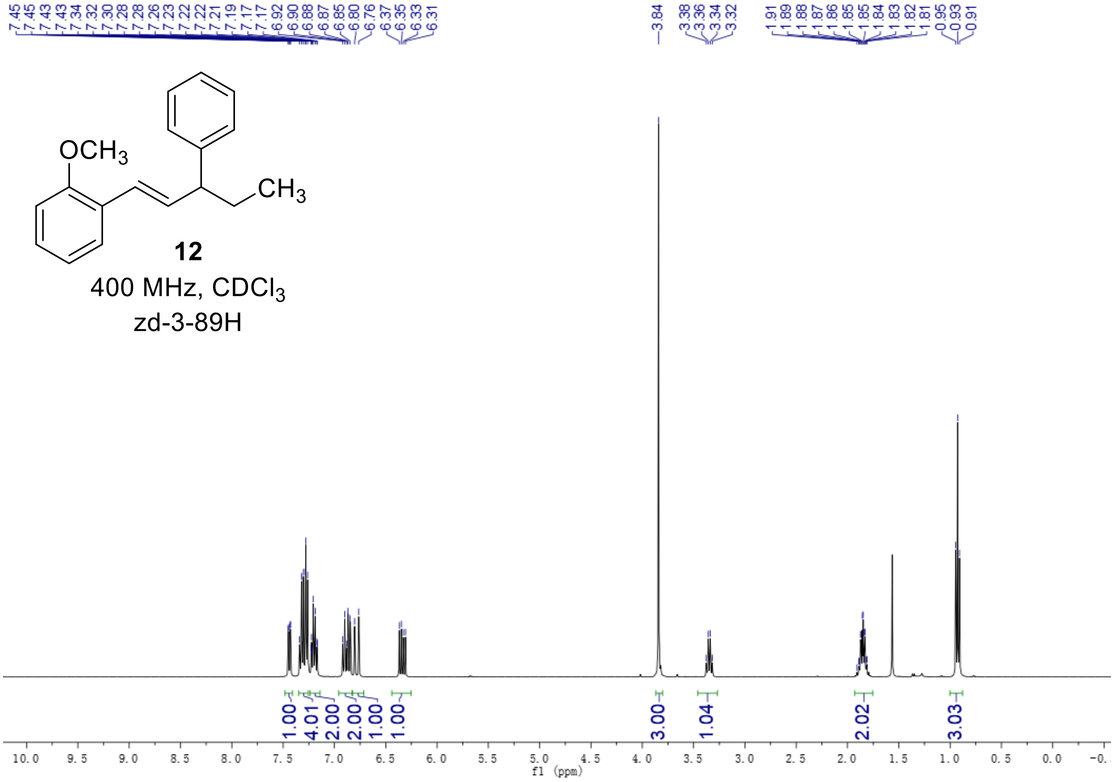

Supplementary Figure 114. ^{1}H NMR (400 MHz, $CDCl_3$, 25 °C) spectra for **12**

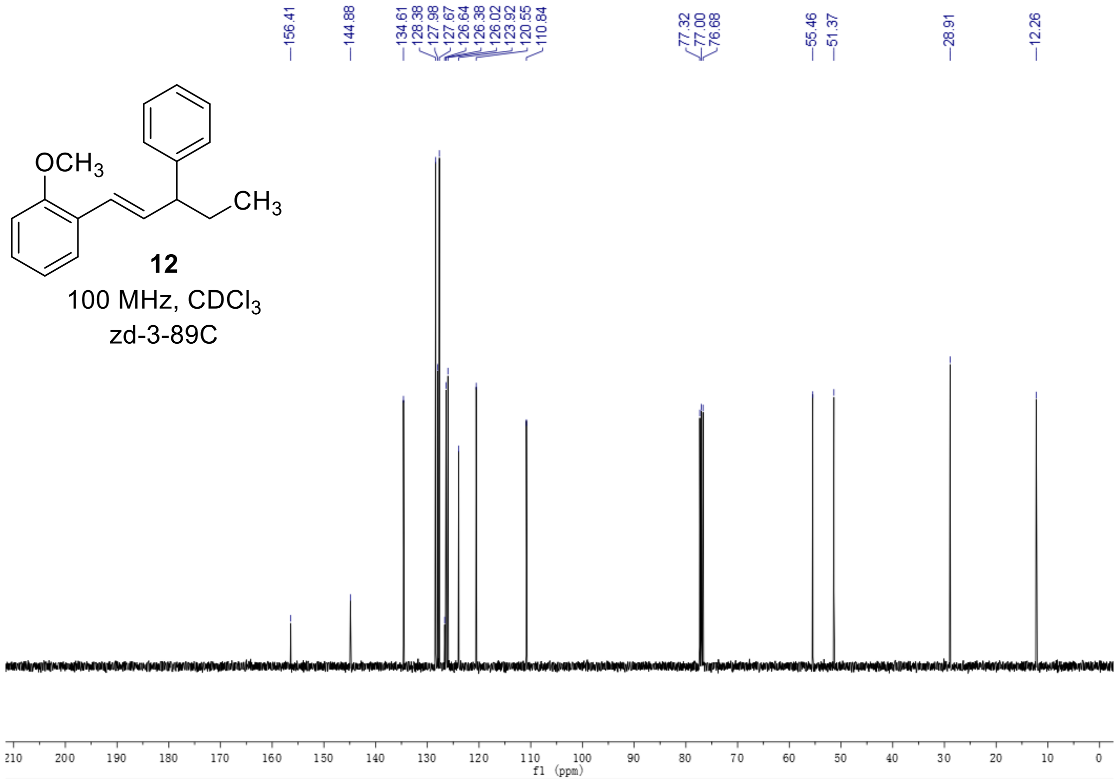

Supplementary Figure 115. ^{13}C NMR (100 MHz, $CDCl_3$, 25 °C) spectra for **12**

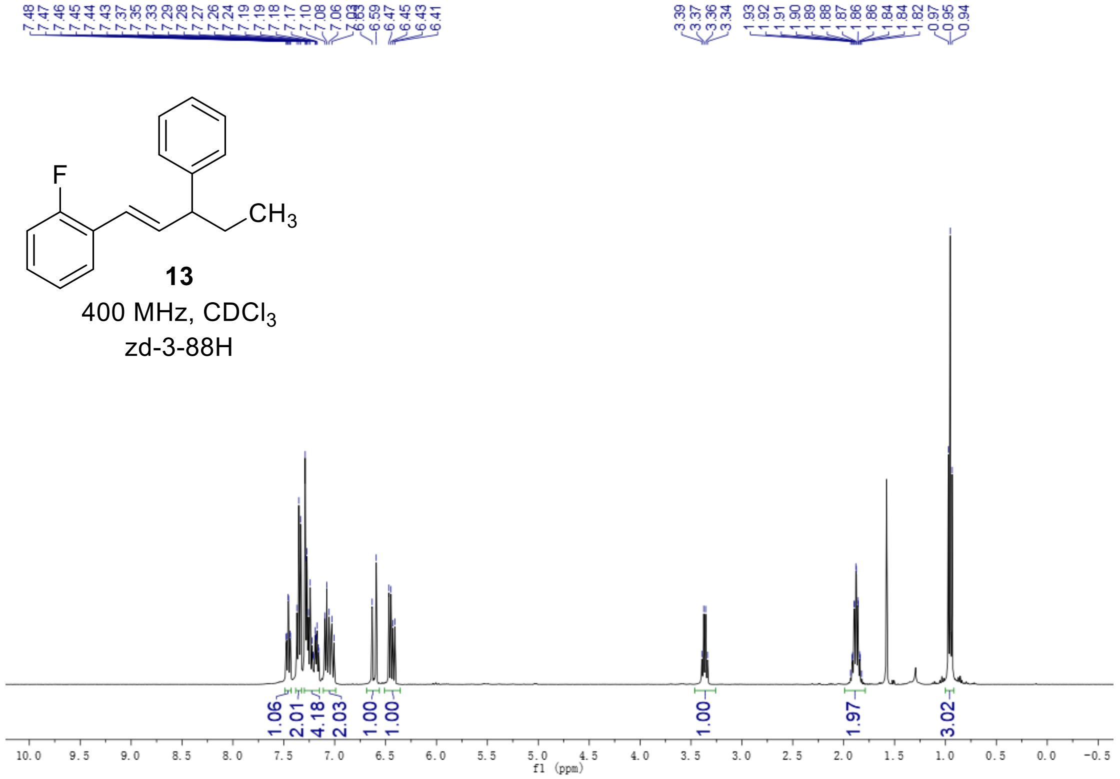

Supplementary Figure 116. ^{1}H NMR (400 MHz, $CDCl_3$, 25 °C) spectra for **13**

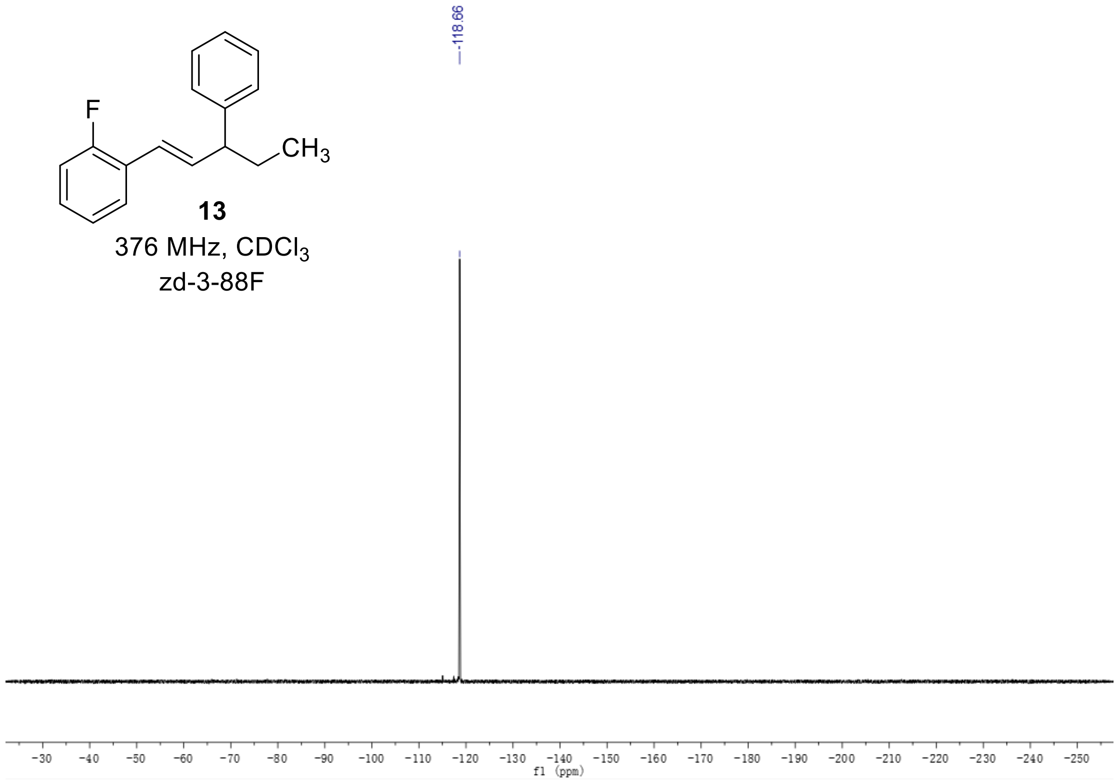

Supplementary Figure 117. ^{19}F NMR (376 MHz, $CDCl_3$, 25 °C) spectra for **13**

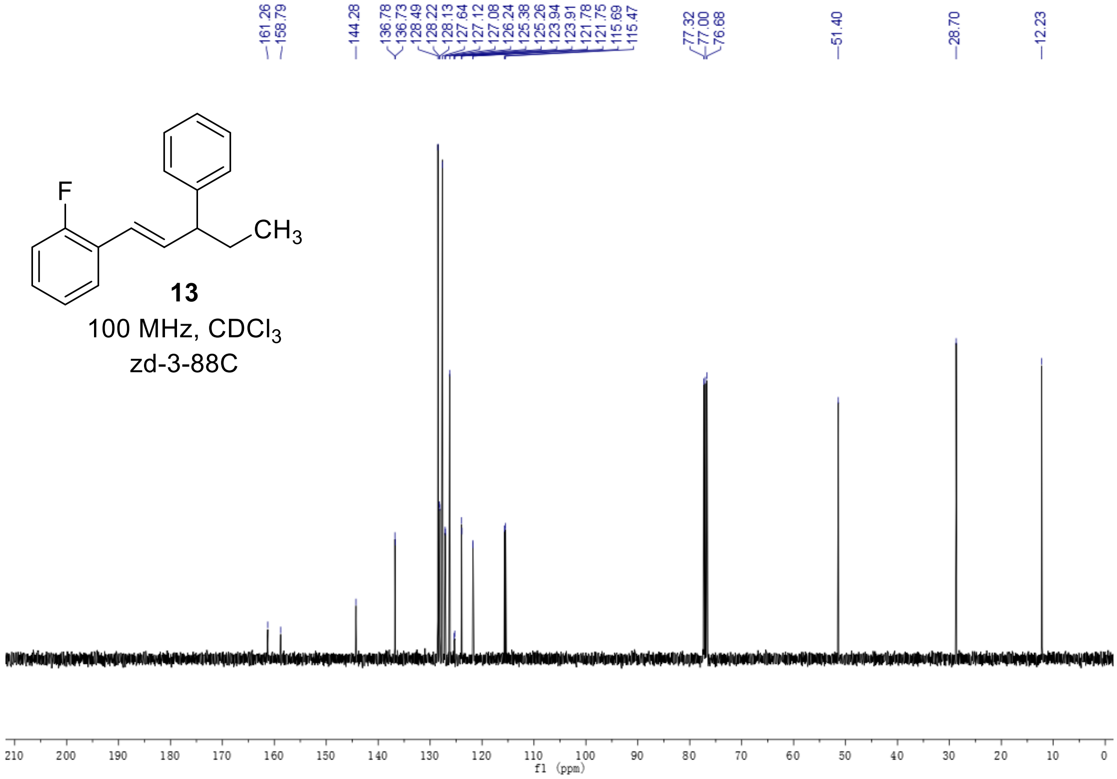

Supplementary Figure 118. ^{13}C NMR (100 MHz, $CDCl_3$, 25 °C) spectra for **13**

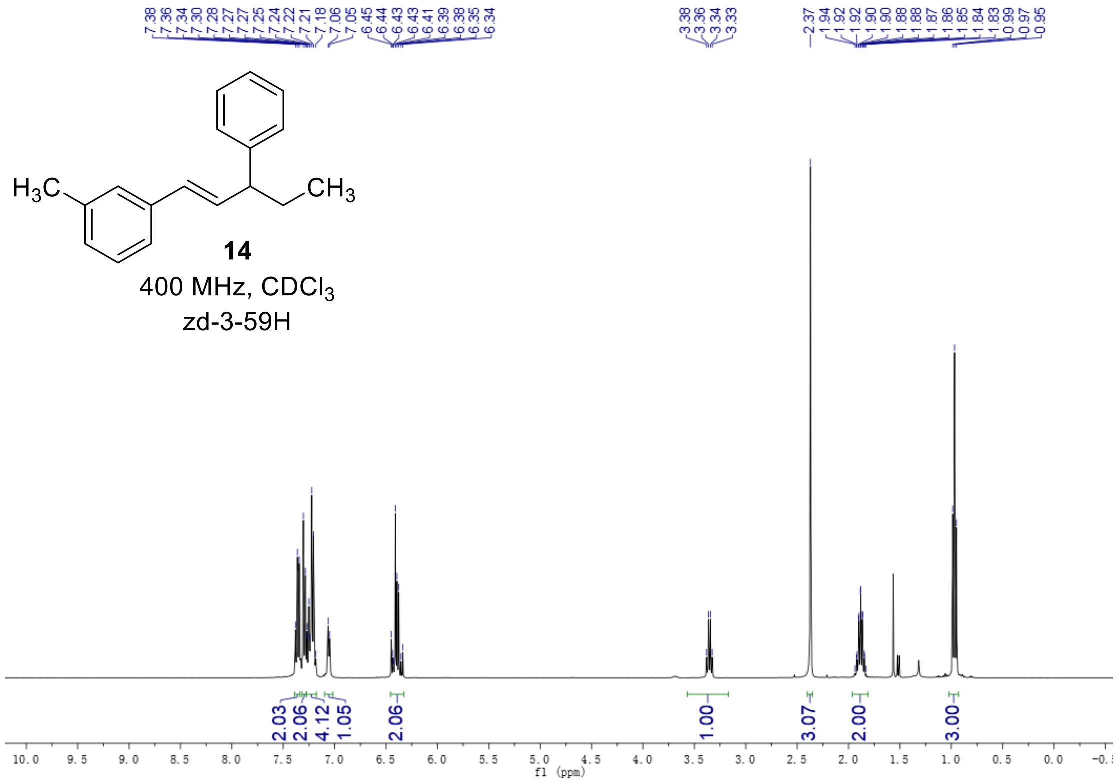

Supplementary Figure 119. ^{1}H NMR (400 MHz, $CDCl_3$, 25 °C) spectra for **14**

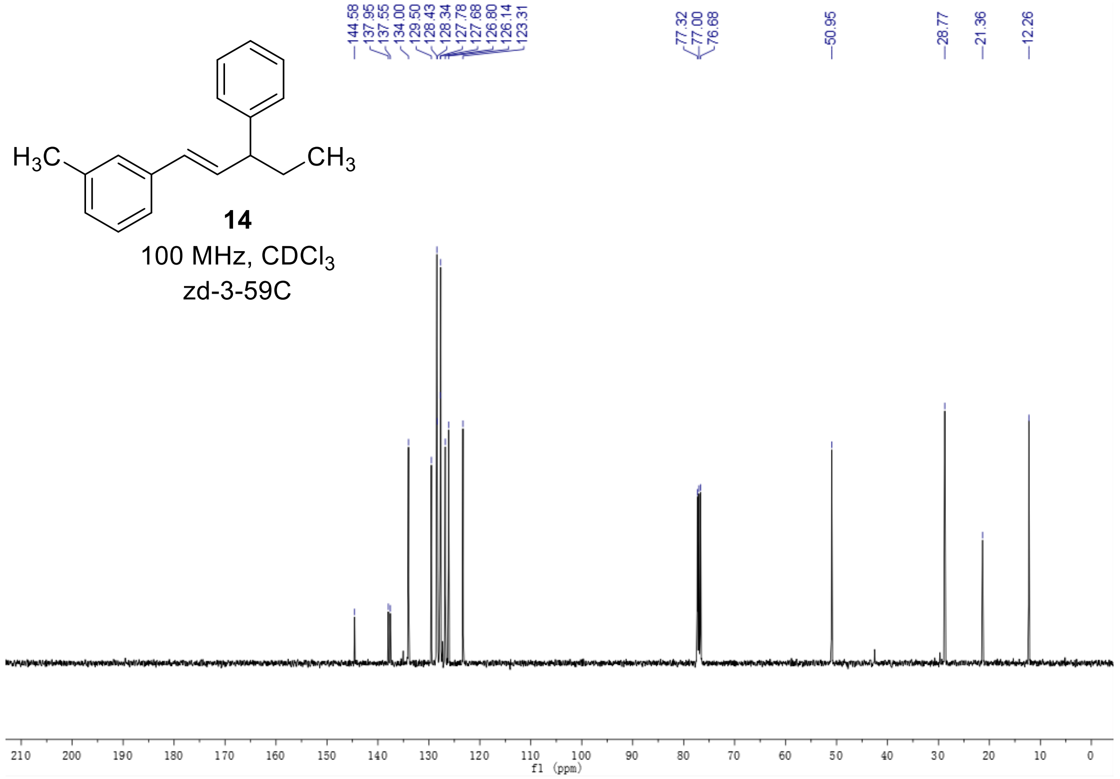

Supplementary Figure 120. ^{13}C NMR (100 MHz, $CDCl_3$, 25 °C) spectra for **14**

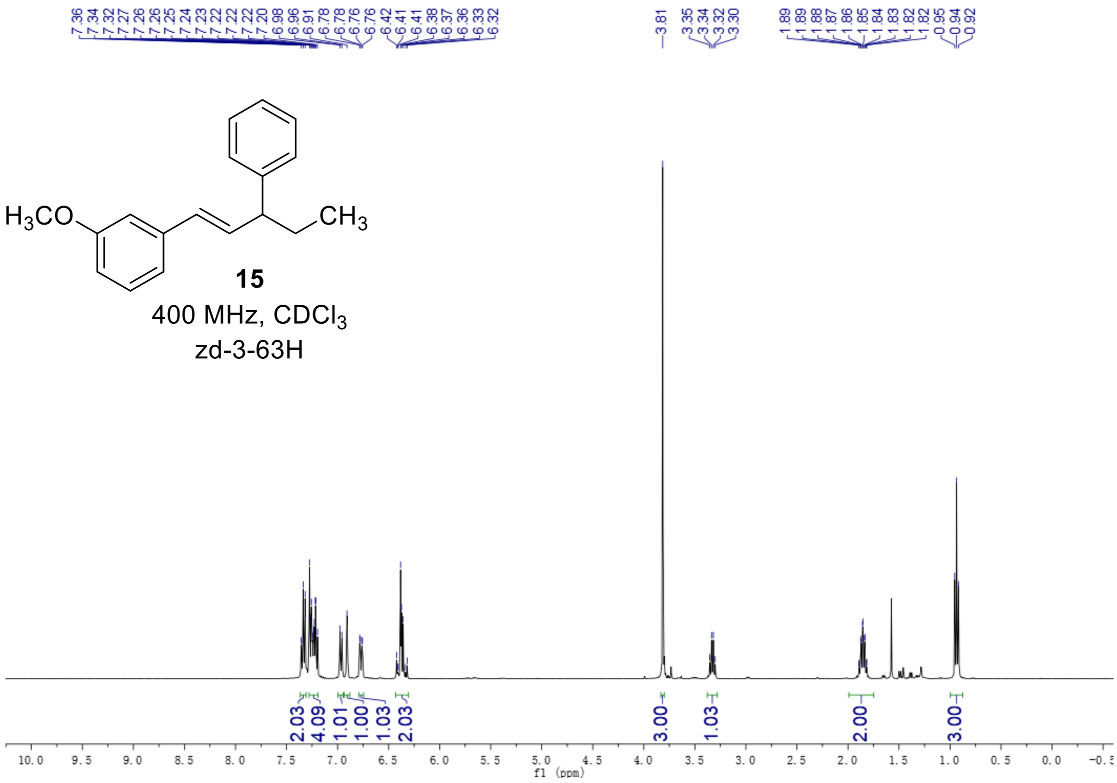

Supplementary Figure 121. ^{1}H NMR (400 MHz, $CDCl_3$, 25 °C) spectra for **15**

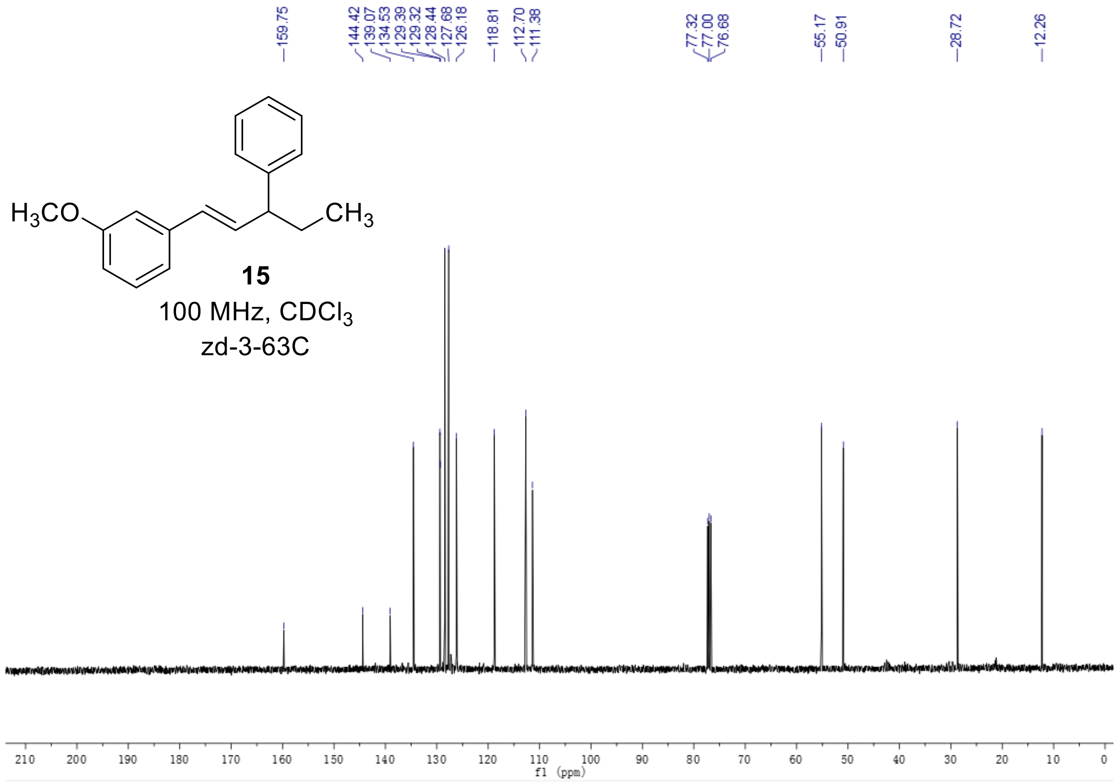

Supplementary Figure 122. ^{13}C NMR (100 MHz, $CDCl_3$, 25 °C) spectra for **15**

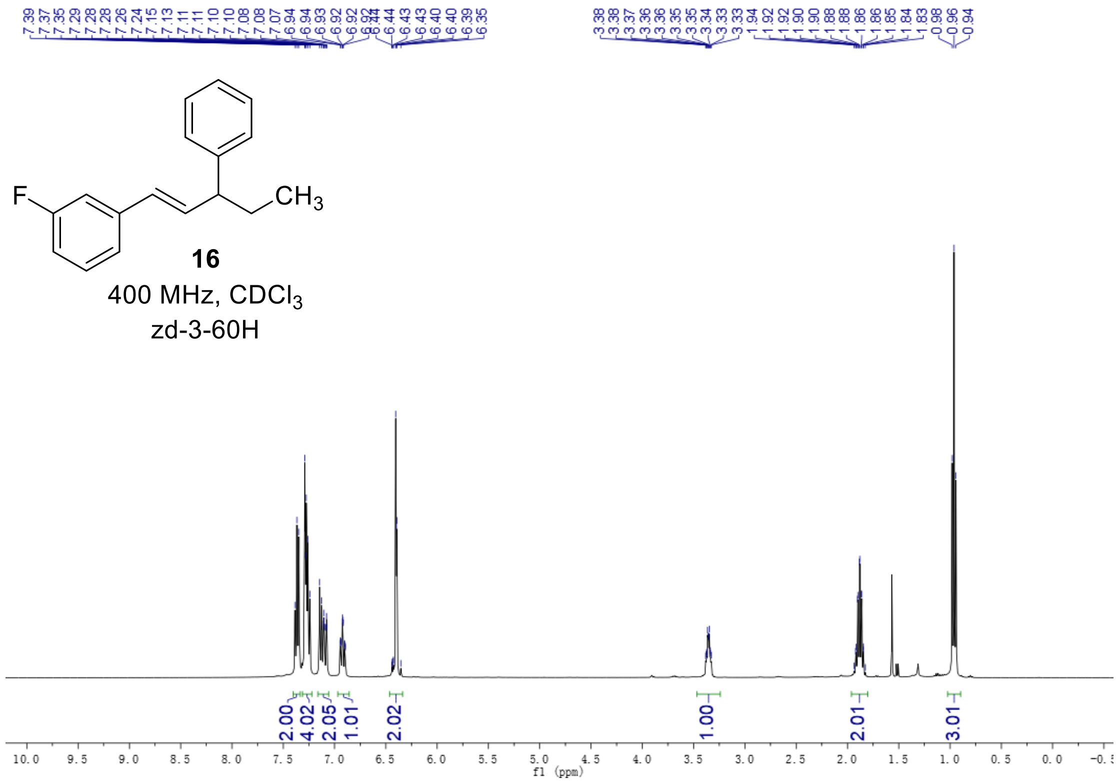

Supplementary Figure 123. ^{1}H NMR (400 MHz, $CDCl_3$, 25 °C) spectra for **16**

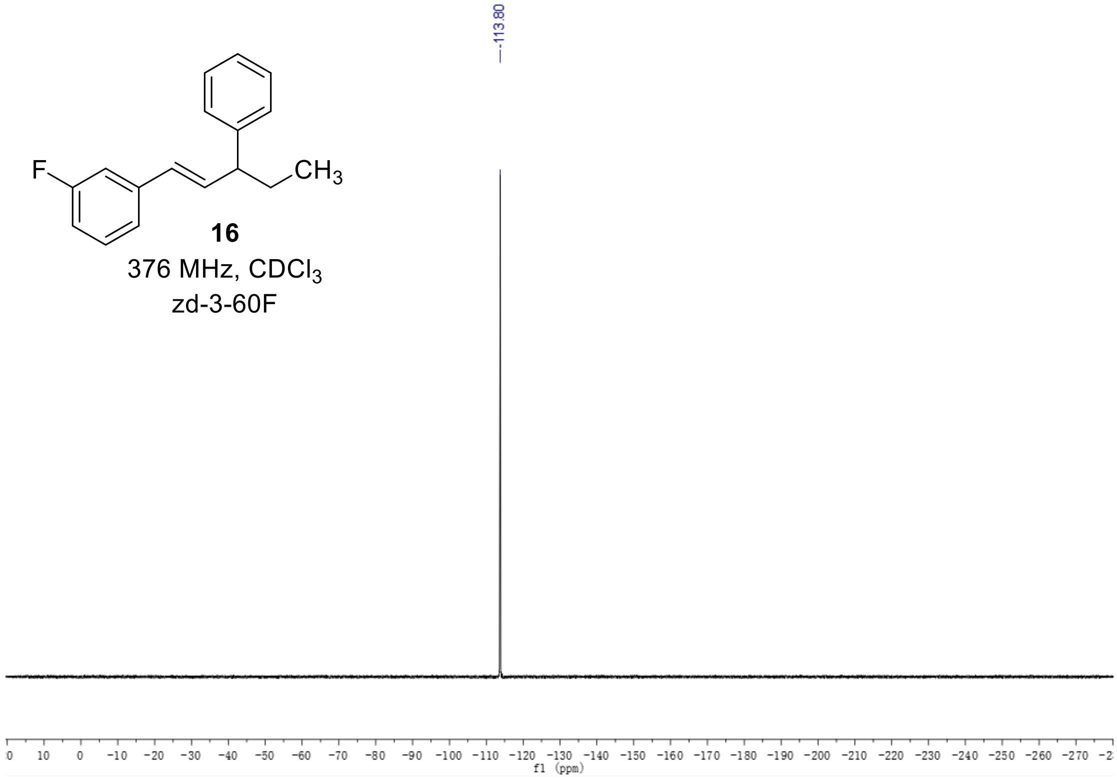

Supplementary Figure 124. ^{19}F NMR (376 MHz, $CDCl_3$, 25 °C) spectra for **16**

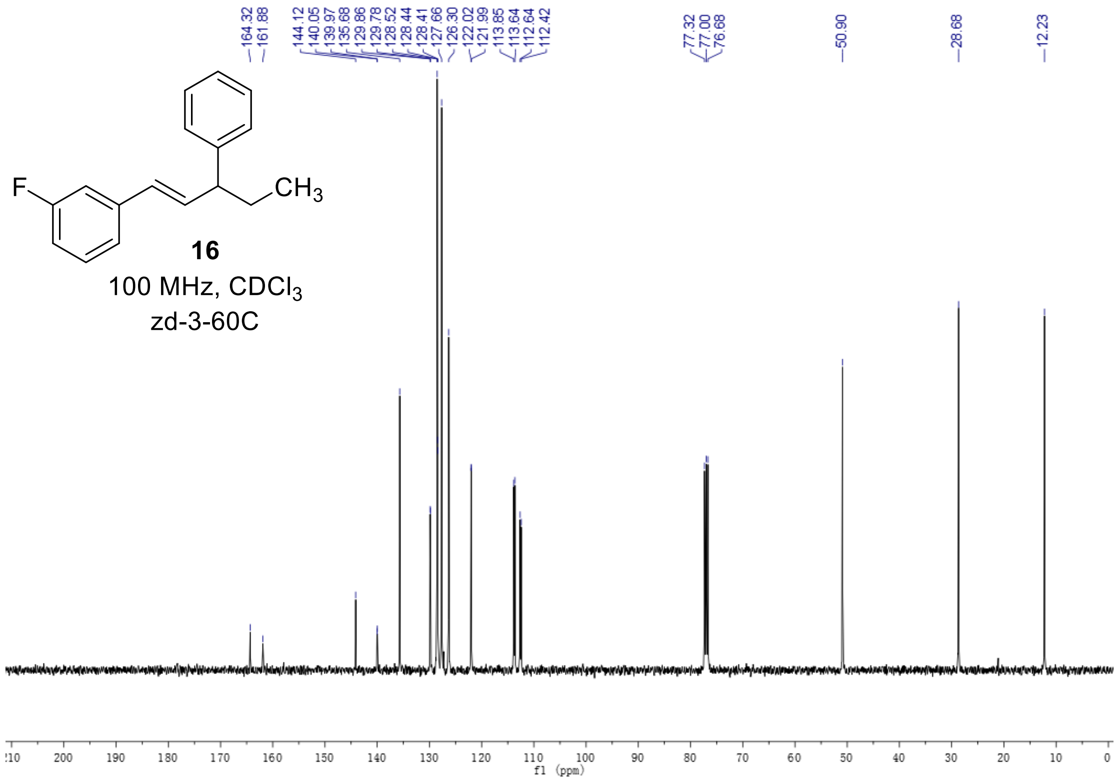

Supplementary Figure 125. ^{13}C NMR (100 MHz, $CDCl_3$, 25 °C) spectra for **16**

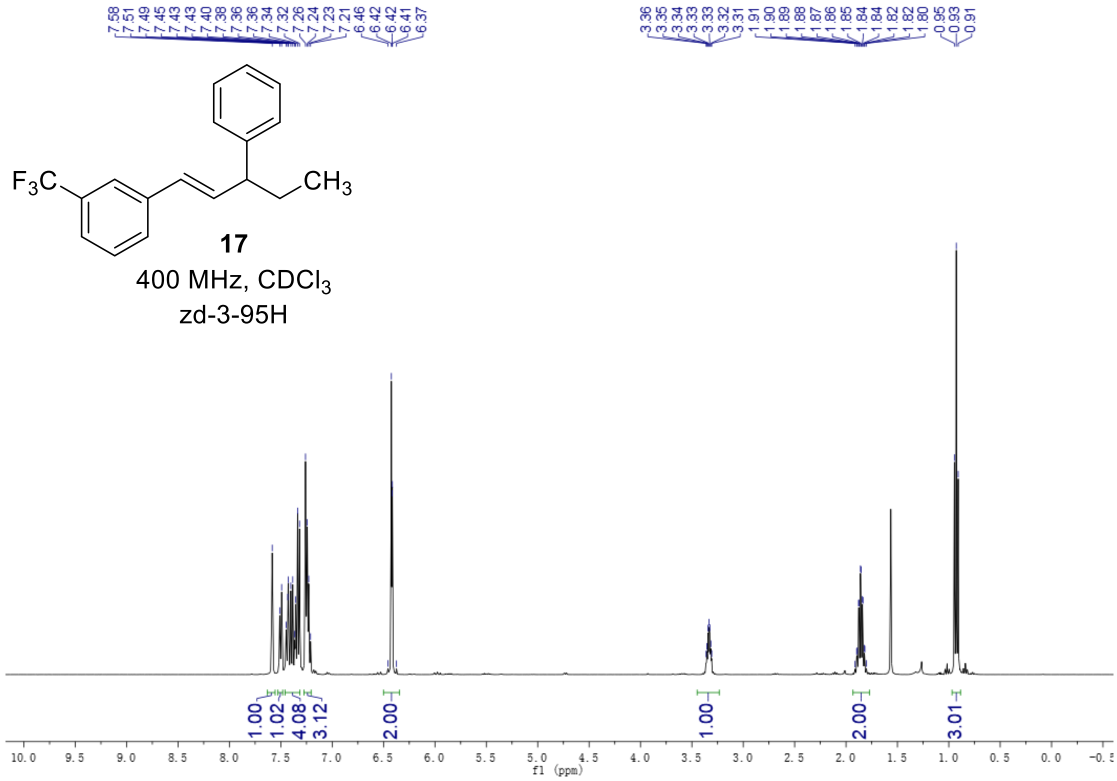

Supplementary Figure 126. ^{1}H NMR (400 MHz, $CDCl_3$, 25 °C) spectra for **17**

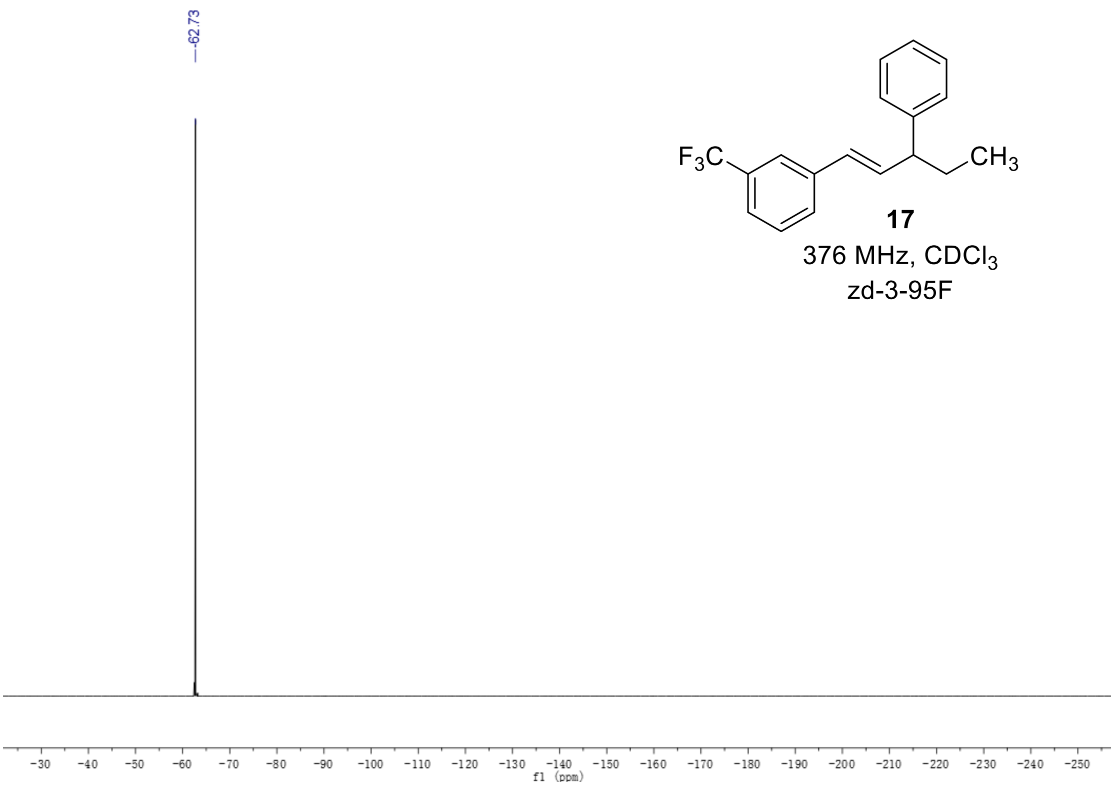

Supplementary Figure 127. ^{19}F NMR (376 MHz, $CDCl_3$, 25 °C) spectra for **17**

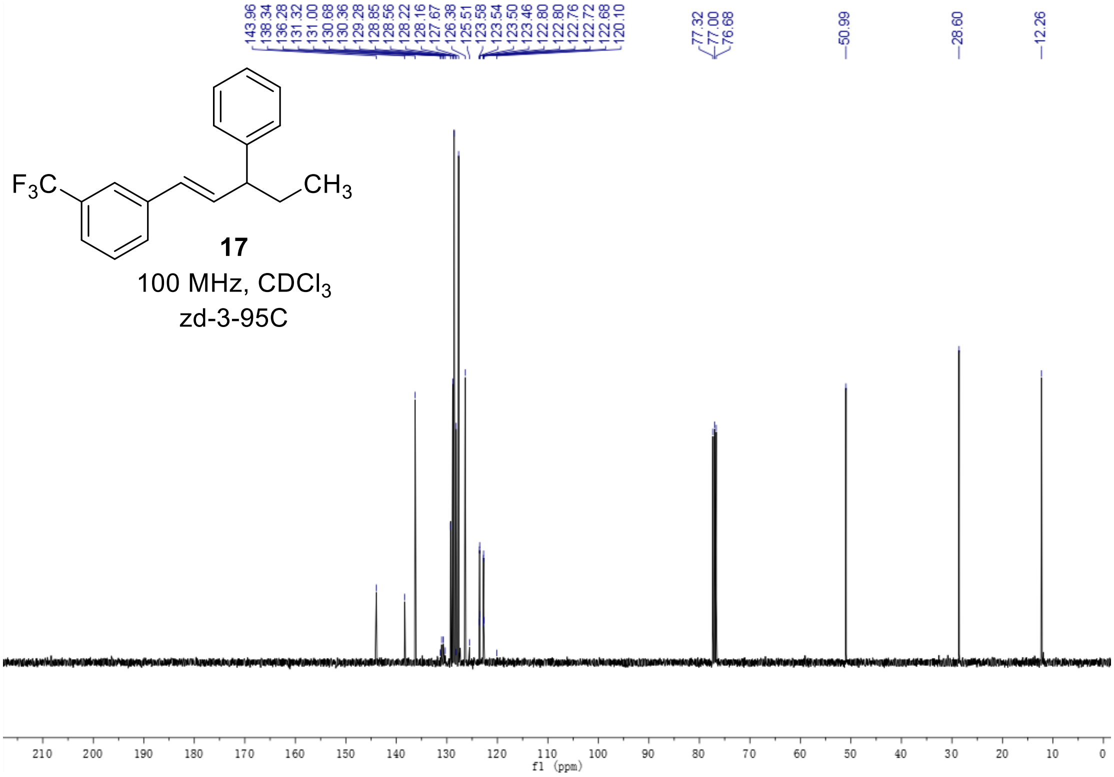

Supplementary Figure 128. ^{13}C NMR (100 MHz, $CDCl_3$, 25 °C) spectra for **17**

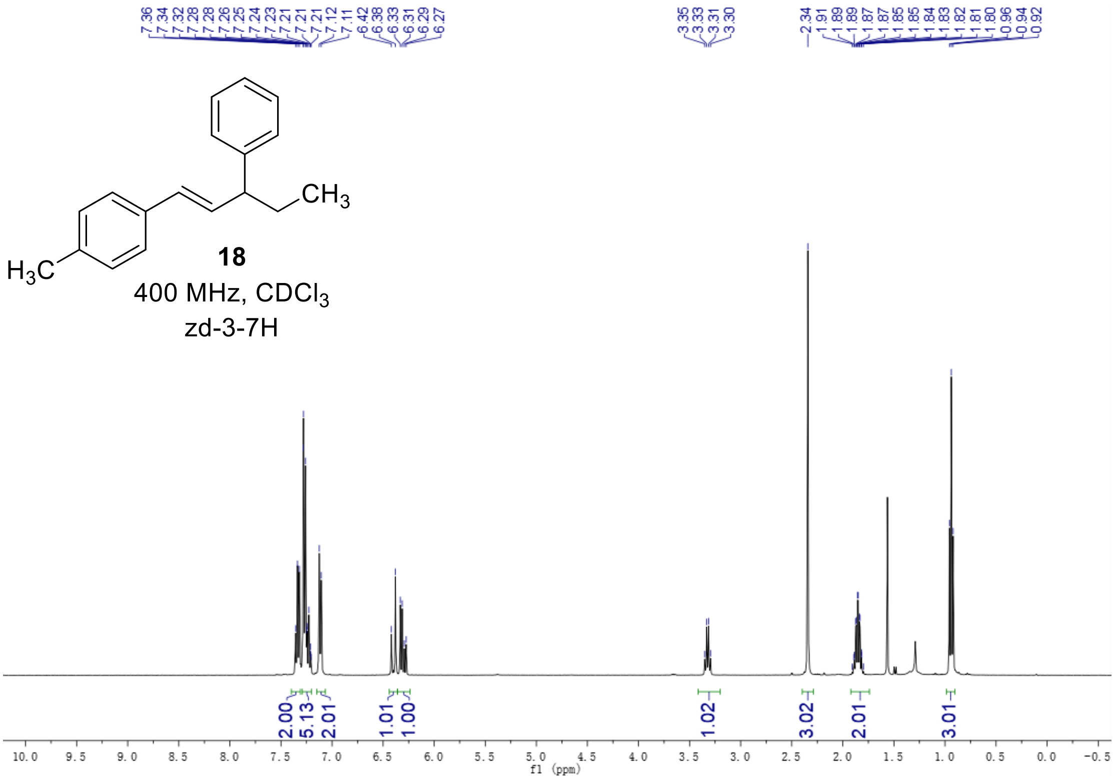

Supplementary Figure 129. ^{1}H NMR (400 MHz, $CDCl_3$, 25 °C) spectra for **18**

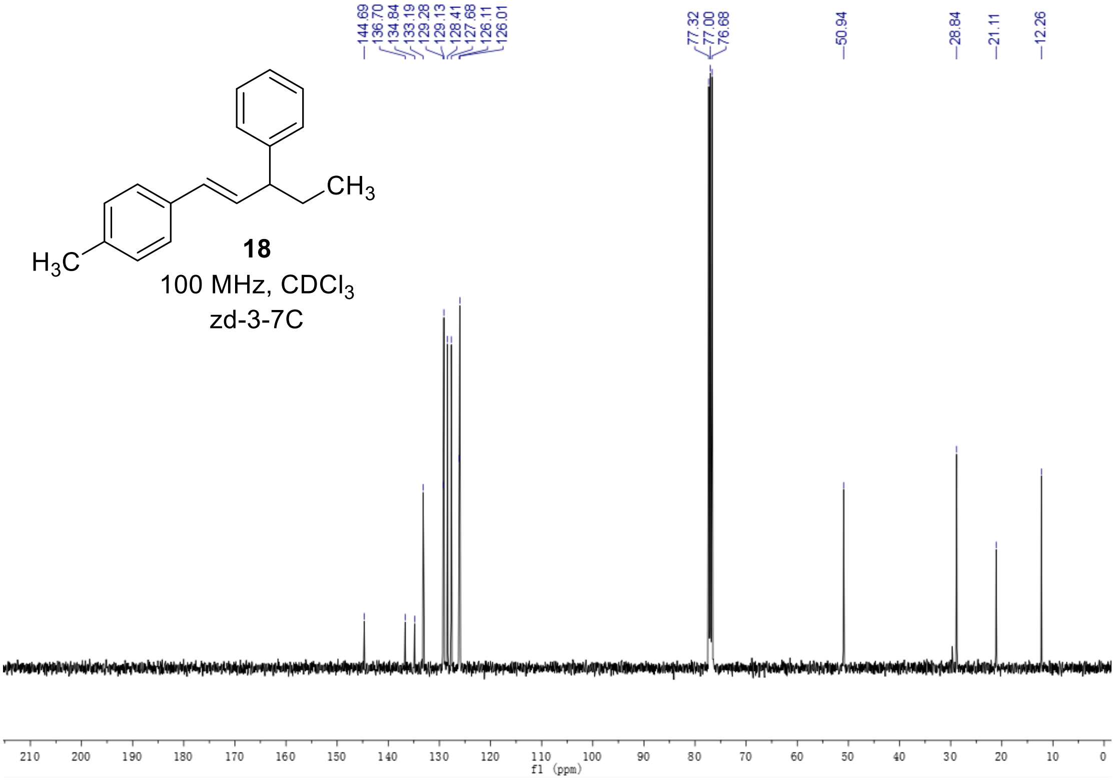

Supplementary Figure 130. ^{13}C NMR (100 MHz, $CDCl_3$, 25 °C) spectra for **18**

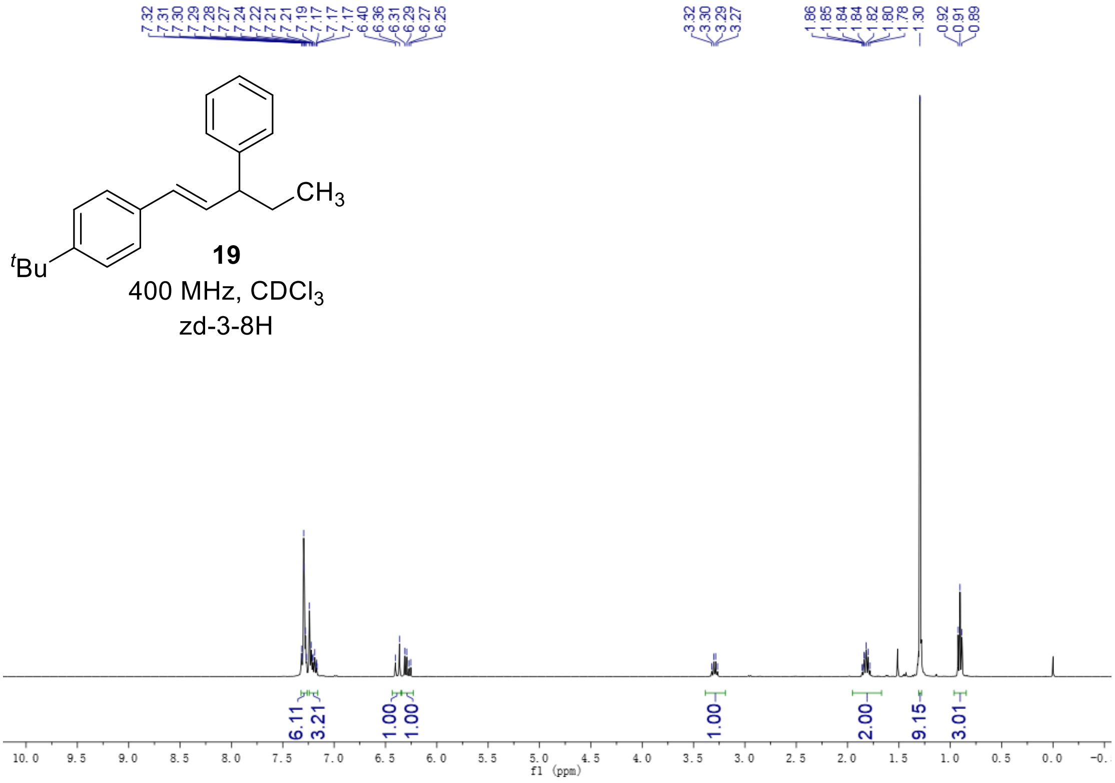

Supplementary Figure 131. ^{1}H NMR (400 MHz, $CDCl_3$, 25 °C) spectra for **19**

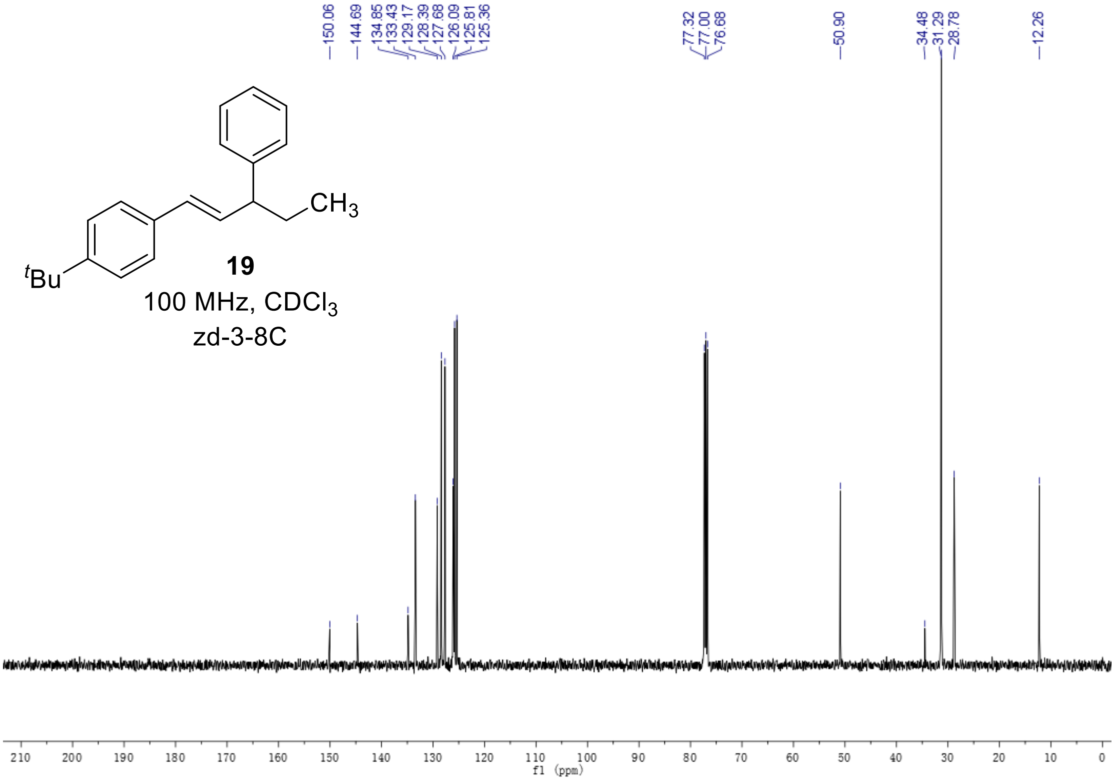

Supplementary Figure 132. ^{13}C NMR (100 MHz, $CDCl_3$, 25 °C) spectra for **19**

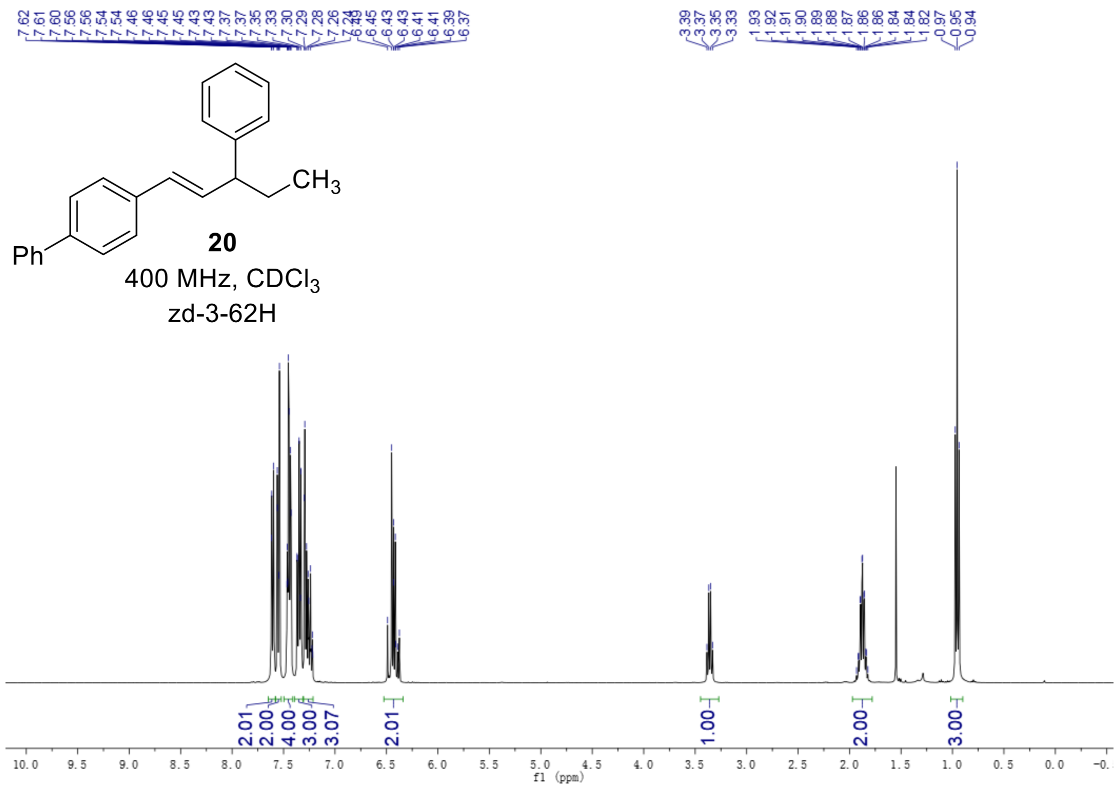

Supplementary Figure 133. ^{1}H NMR (400 MHz, $CDCl_3$, 25 °C) spectra for **20**

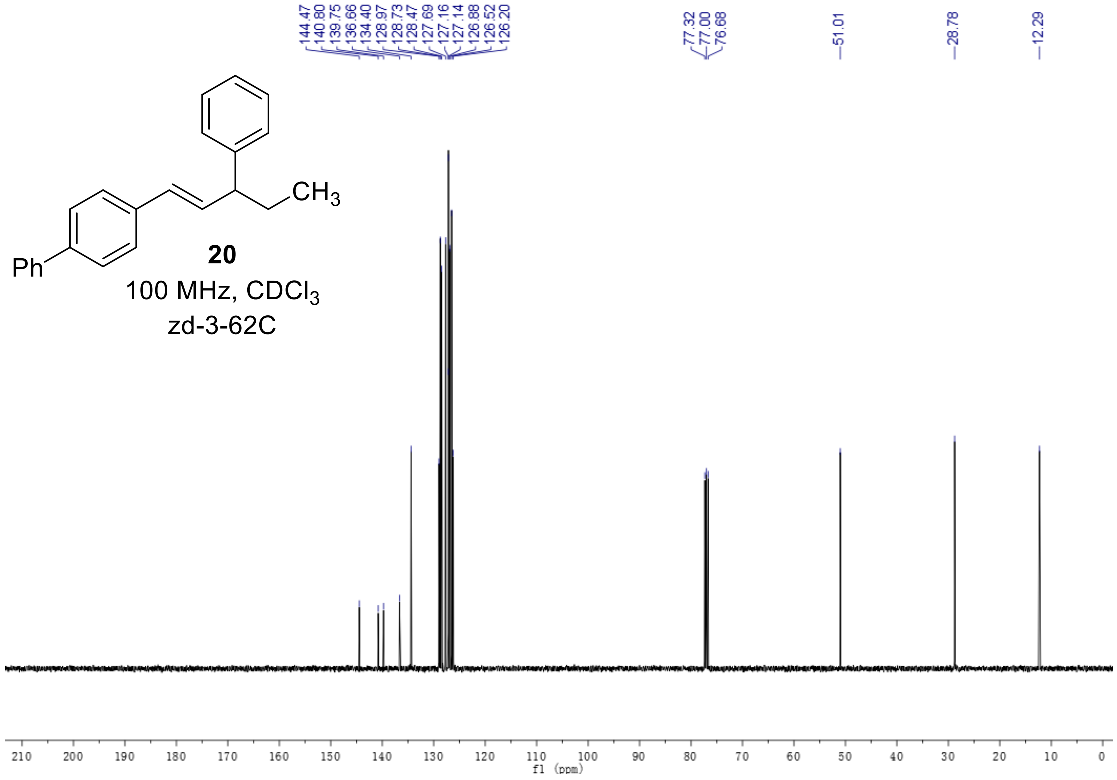

Supplementary Figure 134. ^{13}C NMR (100 MHz, $CDCl_3$, 25 °C) spectra for **20**

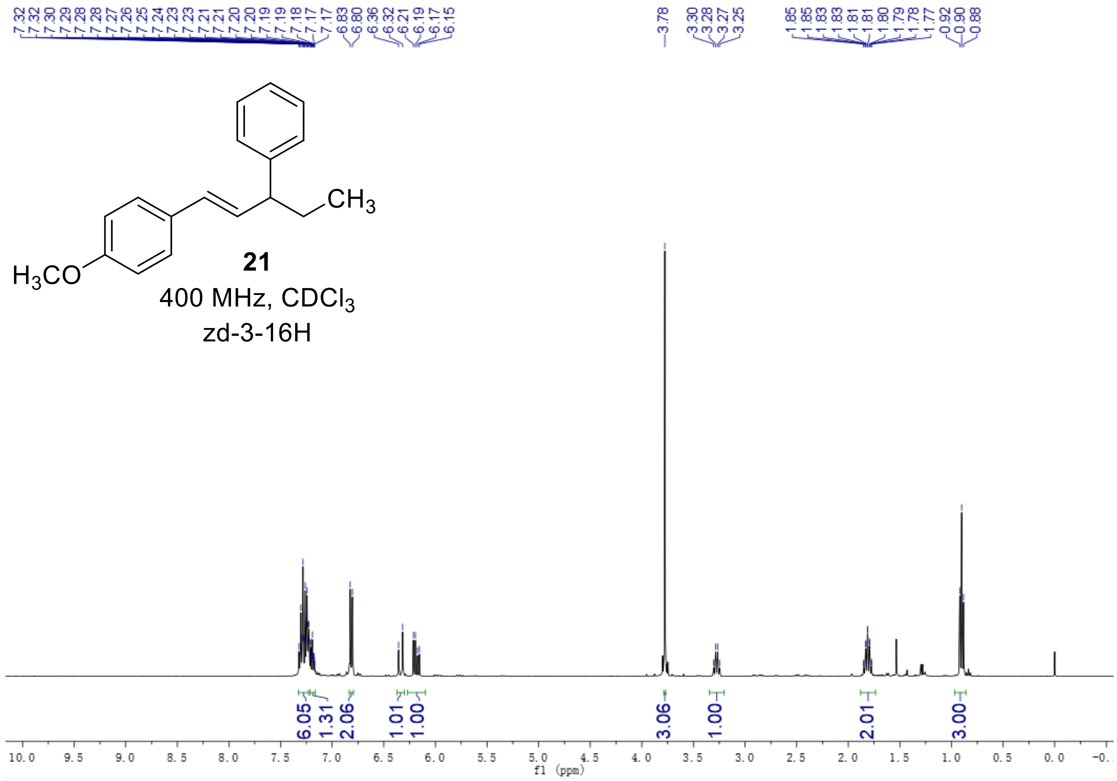

Supplementary Figure 135. ^{1}H NMR (400 MHz, $CDCl_3$, 25 °C) spectra for **21**

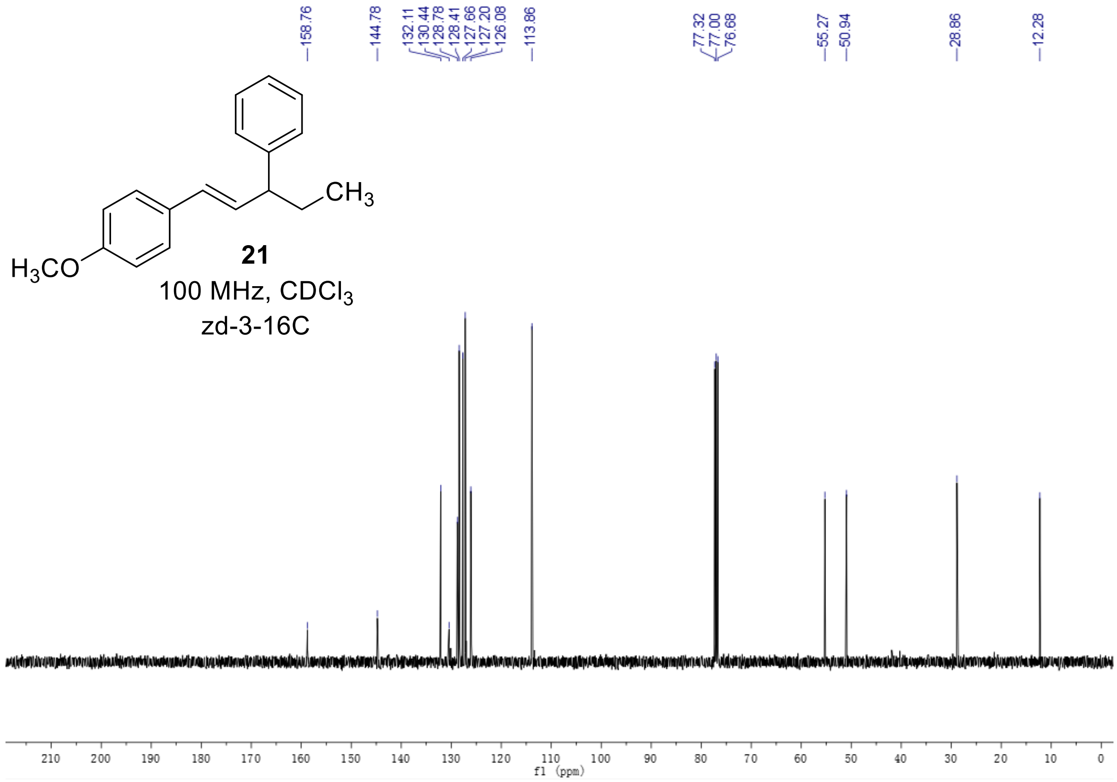

Supplementary Figure 136. ^{13}C NMR (100 MHz, $CDCl_3$, 25 °C) spectra for **21**

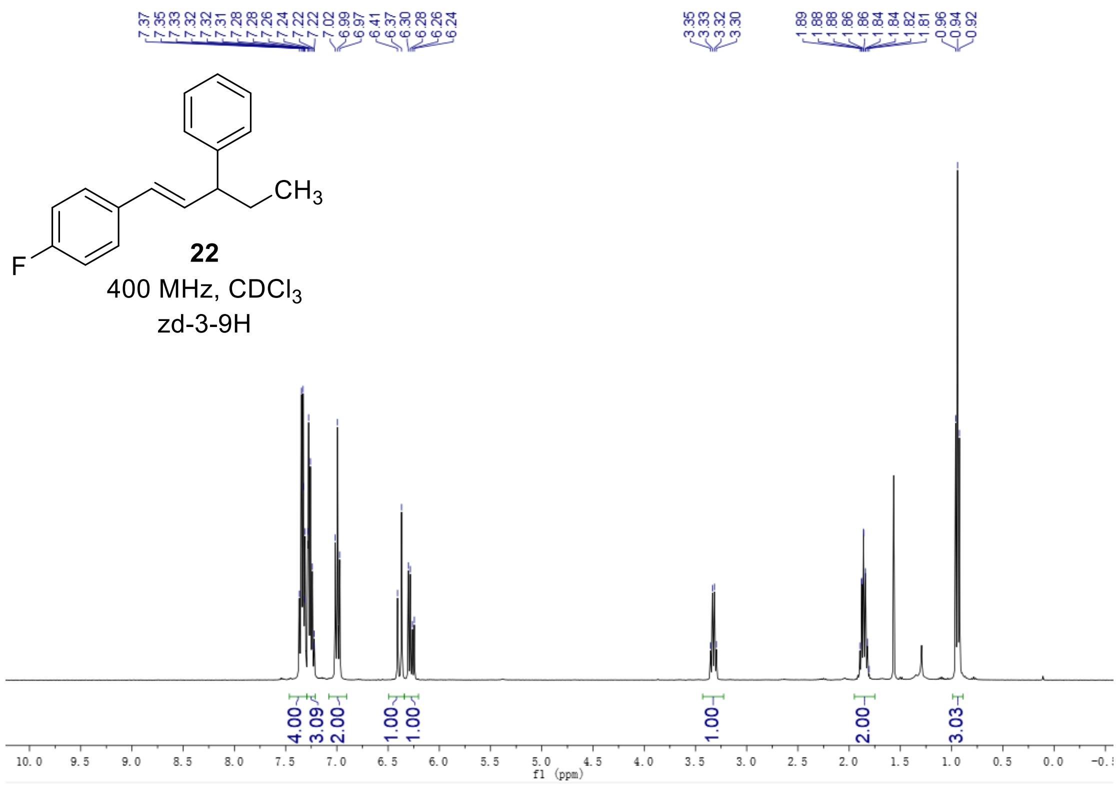

Supplementary Figure 137. ^{1}H NMR (400 MHz, $CDCl_3$, 25 °C) spectra for **22**

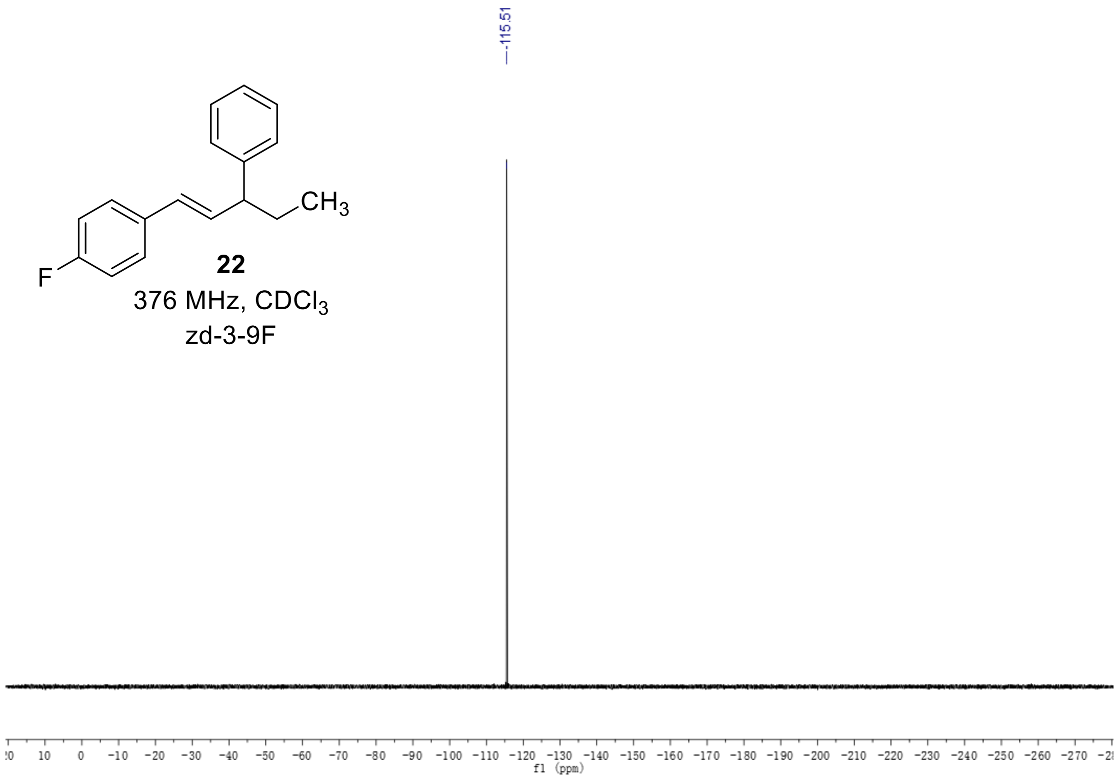

Supplementary Figure 138. ^{19}F NMR (376 MHz, $CDCl_3$, 25 °C) spectra for **22**

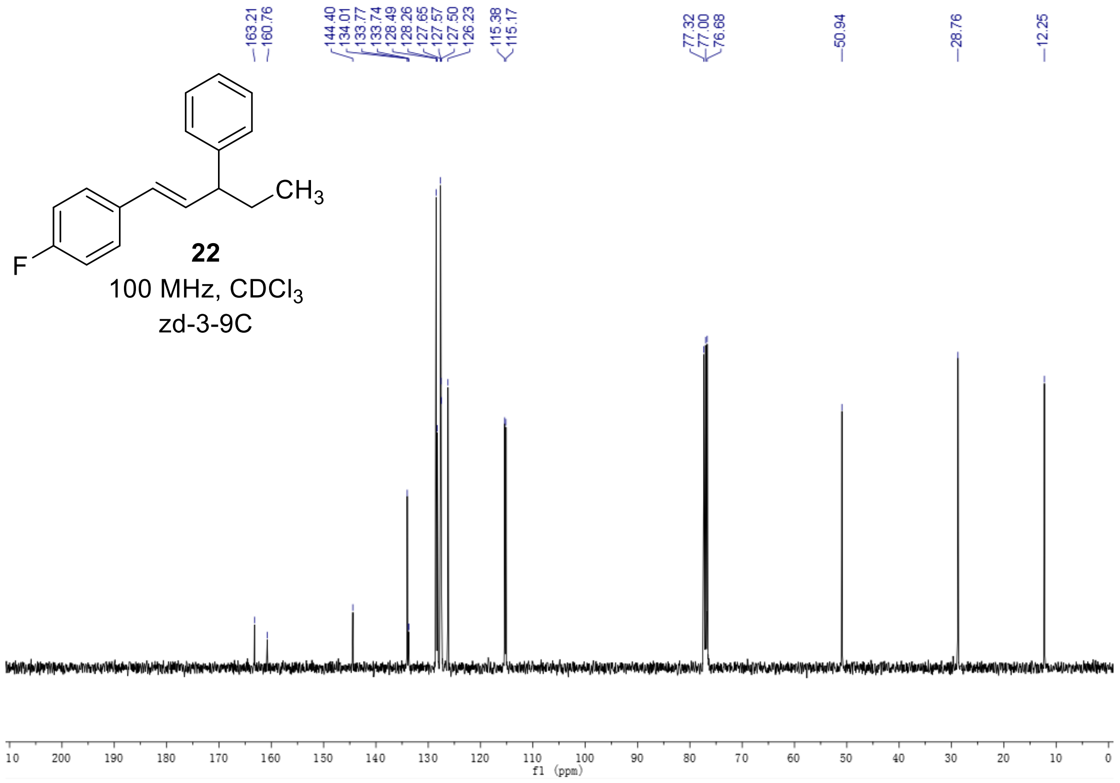

Supplementary Figure 139. ^{13}C NMR (100 MHz, $CDCl_3$, 25 °C) spectra for **22**

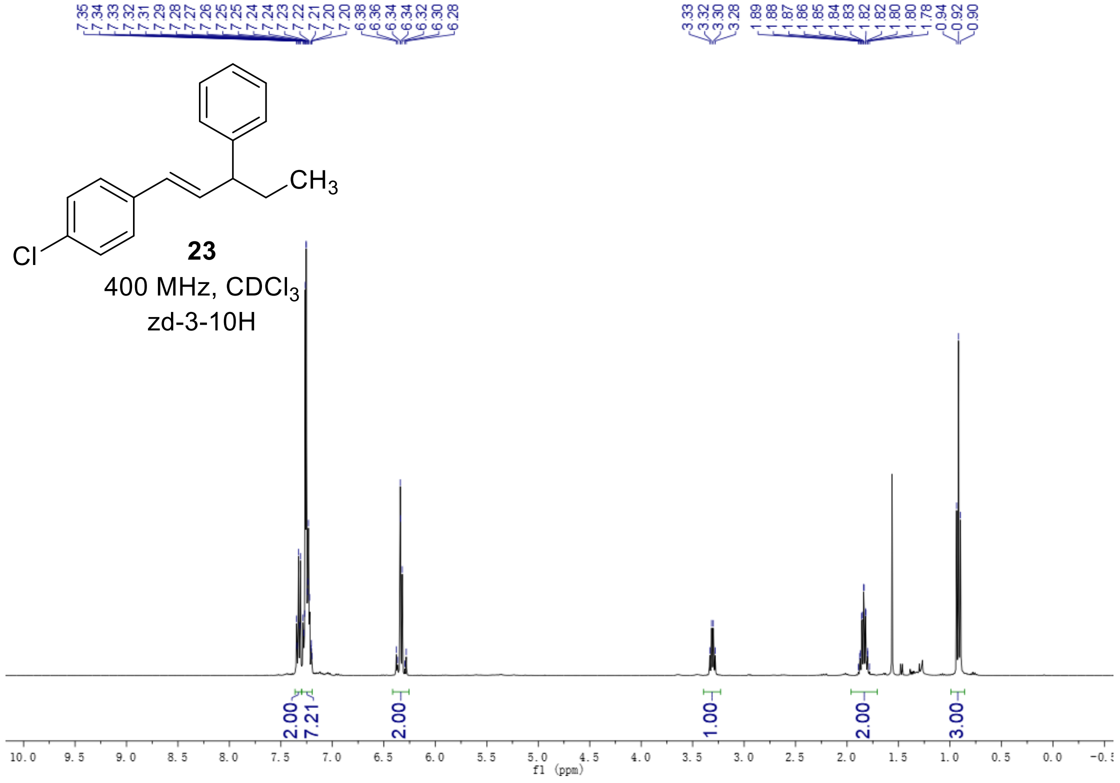

Supplementary Figure 140. ^{1}H NMR (400 MHz, $CDCl_3$, 25 °C) spectra for **23**

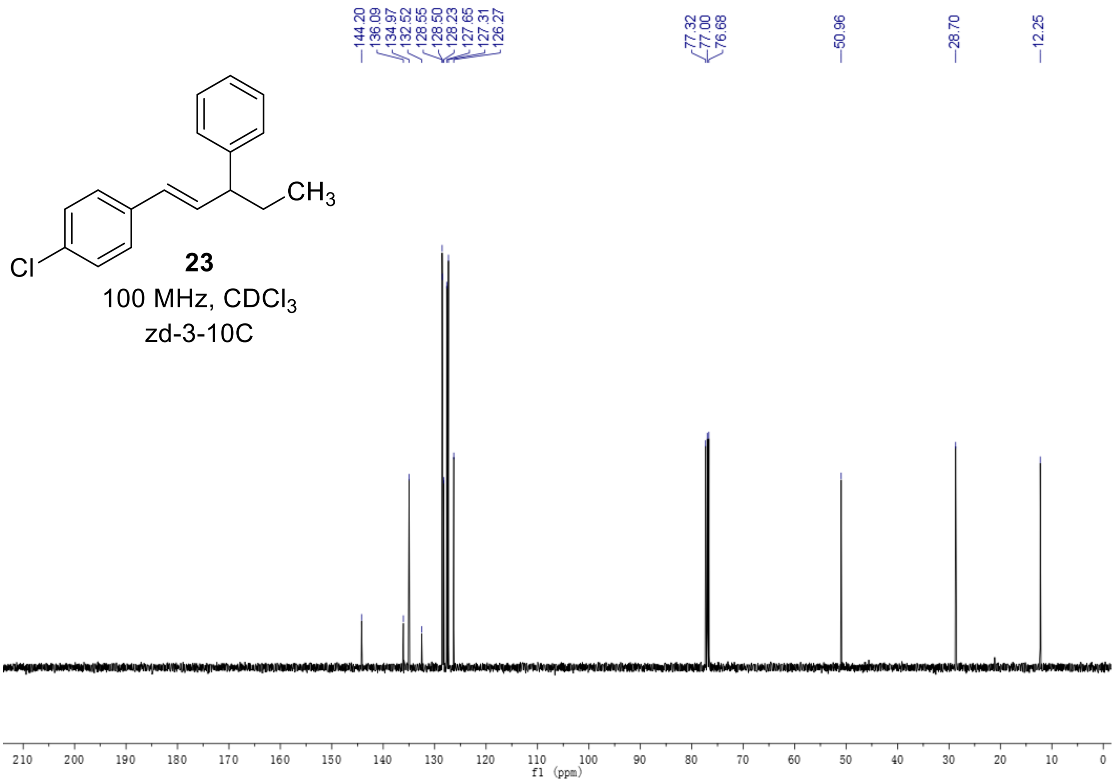

Supplementary Figure 141. ^{13}C NMR (100 MHz, $CDCl_3$, 25 °C) spectra for **23**

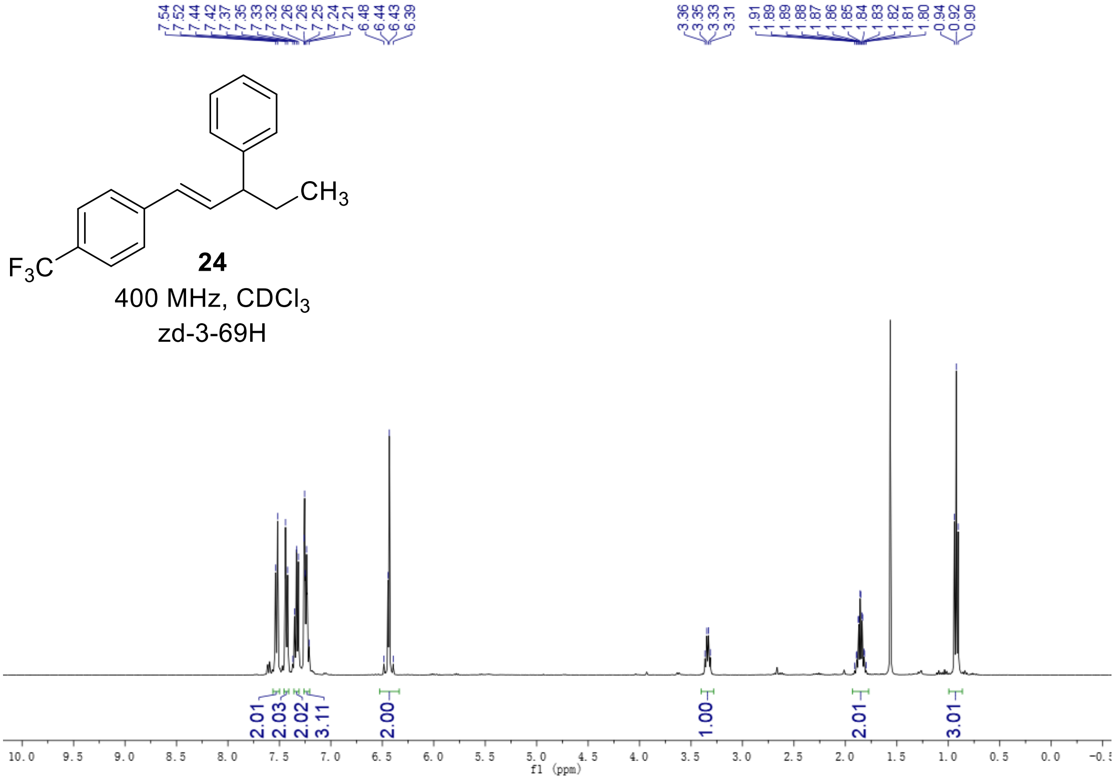

Supplementary Figure 142. ^{1}H NMR (400 MHz, $CDCl_3$, 25 °C) spectra for **24**

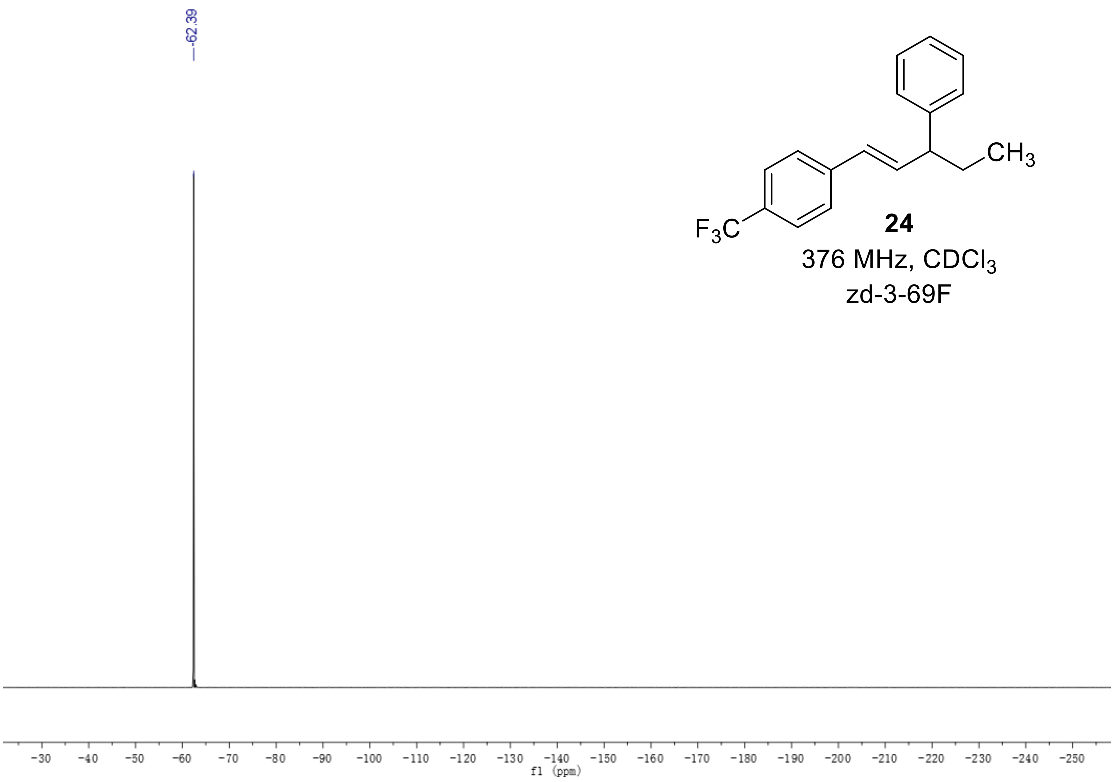

Supplementary Figure 143. ^{1}H NMR (400 MHz, $CDCl_3$, 25 °C) spectra for **24**

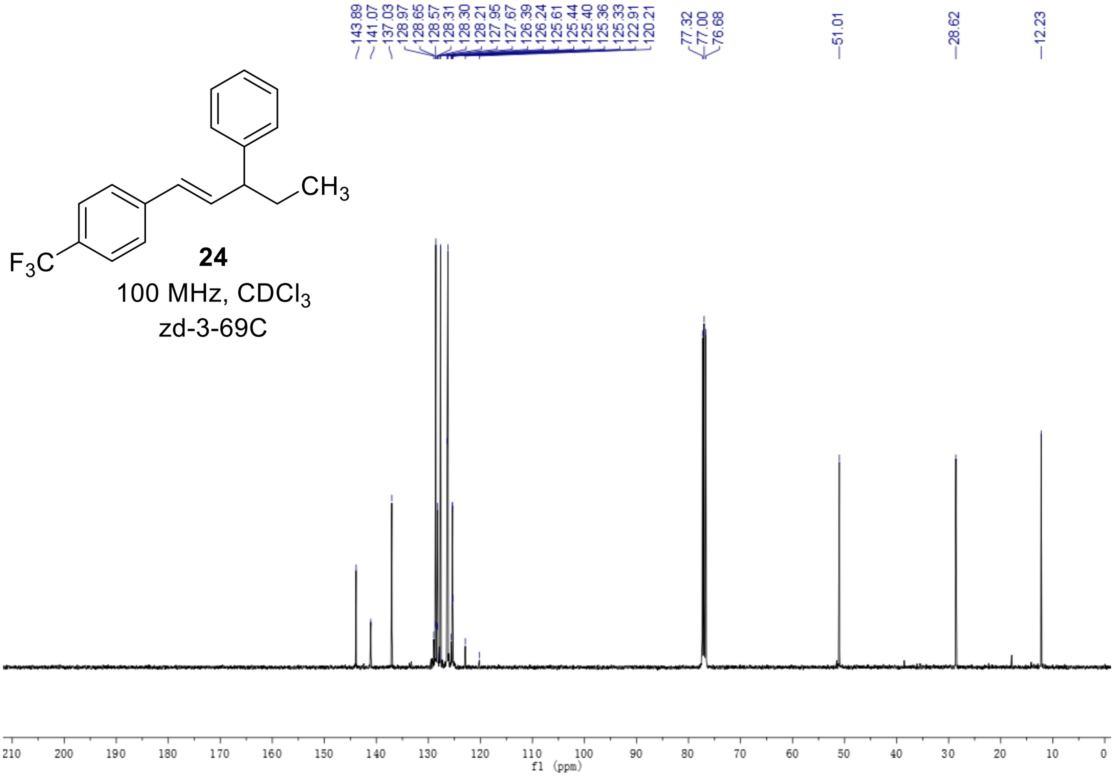

Supplementary Figure 144. ^{13}C NMR (100 MHz, $CDCl_3$, 25 °C) spectra for **24**

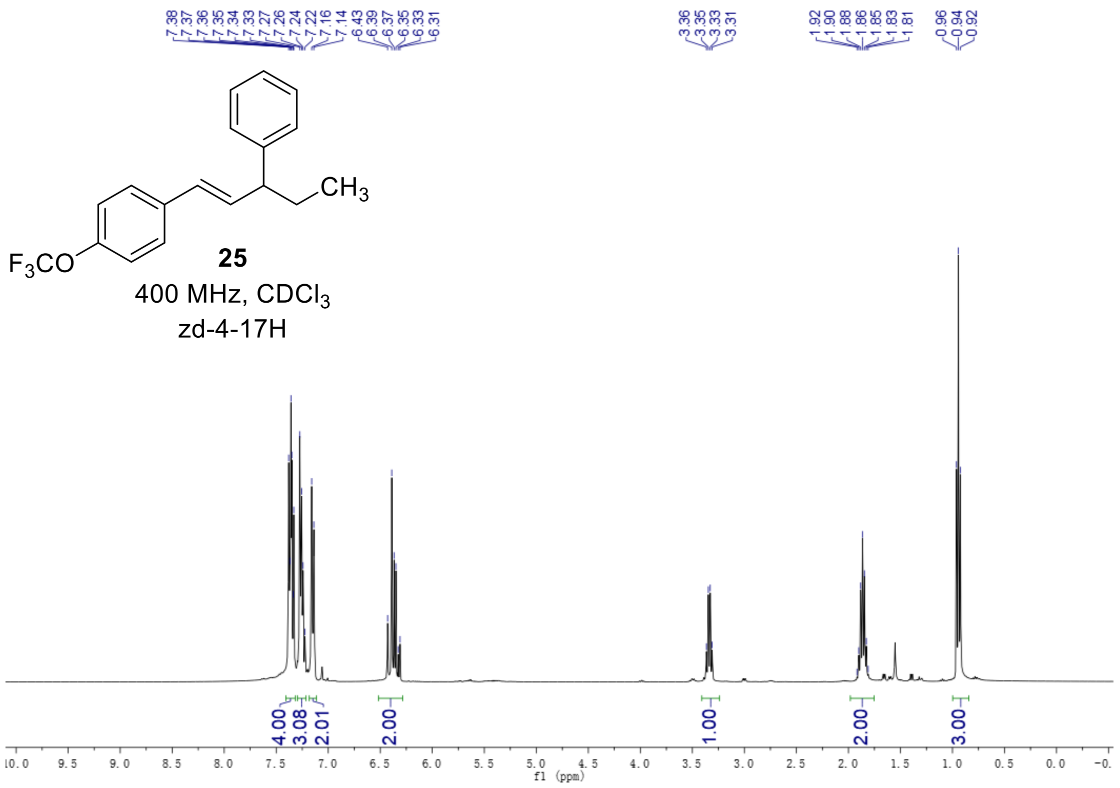

Supplementary Figure 145. ^{1}H NMR (400 MHz, $CDCl_3$, 25 °C) spectra for **25**

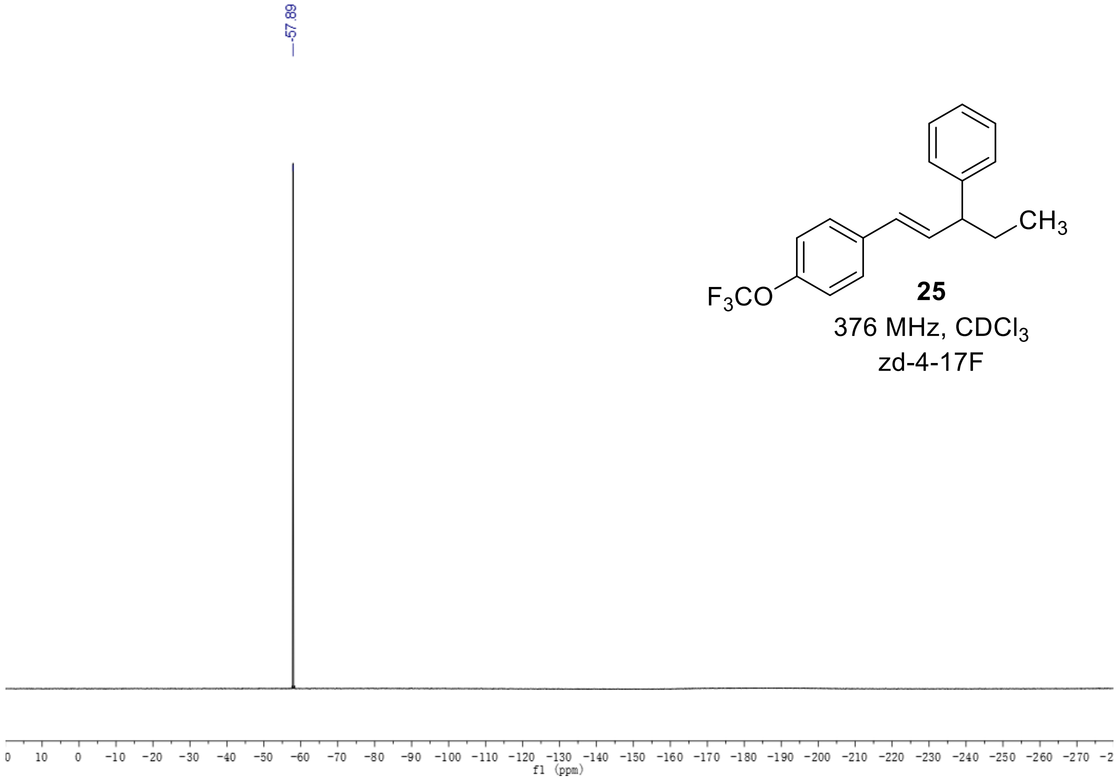

Supplementary Figure 146. ^{19}F NMR (376 MHz, $CDCl_3$, 25 °C) spectra for **25**

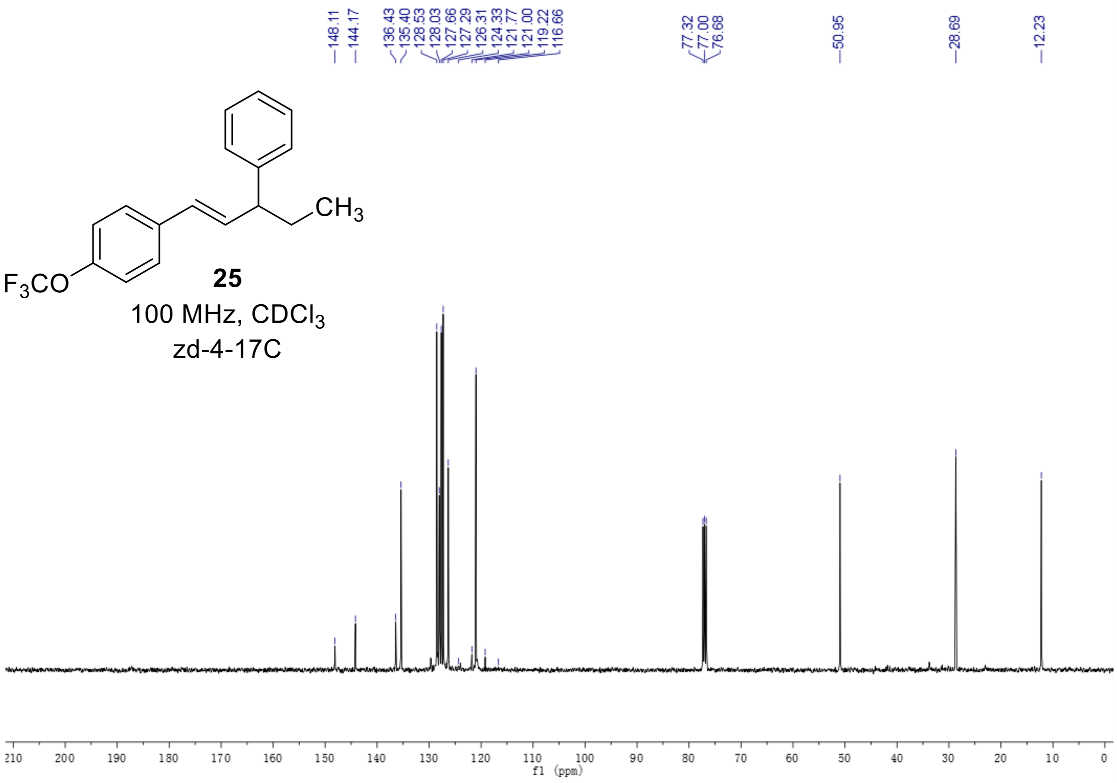

Supplementary Figure 147. ^{13}C NMR (100 MHz, $CDCl_3$, 25 °C) spectra for **25**

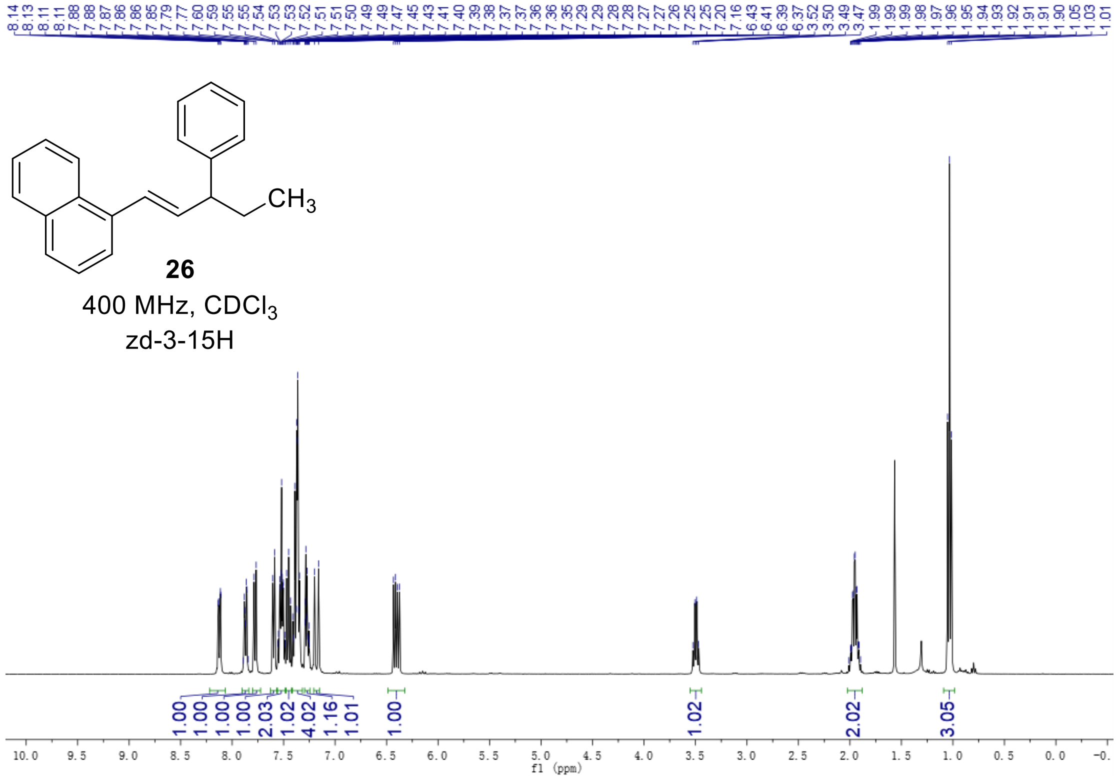

Supplementary Figure 148. ^{1}H NMR (400 MHz, $CDCl_3$, 25 °C) spectra for **26**

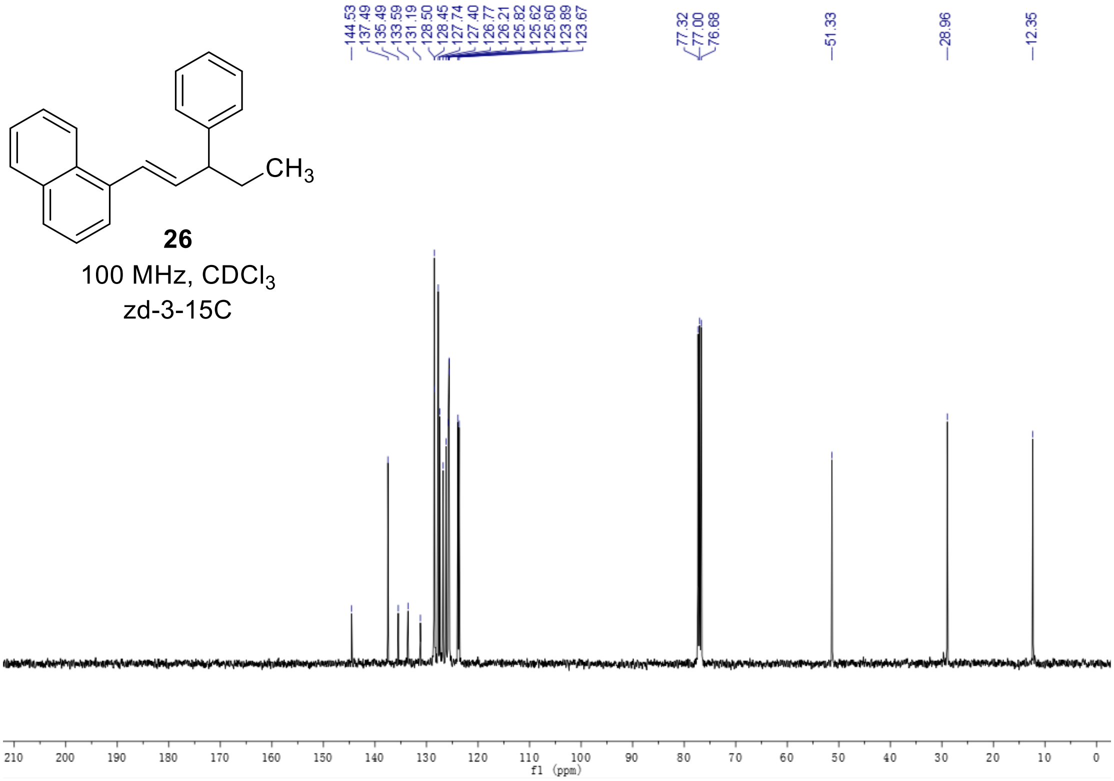

Supplementary Figure 149. ^{13}C NMR (100 MHz, $CDCl_3$, 25 °C) spectra for **26**

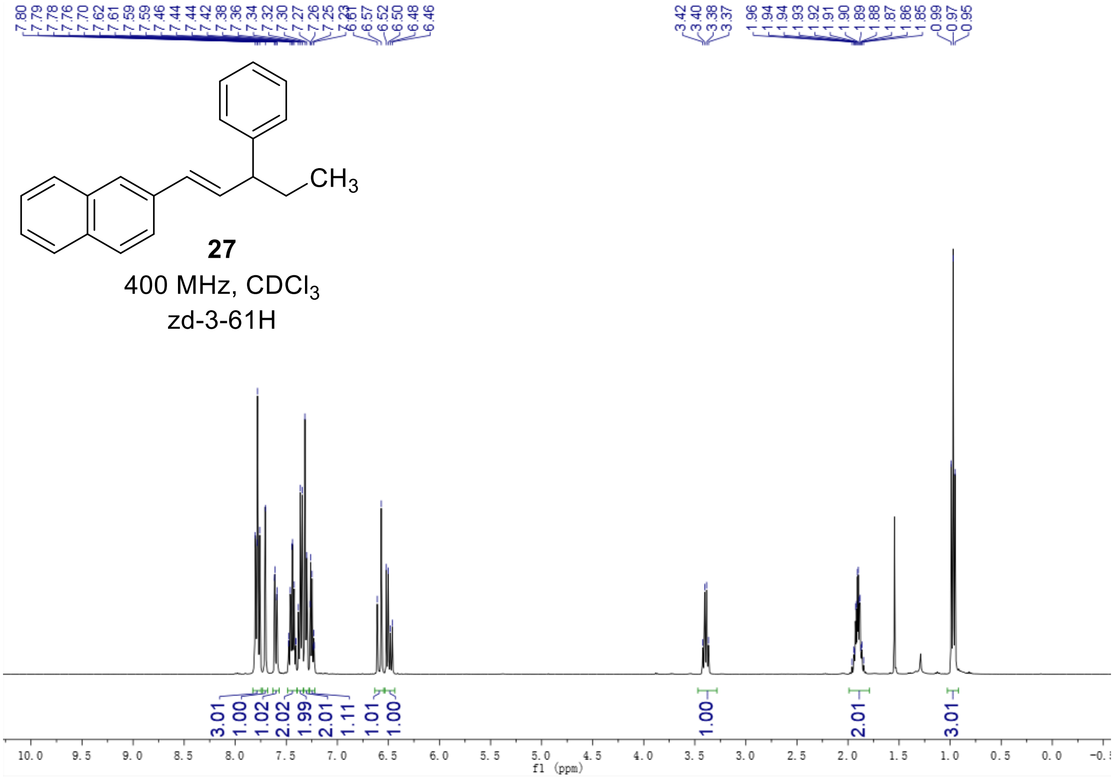

Supplementary Figure 150. ^{1}H NMR (400 MHz, $CDCl_3$, 25 °C) spectra for **27**

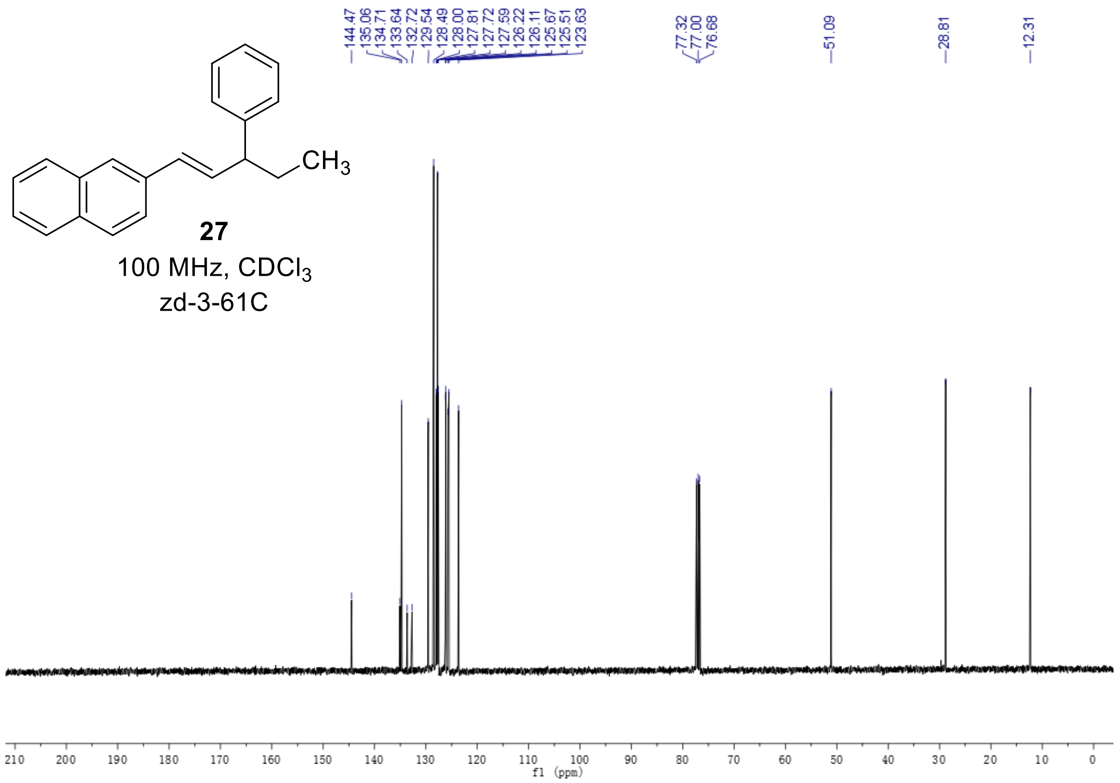

Supplementary Figure 151. ^{13}C NMR (100 MHz, $CDCl_3$, 25 °C) spectra for **27**

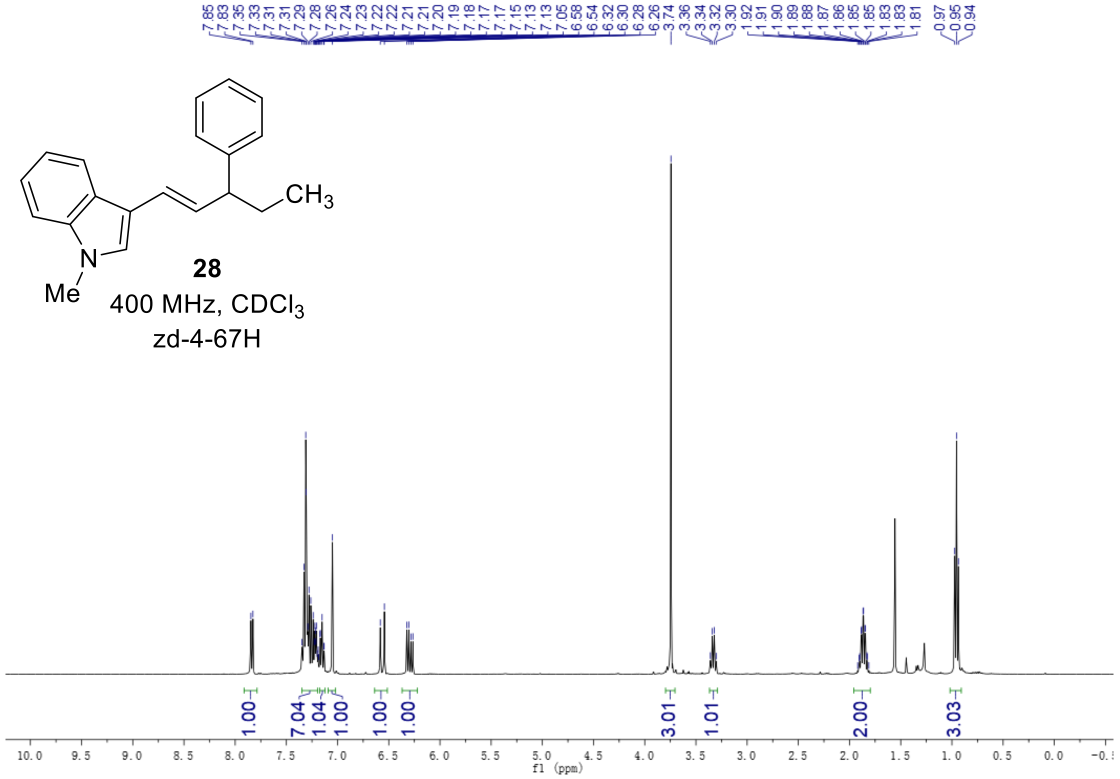

Supplementary Figure 152. ^{1}H NMR (400 MHz, $CDCl_3$, 25 °C) spectra for **28**

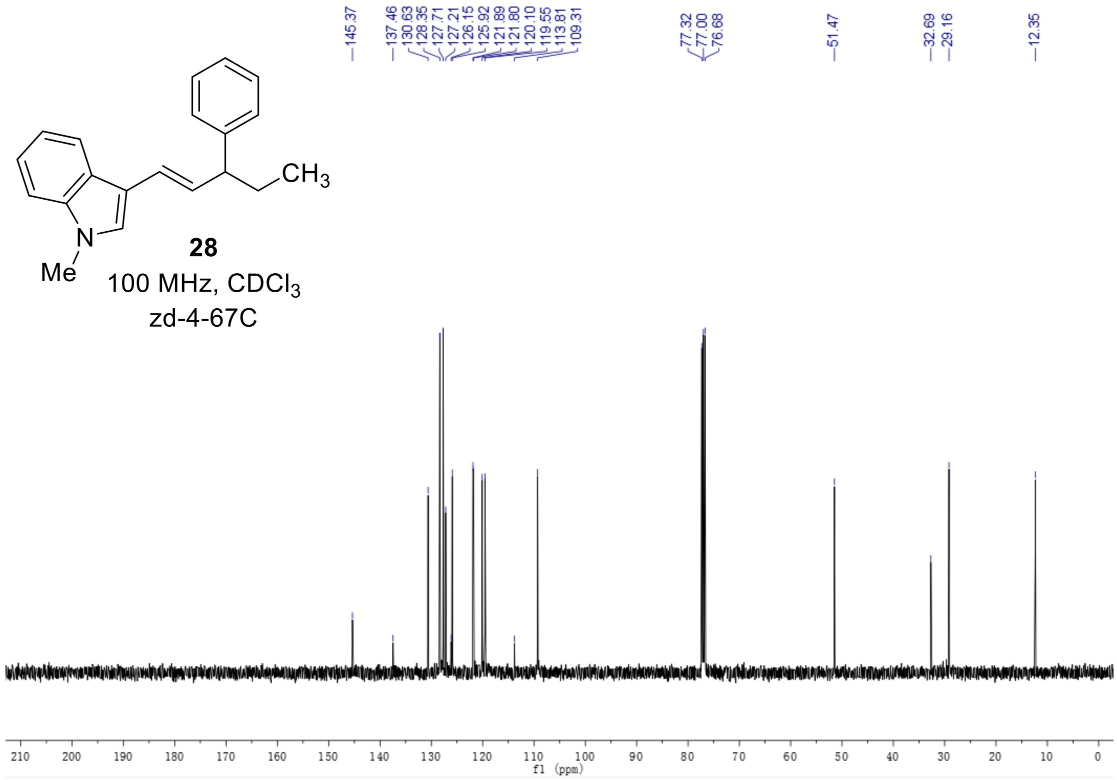

Supplementary Figure 153. ^{13}C NMR (100 MHz, $CDCl_3$, 25 °C) spectra for **28**

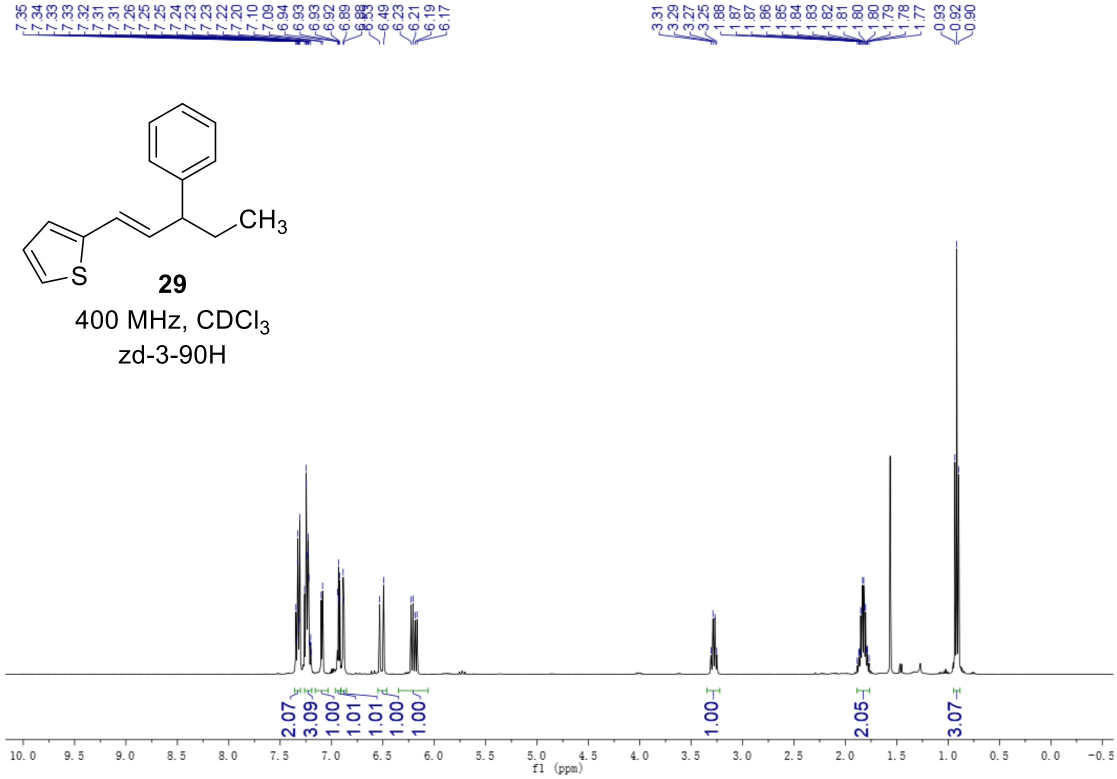

Supplementary Figure 154. ^{1}H NMR (400 MHz, $CDCl_3$, 25 °C) spectra for **29**

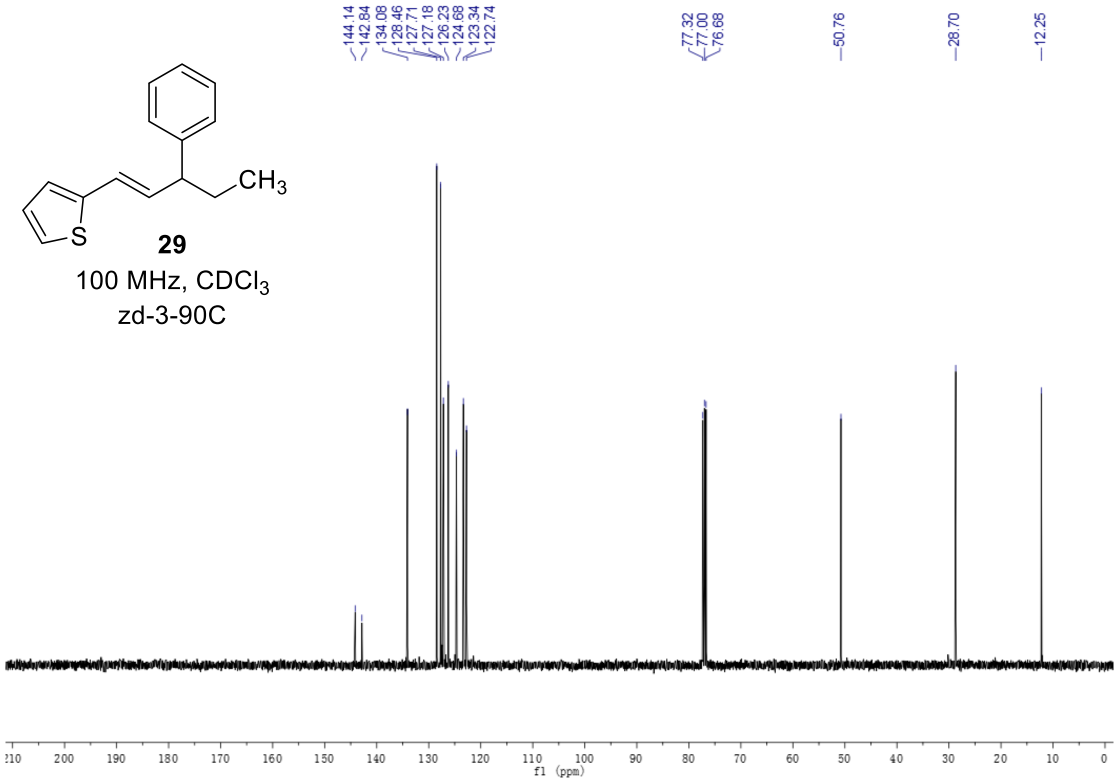

Supplementary Figure 155. ^{13}C NMR (100 MHz, $CDCl_3$, 25 °C) spectra for **29**

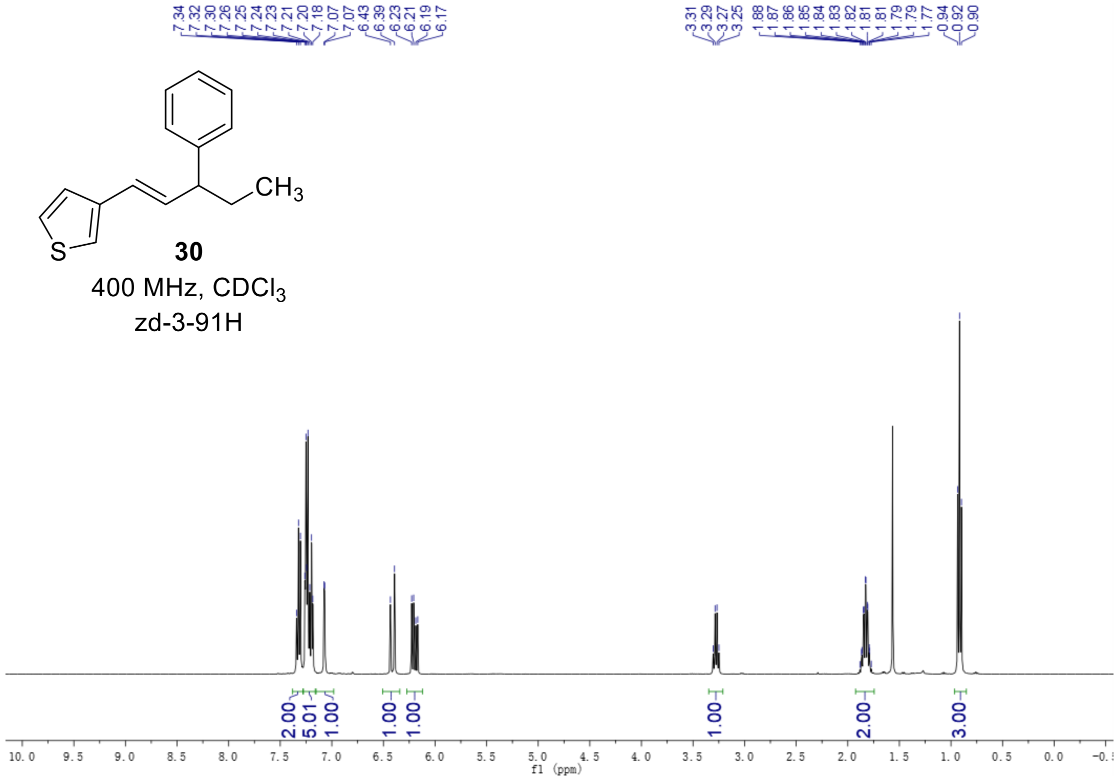

Supplementary Figure 156. ^{1}H NMR (400 MHz, $CDCl_3$, 25 °C) spectra for **30**

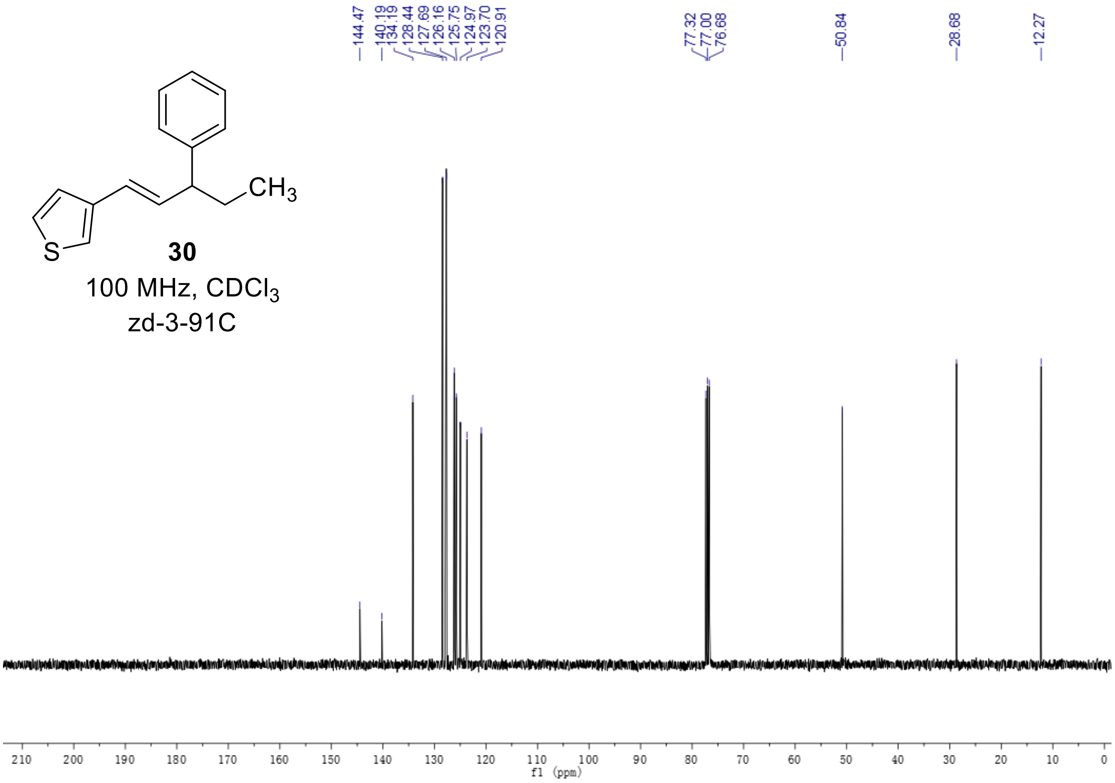

Supplementary Figure 157. ^{13}C NMR (100 MHz, $CDCl_3$, 25 °C) spectra for **30**

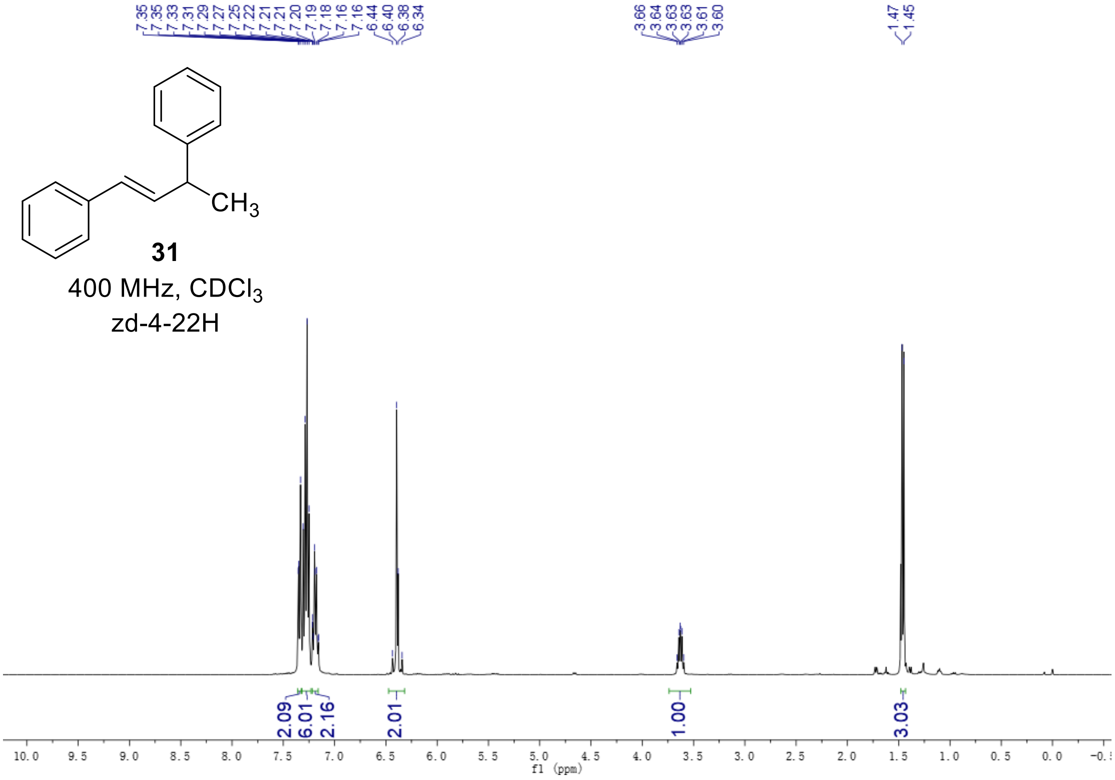

Supplementary Figure 158. ^{1}H NMR (400 MHz, $CDCl_3$, 25 °C) spectra for **31**

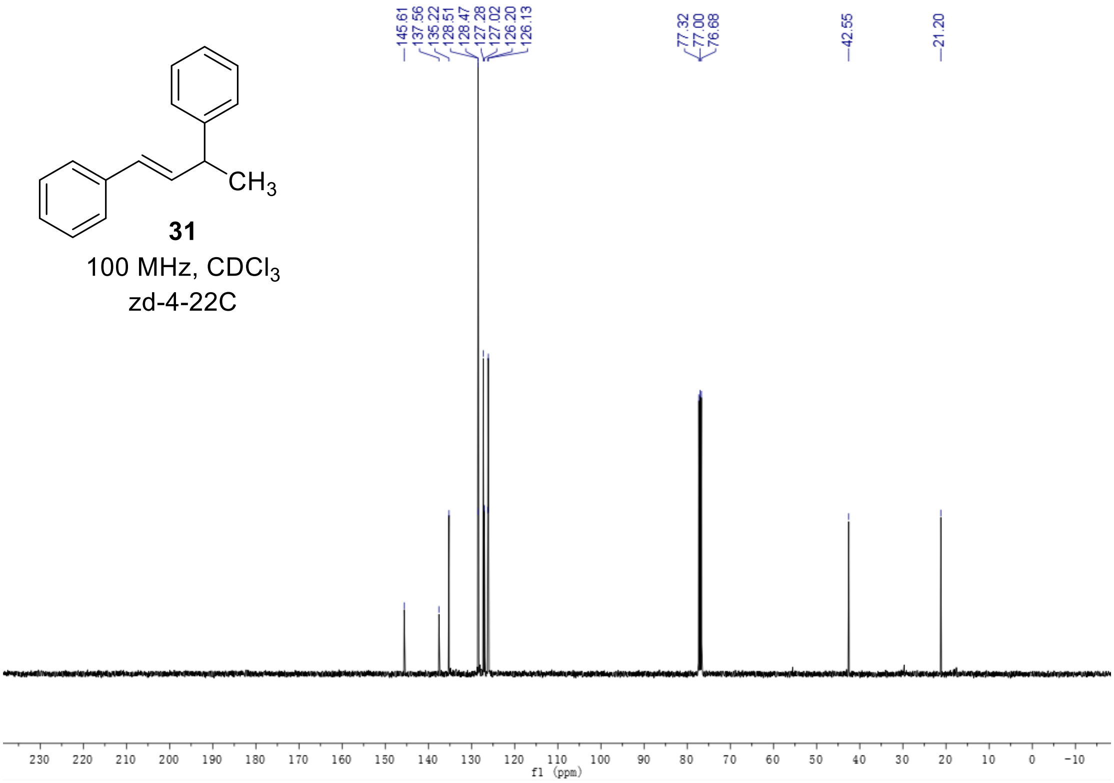

Supplementary Figure 159. ^{13}C NMR (100 MHz, $CDCl_3$, 25 °C) spectra for **31**

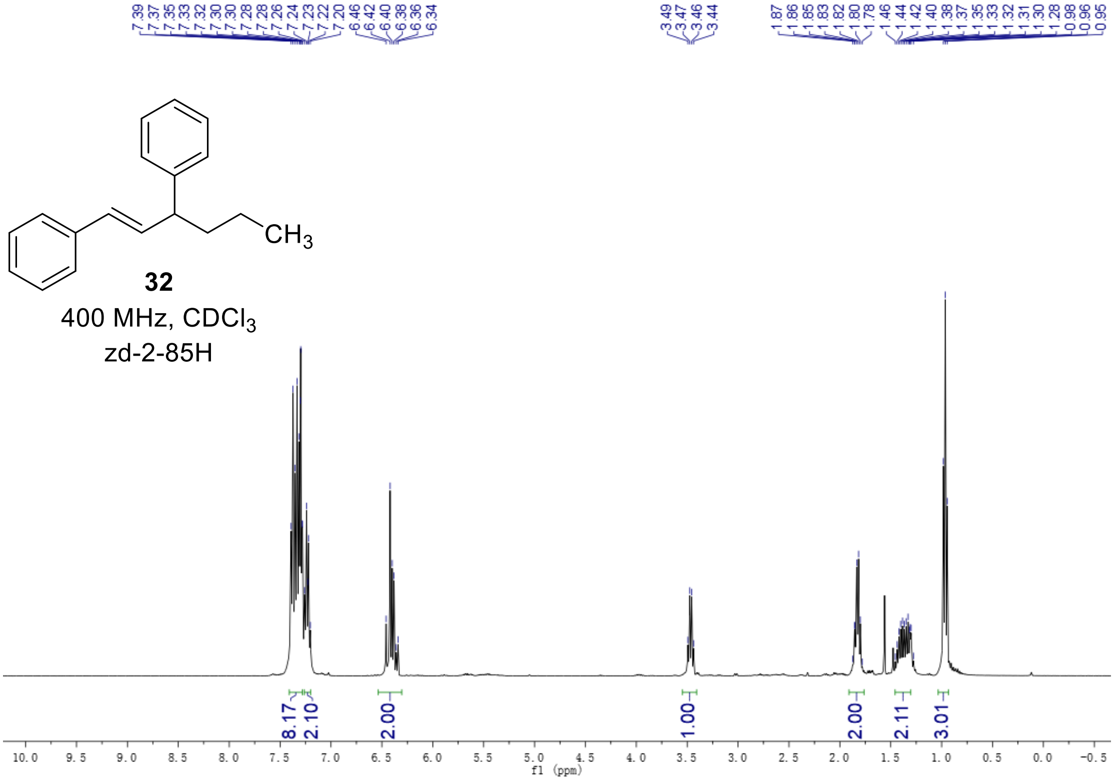

Supplementary Figure 160. ^{1}H NMR (400 MHz, $CDCl_3$, 25 °C) spectra for **32**

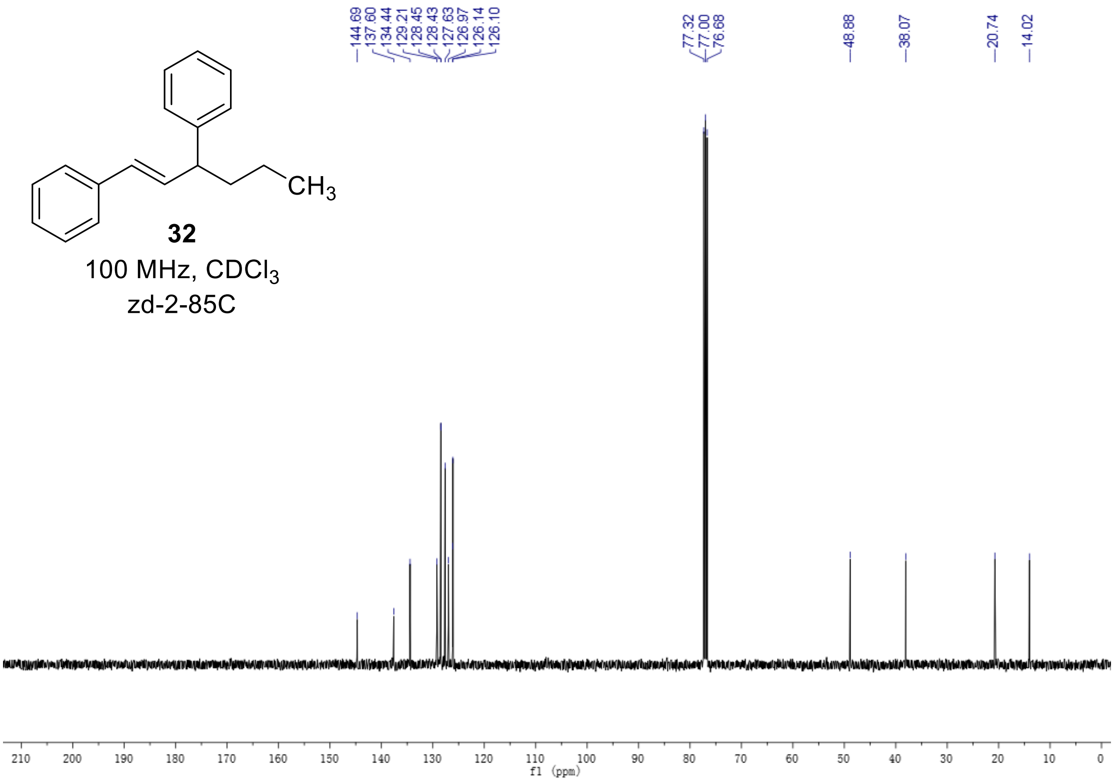

Supplementary Figure 161. ^{13}C NMR (100 MHz, $CDCl_3$, 25 °C) spectra for **32**

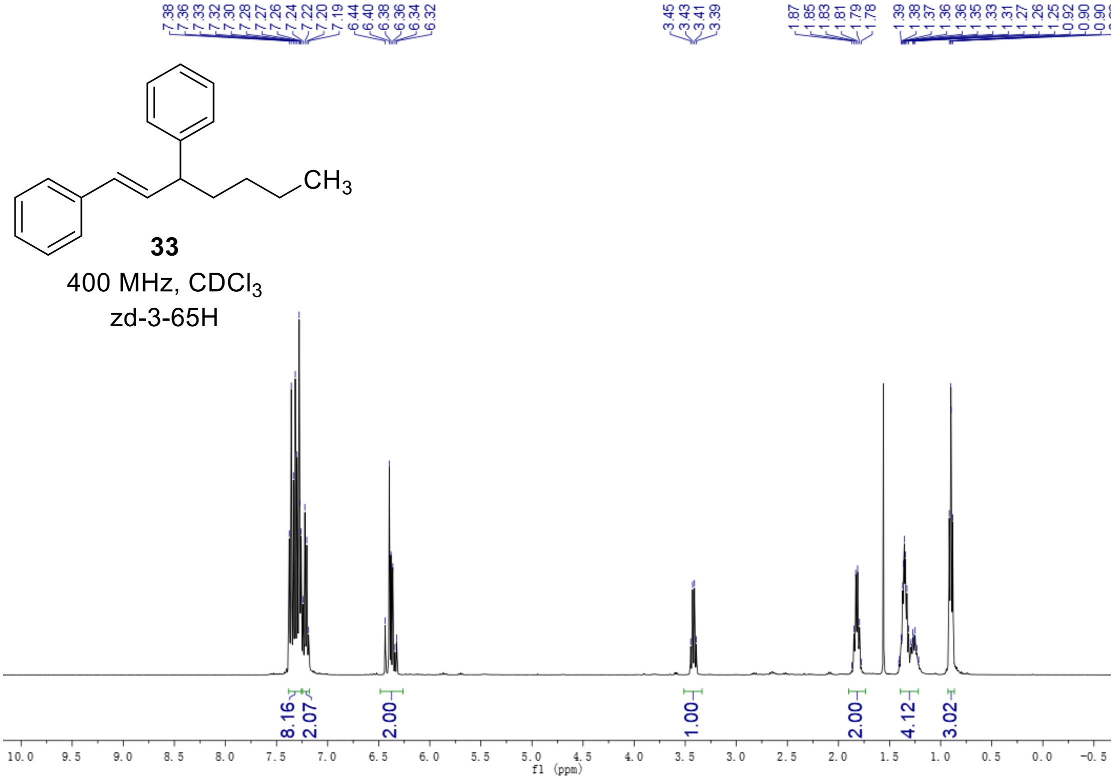

Supplementary Figure 162. ^{1}H NMR (400 MHz, $CDCl_3$, 25 °C) spectra for **33**

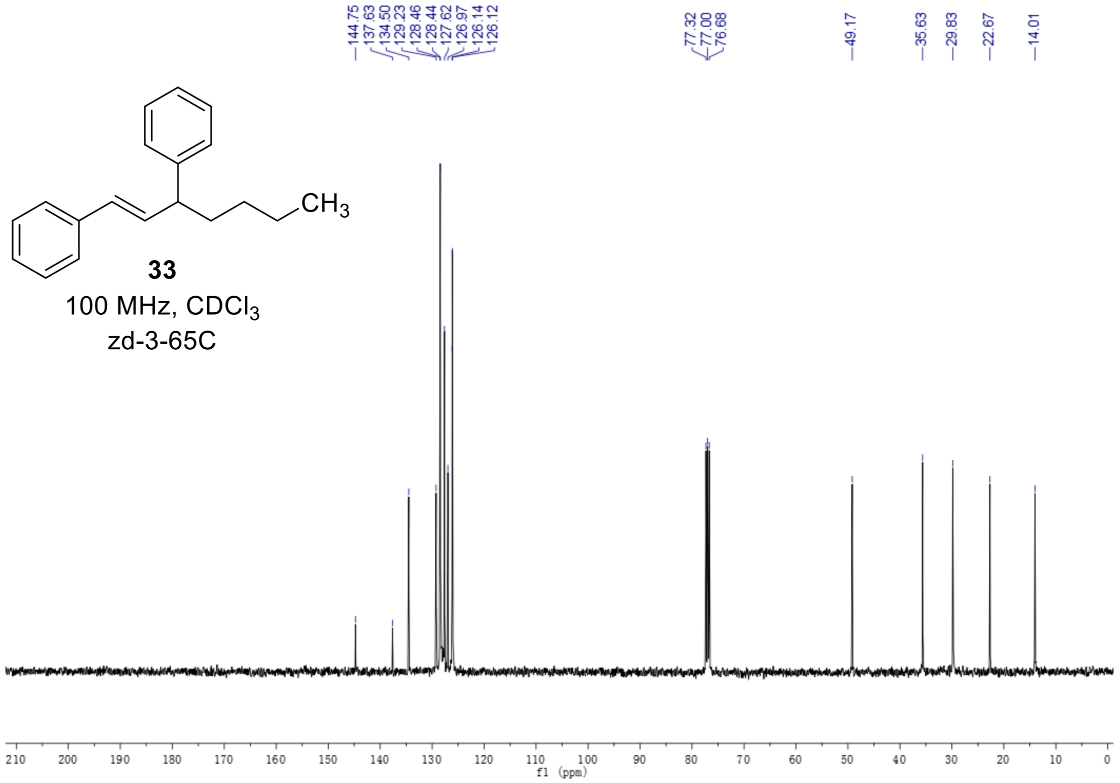

Supplementary Figure 163. ^{13}C NMR (100 MHz, $CDCl_3$, 25 °C) spectra for **33**

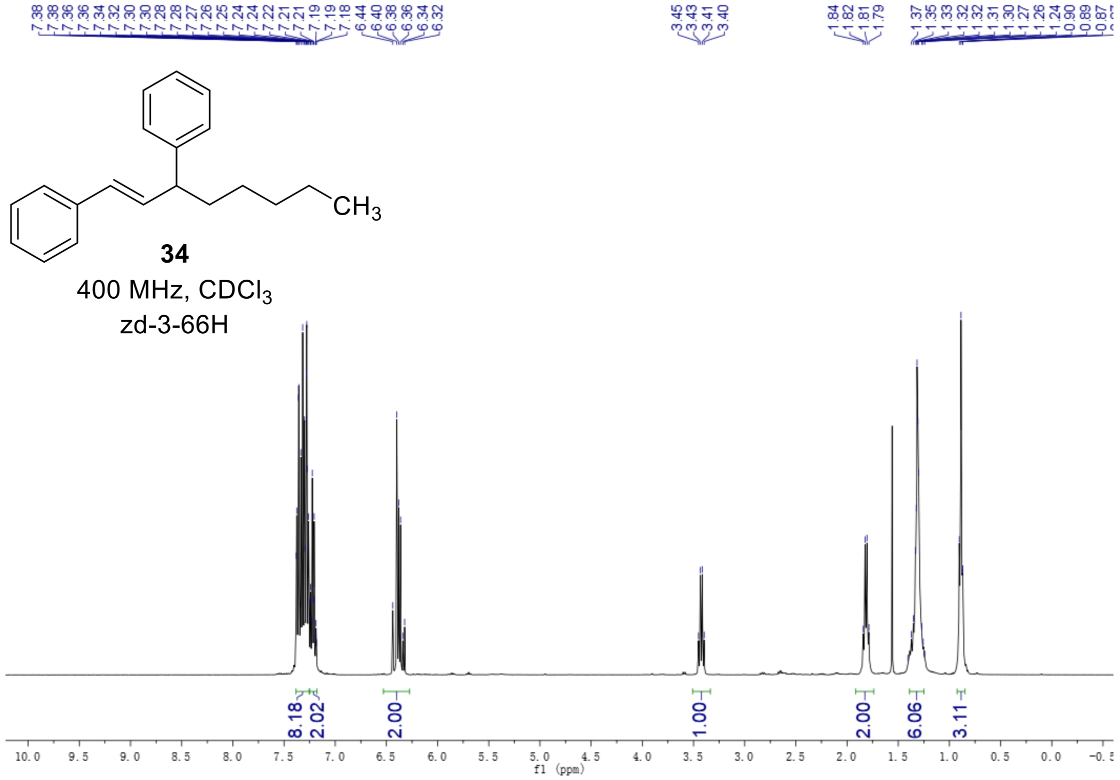

Supplementary Figure 164. ^{1}H NMR (400 MHz, $CDCl_3$, 25 °C) spectra for **34**

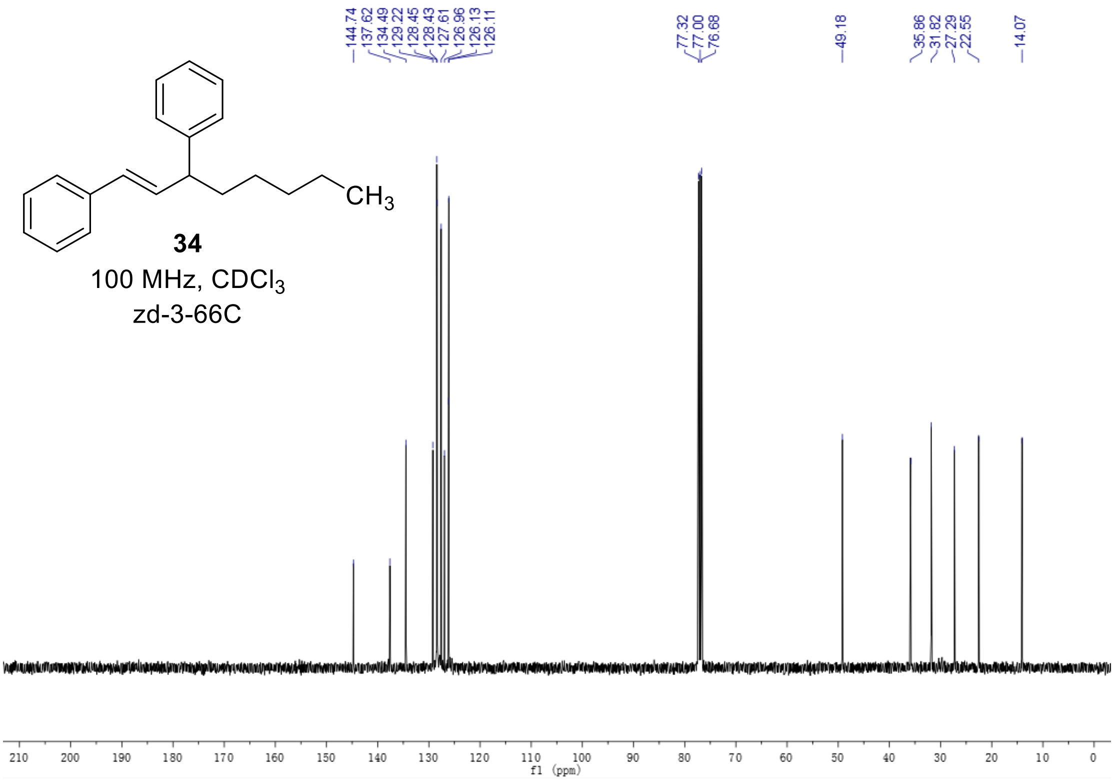

Supplementary Figure 165. ^{13}C NMR (100 MHz, $CDCl_3$, 25 °C) spectra for **34**

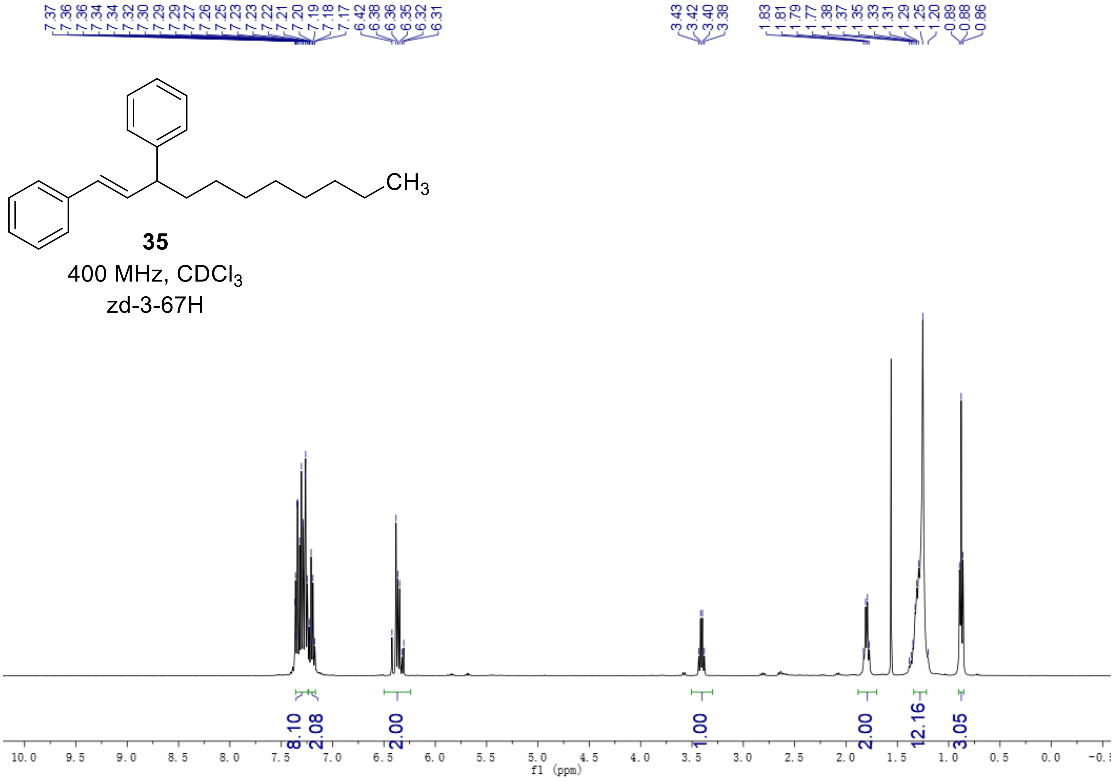

Supplementary Figure 166. ^{1}H NMR (400 MHz, $CDCl_3$, 25 °C) spectra for **35**

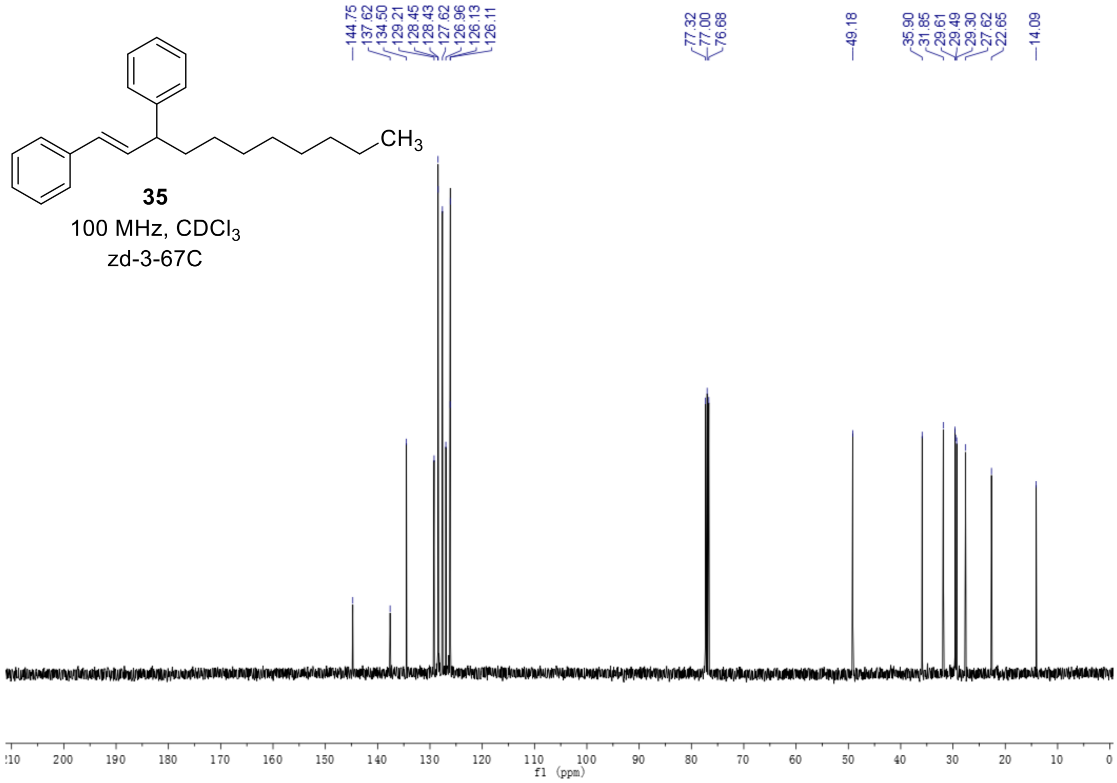

Supplementary Figure 167. ^{13}C NMR (100 MHz, $CDCl_3$, 25 °C) spectra for **35**

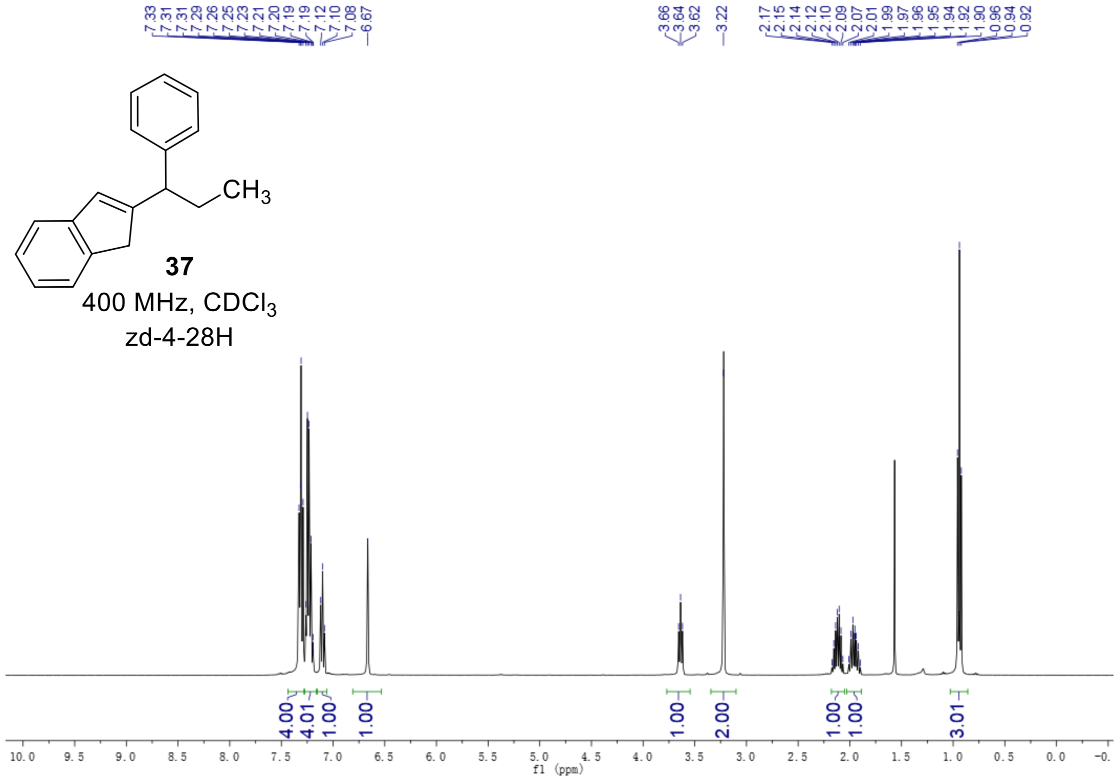

Supplementary Figure 168. ^{1}H NMR (400 MHz, $CDCl_3$, 25 °C) spectra for **37**

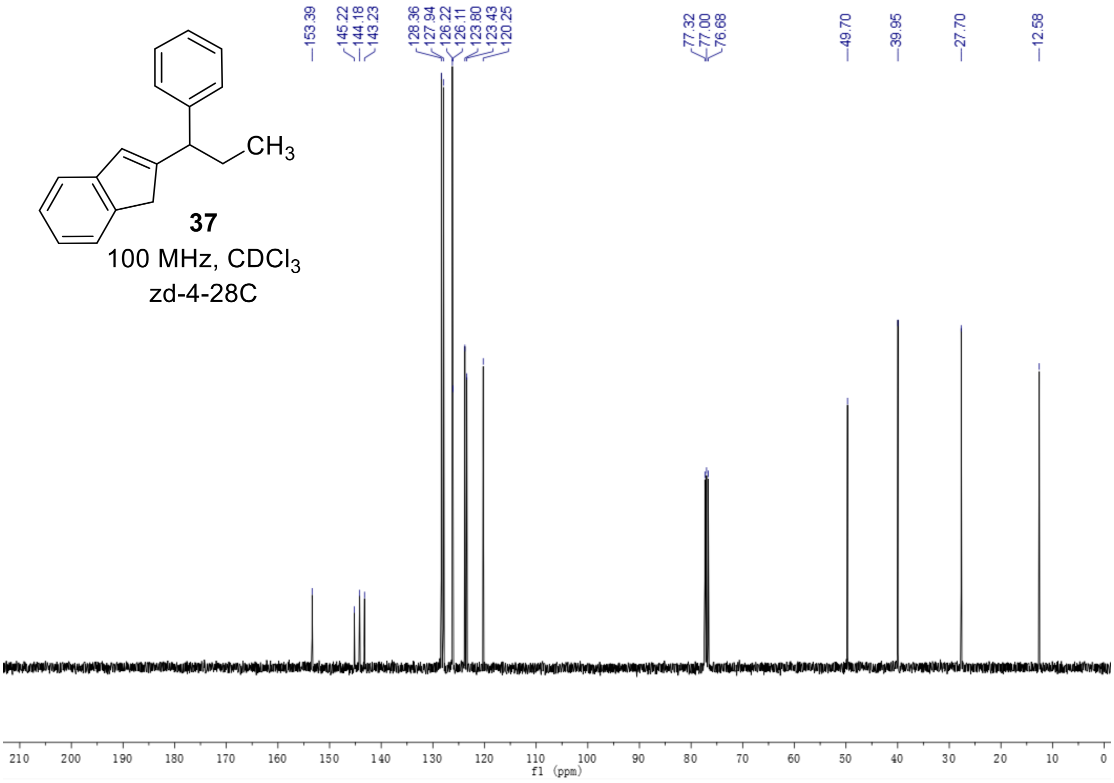

Supplementary Figure 169. ^{13}C NMR (100 MHz, $CDCl_3$, 25 °C) spectra for **37**

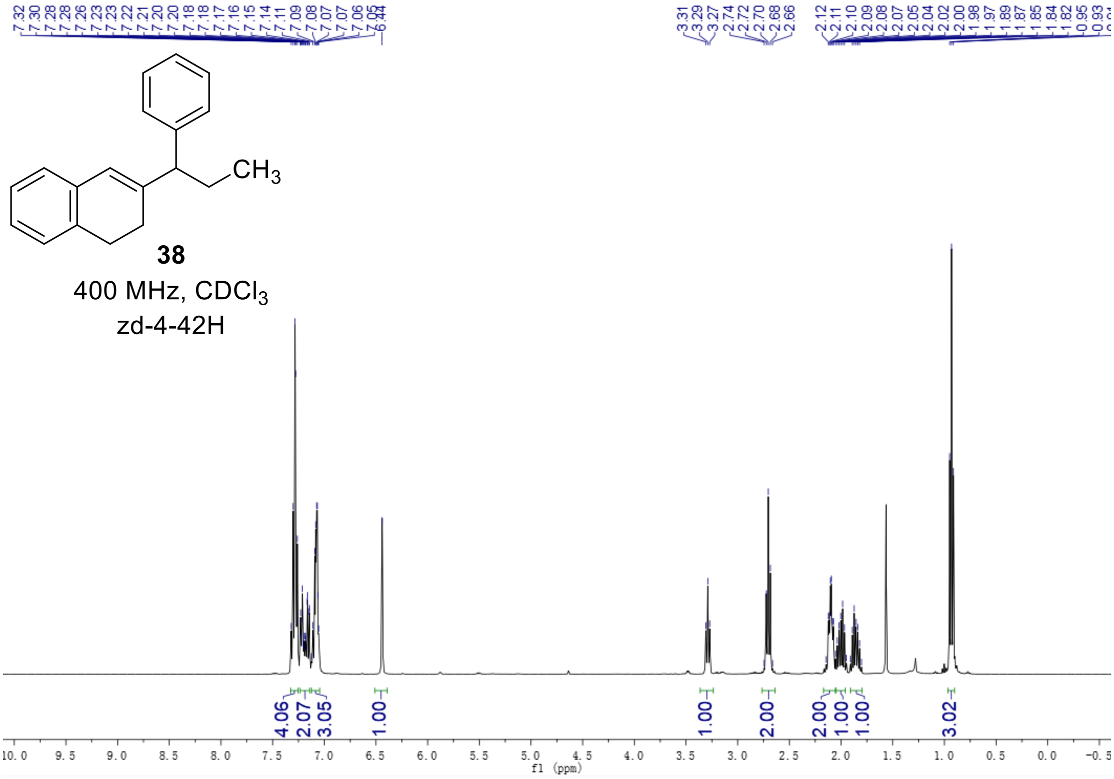

Supplementary Figure 170. ^{1}H NMR (400 MHz, $CDCl_3$, 25 °C) spectra for **38**

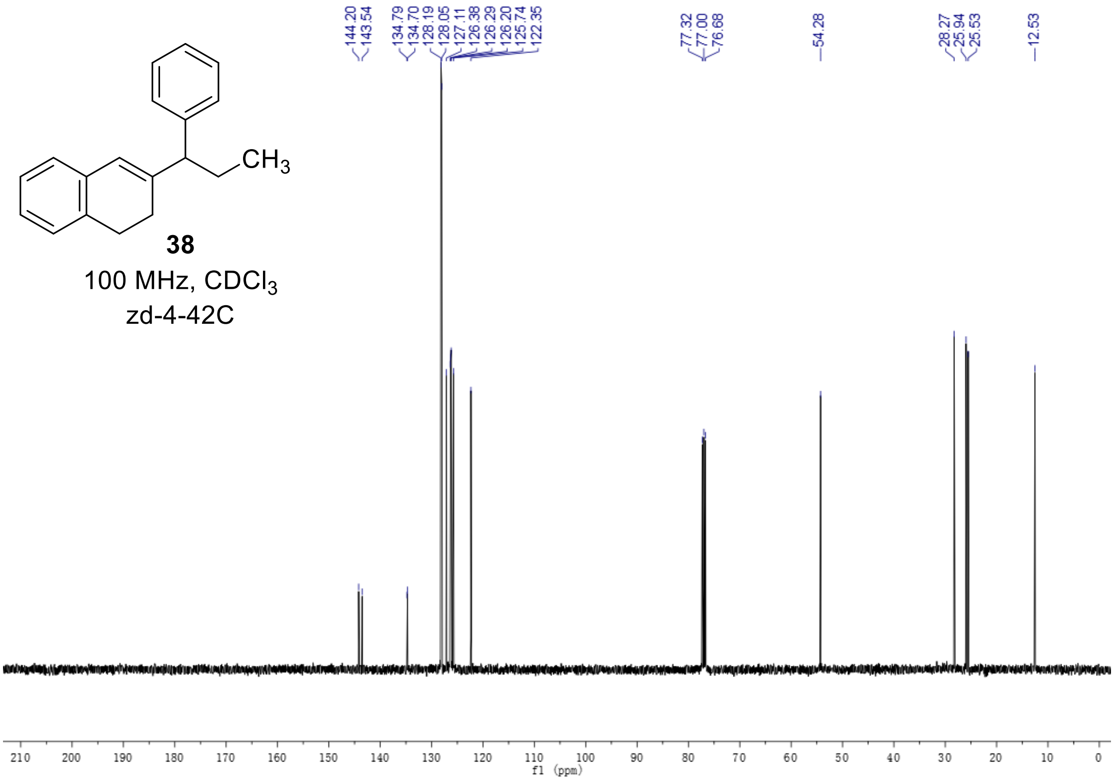

Supplementary Figure 171. ^{13}C NMR (100 MHz, $CDCl_3$, 25 °C) spectra for **38**

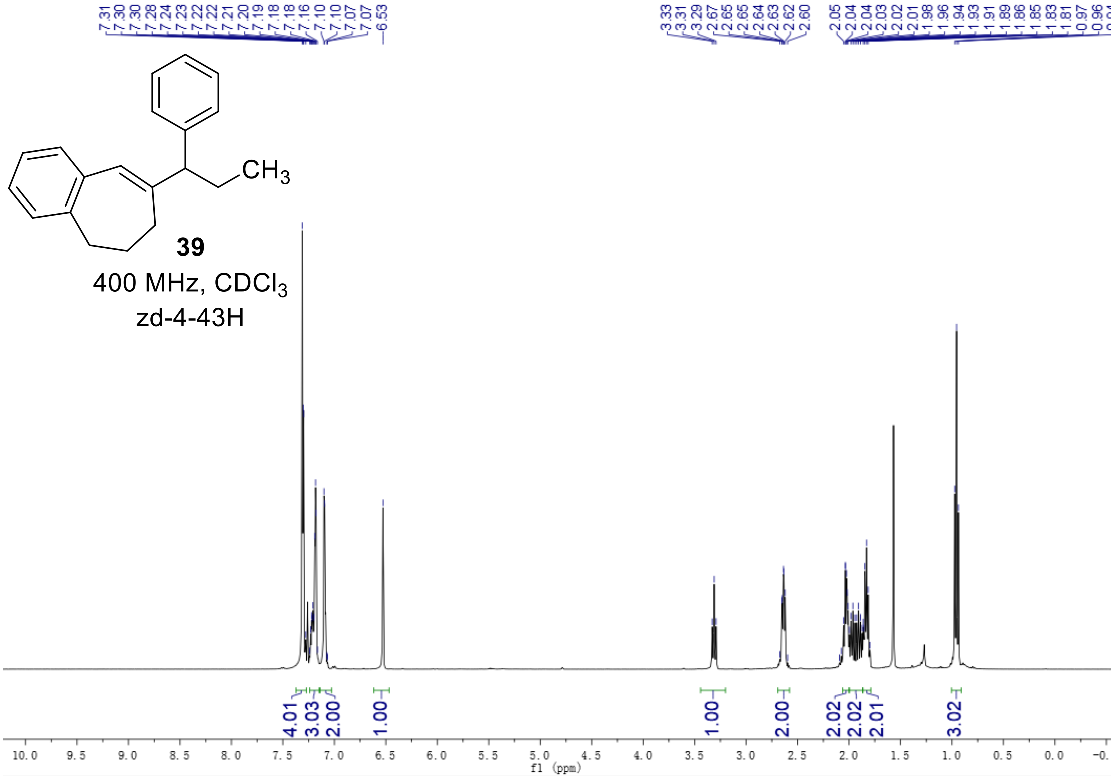

Supplementary Figure 172. ^{1}H NMR (400 MHz, $CDCl_3$, 25 °C) spectra for **39**

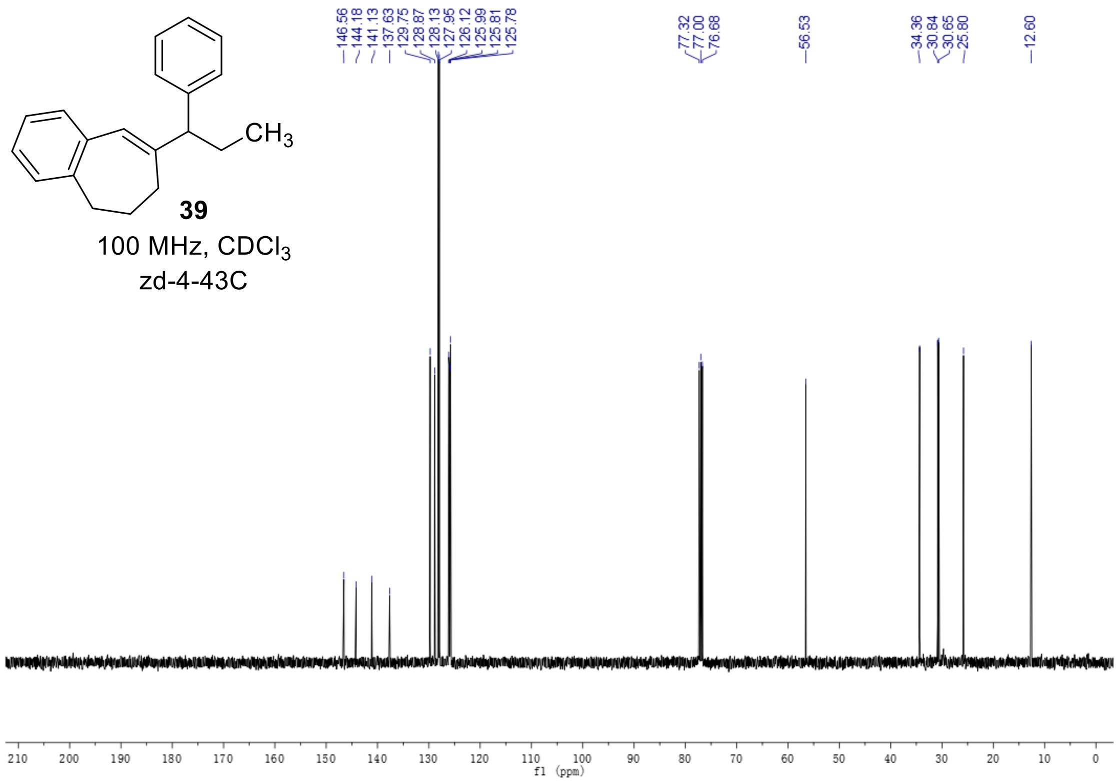

Supplementary Figure 173. ^{13}C NMR (100 MHz, $CDCl_3$, 25 °C) spectra for **39**

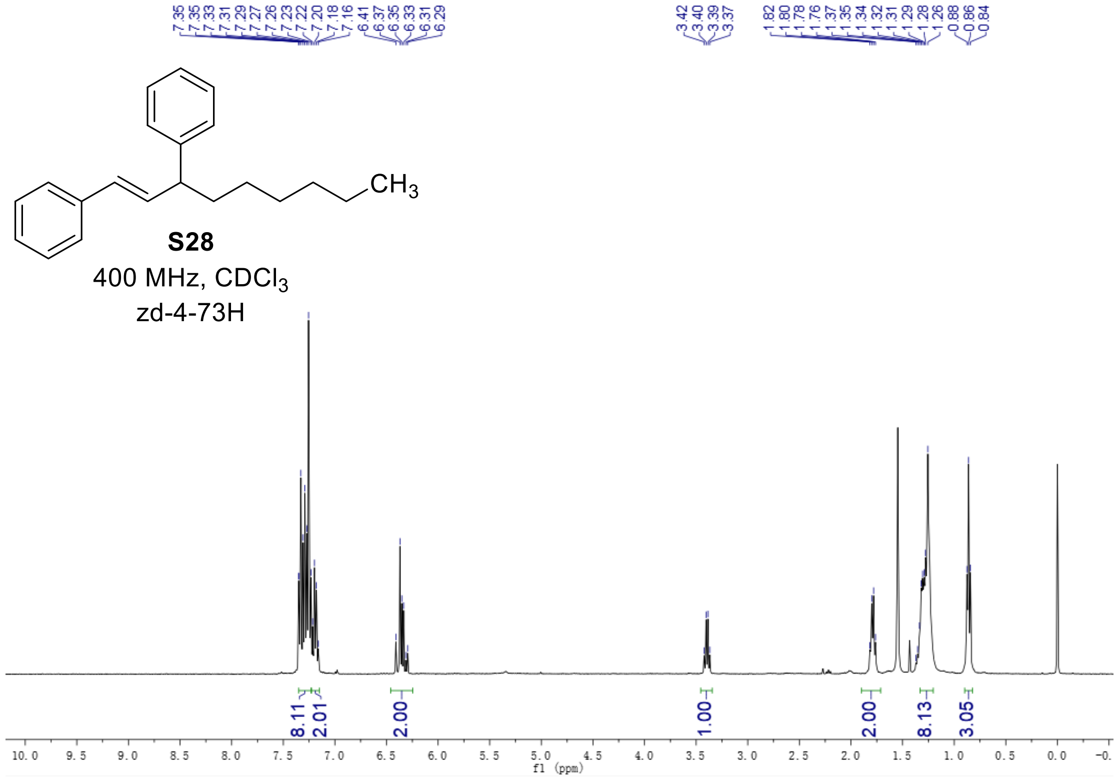

Supplementary Figure 174. ^{1}H NMR (400 MHz, $CDCl_3$, 25 °C) spectra for **S28**

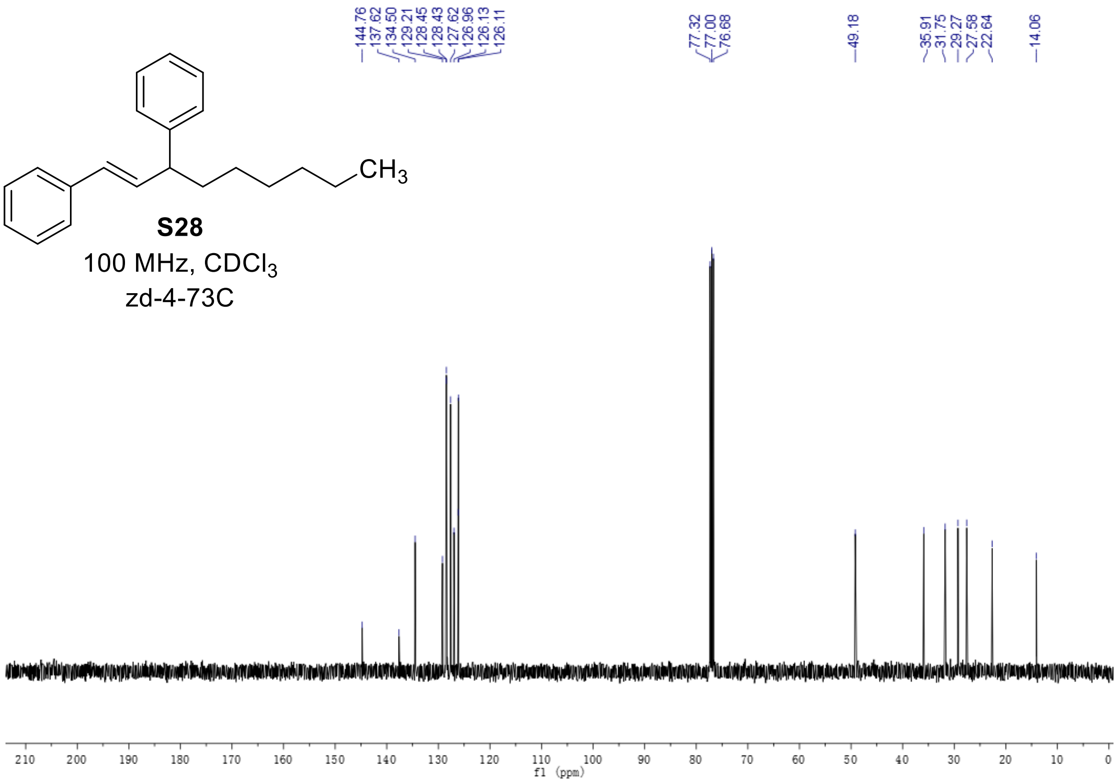

Supplementary Figure 175. ^{13}C NMR (100 MHz, $CDCl_3$, 25 °C) spectra for **S28**

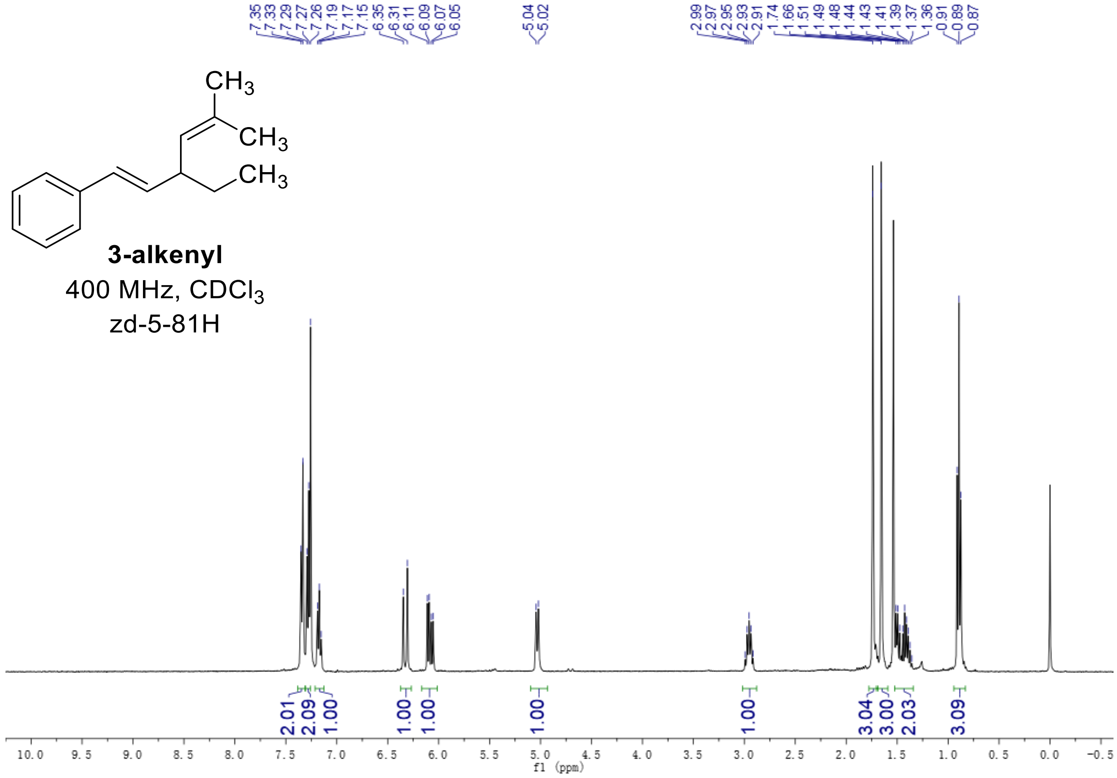

Supplementary Figure 176. ^{1}H NMR (400 MHz, $CDCl_3$, 25 °C) spectra for **3-alkenyl**

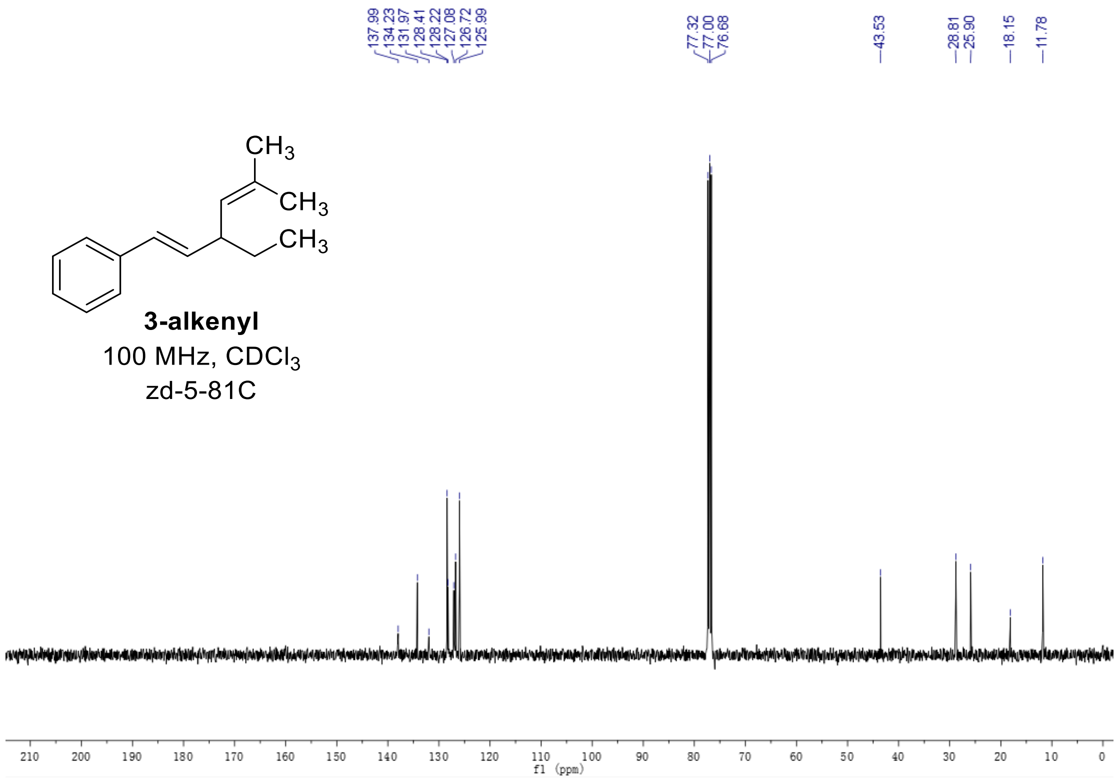

Supplementary Figure 177. ^{13}C NMR (100 MHz, $CDCl_3$, 25 °C) spectra for **3-alkenyl**

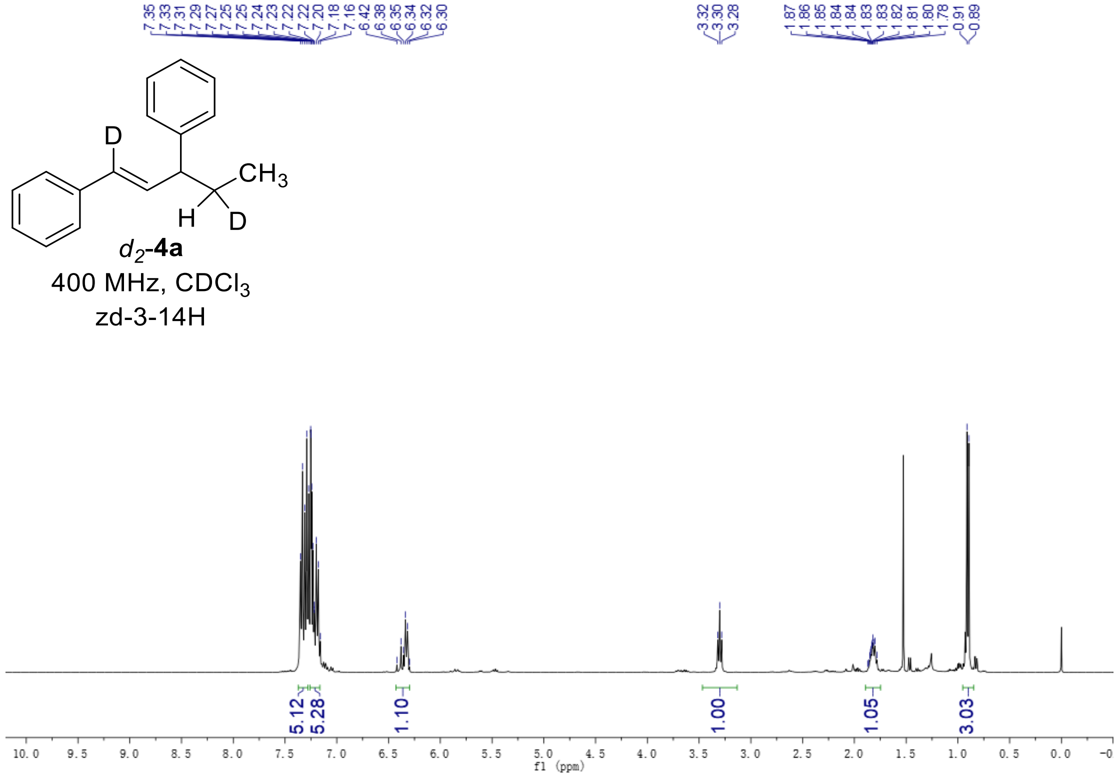

Supplementary Figure 178. ^{1}H NMR (400 MHz, $CDCl_3$, 25 °C) spectra for *d_2*-**4a**

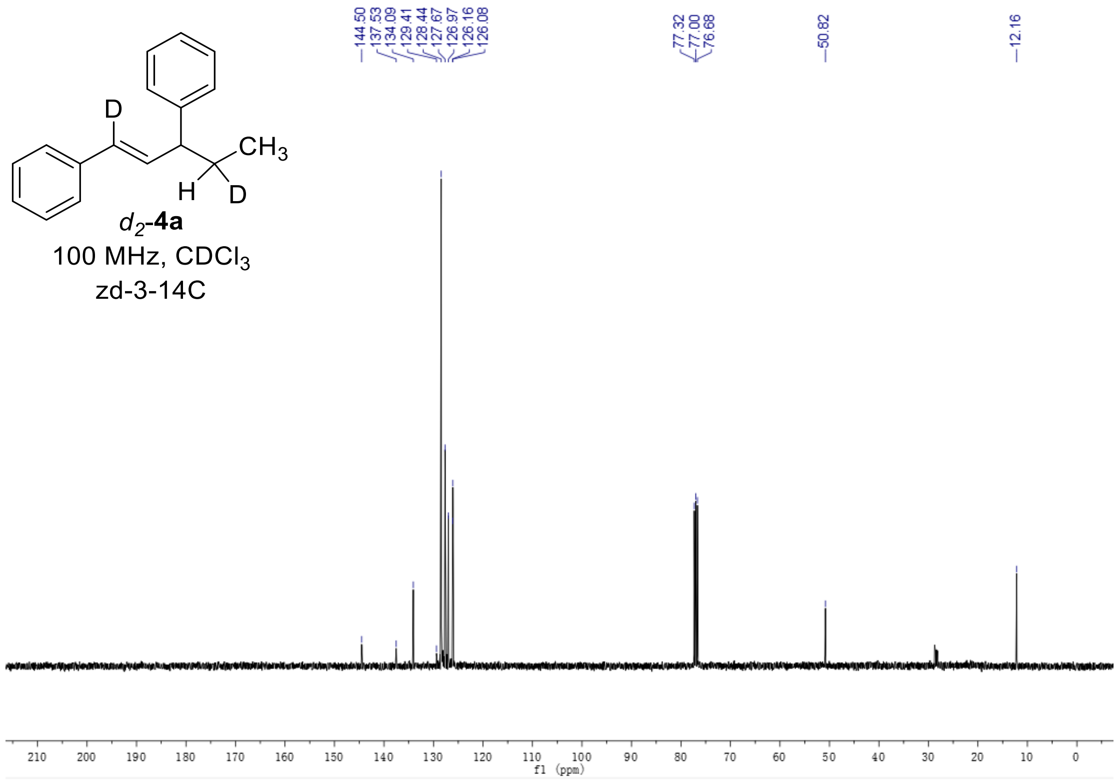

Supplementary Figure 179. ^{13}C NMR (100 MHz, $CDCl_3$, 25 °C) spectra for *d*$_2$-**4a**

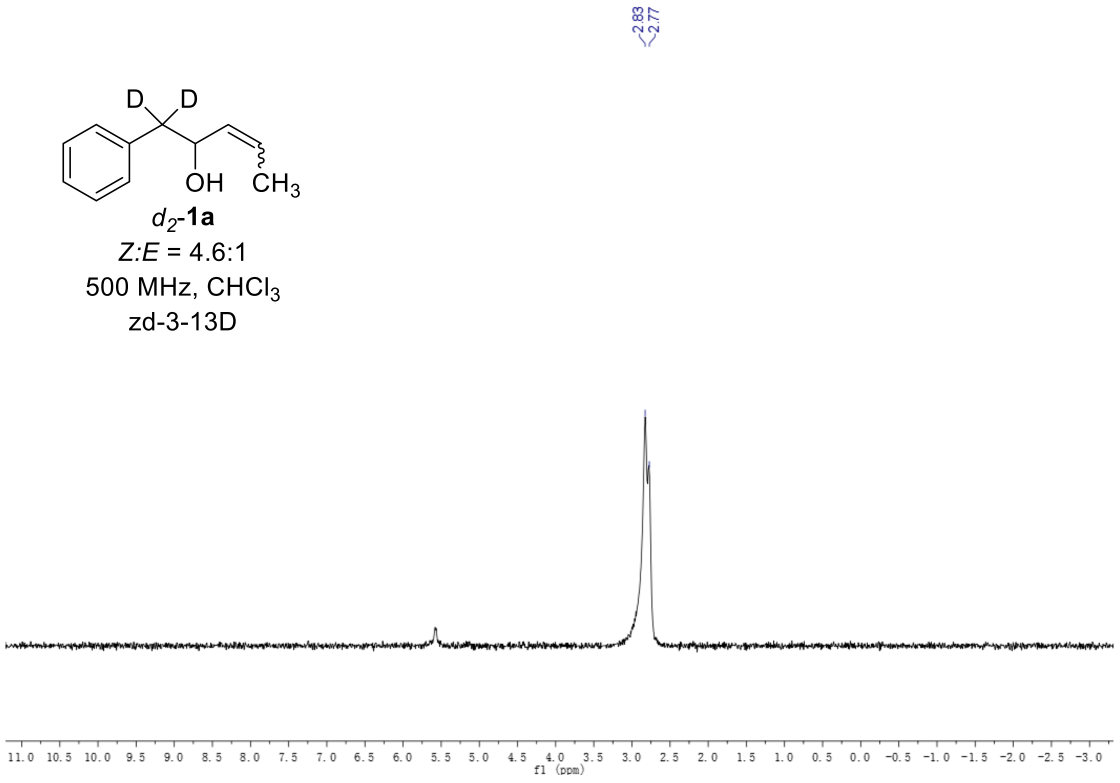

Supplementary Figure 180. ^{2}H NMR (500 MHz, $CHCl_3$, 25 °C) spectra for *d*$_2$-**1a**

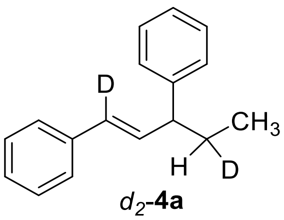

d_2-**4a**
500 MHz, $CHCl_3$
zd-3-14D

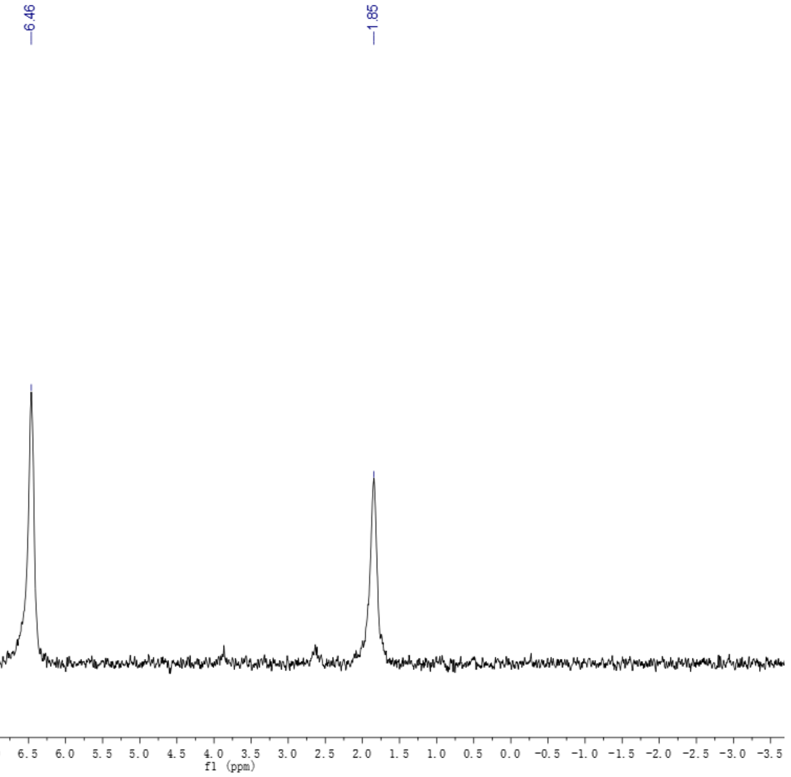

Supplementary Figure 181. ^{2}H NMR (500 MHz, $CHCl_3$, 25 °C) spectra for *d_2*-**4a**

4. Supplementary reference

1. Kohls, H.; Anderson, M.; Dickerhoff, J.; Weisz, K.; Córdova, A.; Berglund, P. & Brundiek, H. Selective Access to All Four Diastereomers of a 1,3-Amino Alcohol by Combination of a Keto Reductase- and an Amine Transaminase-Catalysed Reaction. *Adv. Synth. Catal.* **357**, 1808–1814, (2015).

2. Purino, M.; Ramírez, M.; Daranas, A.; Martín, V. & Padrón, J. Iron(III) Catalyzed Direct Synthesis of *cis*-2,7-Disubstituted Oxepanes. The Shortest Total Synthesis of (+)-Isolaurepan. *Org. Lett.* **14**, 5904–5907, (2012).

3. Fegyverneki, D.; Kolozsvári, N.; Molnár, D.; Egyed, O.; Holczbauer, T. & Soós, T. Size-Exclusion Borane-Catalyzed Domino 1,3-Allylic/Reductive Ireland-Claisen Rearrangements: Impact of the Electronic and Structural Parameters on the 1,3-Allylic Shift Aptitude. *Chem. Eur. J.* **25**, 2179–2183, (2019).

4. Yu, B.; Mohamed, S.; Ardisson, J.; Lannou, M. & Sorin, G. MeOTf-induced carboannulation of arylnitriles and aromatic alkynes: a new metal-free strategy to construct indenones. *Chem. Commun.* **50**, 2775–2777, (2014).

5. Ramachandran, P. V.; Pratihar, D. & Biswas, D. Synthesis of 4-substituted homoallylic alcohols via a one-pot tandem Lewis-acid catalyzed crotylboration-[3,3]-sigmatropic rearrangement. *Chem. Commun.* **15**, 1988–1989, (2005).

6. Li, N.; Sun, B.; Liu, S.; Zhao, J. & Zhang, Q. Highly Enantioselective Construction of Dihydrooxazines via Pd-Catalyzed Asymmetric Carboetherification. *Org. Lett.* **22**, 190–193, (2020).

7. Dong, W.; Yang, H.; Yang, W. & Zhao, W. Rhodium-Catalyzed Remote Isomerization of Alkenyl Alcohols to Ketones. *Org. Lett.* **22**, 1265–1269, (2020).

8. Lonca, G. H.; Ong, D. Y.; Tran, T. M. H.; Tejo, C.; Chiba, S. & Gagosz, F. Anti-Markovnikov Hydrofunctionalization of Alkenes: Use of a Benzyl Group as a Traceless Redox-Active Hydrogen Donor. *Angew. Chem. Int. Ed.* **56**, 11440–11444, (2016).

9. Ng, T. W.; Liao, G.; Lau, K. K.; Pan, H. J. & Zhao, Y. Room-Temperature Guerbet Reaction with Unprecedented Catalytic Efficiency and Enantioselectivity. *Angew. Chem. Int. Ed.* **59**, 11384–11389, (2020).

10. Ding, Y.; Long, J.; Sun, F. & Fang, X. J. Nickel-Catalyzed Isomerization/Allylic Cyanation of Alkenyl Alcohols. *Org. Lett.* **23**, 6073–6078, (2021).

11. Stokes, B. J.; Opra, S. M. & Sigman, M. S. Palladium-Catalyzed Allylic Cross-Coupling Reactions of Primary and Secondary Homoallylic Electrophiles. *J. Am. Chem. Soc.* **134**, 11408–11411, (2012).

12. Okamura, T.; Koyamada, K.; Kanazawa, J.; Miyamoto, K.; Iwabuchi, Y.; Uchiyama, M. & Kanoh, N. Synthetic Access to *gem*-Difluoropropargyl Vinyl Ethers and Their Application to Propargyl Claisen Rearrangement. *J. Org. Chem.* **86**, 1911–1924, (2021).

13. Han, L.; Liu, C.; Zhang, W.; Shi, X. X. & You, S. L. Dearomatization of tryptophols via a vanadium-catalyzed asymmetric epoxidation and ring-opening cascade. *Chem. Commun.* **50**, 1231–1233, (2014).

14. Gu, Y.; Huang, W.; Chen, S. & Wang, X. Bismuth(III) Triflate Catalyzed Three-Component Reactions of Indoles, Ketones, and α-Bromoacetaldehyde Acetals Enable Indole-to-Carbazole Transformation. *Org. Lett.* **20**, 4285–4289, (2018).

15. Kyne, R. E.; Ryan, M. C.; Kliman, L. T. & Morken, J. P. Allylation of Nitrosobenzene with Pinacol Allylboronates. A Regioselective Complement to Peroxide Oxidation. *Org. Lett.* **12**, 3796–3799, (2010).

16. Edelstein, E. K.; Namirembe, S. & Morken, J. P. Enantioselective Conjunctive Cross-Coupling of Bis(alkenyl)borates: A General Synthesis of Chiral Allylboron Reagents. *J. Am. Chem. Soc.* **139**, 5027–5030, (2017).

17. Iwai, R.; Suzuki, S.; Sasaki, S.; Sairi, A. S.; Igawa, K.; Suenobu, T.; Morokuma, K. & Konishi, G. Bridged Stilbenes: AIEgens Designed via a Simple Strategy to Control the Non-radiative Decay Pathway. *Angew. Chem. Int. Ed.* **59**, 10566–10573, (2020).

18. Wu, Q.; Wang, L.; Jin, R.; Kang, C.; Bian, Z.; Du, Z.; Ma, X.; Guo, H. & Gao, L. Nickel-Catalyzed Allylic C(sp^2)-H Activation: Stereoselective Allyl Isomerization and Regiospecific Allyl Arylation of Allylarenes. *Eur. J. Org. Chem.* **32**, 5415–5422, (2016).

19. (a) Becke, A. D.; Density-functional thermochemistry. III. The role of exact exchange. *J. Chem. Phys.* **98**, 5648–5652, (1993). (b) Stephens, P. J.; Devlin, F. J.; Chabalowski, C. F. & Frisch, M. J. Ab Initio Calculation of Vibrational Absorption and

Circular Dichroism Spectra Using Density Functional Force Fields. *J. Phys. Chem.* **98**, 11623–11627, (1994). (c) Lee, C.; Yang, W. & Parr, R. G. Development of the Colle-Salvetti correlation-energy formula into a functional of the electron density. *Phys. Rev. B* **37**, 785–789, (1988).

20. Frisch, M. J.; Trucks, G. W.; Schlegel, H. B.; Scuseria, G. E.; Robb, M. A.; Cheeseman, J. R.; Scalmani, G.; Barone, V.; Mennucci, B.; Petersson, G. A.; Nakatsuji, H.; Caricato, M.; Li, X.; Hratchian, H. P.; Izmaylov, A. F.; Bloino, J.; Zheng, G.; Sonnenberg, J. L.; Hada, M.; Ehara, M.; Toyota, K.; Fukuda, R.; Hasegawa, J.; Ishida, M.; Nakajima, T.; Honda, Y.; Kitao, O.; Nakai, H.; Vreven, T.; Montgomery, Jr., J. A.; Peralta, J. E.; Ogliaro, F.; Bearpark, M.; Heyd, J. J.; Brothers, E.; Kudin, K. N.; Staroverov, V. N.; Kobayashi, R.; Normand, J.; Raghavachari, K.; Rendell, A.; Burant, J. C.; Iyengar, S. S.; Tomasi, J.; Cossi, M.; Rega, N.; Millam, J. M.; Klene, M.; Knox, J. E.; Cross, J. B.; Bakken, V.; Adamo, C.; Jaramillo, J.; Gomperts, R.; Stratmann, R. E.; Yazyev, O.; Austin, A. J.; Cammi, R.; Pomelli, C.; Ochterski, J. W.; Martin, R. L.; Morokuma, K.; Zakrzewski, V. G.; Voth, G. A.; Salvador, P.; Dannenberg, J. J.; Dapprich, S.; Daniels, A. D.; Farkas, Ö.; Foresman, J. B.; Ortiz, J. V.; Cioslowski, J. & Fox, D. J. Gaussian 09, Revision A.02; Gaussian, Inc.: Wallingford, CT, (2009).

21. Hay, P. J. & Wadt, W. R. Ab initio effective core potentials for molecular calculations. Potentials for the transition metal atoms Sc to Hg. *J. Chem. Phys.* **82**, 270–283, (2009).

22. Marenich, A. V.; Cramer,C. J. & Truhlar, D. G. Universal Solvation Model Based on Solute Electron Density and on a Continuum Model of the Solvent Defined by the Bulk Dielectric Constant and Atomic Surface Tensions. *J. Phys. Chem. B* **113**, 6378–6396, (2009).

23. Fukui, K. Formulation of the reaction coordinate. *J. Phys. Chem.* **74**, 4161–4163, (2009).
