# Supplementary material for: Migratory allylic arylation of 1,n-enols enabled by nickel catalysis
Source: Nat Commun. 2023 Jun 7;14:3308. doi: 10.1038/s41467-023-38865-z (PMC10247813; doi:10.1038/s41467-023-38865-z)
Supplement: Supplementary file 3 — Description of Additional Supplementary Files [file 41467_2023_38865_MOESM3_ESM.pdf]

Description of Additional Supplementary Files

Supplementary Data 1: Coordination data sets and free energies for DFT optimized structures
