# Supplementary material for: Migratory allylic arylation of 1,n-enols enabled by nickel catalysis
Source: Nat Commun. 2023 Jun 7;14:3308. doi: 10.1038/s41467-023-38865-z (PMC10247813; doi:10.1038/s41467-023-38865-z)
Supplement: Supplementary file 4 — Supplementary Data 1 [file 41467_2023_38865_MOESM4_ESM.pdf]

Supplementary Data 1

Coordination data sets and free energies for DFT optimized structures at PBE0/combined basis set level at 363 K.

Int1a

-1641.164996

C	1.63246500	-2.89910400	-1.01656400
C	2.05073500	-1.66214500	-1.50254500
C	2.09795700	-0.47759300	-0.70262500
C	2.83517300	-0.29633200	0.60780100
C	4.26699400	0.11117900	0.32210300
C	4.60931000	1.45979700	0.18727100
C	5.91749500	1.83630400	-0.10762100
C	6.90313600	0.86597200	-0.27284300
C	6.57224400	-0.48130900	-0.14145800
C	5.26312400	-0.85462600	0.15111000
H	1.36016700	-3.61942600	-1.78835600
H	1.98886600	-1.52768000	-2.58281100
H	2.10071000	0.43862300	-1.29253500
H	2.82811500	-1.20276100	1.21579000
H	3.84578100	2.22413900	0.32230300
H	6.16809100	2.89018800	-0.20230800
H	7.92555000	1.15771600	-0.49942000
H	7.33723400	-1.24463400	-0.26225600
H	2.34746200	0.49650000	1.18606100
H	5.01513300	-1.90933300	0.25559200
Ni	0.28816300	-1.31843800	-0.62363400
P	-1.21101000	0.22435000	0.03356400
C	1.77299300	-3.48942600	0.35913700
H	1.11680900	-4.35960000	0.45884700
H	2.80165900	-3.84183000	0.52192800
H	1.53653700	-2.79597200	1.17435300
C	-2.57700800	-1.05148300	-0.03915200
C	-3.93015500	-0.64233400	-0.62313900
C	-1.95920900	-2.22992000	-0.81196400
H	-2.72671100	-1.37489500	1.00135900
C	-4.86719800	-1.84675100	-0.73710700
H	-3.79032500	-0.20679800	-1.62210400
H	-4.38907500	0.13249100	0.00105000
C	-2.91142900	-3.42011200	-0.95674100
H	-1.64905200	-1.90192100	-1.82004200
H	-1.06497600	-2.62387900	-0.25616700
C	-4.24252400	-2.97388400	-1.55534900
H	-5.81480500	-1.52923500	-1.18942400
H	-5.10942900	-2.21755000	0.26957900
H	-2.44428800	-4.19608600	-1.57496400
H	-3.08491900	-3.86068600	0.03498100
H	-4.92719200	-3.82898000	-1.60990300
H	-4.08380300	-2.63254700	-2.58901400
C	-1.55976800	1.57543900	-1.20086800
C	-2.53888500	2.67003100	-0.75425700
C	-0.25040200	2.19511600	-1.71139000
H	-2.01331900	1.03262100	-2.04477900
C	-2.79401300	3.67095500	-1.88242300

H	-2.11521000	3.20534600	0.10662600
H	-3.48908900	2.23494600	-0.42690300
C	-0.50905700	3.21593800	-2.81920300
H	0.28068300	2.68175000	-0.87960700
H	0.40621100	1.40501100	-2.09021800
C	-1.49644300	4.29440600	-2.38539600
H	-3.48270600	4.44806500	-1.52771000
H	-3.30188800	3.15878400	-2.71306800
H	0.44219400	3.66625400	-3.12998400
H	-0.90842300	2.69220300	-3.70032700
H	-1.70068500	4.97911100	-3.21789100
H	-1.04821200	4.89966800	-1.58356500
C	-1.15481000	0.88387400	1.76826300
C	-2.51487300	1.26492800	2.37255300
C	-0.44922100	-0.14205400	2.67013300
H	-0.52463300	1.78519600	1.70912400
C	-2.35206200	1.79516800	3.79895900
H	-3.16050800	0.37557200	2.39665500
H	-3.02559200	2.01193900	1.75975000
C	-0.29711800	0.37711900	4.09982700
H	-1.03561200	-1.07448700	2.68330600
H	0.53426000	-0.39539700	2.25845800
C	-1.63834600	0.79088700	4.69787100
H	-3.33988600	2.04168100	4.20826300
H	-1.78201400	2.73563600	3.77309100
H	0.17916100	-0.39248900	4.72039900
H	0.38422300	1.24074400	4.09565300
H	-1.49272700	1.21287900	5.70010600
H	-2.27181600	-0.10011000	4.82205100

Int2a

-1641.157025

C	0.20885700	-1.35155500	2.74882100
C	1.07065100	-2.37848200	2.25341500
C	2.34617300	-2.15349600	1.74236600
C	3.38887400	-1.12890000	2.11100000
C	3.57705800	-0.18574300	0.94196900
C	2.82412600	0.99522300	0.85809600
C	2.98416500	1.86378100	-0.22500900
C	3.87889500	1.55256900	-1.24408900
C	4.61788600	0.37012600	-1.17705900
C	4.46678400	-0.49102400	-0.09463300
H	-0.83352500	-1.66577600	2.80527400
H	0.58640000	-3.29917600	1.92608300
H	2.71961700	-2.93899100	1.08334900
H	4.32932300	-1.65385300	2.32205600
H	2.17502500	1.28067900	1.68364200
H	2.42844800	2.79759600	-0.25468600
H	4.01472700	2.23400900	-2.07979000
H	5.32764700	0.12758500	-1.96395500
H	3.12419200	-0.57548000	3.01383800
H	5.06177100	-1.40026700	-0.03890500
Ni	0.80706900	-0.96161400	0.87325000
P	-0.93443100	0.09792000	-0.09240900
C	0.53322900	-0.28467300	3.75951800
H	1.42006600	0.31242400	3.53931600
H	-0.31410800	0.39677400	3.87229800
H	0.69759100	-0.75827300	4.73877600
C	-0.43260000	-0.29868500	-1.85208000
C	0.01848500	0.88738700	-2.70882300

C	0.70040000	-1.33470100	-1.73521200
H	-1.29059200	-0.77568100	-2.34541500
C	0.49745900	0.41350800	-4.08271600
H	0.84446200	1.41169200	-2.20833500
H	-0.79757300	1.60912900	-2.82709900
C	1.21875500	-1.79116500	-3.09985400
H	1.55315600	-0.86925500	-1.18902400
H	0.36378600	-2.21306200	-1.16276100
C	1.62824200	-0.60576400	-3.96784900
H	0.82456400	1.27917900	-4.67241700
H	-0.34673700	-0.03460800	-4.62728800
H	2.05894500	-2.48379700	-2.96564100
H	0.41981600	-2.35623900	-3.60149200
H	1.92717600	-0.95536100	-4.96393700
H	2.51065600	-0.12140700	-3.52535100
C	-1.08906400	1.94070300	0.12931300
C	-2.31515900	2.63429100	-0.47634100
C	-0.94560300	2.32207600	1.60982400
H	-0.19482600	2.31524100	-0.39249100
C	-2.20365200	4.15256400	-0.32600100
H	-3.21739800	2.30007400	0.05268300
H	-2.44165800	2.36670900	-1.53151400
C	-0.87294200	3.84002600	1.78702500
H	-1.80433800	1.92968700	2.17465000
H	-0.04730700	1.85890800	2.03246700
C	-2.05630900	4.55218100	1.13895900
H	-3.08895300	4.62922200	-0.76558900
H	-1.33644100	4.51450200	-0.89798100
H	-0.81908900	4.08169500	2.85640000
H	0.06146700	4.20766800	1.33705500
H	-1.93886800	5.63928100	1.22917500
H	-2.97878200	4.29216100	1.67918100
C	-2.56470700	-0.76527400	0.17362100
C	-3.68014100	-0.44568700	-0.83396600
C	-2.33676000	-2.28621900	0.20838700
H	-2.89091400	-0.44142200	1.17463800
C	-4.96989200	-1.18797000	-0.47434100
H	-3.36082600	-0.75399200	-1.83964500
H	-3.88096900	0.62623900	-0.88294800
C	-3.62010300	-3.03438800	0.56888200
H	-1.99330400	-2.61832300	-0.78291800
H	-1.54014300	-2.54113600	0.91624000
C	-4.75557700	-2.69545700	-0.39183100
H	-5.74310200	-0.95096300	-1.21619400
H	-5.34135800	-0.81658200	0.49232500
H	-3.42751200	-4.11486600	0.56942400
H	-3.91460300	-2.76769300	1.59495400
H	-5.68060700	-3.19614800	-0.07954800
H	-4.51194400	-3.08323600	-1.39211700

Ts1a

-1641.131439

C	0.20038500	-3.09511200	-1.56474300
C	1.15067500	-2.41975800	-2.31320600
C	2.27868600	-1.64864300	-1.86624200
C	2.70175200	-1.36215800	-0.54435700
C	3.88522900	-0.50893000	-0.28045600
C	4.20363500	0.60307900	-1.07036500
C	5.34385100	1.35093000	-0.80288200
C	6.17881900	1.00470900	0.25964900

C	5.86340700	-0.09184300	1.05686100
C	4.72071900	-0.84132900	0.79147100
H	-0.69779400	-3.35716100	-2.12262700
H	0.90703300	-2.25602800	-3.36216400
H	2.75717000	-1.06891000	-2.65424300
H	2.49603200	-2.07633900	0.25012100
H	3.54901300	0.89754300	-1.88737300
H	5.57839200	2.21384700	-1.42090200
H	7.06796100	1.59399700	0.46796700
H	6.50620700	-0.36539900	1.88947100
H	1.52365200	-0.22403500	-0.00729100
H	4.48056200	-1.69960000	1.41590600
Ni	0.61707400	-0.93015000	-0.96903700
P	-1.05412800	0.10600200	0.05277900
C	0.33885600	-3.84456200	-0.27929000
H	1.25476500	-3.64481600	0.27760700
H	-0.52299500	-3.67745100	0.37522500
H	0.33341400	-4.91784900	-0.52269000
C	-1.01810600	1.84672900	-0.62183300
C	-1.53385400	2.94354300	0.31835400
C	0.38445300	2.19721900	-1.14217100
H	-1.69118700	1.79388200	-1.49219700
C	-1.50316600	4.31259400	-0.36430600
H	-0.90430400	2.97953400	1.21878000
H	-2.55316000	2.72117800	0.64918100
C	0.41363200	3.57055000	-1.81161400
H	1.10038200	2.18407000	-0.30704100
H	0.71887100	1.42547000	-1.85338400
C	-0.10940400	4.65845500	-0.87846400
H	-1.85270800	5.07711700	0.34103500
H	-2.21408400	4.31473600	-1.20391600
H	1.43762500	3.80038900	-2.13281300
H	-0.20244100	3.54321800	-2.72265000
H	-0.12312900	5.62643300	-1.39479900
H	0.57692300	4.76724400	-0.02555900
C	-0.75288900	0.13504700	1.89254900
C	-1.92124700	0.53880500	2.80136200
C	-0.17939700	-1.21401300	2.35438700
H	0.04589800	0.88822800	1.99162600
C	-1.48426200	0.59263000	4.26730300
H	-2.72820700	-0.20034600	2.70191200
H	-2.33490400	1.50769400	2.50743300
C	0.24518300	-1.17231000	3.82235500
H	-0.94036700	-1.99785200	2.22100700
H	0.67805800	-1.48959000	1.72828000
C	-0.90007800	-0.73763900	4.73061000
H	-2.34319400	0.87259500	4.89026800
H	-0.73405600	1.38727200	4.39341500
H	0.62084000	-2.15869400	4.12367400
H	1.08469700	-0.46974900	3.93276500
H	-0.55442500	-0.66273200	5.76930900
H	-1.68787300	-1.50572000	4.71691400
C	-2.75349100	-0.56236900	-0.31669100
C	-3.94163300	0.34789500	0.03242800
C	-2.84993600	-0.96642600	-1.79678700
H	-2.82073100	-1.47796000	0.29186300
C	-5.26810400	-0.36319000	-0.24907200
H	-3.88981400	1.25790700	-0.58151900
H	-3.91382000	0.66641700	1.07757800
C	-4.17104700	-1.67627600	-2.09320100

H	-2.77558200	-0.06384600	-2.42266500
H	-2.00713000	-1.60863500	-2.07298300
C	-5.37155900	-0.82089200	-1.70036300
H	-6.09962900	0.30842000	-0.00052900
H	-5.35925900	-1.23224900	0.41917600
H	-4.21748100	-1.93654300	-3.15847400
H	-4.19957900	-2.62502200	-1.53638900
H	-6.30366300	-1.37791400	-1.85847500
H	-5.41879200	0.06100100	-2.35619100

Int3a

-1641.145964

C	0.34559300	-2.36380700	-2.41112500
C	1.04152700	-1.24387000	-2.78806600
C	2.29379400	-0.73105900	-2.25792800
C	2.88491900	-1.11437000	-1.07179400
C	4.06427300	-0.48682200	-0.46790300
C	4.61238900	0.72291200	-0.92342200
C	5.74649200	1.25164500	-0.32283100
C	6.35580000	0.58764100	0.74301700
C	5.82057000	-0.61087900	1.20681000
C	4.68354400	-1.14157300	0.60707600
H	-0.65889700	-2.44562200	-2.82360100
H	0.55738300	-0.58378100	-3.50726300
H	2.68991600	0.13871500	-2.77628800
H	2.56705300	-2.03168300	-0.57738000
H	4.14930500	1.26414600	-1.74414100
H	6.15823400	2.19009000	-0.68515200
H	7.24315100	1.00678700	1.21017100
H	6.28861700	-1.13495200	2.03589100
H	1.16867000	0.28531400	0.30165800
H	4.27130300	-2.08109900	0.97008100
Ni	0.73870600	-0.48677300	-0.85504600
P	-1.07093500	0.16258900	0.19835300
C	0.84222000	-3.59641100	-1.73176800
H	1.93180800	-3.64503100	-1.66724000
H	0.42167900	-3.72363600	-0.72665300
H	0.50062700	-4.46385900	-2.31098700
C	-0.97859800	1.95959600	0.65657100
C	-0.05692400	2.24013800	1.84907500
C	-0.55485400	2.78285000	-0.56885000
H	-1.99843000	2.25315500	0.94157500
C	0.00990700	3.73814700	2.15548300
H	0.95631200	1.87291500	1.62793200
H	-0.40407400	1.70366500	2.73949700
C	-0.47833400	4.27545900	-0.24905600
H	0.43201100	2.42713000	-0.90464100
H	-1.25004700	2.61977800	-1.40128300
C	0.43990800	4.54928300	0.93755800
H	0.70091800	3.90446200	2.99147700
H	-0.97844700	4.08301300	2.49386500
H	-0.13535900	4.82196900	-1.13674200
H	-1.48799600	4.64878900	-0.02305300
H	0.44364600	5.62022800	1.17625300
H	1.47353900	4.28387200	0.66914600
C	-1.18845700	-0.81066600	1.77250800
C	-2.42698700	-0.54910000	2.63571400
C	-1.01963900	-2.30858900	1.49055000
H	-0.29978000	-0.48202100	2.33167900
C	-2.35704500	-1.35078600	3.93672100

H	-3.32919800	-0.85020200	2.08660400
H	-2.52708000	0.52060800	2.85843700
C	-0.97987200	-3.11742000	2.78729000
H	-1.84839900	-2.66581800	0.86127100
H	-0.09355100	-2.46825300	0.92221000
C	-2.19923000	-2.84393300	3.66377400
H	-3.26145800	-1.16345900	4.52931300
H	-1.50805800	-0.99555800	4.53915100
H	-0.90979500	-4.18697700	2.55128400
H	-0.06698400	-2.85848100	3.34407600
H	-2.11692600	-3.39619800	4.60814600
H	-3.10289600	-3.21797700	3.15981000
C	-2.67553700	-0.07675300	-0.73735100
C	-3.82847400	0.82831200	-0.27491500
C	-2.51612000	0.03816300	-2.25946800
H	-2.94119100	-1.12142500	-0.50876400
C	-5.13140200	0.43787800	-0.97685600
H	-3.59203800	1.87149100	-0.52634100
H	-3.96494100	0.78663200	0.81022600
C	-3.81357200	-0.34203900	-2.97404300
H	-2.24123200	1.06858300	-2.52499600
H	-1.69888300	-0.60113100	-2.60456700
C	-4.99202200	0.50038600	-2.49470100
H	-5.93829600	1.10064600	-0.63912800
H	-5.41590400	-0.58085900	-0.67407500
H	-3.68223800	-0.23492000	-4.05833100
H	-4.02606500	-1.40551700	-2.78745600
H	-5.91922100	0.16566400	-2.97645800
H	-4.83972600	1.54561900	-2.80163800

Int3a-p1

-1215.425622

H	-0.21354900	-0.87176700	2.60237500
Ni	-0.41583300	0.51999500	2.25597300
P	0.08180000	-0.13465700	0.30605100
C	-1.18592200	-1.24092600	-0.45222300
C	-0.90468700	-2.71905300	-0.14746700
C	-2.59326000	-0.84336300	0.02227800
H	-1.11551900	-1.08302200	-1.53886900
C	-1.98329800	-3.61689000	-0.75607200
H	-0.87771200	-2.86724000	0.94213700
H	0.07520300	-3.01682100	-0.53776000
C	-3.66000600	-1.75022100	-0.59185000
H	-2.62831600	-0.92598100	1.11914600
H	-2.81002900	0.20353700	-0.22396700
C	-3.38271200	-3.22109300	-0.29695800
H	-1.77313800	-4.66058400	-0.49136000
H	-1.92672400	-3.55566000	-1.85270600
H	-4.64643600	-1.45955800	-0.20942600
H	-3.68608000	-1.59220200	-1.67993600
H	-4.13400900	-3.85346800	-0.78590600
H	-3.47696500	-3.40121500	0.78407000
C	1.76230600	-0.85036800	0.09416600
C	2.16964900	-0.95417600	-1.38246200
C	2.80913700	-0.08533400	0.91439400
H	1.68289900	-1.86307800	0.51426900
C	3.54878200	-1.60331900	-1.51296800
H	2.20040200	0.05227300	-1.82396300
H	1.42845200	-1.53130100	-1.94910300
C	4.18308100	-0.74137400	0.77004400

H	2.86857600	0.95675900	0.56825700
H	2.51066400	-0.05886500	1.97071700
C	4.60094300	-0.86130900	-0.69353000
H	3.83711400	-1.63326800	-2.57105900
H	3.48877400	-2.64777300	-1.17397900
H	4.92527200	-0.16322200	1.33429100
H	4.15150500	-1.74181000	1.22561600
H	5.56725100	-1.37528400	-0.76809000
H	4.74817600	0.14423800	-1.11454200
C	0.02452800	1.57464100	-0.41669400
C	-0.62312000	1.73033700	-1.79557600
C	-0.66326000	2.46485700	0.64026700
H	1.07588300	1.88885300	-0.48458000
C	-0.65494600	3.20231200	-2.21127800
H	-1.65021100	1.34508100	-1.77268200
H	-0.07642600	1.13828200	-2.53906200
C	-0.71889700	3.93063700	0.20272500
H	-1.69013500	2.10776100	0.82256500
H	-0.09294100	2.44122000	1.59998300
C	-1.36643300	4.06638400	-1.17269900
H	-1.15145200	3.29278300	-3.18513500
H	0.37391400	3.56576800	-2.34785600
H	-1.26505300	4.51767600	0.95049000
H	0.30431600	4.33000000	0.16822300
H	-1.35493200	5.11801700	-1.48341000
H	-2.42375900	3.76942500	-1.11133800

Int3a-p2

-425.703972

C	-3.77733100	-0.02325800	-0.05031600
C	-2.90547100	-0.95287700	-0.48380100
C	-1.45885800	-0.98774400	-0.31178400
C	-0.62856100	0.07491300	-0.29371700
C	0.82429400	0.04084100	-0.12000500
C	1.52040200	-1.09256900	0.33355400
C	2.90261200	-1.07307100	0.46695200
C	3.62693600	0.07908900	0.16003600
C	2.95167600	1.21524100	-0.27859700
C	1.56764900	1.19539700	-0.41296700
H	-4.82803500	-0.18647100	-0.29212700
H	-3.32359200	-1.81921000	-0.99844700
H	-1.02925300	-1.98813200	-0.24190100
H	-1.04542300	1.06518700	-0.47139000
H	0.97413800	-1.99385700	0.59979700
H	3.41995700	-1.96130200	0.82214300
H	4.70847200	0.09117800	0.26968600
H	3.50412900	2.12142200	-0.51581600
H	1.04599600	2.08638000	-0.75756500
C	-3.48533000	1.19256600	0.76472000
H	-2.53199800	1.11237700	1.29779300
H	-3.44069300	2.09901200	0.14357200
H	-4.28179200	1.36225000	1.49980100

Int4a

-1641.141380

C	0.53597400	4.09045000	0.00632500
C	1.19657900	3.50525500	1.07395900
C	2.17032800	2.43497700	1.04335200
C	2.49191600	1.66657100	-0.04881000
C	3.40167500	0.52025200	-0.02802600

C	3.73374900	-0.18155700	1.14294700	H	-3.11397100	1.98315900	0.37733200
C	4.63871000	-1.23387400	1.10005400	C	-5.51056000	0.17854000	-1.36845700
C	5.23217900	-1.60570700	-0.10721200	H	-5.00297600	-1.55015900	-2.57454800
C	4.91093800	-0.91980200	-1.27598300	H	-4.24887900	-0.04887500	-3.10139000
C	3.99779600	0.12854300	-1.23721500	H	-5.53291000	1.97074200	-0.14675700
H	-0.27878200	4.75344000	0.28909800	H	-4.56976800	2.10520700	-1.61567300
H	0.85670200	3.78711500	2.06957900	H	-6.38590300	0.45892300	-1.96774400
H	2.56506300	2.14466500	2.01436300	H	-5.88276200	-0.44560900	-0.54240600
H	2.19357100	1.98605300	-1.04681600	C	-1.24504600	-0.52310000	1.74610900
H	3.27849100	0.08670900	2.09226400	C	-2.18166600	-1.71882100	1.96916200
H	4.88314000	-1.76995200	2.01343700	C	0.10574400	-0.72648800	2.45012300
H	5.94322200	-2.42724700	-0.13407500	H	-1.72166700	0.35782500	2.20435000
H	5.37236000	-1.19967700	-2.21926300	C	-2.35439600	-1.99210900	3.46487900
H	-1.22107800	2.41682300	0.20604000	H	-1.77942200	-2.61587000	1.48487500
H	3.75828700	0.67098700	-2.14961700	H	-3.16093900	-1.52755500	1.51857400
Ni	0.14571600	1.95339700	0.29779400	C	-0.08236700	-1.02298200	3.93754800
P	-0.95271900	0.05560800	0.00204400	H	0.64724100	-1.55884900	1.97906000
C	0.95853600	4.18454200	-1.42841900	H	0.72615800	0.17132100	2.31954200
H	0.36993400	3.52668400	-2.08266500	C	-1.01356000	-2.21135700	4.15922000
H	0.77761800	5.20708900	-1.77970300	H	-3.00422000	-2.86554400	3.60143600
H	2.01910300	3.96036400	-1.57247700	H	-2.87232300	-1.14146200	3.93231700
C	-2.58904600	0.27299600	-0.87173400	H	0.89508500	-1.20809500	4.40145400
C	-3.26327200	-0.99109400	-1.42866800	H	-0.50186100	-0.13477500	4.43295800
C	-3.58028300	1.06594100	-0.00519700	H	-1.16393800	-2.37949500	5.23290800
H	-2.31804800	0.91008600	-1.72826700	H	-0.54286600	-3.12284200	3.76208300
C	-4.52938300	-0.62970300	-2.21005200	C	0.13188800	-1.13656300	-0.93182800
H	-3.52717500	-1.67162300	-0.60943500	C	-0.18380600	-2.63447700	-0.81507200
H	-2.58421100	-1.53887300	-2.08900100	C	0.24902700	-0.68654300	-2.39842000
C	-4.83868800	1.42253100	-0.79624200	H	1.11480500	-0.97662700	-0.46303100
H	-3.86731200	0.46807200	0.87067100	C	0.83127000	-3.46467600	-1.60438400

H	-1.19230700	-2.84856700	-1.18731100
H	-0.15886900	-2.94083900	0.23573900
C	1.24099100	-1.55228200	-3.17612300
H	-0.73056700	-0.74818300	-2.89014400
H	0.55308200	0.36839500	-2.44502200
C	0.90128600	-3.03509200	-3.06592100
H	0.56703500	-4.52694500	-1.52675100
H	1.82478000	-3.35369500	-1.14441200
H	1.25302800	-1.23571200	-4.22698800
H	2.25375600	-1.38807100	-2.78333600
H	1.64789800	-3.63363400	-3.60275200
H	-0.06594500	-3.23034100	-3.55287700

Ts2a

-1641.139759

C	0.20679900	-3.52390600	-1.60611200
C	1.04301100	-2.69322000	-2.38103200
C	2.13638700	-1.84445800	-1.98707700
C	2.48016600	-1.50032500	-0.69500500
C	3.48226500	-0.50983300	-0.30879200
C	4.13054200	0.34031000	-1.22195300
C	5.07915600	1.25335100	-0.78274400
C	5.40194000	1.34050400	0.57257800
C	4.77111100	0.50257900	1.48865600
C	3.82052800	-0.41247000	1.05108800
H	-0.60309500	-3.96898400	-2.18221500
H	0.73841400	-2.56747000	-3.42026500
H	2.60294300	-1.30757400	-2.80959400
H	2.10059400	-2.09130700	0.13695800
H	3.89504800	0.29752800	-2.28163100
H	5.57124600	1.90344800	-1.50141100
H	6.14499200	2.05796300	0.91035700
H	5.02159500	0.55844700	2.54468000
H	-0.99269100	-2.34055500	-0.96848000
H	3.33816100	-1.07281100	1.76894900
Ni	0.15026600	-1.41055000	-1.14352000
P	-0.96803700	0.11379000	0.02225500
C	0.57694900	-4.32827500	-0.39323700
H	-0.27619700	-4.45473800	0.27963300
H	0.86158800	-5.33088200	-0.74199500
H	1.41911700	-3.92985100	0.17262600
C	-2.79231200	-0.04356700	-0.32191900
C	-3.66409300	1.14120900	0.11973200
C	-3.02091300	-0.32058300	-1.81772400
H	-3.09890400	-0.93953900	0.24041500
C	-5.14263700	0.87591400	-0.17506400
H	-3.35160500	2.04326600	-0.42451700
H	-3.53595500	1.35308400	1.18468700
C	-4.49710200	-0.57833800	-2.12031500
H	-2.67731800	0.54573000	-2.40370300
H	-2.41869800	-1.17900500	-2.14242300
C	-5.38007200	0.57345100	-1.65075200
H	-5.73656200	1.74388700	0.13841700
H	-5.48634300	0.02635900	0.43355900
H	-4.62727100	-0.74766900	-3.19682400
H	-4.80852700	-1.50601100	-1.61744800
H	-6.43735400	0.33895500	-1.82769800
H	-5.15421400	1.47018400	-2.24663600
C	-0.51023300	1.84366900	-0.52131600
C	-0.61347600	2.93241600	0.55423200

C	0.87584800	1.85932900	-1.17867500
H	-1.25275000	2.06365600	-1.30409700
C	-0.26530900	4.30838800	-0.01816800
H	0.07839200	2.70086300	1.37654200
H	-1.62122200	2.95993900	0.98135400
C	1.22061900	3.23835000	-1.73907600
H	1.63409600	1.56855000	-0.43939700
H	0.91423000	1.10739400	-1.98012500
C	1.11370400	4.32109200	-0.66977500
H	-0.31914100	5.05822200	0.78147500
H	-1.02338100	4.59200100	-0.76346000
H	2.23237200	3.21805300	-2.16427800
H	0.53590100	3.47426200	-2.56753700
H	1.31900300	5.30716500	-1.10507700
H	1.88262100	4.15125300	0.09859300
C	-0.63693500	-0.11866900	1.84184500
C	-1.61535800	0.52626700	2.83339100
C	-0.50808000	-1.62016200	2.14886800
H	0.35534700	0.34219300	1.97587200
C	-1.16726000	0.29080100	4.27763200
H	-2.61043300	0.08106400	2.69651800
H	-1.71559500	1.59990700	2.65141100
C	-0.07966900	-1.86788100	3.59528100
H	-1.47636400	-2.11060200	1.96754000
H	0.20846000	-2.08515600	1.46042400
C	-1.02250500	-1.19518000	4.58775500
H	-1.88826400	0.75612600	4.96169100
H	-0.20507900	0.79736400	4.44573400
H	-0.03067600	-2.94815300	3.78327300
H	0.93876000	-1.47755000	3.74117600
H	-0.66002000	-1.33876000	5.61333700
H	-2.01054200	-1.67627300	4.53438900

Int5a

-1641.168200

C	0.01226500	4.34107200	-0.22425900
C	-0.58559600	3.48229400	-1.31775400
C	-1.70853100	2.65277400	-1.32079600
C	-2.04177000	1.86021900	-0.18833400
C	-3.08764500	0.82860900	-0.17712200
C	-3.73108800	0.36933300	-1.33749500
C	-4.72383700	-0.59987200	-1.26152300
C	-5.09271100	-1.13699500	-0.02814000
C	-4.46790800	-0.68680600	1.13310000
C	-3.47981400	0.28868200	1.05840400
H	1.02736300	3.98135500	0.01026700
H	-0.23623900	3.76063600	-2.31366900
H	-2.12667500	2.38945100	-2.29109000
H	-1.81324700	2.26234600	0.79695000
H	-3.45898100	0.76597900	-2.31172800
H	-5.21419600	-0.93873100	-2.17060000
H	-5.86884700	-1.89595000	0.02651900
H	-4.75780500	-1.08893000	2.10043500
H	0.17691800	5.32116900	-0.69408100
H	-3.00760500	0.64749900	1.97073700
Ni	-0.13703100	1.52416600	-0.78499900
P	0.71744300	-0.39240200	0.06386500
C	-0.76745400	4.55676200	1.06706200
H	-0.73183000	3.69487700	1.74162500
H	-0.33039600	5.40007700	1.61290800

H	-1.81744100	4.79913900	0.86878200
C	2.46028800	0.14961800	-0.31079400
C	3.52147500	-0.91421800	-0.60337100
C	2.31726200	1.12690900	-1.49133700
H	2.77220600	0.72782400	0.57215500
C	4.85152200	-0.27093100	-1.00119600
H	3.18171400	-1.56119700	-1.42374600
H	3.66185700	-1.55730700	0.27219800
C	3.65082500	1.74403900	-1.91982600
H	1.85597400	0.61477800	-2.34893700
H	1.65992300	1.99547300	-1.21774400
C	4.68884700	0.66064400	-2.19923200
H	5.58332900	-1.05616400	-1.22719200
H	5.25369400	0.29597900	-0.14873400
H	3.49944800	2.37461800	-2.80427800
H	4.01474600	2.40024700	-1.11662100
H	5.64871700	1.12643100	-2.45384500
H	4.38146900	0.07570300	-3.07866500
C	0.37019400	-1.92257900	-0.93661200
C	0.84476500	-3.24882700	-0.32995300
C	-1.11933100	-1.99093200	-1.29938700
H	0.93108100	-1.74570400	-1.86852300
C	0.54244200	-4.42074600	-1.26638800
H	0.32802800	-3.41577800	0.62585900
H	1.91793700	-3.21953500	-0.11350200
C	-1.42199800	-3.17565400	-2.21576700
H	-1.71967300	-2.07659600	-0.38177200
H	-1.42380700	-1.05600600	-1.78335000
C	-0.93979400	-4.49362900	-1.61834900
H	0.87274600	-5.35568000	-0.79626900

H	1.13241600	-4.30962700	-2.18828700
H	-2.50036800	-3.21382000	-2.41620800
H	-0.92802400	-3.01551200	-3.18604800
H	-1.12521900	-5.31891200	-2.31737500
H	-1.51854200	-4.71677200	-0.70973400
C	0.57176400	-0.75767000	1.87877600
C	1.77129600	-1.49208800	2.49573000
C	0.29692000	0.54044400	2.65283000
H	-0.31960900	-1.39948000	1.95862200
C	1.53667500	-1.77195100	3.98161300
H	2.66869100	-0.86621100	2.38674200
H	1.97360100	-2.42972600	1.97072300
C	0.07448100	0.26954100	4.14113700
H	1.15204200	1.22346700	2.53164200
H	-0.57634200	1.05015500	2.23058100
C	1.24260500	-0.49346300	4.75913300
H	2.41546500	-2.27939900	4.39928900
H	0.69357900	-2.47023100	4.08904000
H	-0.08625700	1.21886300	4.66795100
H	-0.84909300	-0.31630500	4.26230400
H	1.02833200	-0.72739300	5.80945100
H	2.13724900	0.14682700	4.75830200

Int1b

-1641.172480

C	1.82978500	2.87941300	0.02025100
C	2.11714400	1.87633600	0.93703500
C	2.12370400	0.51193800	0.51075600
C	2.85089800	0.01470400	-0.72136500
C	4.29435600	-0.29474600	-0.37715000

C	4.67119600	-1.58515800	0.00544800	H	-0.87671100	2.46636100	-0.87031400
C	5.99163600	-1.86948600	0.34582700	C	-4.08230100	3.45631300	-0.05549200
C	6.95437200	-0.86358900	0.30967300	H	-5.72988400	2.05806300	0.09419000
C	6.58867900	0.42692800	-0.06898600	H	-4.91772000	2.05725400	-1.46829500
C	5.26790600	0.70875500	-0.40726900	H	-2.21271300	4.50260600	-0.41831800
H	2.05940300	2.71308800	-1.03562600	H	-2.79827200	3.55524900	-1.78695300
H	2.02378200	2.09663200	2.00144000	H	-4.70986700	4.28709900	-0.40047600
H	2.09252400	-0.22314800	1.31371500	H	-3.99189800	3.56344200	1.03560900
H	2.81286300	0.74621400	-1.53569600	C	-1.67420400	-0.92930700	1.66599000
H	3.92568800	-2.37813000	0.02821900	C	-2.74293600	-2.02985500	1.67692800
H	6.26942800	-2.88048300	0.63403200	C	-0.42487600	-1.38571500	2.43501000
H	7.98623500	-1.08487500	0.57060900	H	-2.07664600	-0.04820600	2.19147800
H	7.33590100	1.21614100	-0.10492300	C	-3.09006000	-2.44286600	3.10841900
H	2.36861500	-0.89982800	-1.08186600	H	-2.36078900	-2.90759800	1.13742000
H	4.99133400	1.71843300	-0.70519800	H	-3.64915800	-1.70112000	1.15772900
Ni	0.38235800	1.35394700	0.07488300	C	-0.77321700	-1.82336800	3.85812200
P	-1.20678500	-0.24394800	-0.00163700	H	0.05513100	-2.22018000	1.90181100
C	1.53447700	4.29053600	0.41390300	H	0.30529900	-0.56984200	2.47065400
H	2.43406600	4.91025500	0.29397600	C	-1.85461500	-2.89889900	3.87756600
H	0.76393300	4.73453600	-0.22644500	H	-3.84307700	-3.24090500	3.08426200
H	1.21039400	4.36579500	1.45701900	H	-3.55425500	-1.59171900	3.62837700
C	-2.48224500	1.01191000	-0.53621700	H	0.13371400	-2.17941400	4.36303000
C	-3.88749100	0.93530400	0.06423000	H	-1.12269100	-0.94712200	4.42463600
C	-1.82252800	2.37724000	-0.26920700	H	-2.12046100	-3.15055300	4.91192500
H	-2.56074300	0.88949500	-1.62647100	H	-1.46267500	-3.82083000	3.42254900
C	-4.74449700	2.12080400	-0.38409000	C	-1.17053000	-1.57339300	-1.29648400
H	-3.82741500	0.93465200	1.16138800	C	-2.54056100	-2.11001400	-1.73901300
H	-4.36900700	-0.00482500	-0.22842800	C	-0.39684800	-1.06290900	-2.52274300
C	-2.69892400	3.55897100	-0.69263800	H	-0.59582200	-2.39471100	-0.84049200
H	-1.58367000	2.48030500	0.80390400	C	-2.38340000	-3.20977500	-2.79184600

H	-3.12745300	-1.28653500	-2.17044000
H	-3.11096100	-2.49677600	-0.89084400
C	-0.23949100	-2.15661700	-3.57878400
H	-0.93980700	-0.21236000	-2.96260700
H	0.58744100	-0.68697600	-2.22422400
C	-1.58880400	-2.72879900	-4.00188400
H	-3.37680600	-3.55940500	-3.10042400
H	-1.87576900	-4.07370400	-2.33765700
H	0.29620400	-1.75327100	-4.44746900
H	0.38890700	-2.96145200	-3.16946800
H	-1.44751100	-3.54965300	-4.71606500
H	-2.16308700	-1.95124500	-4.52737100

Int2b

-1641.169269

C	1.07928700	-1.51504800	-1.64412800
C	1.96742600	-0.47098200	-2.01925600
C	2.67234900	0.23517500	-1.04342000
C	3.26333700	-0.33390500	0.22521200
C	4.77342200	-0.39107800	0.10356100
C	5.57690200	0.57848000	0.70780600
C	6.96277600	0.53920700	0.56873200
C	7.56109900	-0.47189400	-0.17890800
C	6.76684000	-1.44307900	-0.78655500
C	5.38262000	-1.40033100	-0.64866000
H	1.35029400	-2.14317300	-0.79166700
H	1.84697600	-0.01590100	-3.00313800
H	3.09035800	1.19593200	-1.34653100
H	2.86766200	-1.33402400	0.43040200
H	5.11502800	1.36803100	1.29819400
H	7.57526100	1.29849600	1.04887100
H	8.64226800	-0.50468900	-0.28679100
H	7.22751300	-2.23742300	-1.36880200
H	3.00079200	0.30094300	1.08160900
H	4.76853300	-2.16097300	-1.12735100
Ni	0.62090700	0.16489800	-0.67625200
P	-1.46425100	0.01987000	0.15676600
C	0.18345500	-2.16408300	-2.65428700
H	0.67986100	-3.05486400	-3.06653400
H	-0.75752000	-2.50171900	-2.20968200
H	-0.04486000	-1.49592900	-3.49108700
C	-1.20150700	1.55154200	1.18749000
C	-0.72825300	1.26066500	2.61618000
C	-0.12008500	2.34009600	0.42551400
H	-2.11835100	2.15266200	1.23713600
C	-0.35007200	2.55440900	3.34012300
H	0.14635300	0.59168600	2.58927300
H	-1.51043100	0.73741600	3.17716600
C	0.28909700	3.62430900	1.14765100
H	0.82572100	1.72406800	0.37654700
H	-0.42987800	2.56330800	-0.60316100
C	0.71652000	3.34257600	2.58464800
H	-0.00094200	2.31711600	4.35262500
H	-1.24947500	3.17619400	3.45679600
H	1.08991600	4.12698500	0.59203600
H	-0.57472700	4.30461000	1.14271800
H	0.92949600	4.28534800	3.10276500
H	1.65796100	2.77225900	2.58019600
C	-1.99835200	-1.37476000	1.25732900
C	-3.39173700	-1.20508900	1.87528800

C	-1.88094900	-2.72364400	0.53287200
H	-1.25228800	-1.36821300	2.06617500
C	-3.72310400	-2.36962400	2.81012200
H	-4.14254900	-1.17059100	1.07402200
H	-3.46099700	-0.25527900	2.41981600
C	-2.23142800	-3.88472200	1.46486800
H	-2.56072700	-2.73988200	-0.33193500
H	-0.86467600	-2.85487000	0.14261700
C	-3.61114700	-3.71167800	2.09282000
H	-4.73353800	-2.23510600	3.21656500
H	-3.03610500	-2.35402300	3.66902900
H	-2.17796500	-4.82875100	0.90778000
H	-1.47481600	-3.94786800	2.26096900
H	-3.81592100	-4.53381500	2.78980700
H	-4.37949100	-3.76898800	1.30739400
C	-2.84036500	0.34432300	-1.05574400
C	-3.95209800	1.28211500	-0.56390800
C	-2.30232000	0.83681400	-2.40715200
H	-3.27595700	-0.65496200	-1.21441000
C	-5.08240300	1.35989800	-1.59233200
H	-3.53801800	2.29002000	-0.42158700
H	-4.34858300	0.96134300	0.40530800
C	-3.42468800	0.92719300	-3.44115000
H	-1.84830500	1.83032300	-2.27752000
H	-1.50980700	0.16992400	-2.76743200
C	-4.56994400	1.81279500	-2.95667900
H	-5.85871700	2.04549700	-1.22959800
H	-5.55735200	0.37210800	-1.68652900
H	-3.02379100	1.30510900	-4.39041700
H	-3.80522700	-0.08508900	-3.64309500
H	-5.38652500	1.81082700	-3.68961400
H	-4.21817600	2.85264800	-2.88356600

Ts1b

-1641.140451

C	0.21021300	-3.29570400	-0.38836600
C	1.10449400	-3.03101900	-1.40576100
C	2.23808800	-2.15280500	-1.29861800
C	2.70747500	-1.50827100	-0.12962100
C	3.92972100	-0.67072600	-0.13242600
C	4.31004100	0.09971600	-1.23909100
C	5.48154900	0.84590500	-1.20721500
C	6.28779800	0.84132500	-0.06875300
C	5.91251800	0.08717500	1.03922000
C	4.73894500	-0.66095300	1.00899700
H	0.50464400	-3.11745000	0.64741300
H	0.85748800	-3.36271100	-2.41330500
H	2.71641500	-1.88917300	-2.24055600
H	2.48152200	-1.96127600	0.83764000
H	3.68281100	0.12810000	-2.12671900
H	5.76406800	1.44053000	-2.07222400
H	7.20223300	1.42816200	-0.04559400
H	6.53267100	0.07988700	1.93174000
H	1.58337200	-0.19696500	0.07899200
H	4.45150000	-1.25070800	1.87703300
Ni	0.64731000	-1.08985900	-0.66935000
P	-0.98665500	0.21051400	0.04394000
C	-1.02942400	-4.09928800	-0.57948600
H	-0.87960100	-5.10615100	-0.16380900
H	-1.87644300	-3.66632300	-0.03632900

H	-1.29798000	-4.20631100	-1.63474800
C	-0.79839800	1.88307800	-0.76617500
C	-1.36214000	3.06417500	0.03557500
C	0.66009300	2.14787600	-1.16885100
H	-1.38506300	1.78467300	-1.69307400
C	-1.20974600	4.37825000	-0.73296400
H	-0.82576400	3.14355200	0.99146200
H	-2.41810600	2.90178800	0.27359500
C	0.80409200	3.46678100	-1.92712100
H	1.29167600	2.17369500	-0.26840000
H	1.03240600	1.31616000	-1.78427000
C	0.23959500	4.63832200	-1.12905400
H	-1.59524500	5.20350700	-0.12089400
H	-1.83437800	4.34249400	-1.63772700
H	1.86152700	3.63814200	-2.16563100
H	0.27346000	3.39134600	-2.88818600
H	0.31516100	5.56592100	-1.71035800
H	0.84460700	4.78670900	-0.22212600
C	-0.88953300	0.38866900	1.89685900
C	-2.15272800	0.87742100	2.61725100
C	-0.40364200	-0.92800300	2.52367100
H	-0.09329200	1.13748800	2.03525000
C	-1.90384500	1.03270500	4.11895500
H	-2.95952700	0.14729100	2.46430300
H	-2.49895300	1.82954100	2.20491900
C	-0.16857300	-0.78221100	4.02701000
H	-1.15312800	-1.71431200	2.34649800
H	0.52183900	-1.25153100	2.02959200
C	-1.41053000	-0.26597000	4.74735900
H	-2.82808400	1.36675500	4.60714700

H	-1.16033800	1.82684800	4.28273200
H	0.14526600	-1.74746800	4.44504400
H	0.66495100	-0.08325100	4.19123700
H	-1.19622900	-0.11729900	5.81307900
H	-2.20666600	-1.02343100	4.69059200
C	-2.66073000	-0.43221700	-0.45751700
C	-3.83276200	0.55798500	-0.38360300
C	-2.57321900	-1.01858800	-1.87626500
H	-2.85406800	-1.25898300	0.24470600
C	-5.14540800	-0.12256000	-0.78081600
H	-3.64337900	1.39163900	-1.07425100
H	-3.93087500	0.98767900	0.61722100
C	-3.88088100	-1.69935100	-2.28014600
H	-2.35759800	-0.20733500	-2.58824100
H	-1.73727000	-1.72599700	-1.94510700
C	-5.06513400	-0.74290400	-2.17232600
H	-5.96126500	0.61004200	-0.73660100
H	-5.38642500	-0.90276700	-0.04353900
H	-3.79365200	-2.08866600	-3.30265900
H	-4.05231200	-2.56776100	-1.62673500
H	-5.99945300	-1.26687600	-2.41025700
H	-4.95494100	0.05625900	-2.92047500

Int3b

-1641.147387

C	0.48340400	-3.16798600	-0.42770100
C	1.18273800	-2.72016500	-1.51741700
C	2.36598400	-1.87926700	-1.47349100
C	3.00302900	-1.46906200	-0.32024400
C	4.14354300	-0.54968800	-0.26898700

C	4.45373900	0.34486400	-1.30562500
C	5.57526600	1.15784800	-1.22009300
C	6.40474000	1.09995900	-0.09863600
C	6.10030900	0.22707100	0.94203300
C	4.97579500	-0.58748100	0.85874300
H	0.91243300	-3.08638200	0.57255000
H	0.77180700	-2.91251000	-2.50717900
H	2.70419500	-1.48816200	-2.43036700
H	2.80215900	-1.99074400	0.61700100
H	3.80512000	0.42203800	-2.17454900
H	5.80266900	1.84738600	-2.02887600
H	7.28068500	1.74015800	-0.03559000
H	6.73745000	0.17935600	1.82112500
H	1.27697900	0.42285200	0.01043400
H	4.74258600	-1.27199400	1.67168000
Ni	0.83857800	-0.86831400	-0.49639700
P	-0.98365300	0.21405600	0.06222900
C	-0.75295700	-3.99120600	-0.52694900
H	-0.51421900	-5.03179500	-0.26408900
H	-1.51683600	-3.66176700	0.18619800
H	-1.17222400	-3.98762000	-1.53683100
C	-1.08459900	1.94747200	-0.63228000
C	-1.89088400	2.92108100	0.24214300
C	0.28402300	2.54738500	-0.97892200
H	-1.63123700	1.80923600	-1.57729600
C	-2.03112400	4.28426500	-0.43814100
H	-1.38154200	3.05073900	1.20703800
H	-2.88627800	2.52040700	0.45793800
C	0.13675900	3.91271000	-1.65052400
H	0.88250800	2.65494500	-0.06202200
H	0.83836300	1.86452700	-1.63488600
C	-0.67465100	4.88109000	-0.79578900
H	-2.58898900	4.96168700	0.22075700
H	-2.63341200	4.17019500	-1.35172900
H	1.13217100	4.32508200	-1.85854500
H	-0.35788700	3.78315800	-2.62468300
H	-0.80517200	5.83499100	-1.32225700
H	-0.11988700	5.10527000	0.12729600
C	-0.98658300	0.28344700	1.92582000
C	-2.35048000	0.40584700	2.61784700
C	-0.21318800	-0.91712900	2.49521000
H	-0.39864100	1.18985500	2.14035400
C	-2.19050200	0.50485200	4.13663500
H	-2.95814200	-0.47873300	2.38048200
H	-2.89854900	1.27880200	2.25241600
C	-0.06095000	-0.82026100	4.01288400
H	-0.74592300	-1.84658900	2.24385200
H	0.77959500	-0.97374000	2.02615000
C	-1.41271400	-0.67763200	4.70449200
H	-3.18279600	0.57050200	4.60079700
H	-1.66692800	1.43985200	4.38443800
H	0.47050000	-1.70531000	4.38545400
H	0.56799400	0.04873500	4.25556700
H	-1.27537800	-0.55924900	5.78654300
H	-1.99422600	-1.60064000	4.56146900
C	-2.49515000	-0.66257200	-0.59099600
C	-3.82981200	0.08944500	-0.47128000
C	-2.25850300	-1.06323000	-2.05730100
H	-2.55826500	-1.58309200	0.00903700
C	-4.98332700	-0.76542700	-1.00295200

H	-3.78036800	1.01678800	-1.05838900
H	-4.03480100	0.37594900	0.56359900
C	-3.41364600	-1.90591700	-2.59983200
H	-2.16082900	-0.15483800	-2.67076700
H	-1.31172500	-1.60800300	-2.15565500
C	-4.75236900	-1.19018200	-2.44912100
H	-5.92113400	-0.20237900	-0.91362400
H	-5.09386700	-1.65856300	-0.37005500
H	-3.22510900	-2.15104000	-3.65288600
H	-3.45110100	-2.86151400	-2.05599100
H	-5.56979300	-1.83664400	-2.79224200
H	-4.76468700	-0.30005200	-3.09514600

Int4b

-1641.152086

C	-0.39321500	4.02078500	0.52916500
C	-1.04118700	3.67605000	-0.63824700
C	-2.01170200	2.60753100	-0.77781500
C	-2.43510700	1.79163300	0.24072200
C	-3.38685500	0.69035600	0.12511400
C	-3.94473100	0.27149600	-1.09575800
C	-4.86593700	-0.76602100	-1.12975100
C	-5.25261400	-1.40673800	0.04885900
C	-4.71533800	-0.99798400	1.26693400
C	-3.79111200	0.04031200	1.30303100
H	-0.76771100	3.66318600	1.49095300
H	-0.73281500	4.16760100	-1.55980400
H	-2.35138000	2.41620200	-1.79276300
H	-2.16620600	2.04517400	1.26804700
H	-3.67200700	0.76559800	-2.02408400
H	-5.29129100	-1.07552300	-2.08075500
H	-5.97746500	-2.21592800	0.01574300
H	-5.01994200	-1.48366500	2.19019400
H	1.34186400	2.32176500	0.08429200
H	-3.38508600	0.36829200	2.25741600
Ni	-0.04088400	1.95898400	-0.11190800
P	0.91515300	-0.03528500	-0.00658400
C	0.63080400	5.10283400	0.60202200
H	0.16403600	6.01728400	0.99454300
H	1.44102700	4.83958100	1.28930900
H	1.05868400	5.33090600	-0.37853800
C	2.70859500	0.05957500	0.50180200
C	3.38846100	-1.23925200	0.95700100
C	3.53513200	0.75857800	-0.58945500
H	2.67323400	0.73445100	1.37093500
C	4.82370900	-0.96463000	1.41462500
H	3.40228000	-1.97293400	0.14158500
H	2.83266600	-1.69357000	1.78268900
C	4.96553200	1.02048400	-0.11865900
H	3.56469400	0.12957800	-1.48952700
H	3.06111800	1.70551800	-0.88035900
C	5.64659500	-0.26601800	0.33759100
H	5.29705500	-1.91070200	1.70614700
H	4.79997400	-0.33726600	2.31805000
H	5.53538000	1.49173300	-0.92966100
H	4.94806400	1.74177600	0.71204400
H	6.65597000	-0.05095000	0.71039600
H	5.76721900	-0.93906700	-0.52426100
C	0.78907400	-0.71560000	-1.73495300
C	1.58144300	-1.98525900	-2.07709300

C	-0.68847400	-0.86325100	-2.12449700
H	1.20735700	0.10584400	-2.33880700
C	1.42234900	-2.32869300	-3.56022300
H	1.22701500	-2.82854600	-1.47246500
H	2.64346000	-1.85249100	-1.84823800
C	-0.83749500	-1.22335900	-3.60212500
H	-1.15598900	-1.64690600	-1.51279000
H	-1.22419600	0.06862500	-1.90463100
C	-0.04370200	-2.47754500	-3.95562000
H	1.97403300	-3.25155500	-3.78024000
H	1.88527500	-1.53556600	-4.16621800
H	-1.90022600	-1.36220700	-3.83929500
H	-0.48445200	-0.38258400	-4.21786800
H	-0.12795200	-2.68944000	-5.02888300
H	-0.47685200	-3.34196300	-3.43086100
C	-0.05458900	-1.06426900	1.20147900
C	0.11537300	-2.58940500	1.15175400
C	0.15387500	-0.51159900	2.62186800
H	-1.09453100	-0.84515200	0.91483200
C	-0.79403000	-3.26685900	2.18012900
H	1.15628700	-2.86959100	1.35023400
H	-0.12981000	-2.96161200	0.15155700
C	-0.74903500	-1.21744700	3.63359100
H	1.20020900	-0.64752100	2.92623600
H	-0.03695700	0.57112100	2.63766700
C	-0.56064700	-2.73056700	3.58942600
H	-0.62872800	-4.35126900	2.14935200
H	-1.84450200	-3.10191900	1.89789200
H	-0.54095000	-0.83202900	4.63969800
H	-1.79997500	-0.97835300	3.41477700
H	-1.24178200	-3.21771400	4.29836700
H	0.46034200	-2.98132800	3.91375300

Ts2b

-1641.143918

C	0.04809200	3.91320500	0.36080600
C	-0.81899200	3.70446900	-0.73125100
C	-1.93280700	2.80058300	-0.80560500
C	-2.30095000	1.91892000	0.19458300
C	-3.34602300	0.90279600	0.10668000
C	-4.01578800	0.58006700	-1.08626300
C	-5.00547100	-0.39305300	-1.09655600
C	-5.34915500	-1.06384800	0.07864300
C	-4.69974500	-0.75004500	1.26996200
C	-3.70802400	0.22442400	1.28269000
H	-0.29985500	3.67963200	1.36917700
H	-0.54493000	4.18753500	-1.66934700
H	-2.41033900	2.73241900	-1.77994800
H	-1.92718200	2.08685300	1.20588700
H	-3.76904900	1.09254400	-2.01229400
H	-5.51700000	-0.62918300	-2.02601100
H	-6.12501600	-1.82489600	0.06391500
H	-4.96818900	-1.26086600	2.19094400
H	1.15811500	2.53666100	0.25083400
H	-3.21216600	0.47867500	2.21741200
Ni	-0.04348000	1.88811700	-0.33538500
P	0.85070300	-0.11031000	-0.02746800
C	1.11334300	4.96995700	0.29549600
H	0.67599200	5.93291500	0.59007700
H	1.93573500	4.75808200	0.98475300

H	1.52077300	5.07535000	-0.71452600
C	2.66375600	0.05446100	0.36155300
C	3.42054600	-1.18416000	0.85937600
C	3.38808200	0.70788500	-0.82539800
H	2.65955300	0.78699300	1.18361100
C	4.86729300	-0.82377900	1.20652500
H	3.41885200	-1.97006300	0.09502600
H	2.93162400	-1.60208100	1.74500000
C	4.83382500	1.05302200	-0.46859900
H	3.38581400	0.02038300	-1.68250900
H	2.85145800	1.61349300	-1.14134600
C	5.59181300	-0.17385000	0.03219700
H	5.39886300	-1.72686000	1.53198000
H	4.87178000	-0.13302000	2.06272000
H	5.33545400	1.48413000	-1.34444700
H	4.83901200	1.82957800	0.31097100
H	6.61320100	0.10309400	0.32227000
H	5.68648000	-0.90216400	-0.78700100
C	0.62877600	-0.99240200	-1.65653300
C	1.43442000	-2.27485800	-1.90722500
C	-0.86939600	-1.20637200	-1.91972400
H	0.98282900	-0.24021600	-2.38037600
C	1.16223800	-2.81866000	-3.31257000
H	1.17497200	-3.04066700	-1.16636900
H	2.50602600	-2.07802600	-1.80098200
C	-1.11660900	-1.75881000	-3.32290100
H	-1.28106500	-1.90888500	-1.18212500
H	-1.41107100	-0.26150700	-1.78659500
C	-0.32590100	-3.04097400	-3.56376000
H	1.71931300	-3.75384000	-3.45281000
H	1.55358200	-2.10806400	-4.05585500
H	-2.19084700	-1.93584000	-3.46145400
H	-0.82273400	-1.00453200	-4.06826000
H	-0.48795900	-3.40109200	-4.58745700
H	-0.69743800	-3.82920600	-2.89201100
C	-0.02759400	-1.01044700	1.34289200
C	0.13626600	-2.53397400	1.43436900
C	0.28178400	-0.33141800	2.68759800
H	-1.08572400	-0.81338400	1.11057800
C	-0.72046400	-3.09929500	2.56980000
H	1.18592400	-2.79919000	1.60783500
H	-0.15968600	-3.00395900	0.49116000
C	-0.56564500	-0.92124600	3.81557500
H	1.34383500	-0.46300800	2.93531900
H	0.11058300	0.75193400	2.61574100
C	-0.40228800	-2.43577400	3.90589300
H	-0.56834800	-4.18425100	2.63391300
H	-1.78356900	-2.94638000	2.33045700
H	-0.29140700	-0.44661700	4.76633200
H	-1.62464100	-0.68181000	3.63822900
H	-1.04879000	-2.83985200	4.69511400
H	0.63185900	-2.67439000	4.19575600

Int5b

-1641.168128

C	-0.31391700	4.27859800	0.12814300
C	-0.86069900	3.58685900	-1.08872900
C	-1.92797000	2.69718300	-1.15640300
C	-2.17561500	1.81683000	-0.05954800
C	-3.13299300	0.70623000	-0.10381100

C	-3.56553600	0.11355700	-1.30233200
C	-4.50159900	-0.91243200	-1.28634800
C	-5.02138400	-1.37417400	-0.07662800
C	-4.59863300	-0.80033000	1.12003800
C	-3.66202000	0.22788800	1.10591200
H	-0.66556400	3.81239600	1.05425400
H	-0.54490300	4.02428800	-2.03828800
H	-2.37058900	2.49172500	-2.12921900
H	-1.98823000	2.19936700	0.94511200
H	-3.16967200	0.45237300	-2.25591200
H	-4.82850300	-1.35610400	-2.22320100
H	-5.75546900	-2.17574000	-0.06924000
H	-5.00330600	-1.14899100	2.06659800
H	0.78348000	4.20298600	0.12620400
H	-3.34912600	0.68660700	2.04144800
Ni	-0.29948700	1.61485300	-0.72145500
P	0.68664100	-0.28943400	-0.01215100
C	-0.69822300	5.76228200	0.12637200
H	-1.78643400	5.88499900	0.15804000
H	-0.27140900	6.26599800	1.00058100
H	-0.32506100	6.26915000	-0.77085900
C	2.29739300	0.46088800	0.54155100
C	3.36963400	-0.40001500	1.21910200
C	2.87940400	1.25285700	-0.64107300
H	1.96292400	1.20001900	1.28783700
C	4.55626200	0.46720900	1.64721600
H	3.72248800	-1.18433400	0.53895000
H	2.95156900	-0.90480000	2.09693000
C	4.06854400	2.10687800	-0.20375000
H	3.19892000	0.56039700	-1.43179000
H	2.10666000	1.90365000	-1.09680200
C	5.13855300	1.25661400	0.47737500
H	5.32767500	-0.16736000	2.10127200
H	4.22838100	1.16717500	2.43049100
H	4.48762000	2.63105900	-1.07175500
H	3.71844200	2.88081600	0.49525700
H	5.96136800	1.89509800	0.82258900
H	5.57078100	0.55977300	-0.25594400
C	0.94673800	-1.34122200	-1.52820500
C	2.11956100	-2.32855900	-1.55063200
C	-0.37405600	-2.03032700	-1.90947300
H	1.14727300	-0.58315600	-2.30327000
C	2.21034700	-3.03006900	-2.90811800
H	2.00129800	-3.07997700	-0.76025200
H	3.06025700	-1.80393600	-1.35378900
C	-0.26165500	-2.73331900	-3.26204100
H	-0.63968000	-2.77197900	-1.14298100
H	-1.19211300	-1.29926200	-1.93316100
C	0.90267900	-3.71953100	-3.28548300
H	3.03410500	-3.75485500	-2.88747200
H	2.46655500	-2.28956600	-3.68073200
H	-1.20531000	-3.24668500	-3.48645600
H	-0.11906100	-1.98064400	-4.05198700
H	0.99037500	-4.18233500	-4.27660100
H	0.70321600	-4.53595600	-2.57546000
C	-0.08250900	-1.27144300	1.35856600
C	0.51839700	-2.65246100	1.65664700
C	-0.14958400	-0.41461500	2.63150100
H	-1.11560300	-1.42262700	1.00802600
C	-0.26468900	-3.35216800	2.76978700

H	1.56808700	-2.54908900	1.95936700
H	0.50751100	-3.27770500	0.75840600
C	-0.92680600	-1.12888500	3.73786400
H	0.86891200	-0.20560700	2.98770900
H	-0.61321000	0.55664700	2.41307900
C	-0.33694100	-2.50448100	4.03584700
H	0.19785500	-4.32377300	2.98476000
H	-1.28437200	-3.56246800	2.41426000
H	-0.93205900	-0.50804700	4.64281100
H	-1.97587700	-1.24323300	3.42747300
H	-0.93388400	-3.01474300	4.80221900
H	0.67358700	-2.38498700	4.4544200
